# Benzoxazole derivatives as new VEGFR-2 inhibitors and apoptosis inducers: design, synthesis, *in silico* studies, and antiproliferative evaluation

**DOI:** 10.1080/14756366.2022.2103552

**Published:** 2022-07-25

**Authors:** Mohammed S. Taghour, Hazem A. Mahdy, Maher H. Gomaa, Ahmed Aglan, Mahmoud Gomaa Eldeib, Alaa Elwan, Mohammed A. Dahab, Eslam B. Elkaeed, Aisha A. Alsfouk, Mohamed M. Khalifa, Ibrahim H. Eissa, Hazem Elkady

**Affiliations:** a Pharmaceutical Medicinal Chemistry & Drug Design Department, Faculty of Pharmacy (Boys), Al-Azhar University, Cairo, Egypt; b Biochemistry and Molecular Biology Department, Faculty of Pharmacy (Boys), Al-Azhar University, Cairo, Egypt; c Department of Pharmaceutical Organic Chemistry, Faculty of Pharmacy (Boys), Al-Azhar University, Cairo, Egypt; d Department of Pharmaceutical Sciences, College of Pharmacy, Princess Nourah bint Abdulrahman University, Riyadh, Saudi Arabia

**Keywords:** Anticancer, cell cycle, apoptosis, benzoxazole, VEGFR-2

## Abstract

In this study, a set of novel benzoxazole derivatives were designed, synthesised, and biologically evaluated as potential VEGFR-2 inhibitors. Five compounds (**12d**, **12f**, **12i**, **12l**, and **13a**) displayed high growth inhibitory activities against HepG2 and MCF-7 cell lines and were further investigated for their VEGFR-2 inhibitory activities. The most potent anti-proliferative member **12 l (**IC_50_ = 10.50 μM and 15.21 μM against HepG2 and MCF-7, respectively**)** had the most promising VEGFR-2 inhibitory activity (IC_50_ = 97.38 nM). A further biological evaluation revealed that compound **12l** could arrest the HepG2 cell growth mainly at the Pre-G1 and G1 phases. Furthermore, compound **12l** could induce apoptosis in HepG2 cells by 35.13%. likely, compound **12l** exhibited a significant elevation in caspase-3 level (2.98-fold) and BAX (3.40-fold), and a significant reduction in Bcl-2 level (2.12-fold). Finally, docking studies indicated that **12l** exhibited interactions with the key amino acids in a similar way to sorafenib.

## 1. Introduction

Cancer chemotherapy has been considered one of the most important medical advances in the past few decades^1^. However, the narrow therapeutic index besides the unpredictable effects were the major drawbacks of the primary introduced drugs^2^. In contrast, the recently developed targeted therapies gained the advantages of interfering with specific molecular targets almost located in the tumour cells with minimised effect on the normal cells^3^. Thus, these agents provide a high specific therapeutic window with limited non-specific toxicities.

Among the major vital cancer drug targets are tyrosine kinases (TKs) because of their potential role in the modulation of growth factor signalling^4^^,^^5^. Upon their activation, TKs increase both proliferation and growth of tumour cells with induction of apoptosis and reinforcement of angiogenesis and metastasis^6^. Thus, TKs inhibition by different inhibitors became a key approach in cancer management^7^. The evidenced drug ability as well as the safety profile of the FDA-approved TKs inhibitors emphasised the attractiveness of TKs as drug targets.

Owing to their significant participation in modulating angiogenesis, vascular endothelial growth factors (VEGFs) have been considered the key players over other TKs^8^. VEGFs action is performed after their binding to three different tyrosine kinase (TK) receptors, namely, VEGFR-1, VEGFR-2, and VEGFR-3^8^. VEGFR-2 receptor possesses the most crucial role among the rest subtypes as its activation leads to initiation of downstream signal transduction pathway *via* dimerisation followed by autophosphorylation of tyrosine receptor, a pathway resulting finally to angiogenesis^9^. Therefore, hindering VEGF/VEGFR-2 pathway or, even, weakening its response is of considered targets of the recent chemotherapeutic agents^10^. Despite a large number of small molecules with various chemical scaffolds being evidenced to tackle this pathway, resistance development in addition to different adverse effects still the main drawback of the current known VEGFR-2 inhibitors drugs^11^. Thus, the discovery of more effective and less dangerous VEGFR-2 inhibitors becomes an attractive therapeutic target for cancer drug discovery^12^. It has been discovered that VEGFR-2 inhibition in cancer cells causes and expedites apoptosis, which works in concert to enhance the antitumor effect. Hence, the most potent derivative has thoroughly discoursed in our work through the assessment of certain apoptotic markers such as caspase-3 (a crucial component in apoptosis that coordinates the destruction of cellular structures such as DNA and cytoskeletal proteins^13^, BAX and Bcl-2 (members of the Bcl-2 family and core regulators of the intrinsic pathway of apoptosis)^14^.

**Figure 1.**
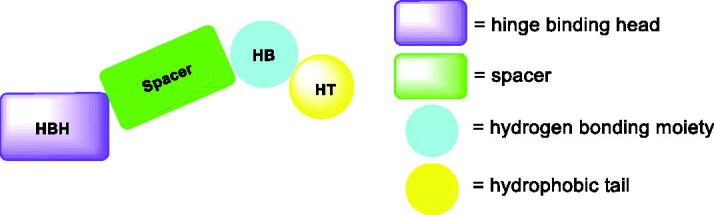
The four main pharmacophoric requirements of VEGFR-2 inhibitors.

Over the last decade, we have built a project that is concerned with cancer management. Our high-throughput efforts gave us the opportunity to identify several small molecules that may serve as anti-angiogenic agents^9^. Most of these molecules exhibited VEGFR-2 inhibitory activity comparable to that of the FDA-approved inhibitor, sorafenib. These molecules were precisely designed to resemble the four main structural parts of sorafenib and other VEGFR-2 inhibitors^15–17^. Those parts were well-known to be a hydrophobic hinge binding head, a linker, a hydrogen-bonding moiety, and a hydrophobic tail (Figure 1). These previously mentioned parts enabled the designed compounds to fit perfectly in the TK active pocket. Based on the promising biological results in our former published work in which we utilised benzoxazole moieties as a hinge-binding core^18^, we decided to continue our preliminary VEGFR-2 studies using the same three different scaffolds of benzoxazole but with two main considerable additional modifications; a) For the allosteric hydrophobic pocket, we used different terminal aliphatic hydrophobic moieties including cyclopentyl (compounds **12a-c**) and ter-butyl moiety (compounds **12d-f**). This allowed us to make a comparative study between aliphatic and aromatic derivatives of each scaffold and study the SAR of the obtained compounds as anticancer leads with significant VEGFR-2 inhibitory potentialities, as was planned in our design. b) The pharmacophore moiety was selected to be amide derivative (compounds **12a-l**) or diamide derivatives (compounds **13a-c**) to study which derivative is more preferred biologically.

### 1.1. Rationale and design

**Figure 2.**
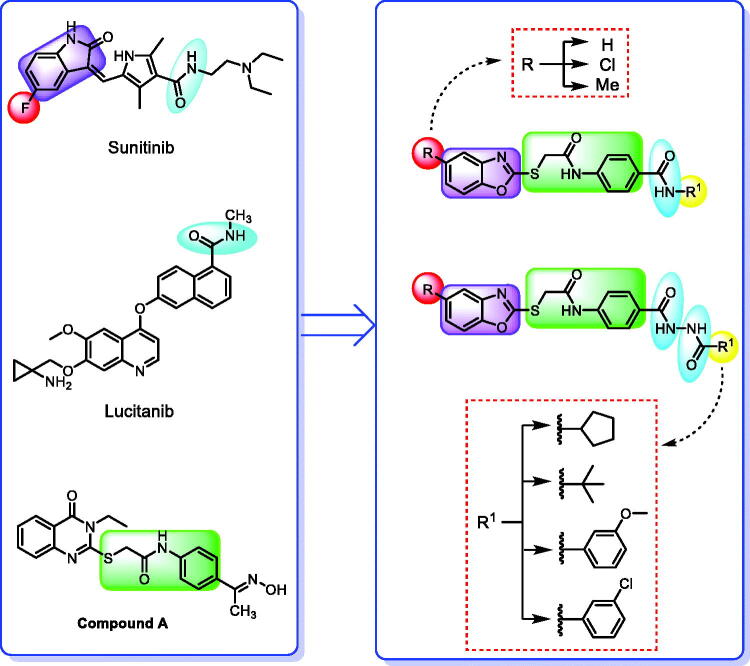
Summary of the suggested rationale.

Forcing by the fact that molecular hybridisation is one of the most important drug discovery approaches, our team co-workers started the present work. Sunitinib, a multi-targeted receptor tyrosine kinase (RTK) inhibitor^19^, lucitanib, a dual VEGFRs and FGFRs inhibitor^20^, and compound **A**, a potent VEGFR-2 inhibitor were our guides for building a new anti-angiogenic hybrid^21^. Thus, the indolinylidene moiety of sunitinib was altered to be benzoxazole in the new hybrid to investigate its ability to modify the biological effects. In addition, we did another modification to the sunitinib structure *via* replacing the fluorine atom by either hydrogen, methyl, or chlorine atoms that allowed us to measure the biological effects of these atoms compared to the fluorine atom. In contrast, the carboxamide moiety of both sunitinib and lucitanib was kept or expanded to continue acting as a hydrogen bonding part. On the other side, the hydrophobic tail in the new hybrid was suggested to be either aliphatic (*tert*-butyl), alicyclic (cyclopentyl), or aromatic (methoxy or chloro phenyl) to get a diverse number of congeners with a higher chance to study the structure-activity relationship of the newly designed hybrid. However, an *in silico* study was also carried out through the docking tools to confirm the proposed design (Figure 2).

## 2. Results and discussion

### 2.1. Chemistry

**Scheme 1.**
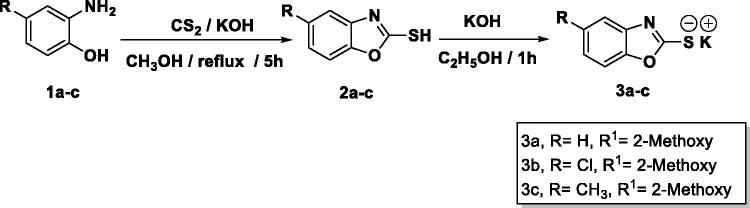
Synthesis of the starting materials **3a-c**.

**Scheme 2.**
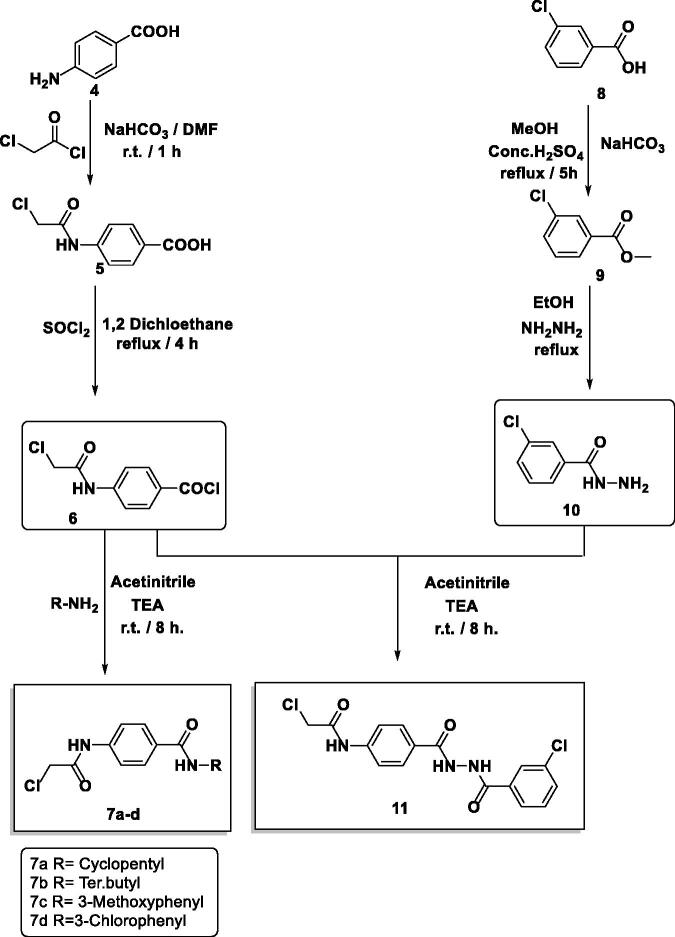
Synthesis of the intermediates **7a-d** and **11**.

**Scheme 3.**
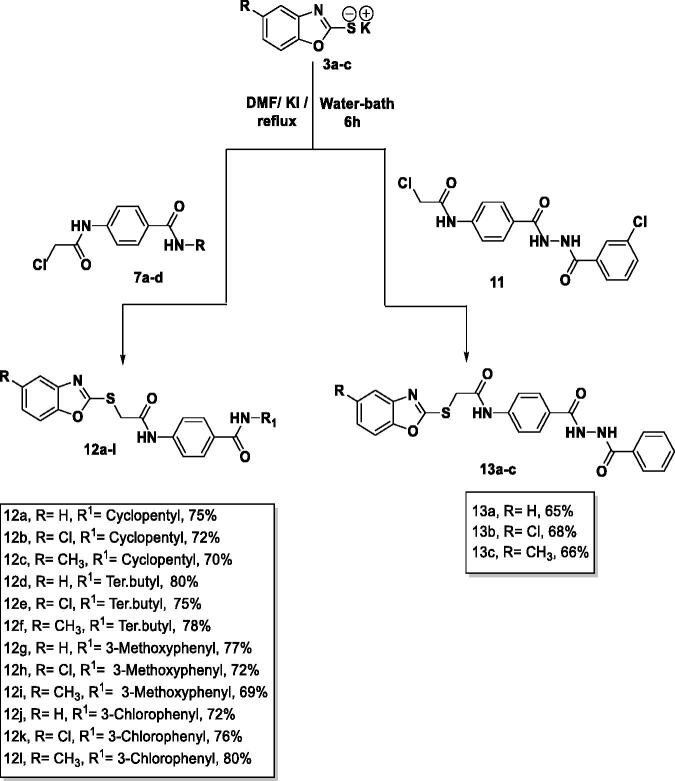
Synthesis of the final compounds **12a-l** and **13a-c.**

The final benzoxazoles **12a**-**l** and **13a**-**c** were synthesised as presented in Schemes 1–3. The starting materials and key intermediates **2a-c**, **3a**-**c**, **5**, **6**, **7a**-**d**, **9**, **10,** and **11** were primarily prepared according to the reported methods^22–25^ as delineated in Schemes 1 and 2.

The final target candidates **12a**-**l** and **13a**-**c** were furnished in dry DMF *via* heating the potassium salts 3**a-c** with the previously synthesised intermediates **7a**-**d** and **11**, respectively (Scheme 3). Infra-red (IR) spectra of compounds **12a-l** indicated the presence of characteristic NH and C = O groups stretching bands at a range of 3181–3412 and 1644–1688 cm^−1^, respectively. Moreover, their ^1^H NMR spectra showed the presence of the two NH amide group signals at a range of *δ* 7.73–10.83 ppm. The formation of compounds **13a**-**c** was confirmed by ^1^H NMR spectra which showed the appearance of three singlet signals at a range of *δ* 10.53–10.79 ppm corresponding to the NH protons.

### 2.2. Biological evaluation

#### 2.2.1. *In-vitro antiproliferative activities* against MCF-7 and HepG2 cell lines

The *in vitro* antiproliferative effects of the newly synthesised benzoxazole derivatives **12a-l** and **13a-c** were determined against hepatocellular cancer (HepG2) and breast cancer (MCF-7) cell lines employing the standard MTT assay protocol wherein sorafenib was applied as a reference. The cytotoxicity results were obtained as median growth inhibitory concentration (IC_50_). As presented in Table 1, major members of the synthesised compounds displayed promising anticancer activity.

**Table 1.** *In vitro* anti-proliferative effects of the obtained compounds against HepG2 and MCF-7 cell lines. 
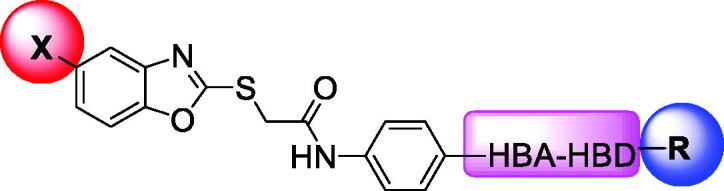

Comp. No.	X	HBA-HBD	R	*In vitro* IC_50_ (µM) ^a^	
				HepG2	MCF-7
**12a**	H	-NH-CO-	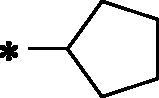	38.83 ± 3.2	33.27 ± 2.9
**12b**	Cl	-NH-CO-	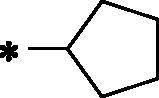	64.16 ± 6.1	77.03 ± 7.3
**12c**	CH_3_	-NH-CO-	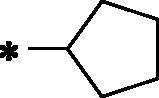	74.30 ± 6.8	36.72 ± 3.3
**12d**	H	-NH-CO-	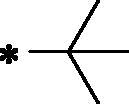	23.61 ± 2.1	44.09 ± 3.8
**12e**	Cl	-NH-CO-	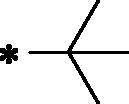	71.59 ± 6.7	62.29 ± 5.8
**12f**	CH_3_	-NH-CO-	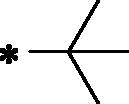	36.96 ± 3.4	22.54 ± 1.8
**12g**	H	-NH-CO-	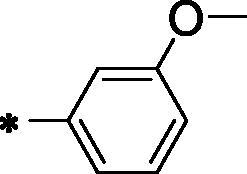	36.67 ± 2.9	53.01 ± 5.1
**12h**	Cl	-NH-CO-	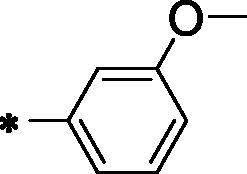	102.10 ± 8.5	85.62 ± 8.2
**12i**	CH_3_	-NH-CO-	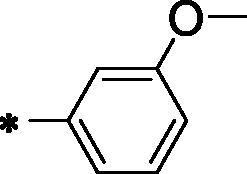	27.30 ± 2.2	27.99 ± 2.1
**12j**	H	-NH-CO-	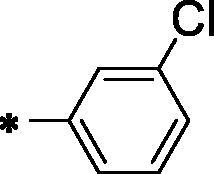	50.92 ± 4.6	33.61 ± 2.8
**12k**	Cl	-NH-CO-	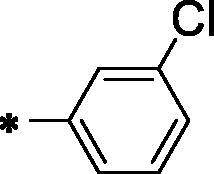	28.36 ± 2.5	86.62 ± 7.8
**12l**	CH_3_	-NH-CO-	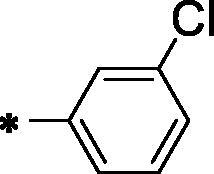	**10.50 **±** **0.8	**15.21 **±** **1.1
**13a**	H	-CO -NH-NH-CO-	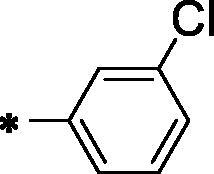	25.47 ± 2.1	32.47 ± 2.9
**13b**	Cl	-CO -NH-NH-CO-	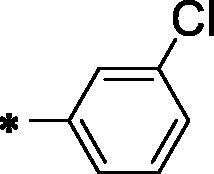	42.06 ± 3.8	26.31 ± 2.2
**13c**	CH_3_	-CO -NH-NH-CO-	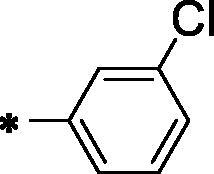	24.25 ± 2.1	53.13 ± 3.7
**Sorafenib**	**-**	**-**	**-**	**5.57 **±** **0.4	**6.46 **±** **0.3

**^a^**Data are presented as mean of the IC_50_ values from three different experiments.

Observing the results of anti-proliferative activity, valuable data concerning the structure-activity relationships was determined. In general, the 5-methylbenzo[*d*]oxazole containing derivatives (compounds **12c, 12f, 12i, 12 l,** and **13c**) (IC_50_ values ranging from 10.50 to 74.30 μM) were more active than the unsubstituted benzo[*d*]oxazole derivatives (compounds **12a, 12d, 12 g, 12j,** and **13a**) (IC_50_ values ranging from 25.47 to 53.01 μM). In the meantime, the 5-chlorobenzo[*d*]oxazole derivatives (compounds **12b, 12e, 12h, 12k,** and **13b**) (IC_50_ values ranging from 26.31 to 102.10 μM) exhibited less potent activities.

A closer look to the results indicated that compound **12l** achieved the most potent anticancer activity against HepG2 and MCF-7 cell lines with IC_50_ values of 10.50 μM and 15.21 μM, respectively, compared to sorafenib with IC_50_ value of 5.57 μM and 6.46 μM against HepG2 and MCF-7, respectively. This indicated that hybridisation of 5-methylbenzo[*d*]oxazole with terminal 3-chlorophenyl moiety potentiates the anticancer activity against HepG2 and MCF-7 cell lines. Moreover, compounds **12d** (IC_50_ = 23.61 and 44.09 µM), **12f** (IC_50_ = 36.96 and 22.54 µM), **12i** and (IC_50_ = 27.30 and 27.99 µM) exhibited promising activities against HepG2 and MCF-7 cell lines, respectively.

Initially, the effect of a hydrogen-bonding moiety on cytotoxic activities has been explored. Regarding the unsubstituted benzo[*d*]oxazole derivatives, it was noticed that the diamide derivative **13a** (IC_50_ = 25.47 and 32.47 µM against HepG2 and MCF-7, respectively) displayed better effects than the corresponding amide derivative **12j** (IC_50_ = 50.92 and 33.61 µM against MCF-7 and HepG2, respectively). Conversely, in 5-methylbenzo[*d*]oxazole derivatives, the decreased IC_50_ value of the amide derivative **12l** (IC_50_ = 10.50 μM and 15.21 μM against HepG2 and MCF-7, respectively) in comparison to the corresponding diamide member of the same scaffold **13c** (IC_50_ = 24.25 and 53.13 μM) indicated that the amide derivatives more preferred biologically than the corresponding diamide derivatives.

We then investigated the impact of the terminal hydrophobic tail on the *in-vitro* antiproliferative activities. Concerning the unsubstituted benzo[*d*]oxazole derivatives, compound **12d**, containing terminal *tert*-butyl moiety displayed the highest inhibitory activity against the HepG2 cell line with an IC_50_ value of 23.61 μM while **13a,** containing terminal 3-chlorophenyl moiety exhibited the lowest IC_50_ value (32.47 µM) against MCF-7 cell line. On the other hand, among 5-chlorobenzo[*d*]oxazole-based derivatives, the amide member bearing terminal 3-chlorophenyl arm **12k** displayed the most potent *in-vitro* antiproliferative activities against the HepG2 cell line with an IC_50_ value of 28.36 μM. In the meantime, the diamide member **13 b,** bearing the same terminal arm presented the most promising activity against MCF-7 cell line with IC_50_ value of 26.31 μM.

#### 2.2.2. Vegfr-2 inhibitory assay

VEGFR-2 inhibitory effect of the most cytotoxic candidates **12d, 12f, 12i, 12 l**, and **13a** was investigated and summarised in Table 2. Sorafenib was used as a reference.

**Table 2.** IC_50_ values of the tested compounds on the inhibitory activities against VEGFR-2 Kinases Assay.
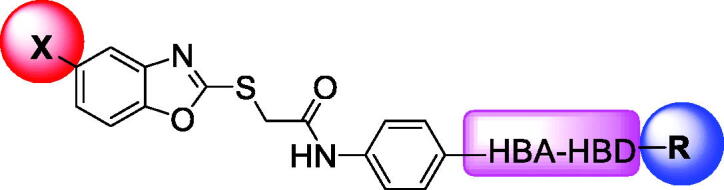

Comp. No.	X	HBA-HBD	R	VEGFR-2, IC_50_ (nM)
**12d**	H	-NH-CO-	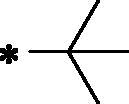	**194.60**
**12f**	CH_3_	-NH-CO-	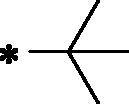	**264.90**
**12i**	CH_3_	-NH-CO-	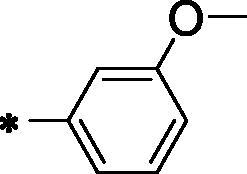	**155.00**
**12l**	CH_3_	-NH-CO-	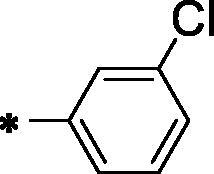	**97.38**
**13a**	H	-CO -NH-NH-CO-	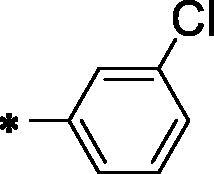	**267.80**
**Sorafenib**	**-**	**-**	**-**	**48.16**

Matching with the cytotoxicity results, compound **12l**, the most cytotoxic member, displayed the strongest VEGFR-2 inhibitory effect (IC_50_ = 97.38 nM) comparing sorafenib (IC_50_ = 48.16 nM). Additionally, compounds **12d** and **12i** showed moderate VEGFR-2 inhibitory effects with the concentrations of 194.6 and 155 nM, respectively. Unlikely, compounds **12f** and **13a** showed weak VEGFR-2 effects with the concentration of 264.90 and 267.80 nM, respectively.

#### 2.2.3. Correlation study between cytotoxicity and VEGFR-2 inhibition

**Figure 3.**
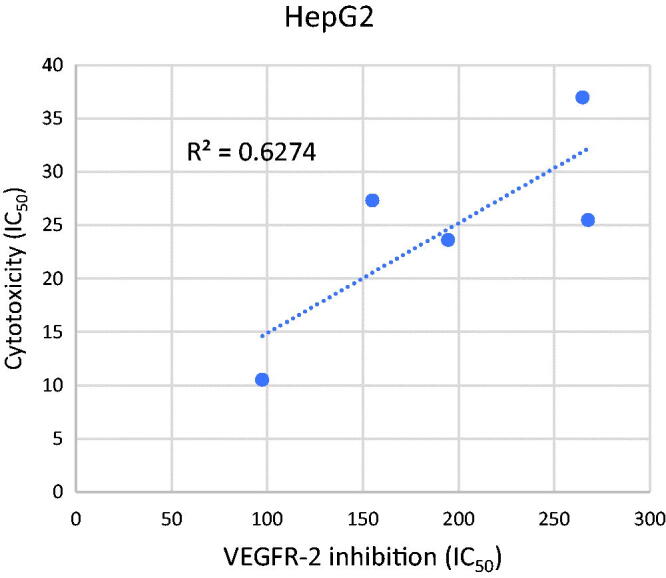
Correlation graph study.

The VEGFR-2 inhibitory activities of the tested compounds were plotted against their corresponding cytotoxicity in a simple linear regression for the HepG2 cell line in order to confirm the relationship between VEGFR-2 inhibition and cytotoxicity. The calculated R^2^ square value 0.6274) shows a significant correlation between the tested compounds' induction of cytotoxicity and inhibition of VEGFR-2. As a result, one possible mechanism of the established compounds' cytotoxicity in the established cell line is their inhibition of VEGFR-2 activity (Figure 3).

#### 2.2.4. Evaluation of *in vitro* cytotoxicity against normal cell line

The most potent members **12d**, **12i**, and **12l** were assessed for their *in vitro* cytotoxicity against normal cell lines using WI-38 (a human lung cell line) and sorafenib as a reference The IC_50_ values for compounds **12d**, **12i**, and **12l** were 99.41, 76.78, and 37.97 M, respectively (Table 3). Such values were very high in comparison to the corresponding values on cancer cell lines, which reflect high safety profile of the tested candidates towards normal cell lines.

**Table 3.** IC_50_ results of **12d**, **12i**, and **12 l** against WI-38 cell line.

Compound	WI-38, IC_50_ (µM)
**12d**	99.41
**12i**	76.78
**12l**	37.79
**Sorafenib**	22.10

#### 2.2.5. Cell cycle analysis

**Figure 4.**
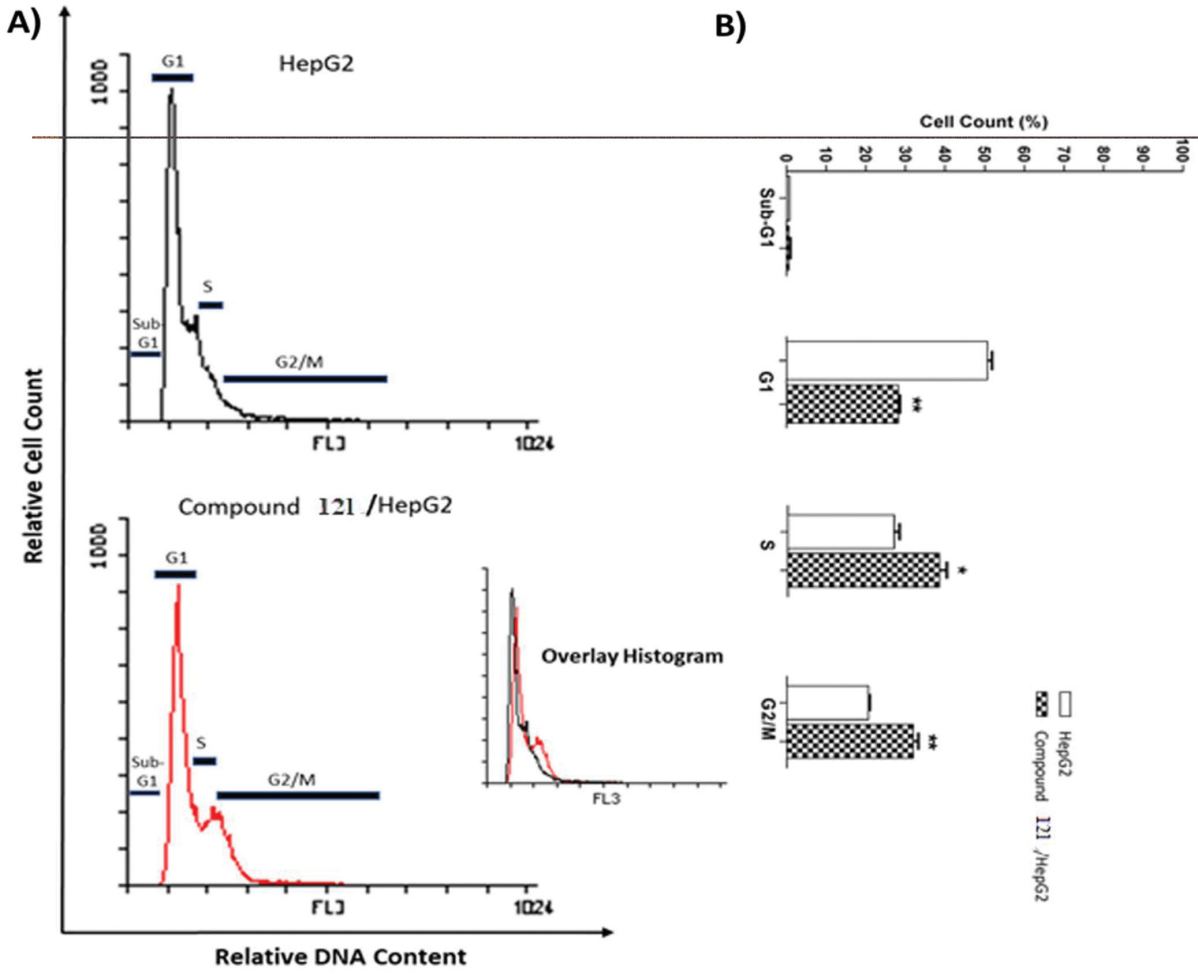
Flow cytometry analysis of HepG2 cell cycle after the treatment of compound **12 l**.

Compound **12I,** achieved notable cytotoxic and VEGFR-2 inhibitory potencies was further studied mechanistically for cell cycle progression and induction of apoptosis in HepG2 cells. Cell cycle process was analysed after exposure of HepG2 cells to **12I** with a concentration of 10.50 µM for 24 h. Flow cytometry data revealed that the percentage of cells arrested at Pre-G1 phase decreased from 0.93% (in control cells) to 0.79% (in **12I**) treated cells. Additionally, a marked decrease in cell population was observed at the G1 phase (28.34%) comparing to control cells (51.07%). For the S phase compound **12l** induced a significant increase in the cell population (38.68%) comparing to control cells (27.22%). Finally, compound **12I** exhibited significant increase in the cell population (32.10%) at the G2/M phase, comparing to the control cells (20.78%). Such outputs verify that compound **12I** arrested the HepG2 cancer cell’s growth mainly at the Pre-G1 and G1 phases (Table 4 and Figure 4).

**Table 4.** Supressing potentialities of **12I** on the cell cycle of HepG2 cells after 24 h treatment.

Sample	Cell cycle distribution (%)^a^			
	%Sub-G1	%G1	%S	% G2/M
**HepG2**	0.93 ± 0.02	51.07 ± 1.03	27.22 ± 1.24	20.78 ± 0.23
**Compound 12 l /HepG2**	0.79 ± 0.25	28.43 ± 0.37**	38.68 ± 1.81*	32.10 ± 181**

**^a^**Values are given as mean ± SEM of two independent experiments and *p < 0.05; **p < 0.01.

#### 2.2.6. Apoptosis analysis

**Figure 5.**
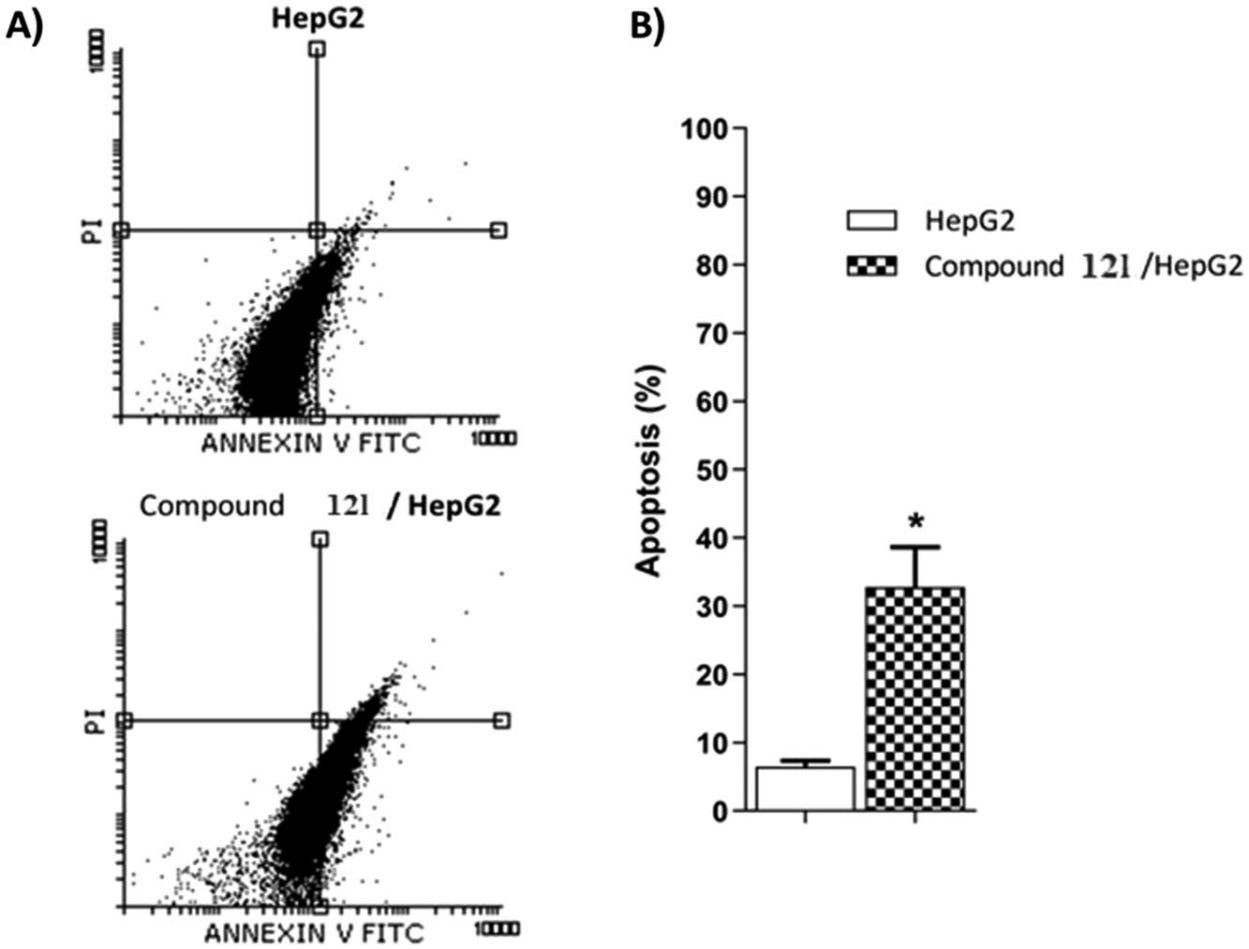
Flow cytometry analysis of compound **12 l** apoptotic induction against HepG2 cells.

The most potent anticancer agent **12l** was selected for the assessment of apoptosis in HepG2 cells using Annexin V/propidium iodide (PI) double staining assay method. In this method, HepG2 cells were incubated with compound **12l** at the IC_50_ concentration (10.50 µM) for 24 h. The results revealed that compounds **12l** could induce apoptosis more than the untreated control cells by a ratio of 35.13%. In details, 32.45 and 2.86% for early and late apoptotic phases, respectively compared to control, (6.56%,5.34%,1.22%, respectively) (Figure 5 and Table 5).

**Table 5.** Apoptotic potentialities compound **12 l** against HepG2 cells after 24 h treatment.

Sample	**Viable a** (Left Bottom)	Apoptosis a		**Necrosis a** (Left Top)
		**Early** (Right Bottom)	**Late** (Right Top)	
**HepG2**	92.96 ± 0.55	5.34 ± 0.01	1.22 ± 0.77	0.48 ± 0.27
**12l / HepG2**	64.55 ± 3.43	32.45 ± 3.13*	2.86 ± 0.21	0.14 ± 0.06

**^a^**Values are given as mean ± SEM of two independent experiments. *p < 0.05.

#### 2.2.7. Evaluation of BAX and bcl-2 expressions

**Figure 6.**
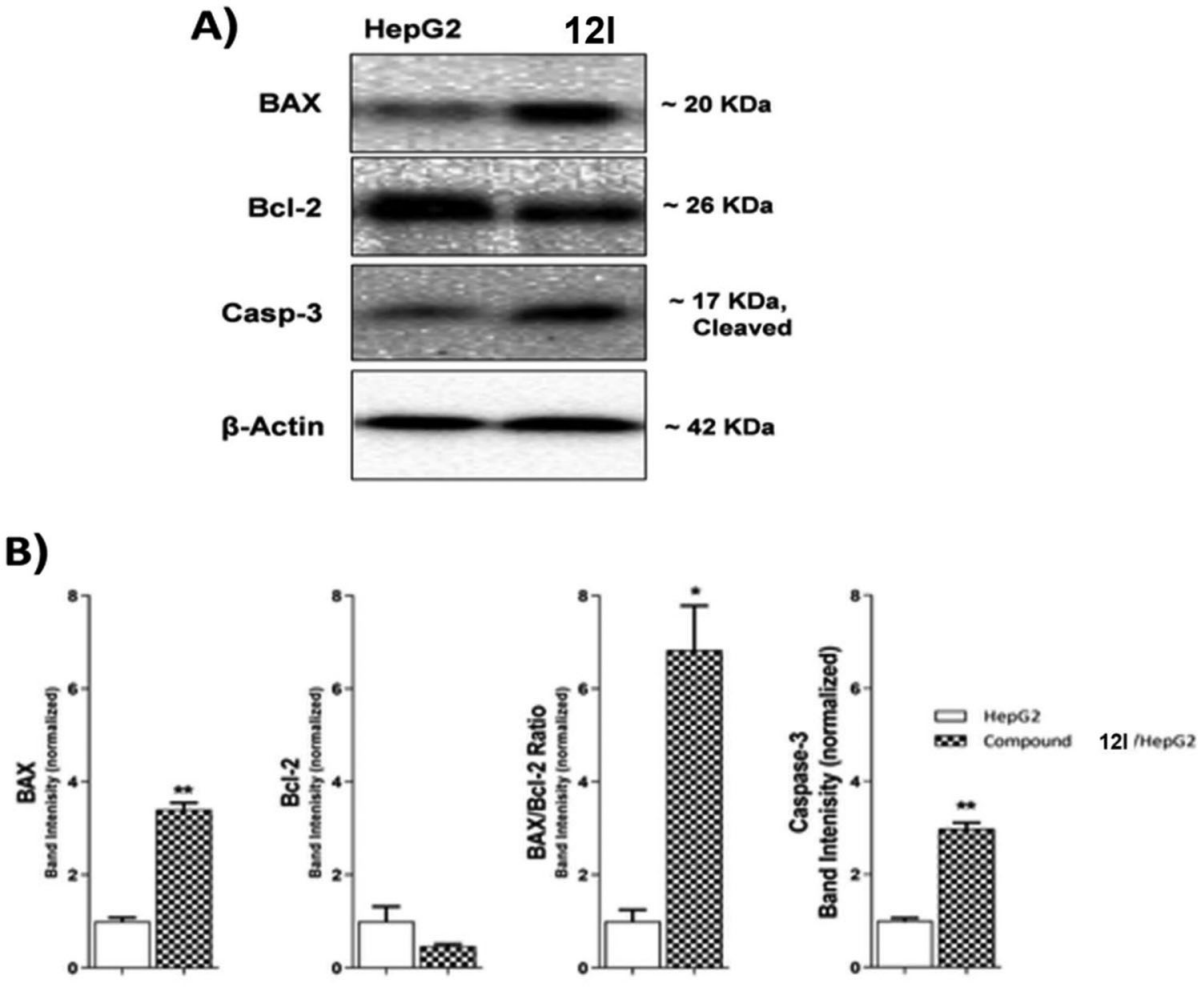
The immunoblotting of effect of compound **12 l** against BAX, Bcl-2, and Caspase-3.

Compound **12l**was subjected to further cellular mechanistic study. The cellular levels of BAX and Bcl-2 were measured using the western blot technique after compound **12l** was applied to HepG2 cells for 24 h. The results indicated that compound **12l** increased the concentration of the pro-apoptotic factor BAX by 3.40-fold while decreasing the concentration of the anti-apoptotic protein Bcl-2 by 2.12-fold. Furthermore, a significant increase in the BAX/Bcl-2 ratio by 6.83-fold was observed. The obtained findings indicated that compound **12l** was effective in the apoptosis cascade and may encourage the apoptotic pathway (Table 6 and Figure 6).

**Table 6.** Effect of compound **12 l** on the levels of BAX, Bcl-2, and Caspase-3 proteins expression in HepG2 cells treated for 24 h.

Sample	Protein expression (normalized to β-actin) ^a^			
	BAX	**Bcl-2**	BAX/Bcl-2 ratio	**Caspase-3**
HepG2	1.00 ± 0.08	1.00 ± 0.32	1.00 ± 0.25	1.00 ± 0.06
**12l**	3.40 ± 0.15**	0.47 ± 0.05	6.83 ± 0.96*	2.98 ± 0.13**

**^a^**Values are given as mean ± SEM of two independent experiments. *p < 0.05, **p < 0.01.

#### 2.2.8. Caspase 3 assay

Caspase-3 has a key role in apoptosis initiation and execution^26^^,^^27^. The western blot technique was used to investigate the effect of compound **12 l,** the most promising member, on the caspase-3 level. HepG2 cells were treated with **12l** (10.50 µM) for 24 h. Comparing control HepG2 cells, compound **12l** caused a significant increase in the cellular levels of caspase-3 (2.98-fold) as presented in Table 6 and Figure 6.

### 2.3. *In silico* studies

#### 2.31.1. Docking study

**Figure 7.**
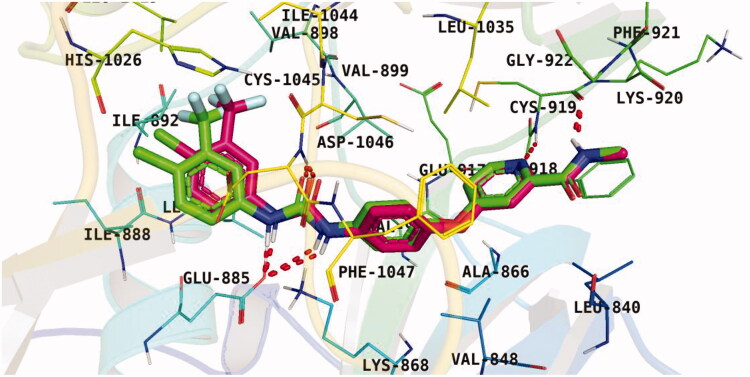
Results of the re-docking step into the VEGFR-2 catalytic site; native ligand (green) and the obtained pose (red).

To understand the pattern by which the synthesised compounds bound to the active site^28^^,^^29^, all compounds were subjected to a docking study into the VEGFR-2 ATP binding site (PDB: 4ASD, resolution: 2.03 Å). The native co-crystallized inhibitor, sorafenib, was adopted as a reference in the present work. Following the preparation of the downloaded protein, a validation step was carried out in which the native inhibitor, sorafenib, was re-docked against the catalytic VEGFR-2 site. Results of the previous step successfully reproduced an identical binding pattern to that of the co-crystallized ligand with an RMSD value of 0.71 Å Figure 7. Thus, the later findings supported the validity of the suggested docking protocol.

**Figure 8.**
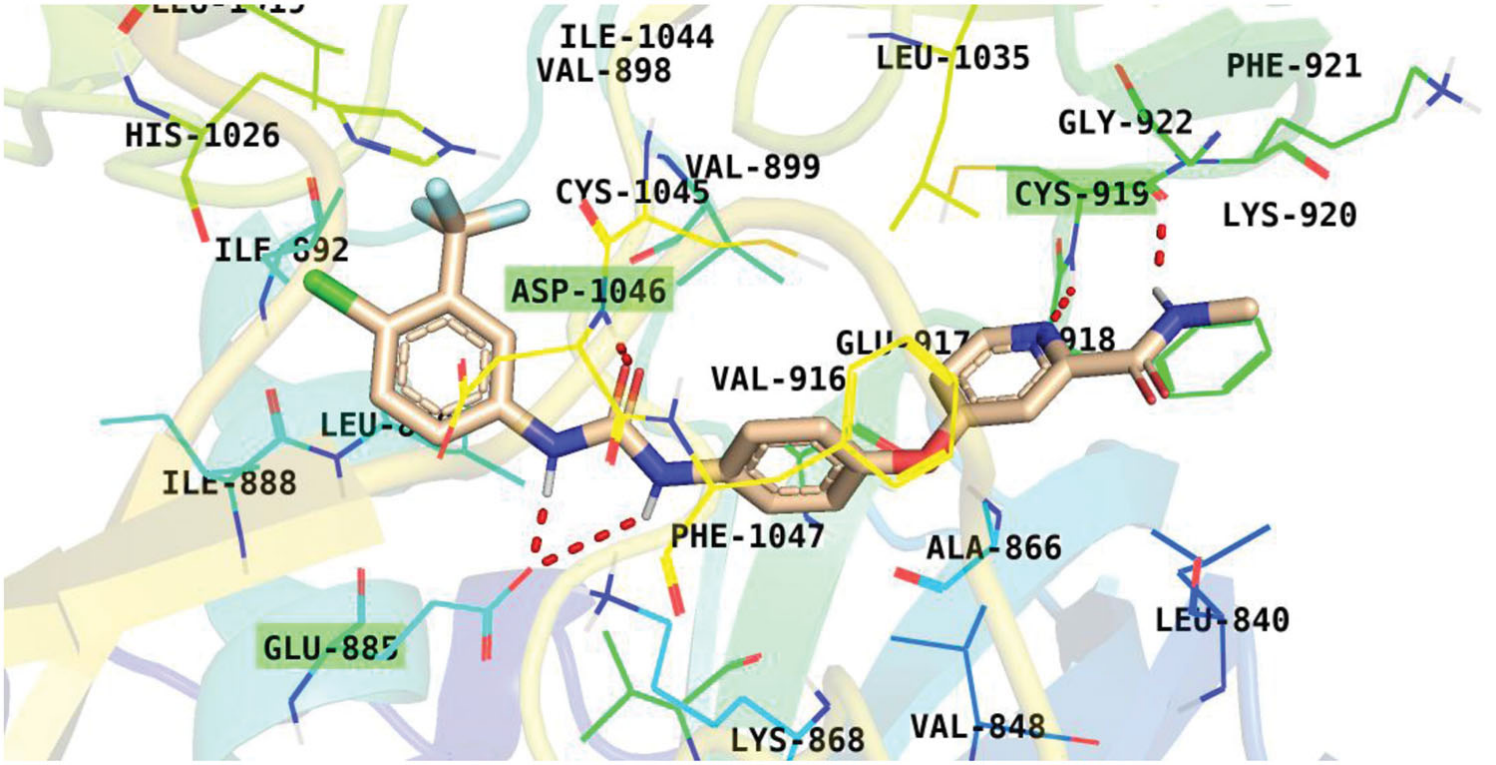
Sorafenib binding interactions with VEGFR-2 catalytic site.

Observation of the kinds of interaction between sorafenib and the VEGFR-2 catalytic site revealed that it could form two interaction types (Figure 8). The 1^st^ type is an H-bonding interaction, as sorafenib formed two H-bonds with a critical amino acid (Cys919) in the hinge region in addition to three H-bonds with the DFG motif amino acids (Asp1046 and Glu885). The 2^nd^ interaction type included different π interactions between sorafenib and the hydrophobic amino acids among the active pocket.

**Figure 9.**
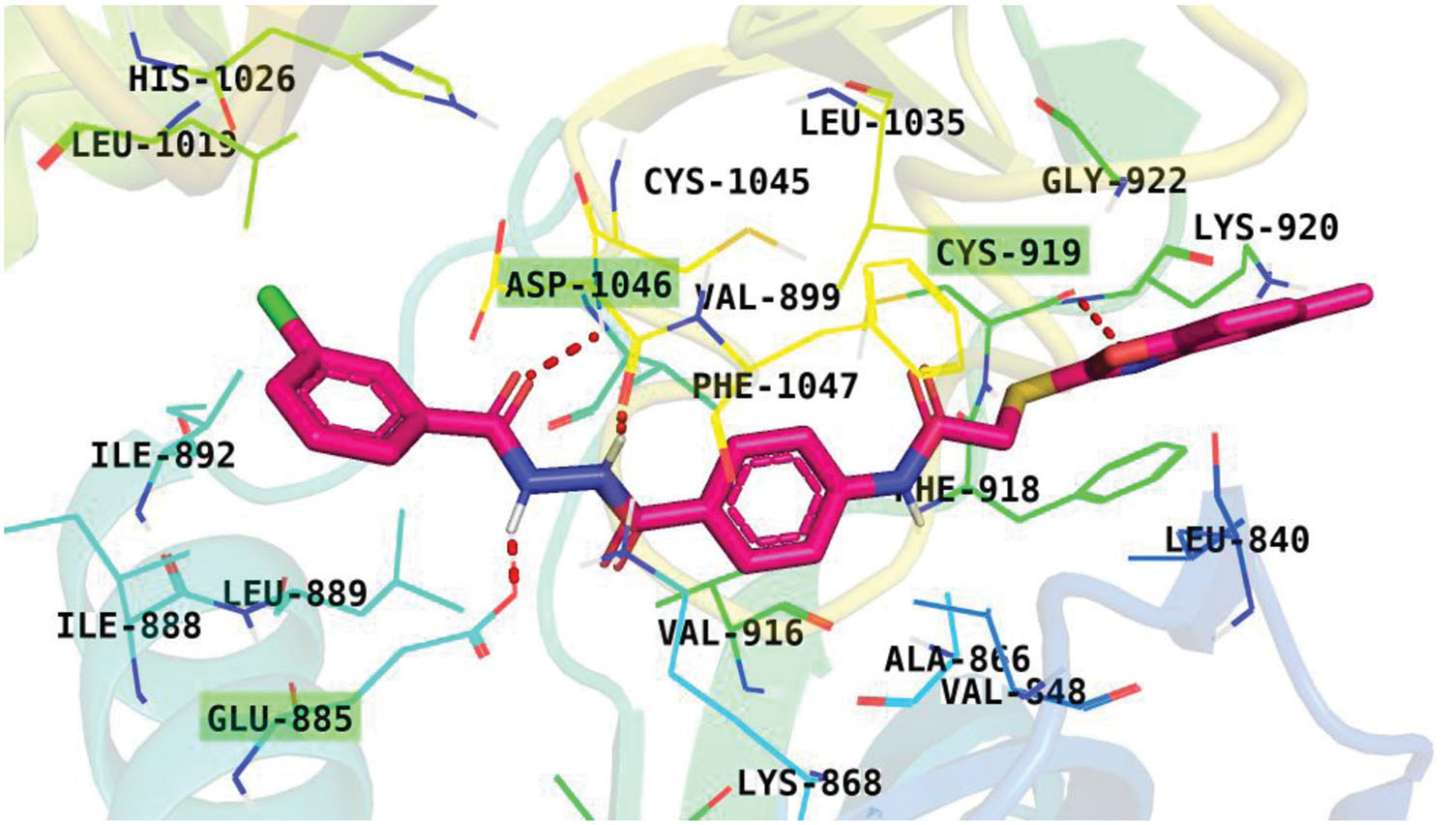
Binding pose of **12 l** with the active site of VEGFR-2.

**Figure 10.**
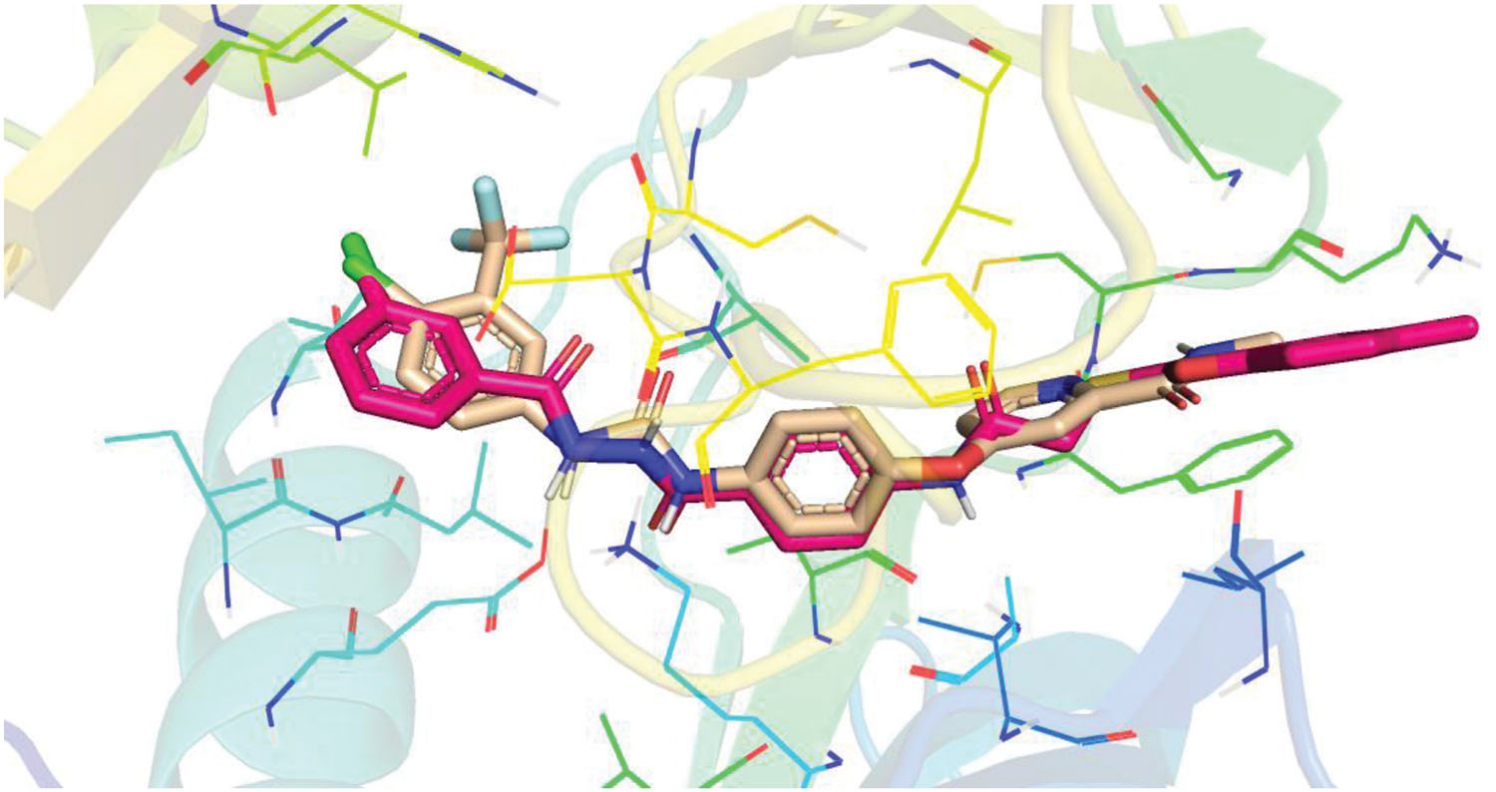
Superimposition of **12 l** (red) and sorafenib (wheat) inside the VEGFR-2 catalytic site.

Docking conformations of the synthesised derivatives revealed that they were stacked onto the VEGFR-2 catalytic site in a way similar to that of the original ligand. However, the predicted docking pose of compound **12l** showed that its benzoxazole fragment was linked to the hinge region Cys919 amino acid *via* a strong H-bond. Additionally, compound **12l** interacted by an H-bond with Glu885 and two H-bonds with Asp1046 in the DFG motif (Figure 9). The later binding pattern gave a reasonable explanation for **12l** of being the most active biologically among the tested compounds. A superimposition poses of **12l** and the native ligand, sorafenib, provided additional evidence to the obtained results. As presented in Figure 10, compound **12l** and sorafenib generally overlapped well and had the same 3-D orientation. Niceties revealed that the pharmacophoric moieties of sorafenib represented by *N*-methylpicolinamide, phenoxy, urea, and 4-chloro-3-(trifluoromethyl)phenyl) moieties had the same orientation with the *5-methylbenzo[d]oxazol*, *N*-phenylacetamide, amide, and 3-chlorophenyl moieties, respectively of compound **12l**.

#### 2.3.2. Pharmacokinetic profiling study

In the current study, an *in silico* computational study of the tested candidates was conducted following the directions of Veber’s and Lipinski’s rule of five^30^^,^^31^.

The obtained findings presented in Table 7 showed that all tested compounds showed no contravention of Lipinski’s and Veber’s Rules and hence display a drug-like molecular nature. In detail, the LogP, molecular weight, number of H-bond donors, and number of H-bond acceptors of these fifteen compounds are within the accepted values of less than 5, 500, 5, and 10, respectively. Moreover, the number of rateable bonds and TPSA of such compounds are within the acceptable values of less than 10 and 140 Å^2^, respectively.

**Table 7.** Physicochemical properties of the tested compounds passed Lipinski and Veber Rules

Comp.	Lipinski Rules				**Veber Rules**	
	Num HD	Num HA	M Wt	AlogP	Num Rotatable Bonds	TPSA
**12a**	2	4	395.475	3.685	6	109.53
**12b**	2	4	429.92	4.35	6	109.53
**12c**	2	4	409.501	4.171	6	109.53
**12d**	2	4	383.464	3.214	6	109.53
**12e**	2	4	417.909	3.879	6	109.53
**12f**	2	4	397.491	3.701	6	109.53
**12g**	2	5	433.48	3.843	7	118.76
**12h**	2	5	467.925	4.508	7	118.76
**12i**	2	5	447.506	4.33	7	118.76
**12j**	2	4	437.899	4.524	6	109.53
**12k**	2	4	472.344	5.189	6	109.53
**12l**	2	4	451.925	5.01	6	109.53
**13a**	3	5	446.478	3.117	7	138.63
**13b**	3	5	480.923	3.781	7	138.63
**13c**	3	5	460.505	3.603	7	138.63

#### 2.3.3. Swissadme study

To compute the physicochemical properties and the drug likeness properties of the most potent compounds **12d, 12i,** and **12 l,** SwissADME online web tool was applied. The obtained results predicted that the physicochemical properties of the three candidates were in acceptable ranges, hence they may have good oral bioavailability. Also, they are expected to hve undesirable effects on CNS as they cannot pass BBB (Table 8). Furthermore, SwissADME revealed that compounds **12d, 12i,** and **12l** fulfilled Lipinskìs, Veber’s, and Ghose’s rules predicting that these compounds have promising drug-likeness profiles (Table 7). Moreover, the radar charts which involved the calculation of six parameters including lipophilicity, polarity, flexibility, size, saturation, and solubility showed that compounds **15 b** and **17 b** (represented by red lines and integrated into the pink area) are almost predicting acceptable oral bioavailability (Table 9).

**Table 8.** ADME profile of compounds **12d, 12i,** and **12 l**

Parameter	12d	12i	12l
**Physicochemical properties**			
***Molecular weight***	383.46	447.51	451.93
***Num. heavy atoms***	27	32	31
***Num.* H-bond acceptors**	4	5	4
***Num.* H-bond donors**	2	2	2
**Molar Refractivity**	107.02	125.24	123.75
**TPSA**	109.53 Å²	118.76 Å²	109.53 Å²
**Consensus Log *P*_o/w_**	3.34	3.98	4.48
**Log *S* (ESOL)**	Moderately soluble	Moderately soluble	Moderately soluble
***Drug likeness***			
**Lipinski violations**	Yes; 0 violation	Yes; 0 violation	Yes; 0 violation
**Ghose violations**	Yes	Yes	Yes
**Veber violations**	Yes	Yes	Yes
**Bioavailability Score**	0.55	0.55	0.55
***Pharmacokinetics***			
**GI absorption**	High	Low	Low
**BBB permeant**	No	No	No
**CYP1A2 inhibitor**	Yes	Yes	Yes
**CYP2C19 inhibitor**	Yes	Yes	Yes
**CYP2C9 inhibitor**	Yes	Yes	Yes
**CYP2D6 inhibitor**	Yes	Yes	Yes
**CYP3A4 inhibitor**	Yes	Yes	Yes

**Table 9.** Radar charts for prediction of oral bioavailability profile of compounds **12d, 12i,** and **12 l**

Compounds	12d	12i	12l
***Radar images***	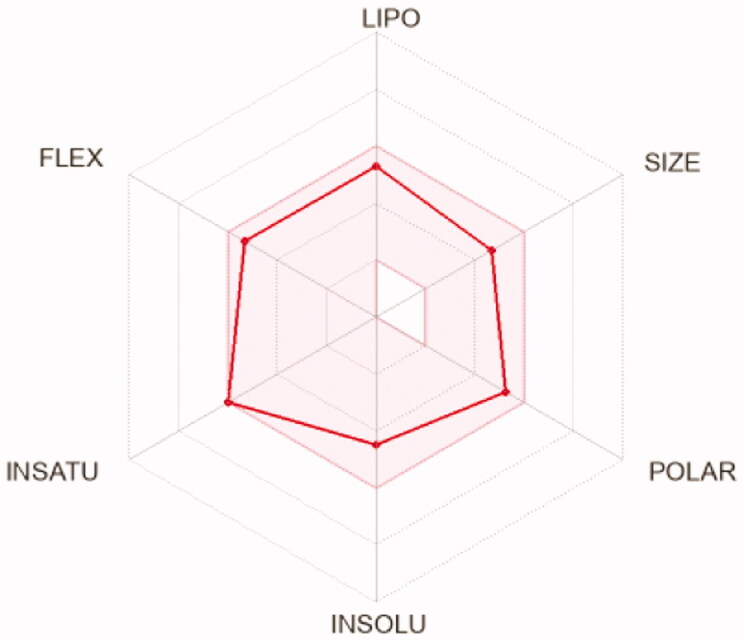	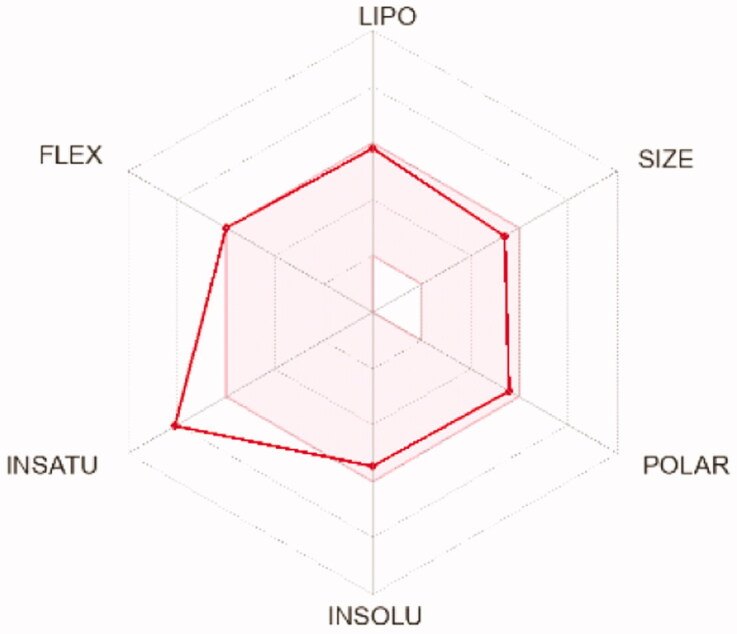	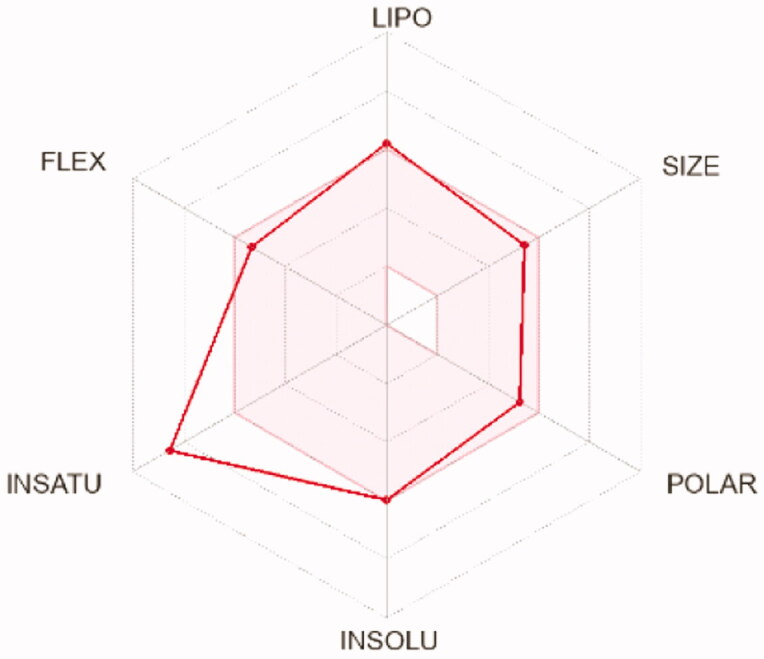

## 3. Conclusion

In the present study, fifteen benzoxazole derivatives were designed, synthesised as potential anticancer and VEGFR-2 inhibitors. The anticancer potentialities of the obtained derivatives were estimated against HepG2, and MCF-7 cell lines cell lines. Five compounds **12d** (IC_50_ = 23.61 & 44.09 µM), **12f** (IC_50_ = 36.96 & 22.54 µM), **12i** (IC_50_ = 27.30 & 27.99 µM), compounds **12d** (IC_50_ = 23.61 & 44.09 µM), **12f** (IC_50_ = 36.96 & 22.54 µM), **12i** (IC_50_ = 27.30 & 27.99 µM), and **13a** (IC_50_ = 11.4 & 14.2 µM) displayed noticeable anticancer activities against HepG2 and MCF-7, respectively. Moreover, VEGFR-2 kinase inhibition assay results revealed that compound **12l** showed the most potent inhibitory activity against VEGFR-2, comparing the reference drug, sorafenib. Owing to its notable high antiproliferative and VEGFR-2 inhibitory activities, derivative **12l** was selected for further evaluation to understand its mechanistic studies. Cell cycle analysis indicated that **12l** could arrest the malignant HepG2cells at the Pre-G1 and G1 phases and induced apoptosis by 35.13%, compared to 6.56% in the control cells. Additionally, compound **12l** exhibited significant potential to increase caspase 3 (BAX and BAX/Bcl-2 ratio with (2.98, 3.40- and 6.83 folds, respectively). Similarly, it decreased Bcl-2 (2.12-fold) comparing the untreated cells. Molecular docking studies were accomplished for all the target derivatives. Docking findings supported biological activity results where the most potent VEGFR-2 inhibitor was able to incorporate the tyrosine kinase domain of VEGFR-2 in a fashion comparable to that of the well-known VEGFR-2 inhibitor, sorafenib.

## 4. Experimental

### 4.1. Chemistry

In Supplementary data, all apparatus used in the analysis of produced chemicals were elucidated. Compounds **2a-c**, **3a-c**, **6**, **7a-c**, **9**, **10**, and **11** were synthesised using procedures that have previously been reported^32^. The ^1^H/^13^C NMR analyses were carried out at 400 and 100 MHz, respectively in DMSO-d_6_ as a solvent. The chemical shifts were presented as ppm. The infra-red investigations were carried out using KBr disc and the results were presented as cm^−1^. The colours and meting points of the final compounds **12a-l** and **13a-c** were presented in Table 10.

**Table 10.** Colours, yields, and meting points of the target compounds

Compounds	Color	Meting points (°C)
**12a**	White crystals	230–232
**12b**	White crystals	240–242
**12c**	White crystals	235–237
**12d**	White crystals	211–215
**12e**	White crystals	233–235
**12f**	White crystals	222–224
**12g**	White crystals	252–254
**12h**	White crystals	244–246
**12i**	White crystals	255–257
**12j**	White crystals	240–242
**12k**	White crystals	220–222
**12l**	White crystals	266–268
**13a**	White crystals	223–225
**13b**	White crystals	211–213
**13c**	White crystals	235–237

#### 4.1.1. General procedure for preparation of the target compounds 12a-l

In 10 ml DMF containing 0.001 mol KI, 0.001 mol of **3a-c** and 0.001 mol of the appropriate benzamide derivatives **7a-d** were mixed and heated under reflux for 6 h. The reaction content was then poured on crushed ice. The collected crystals were filtered and crystalised from methanol to afford **12a-l**.

##### 4.1.1.1. 4–(2-(Benzo[d]oxazol-2-ylthio)acetamido)-N-cyclopentylbenzamide 12a

IR: 3495, 3383 (NH), 3054 (CH aromatic), 2951 (CH aliphatic), 1661, 1623 (C = O); ^1^H NMR: 10.69 (s, 1H), 8.20 (d, *J* = 7.3 Hz, 1H), 7.85 (d, *J* = 8.4 Hz, 2H), 7.75–7.59 (m, 4H), 7.40–7.28 (m, *J* = 6.7, 5.4 Hz, 2H), 4.44 (s, 2H), 4.23 (h, *J* = 7.0 Hz, 1H), 1.89 (m, 2H), 1.79–1.64 (m, 2H), 1.59–1.50 (m, 4H); ^13 ^C NMR: 165.81, 165.79, 164.32, 151.82, 141.68, 141.52, 128.74, 125.15, 124.83, 118.71, 110.69, 51.38, 37.26, 32.62, 24.10; MS (*m/z*) for C_21_H_21_N_3_O_3_S (395.48): 395.50 (M^+,^ 100%).

##### 4.1.1.2. 4–(2-((5-Chlorobenzo[d]oxazol-2-yl)thio)acetamido)-N-cyclopentylbenzamide 12b

IR: 3414, 3272 (NH), 3064 (CH aromatic), 2938 (CH aliphatic), 1656 (C = O); ^1^H NMR: 10.68 (s, 1H), 8.20 (d, *J* = 7.3 Hz, 1H), 7.85 (d, *J* = 8.3 Hz, 2H), 7.76–7.63 (m, 4H), 7.37 (dd, *J* = 8.7, 2.1 Hz, 1H), 4.45 (s, 2H), 4.22 (h, *J* = 7.2 Hz, 1H), 1.89 (m, 2H), 1.69 (m, 2H), 1.53 (m, 4H); ^13 ^C NMR: 166.37, 165.93, 165.66, 150.58, 142.91, 141.44, 130.14, 129.48, 128.72, 124.80, 118.78, 118.46, 111.99, 51.40, 37.22, 32.57, 24.07.

##### 4.1.1.3. N-Cyclopentyl-4–(2-((5-methylbenzo[d]oxazol-2-yl)thio)acetamido)benzamide 12c

IR: 3273 (NH), 3041 (CH aromatic), 2945 (CH aliphatic), 1657, 1618 (C = O); ^1^H NMR: 10.68 (s, 1H), 8.20 (d, *J* = 7.3 Hz, 1H), 7.86 (d, *J* = 8.4 Hz, 2H), 7.67 (d, *J* = 8.4 Hz, 2H), 7.51 (d, *J* = 8.3 Hz, 1H), 7.42 (s, 1H), 7.16–7.09 (m, 1H), 4.42 (s, 2H), 4.23 (h, *J* = 7.0 Hz, 1H), 2.39 (s, 3H), 1.88 (m, 2H), 1.76–1.64 (m, 2H), 1.62–1.46 (m, 4H); ^13 ^C NMR: 165.81, 164.17, 150.07, 141.70, 134.54, 130.16, 129.46, 125.60, 118.66, 110.04, 51.38, 37.26, 32.62, 24.10, 21.38; MS (*m/z*) for C_22_H_23_N_3_O_3_S (409.50): 409.48 (M^+^, 100%).

##### 4.1.1.4. 4–(2-(Benzo[d]oxazol-2-ylthio)acetamido)-N-(tert-butyl)benzamide 12d

IR: 3377, 3272 (NH), 3038 (CH aromatic), 2971 (CH aliphatic), 1613 (C = O); ^1^H NMR: 10.68 (s, 1H), 7.81 (d, *J* = 8.3 Hz, 2H), 7.75 − 7.57 (m, 5H), 7.39–7.27 (m, 2H), 4.44 (s, 2H), 1.38 (s, 9H); MS (*m/z*) for C_20_H_21_N_3_O_3_S (383.47): 383.28 (M^+^, 100%).

##### 4.1.1.5. N-(Tert-butyl)-4–(2-((5-chlorobenzo[d]oxazol-2-yl)thio)acetamido)benzamide 12e

IR: 3412, 3277 (NH), 3072 (CH aromatic), 2951 (CH aliphatic), 1655, 1604 (C = O); ^1^H NMR: 10.70 (s, 1H), 7.81 (d, *J* = 8.5 Hz, 2H), 7.73 (d, *J* = 2.1 Hz, 1H), 7.71–7.62 (m, 4H), 7.36 (dd, *J* = 8.7, 2.2 Hz, 1H), 4.45 (s, 2H), 1.38 (s, 9H); ^13 ^C NMR: 166.42, 166.08, 165.57, 150.60, 142.96, 141.32, 131.22, 129.46, 128.77, 124.74, 118.61, 118.48, 111.95, 51.17, 37.41, 29.10; MS (*m/z*) for C_20_H_20_ClN_3_O_3_S (417.91): 417.36 (M^+^, 100%).

##### 4.1.1.6. N-(Tert-butyl)-4–(2-((5-methylbenzo[d]oxazol-2-yl)thio)acetamido)benzamide 12f

IR: 3383, 3286 (NH), 3072 (CH aromatic), 2965 (CH aliphatic), 1709, 1626 (C = O); ^1^H NMR: 10.66 (s, 1H), 7.88–7.73 (m, 2H), 7.72–7.58 (m, 3H), 7.53 (d, *J* = 8.3 Hz, 1H), 7.47–7.36 (m, 1H), 7.14 (dd, *J* = 8.4, 1.7 Hz, 1H), 4.41 (s, 2H), 2.40 (s, 3H), 1.38 (s, 9H); MS (*m/z*) for C_21_H_23_N_3_O_3_S (397.49): 397.43 (M^+^, 100%).

##### 4.1.1.7. 4–(2-(Benzo[d]oxazol-2-ylthio)acetamido)-N-(3-methoxyphenyl)benzamide 12 g

IR: 3262 (NH), 3033 (CH aromatic), 2927 (CH aliphatic), 1647 (C = O); ^1^H NMR: 10.83 (s, 1H), 10.17 (s, 1H), 8.00 (d, *J* = 8.3 Hz, 2H), 7.78 (d, *J* = 8.3 Hz, 2H), 7.66 (p, *J* = 5.8 Hz, 2H), 7.51 (t, *J* = 2.3 Hz, 1H), 7.41 (d, *J* = 8.1 Hz, 1H), 7.38–7.27 (m, 2H), 7.25 (d, *J* = 8.1 Hz, 1H), 6.68 (dd, *J* = 8.3, 2.5 Hz, 1H), 4.48 (s, 2H), 3.77 (s, 3H); ^13 ^C NMR: 165.99, 165.32, 164.17, 159.89, 150.09, 141.88, 140.93, 134.58, 129.82, 129.23, 125.65, 118.86, 118.67, 113.01, 110.10, 106.48, 55.47, 21.40; MS (*m/z*) for C_23_H_19_N_3_O_4_S (433.48): 433.34 (M^+^, 100%).

##### 4.1.1.8. 4–(2-((5-Chlorobenzo[d]oxazol-2-yl)thio)acetamido)-N-(3-methoxyphenyl)benzamide 12 h

IR: 3412, 3259 (NH), 3065 (CH aromatic), 2991, 2933 (CH aliphatic), 1656 (C = O); ^1^H NMR: 10.80 (s, 1H), 10.15 (s, 1H), 7.99 (d, *J* = 8.3 Hz, 2H), 7.86–7.70 (m, 3H), 7.67 (d, *J* = 8.7 Hz, 1H), 7.50 (s, 1H), 7.38 (dd, *J* = 18.8, 8.4 Hz, 2H), 7.25 (t, *J* = 8.2 Hz, 1H), 6.67 (d, *J* = 8.3 Hz, 1H), 4.48 (s, 2H), 3.76 (s, 3H); ^13 ^C NMR: 166.42, 166.08, 165.57, 150.60, 142.96, 141.32, 131.22, 129.46, 128.77, 124.74, 118.61, 118.48, 111.95, 51.17, 29.10; MS (*m/z*) for C_23_H_18_ClN_3_O_4_S (467.92): 467.17 (M^+^, 40%), 345.36 (100%).

##### 4.1.1.9. N-(3-Methoxyphenyl)-4–(2-((5-methylbenzo[d]oxazol-2-yl)thio)acetamido)benzamide 12i

IR: 3385, 3282 (NH), 3073 (CH aromatic), 2931 (CH aliphatic), 1688, 1648 (C = O); ^1^H NMR: 10.79 (s, 1H), 10.15 (s, 1H), 7.98 (d, *J* = 8.4 Hz, 2H), 7.76 (d, *J* = 8.4 Hz, 2H), 7.56–7.46 (m, 2H), 7.44 (d, *J* = 1.7 Hz, 1H), 7.39 (dd, *J* = 8.1, 1.9 Hz, 1H), 7.25 (t, *J* = 8.1 Hz, 1H), 7.14 (dd, *J* = 8.4, 1.7 Hz, 1H), 6.68 (dd, *J* = 8.2, 2.5 Hz, 1H), 4.45 (s, 2H), 3.76 (s, 3H), 2.40 (s, 3H); ^13 ^C NMR: 165.99, 165.32, 164.17, 159.89, 150.09, 142.14, 141.88, 140.93, 134.58, 130.11, 129.82, 129.23, 125.65, 118.86, 118.67, 113.01, 110.10, 109.50, 106.48, 55.47, 37.27, 21.40; MS (*m/z*) for C_24_H_21_N_3_O_4_S (447.51): 447.32 (M^+^, 100%).

##### 4.1.1.10. 4–(2-(Benzo[d]oxazol-2-ylthio)acetamido)-N-(3-chlorophenyl)benzamide 12j

IR: 3384, 3276 (NH), 3066 (CH aromatic), 2981 (CH aliphatic), 1657 (C = O); ^1^H NMR: 10.80 (s, 1H), 10.33 (s, 1H), 8.03–7.93 (m, 3H), 7.82–7.63 (m, 5H), 7.43–7.31 (m, 3H), 7.17 (td, *J* = 9.2, 8.1, 2.2 Hz, 1H), 4.34 (d, *J* = 96.7 Hz, 2H); ^13 ^C NMR: 166.00, 165.55, 164.30, 151.83, 142.34, 141.68, 141.23, 133.41, 130.75, 129.34, 124.85, 120.16, 118.92, 118.74, 110.72, 37.29. MS (*m/z*) for C_22_H_16_ClN_3_O_3_S (437.90): 437.33 (M^+^, 10%), 120.20 (100%).

##### 4.1.1.11. 4–(2-((5-Chlorobenzo[d]oxazol-2-yl)thio)acetamido)-N-(3-chlorophenyl)benzamide 12k

IR: 3379, 3265 (NH), 3093 (CH aromatic), 2980 (CH aliphatic), 1644 (C = O); ^1^H NMR: 10.82 (s, 1H), 10.33 (s, 1H), 8.10–7.91 (m, 3H), 7.85–7.59 (m, 5H), 7.42–7.32 (m, 2H), 7.14 (dd, *J* = 8.0, 2.1 Hz, 1H), 4.48 (s, 2H); ^13 ^C NMR: 166.39, 165.79, 165.52, 150.61, 142.96, 142.31, 141.24, 133.41, 130.70, 129.34, 124.73, 120.15, 118.91, 118.49, 111.94, 37.46; MS (*m/z*) for C_22_H_15_Cl_2_N_3_O_3_S (472.34): 472.70 (M^+,^ 30%) 345.20 (100%).

##### 4.1.1.12. N-(3-Chlorophenyl)-4–(2-((5-methylbenzo[d]oxazol-2-yl)thio)acetamido)benzamide 12 l

IR: 3384, 3181 (NH), 3034 (CH aromatic), 2970 (CH aliphatic), 1651 (C = O); ^1^H NMR 10.83 (s, 1H), 10.35 (s, 1H), 8.26–6.84 (m, 11H), 4.45 (s, 2H), 2.44 (s, 3H); MS (*m/z*) for C_23_H_18_ClN_3_O_3_S (451.93): 451.30 (M^+^, 100%).

#### 4.1.2. General procedure for preparation of the target compounds 13a-c

In 10 ml DMF containing 0.001 mol KI, 0.001 mol of **3a-c** and 0.001 mol of *N*-(4–(2-benzoylhydrazine-1-carbonyl)phenyl)-2-chloroacetamide **11**, were mixed well and refluxed for 6 h. The reaction content was then poured on crushed ice. The collected crystals were filtered and crystalised from methanol to afford **13a-c**.

##### 4.1.2.1. 2-(Benzo[d]oxazol-2-ylthio)-N-(4–(2-(3-chlorobenzoyl)hydrazine-1-carbonyl)phenyl)- acetamide 13a

IR: 3384, 3279 (NH), 3014 (CH aromatic), 2853 (CH aliphatic), 1660 (C = O); ^1^H NMR: 10.79 (s, 1H), 10.65 (s, 1H), 10.53 (s, 1H), 8.06–7.85 (m, 4H), 7.76 (d, *J* = 8.3 Hz, 2H), 7.66 (q, *J* = 8.7, 8.2 Hz, 3H), 7.58 (t, *J* = 7.8 Hz, 1H), 7.41–7.29 (m, 2H), 4.47 (s, 2H); MS (*m/z*) for C_23_H_17_ClN_4_O_4_S (480.92): 480.42 (M^+^, 10%), 311.23 (100%).

##### 4.1.2.2. 2-((5-Chlorobenzo[d]oxazol-2-yl)thio)-N-(4–(2-(3-chlorobenzoyl)hydrazine-1-carbonyl)- phenyl)acetamide 13 b

IR: 3279, 3167 (NH), 3017 (CH aromatic), 2855 (CH aliphatic), 1656 (C = O); ^1^H NMR: 10.79 (s, 1H), 10.65 (s, 1H), 10.53 (s, 1H), 8.10–7.85 (m, 4H), 7.81–7.72 (m, 3H), 7.69 (dd, *J* = 9.0, 3.4 Hz, 2H), 7.58 (t, *J* = 7.9 Hz, 1H), 7.37 (dd, *J* = 8.7, 2.2 Hz, 1H), 4.48 (s, 2H); ^13 ^C NMR: 166.39, 165.80, 165.04, 150.61, 142.96, 134.99, 133.87, 131.07, 129.48, 129.08, 127.75, 126.66, 124.76, 119.02, 118.51, 111.97, 37.44; MS (*m/z*) for C_23_H_16_Cl_2_N_4_O_4_S (515.37): 415.76 (M^+^, 10%), 345.24 (100%).

##### 4.1.2.3. N-(4–(2-(3-Chlorobenzoyl)hydrazine-1-carbonyl)phenyl)-2-((5-methylbenzo[d]oxazol-2-yl)thio)acetamide 13c

IR: 3279 (NH), 3015 (CH aromatic), 2927 (CH aliphatic), 1657 (C = O); ^1^H NMR: 10.78 (s, 1H), 10.67 (s, 1H), 10.55 (s, 1H), 8.01–7.89 (m, 4H), 7.78 (d, *J* = 8.3 Hz, 2H), 7.71–7.64 (m, 1H), 7.58 (t, *J* = 7.9 Hz, 1H), 7.51 (d, *J* = 8.3 Hz, 1H), 7.42 (s, 1H), 7.11 (d, *J* = 8.3 Hz, 1H), 4.46 (s, 2H), 2.39 (s, 3H); ^13 ^C NMR: 166.05, 165.76, 165.07, 164.16, 150.10, 142.41, 141.89, 135.00, 134.55, 133.89, 131.05, 129.08, 127.77, 126.66, 125.62, 119.02, 110.05, 37.32, 21.39; MS (*m/z*) for C_24_H_19_ClN_4_O_4_S (494.95): 494.47 (M^+^, 10%), 402.37 (100%).

### 4.2. Biological evaluation

#### 4.2.1. *In vitro* anti-proliferative activity

MTT assay protocol^32^. This method was applied in accordance with the comprehensive description in Supplementary data.

#### 4.2.2. *In vitro* VEGFR-2 kinase assay

The assay was applied by ELISA kits in accordance with the comprehensive description in^18^^,^^33^ as described in Supplementary data.

#### 4.2.3. Flow cytometry analysis for cell cycle

This assay was applied using propidium iodide (PI) staining in accordance with the comprehensive description in Supplementary data^34^^,^^35^

#### 4.2.4. Flow cytometry analysis for apoptosis

Apoptotic effect was applied in accordance with the comprehensive description in Supplementary data^36^^,^^37^

#### 4.2.5. Western blot analysis

The western blot technique was applied in accordance with the comprehensive description in Supplementary data^38–40^

### 4.3. *In silico* studies

#### 4.3.1. Docking studies

Docking studies were applied using MOE 2014^41–43^ in accordance with the comprehensive description in were carried out against VEEGFR-2 (PDB ID: 4ASD, resolution: 2.03 Å) as described in Supplementary data.

#### 4.3.2. Pharmacokinetic profiling study

This study was applied using Discover studio 4 in accordance with the comprehensive description in Supplementary data^44^.

#### 4.3.3. ADME studies

was used to compute the physicochemical properties and predict the drug likeness properties of the most potent compounds This study was applied using the SwissADME online web tool in accordance with the comprehensive description in Supplementary data^45–47^.

## Supplementary Material

Supplemental Material
