# Supplementary material for: Benzoxazole derivatives as new VEGFR-2 inhibitors and apoptosis inducers: design, synthesis, in silico studies, and antiproliferative evaluation
Source: J Enzyme Inhib Med Chem. 2022 Jul 25;37(1):2063–77. doi: 10.1080/14756366.2022.2103552 (PMC9327782; doi:10.1080/14756366.2022.2103552)

Benzoxazole derivatives as new VEGFR-2 inhibitors and apoptosis inducer: design, synthesis, *in silico* studies, and antiproliferative evaluation

Mohammed S. Taghour[a], Hazem A. Mahdy[a], Maher H. Gomaa[b], Ahmed Aglan[b], Mahmoud Gomaa Eldeib[b], Alaa Elwan[a], Mohammed A. Dahab[a], Eslam B. Elkaeed[c], Aisha A. Alsfouk[d], Mohamed M. Khalifa[a], Ibrahim H. Eissa[a]*, Hazem Elkady[a]*

[a] Pharmaceutical Medicinal Chemistry & Drug Design Department, Faculty of Pharmacy (Boys), Al-Azhar University, Cairo 11884, Egypt.

[b] Biochemistry and Molecular Biology Department, Faculty of Pharmacy (Boys), Al-Azhar University, Cairo 11884, Egypt.

[c] Department of Pharmaceutical Organic Chemistry, Faculty of Pharmacy (Boys), Al-Azhar University, Nasr City, 11884, Cairo, Egypt.

[d] Department of Pharmaceutical Sciences, College of Pharmacy, Princess Nourah bint Abdulrahman Uni-versity, P.O. Box 84428, Riyadh 11671, Saudi Arabia

*** Corresponding authors:**

Ibrahim H. Eissa

Pharmaceutical Medicinal Chemistry & Drug Design Department, Faculty of Pharmacy (Boys), Al-Azhar University, Cairo 11884, Egypt

Email: Ibrahimeissa@azhar.edu.eg

Hazem Elkady:

Pharmaceutical Medicinal Chemistry & Drug Design Department, Faculty of Pharmacy (Boys), Al-Azhar University, Cairo 11884, Egypt.

Email: Hazemelkady@azhar.edu.eg

Content

**1**	Experimental chemistry
**2**	Experimental of biological testing
**3**	Molecular docking
**4**	Spectral data of the final target compounds **12a-l** & **13a-c**
**5**	Raw data of cytotoxity and VEGFR-2 assay

Experimental chemistry

All solvents and reagents were commercially available and used without further purification. Reactions progression were monitored by TLC sheets coated with UV fluorescent silica gel (Kieselgel 0.25mm, 60 F254, Merck Germany) and were visualized using UV lamp. The melting points were determined using a Gallen lamp melting point apparatus. The infrared spectra were recorded on FT/IR-6600typeA spectrophotometer. ^{1}H NMR spectra were recorded at 400 and 700 MHz, while ^{13}C NMR spectra were run at 100 and 176 MHz, on a Bruker Avance NEO-600 equipped with a 1.7 mm TCI CryoProbe. Chemical shifts were expressed in δ (ppm) with reference to TMS and coupling constant (J) in Hertz using DMSO-d_6 and $CDCl_3$-d_6 as solvents. The mass spectra were recorded on an Agilent 6410 triple-quadrupole mass spectrometer equipped with an ESI source.

Experimental of biological testing

1. *In vitro* anti-proliferative activity

MTT assay protocol was applied to assess the anti-proliferative activity of the synthesized compounds. Two human cancer cell lines (MCF-7 and HepG-2) and one normal cell line (WI-38) were used in this test. At first, the cell lines were cultured in RPMI-1640 medium with 10% fetal bovine serum. Antibiotics (100 units/ml penicillin and100 µg/ml streptomycin) were added at 37°C in a 5% CO_2 incubator. The cell lines were seeded in a 96-well plate at a density of 1.0 x 104 cells / well at 37 °C for 24 h under 5% CO_2. After incubation, the cells were treated with different

concentration of the synthesized compounds and incubated for 24 h. After 24 h of drug treatment, 20 µl of MTT solution at 5mg/ml was added and incubated for 4 h. Dimethyl sulfoxide (DMSO) in volume of 100 µl was added into each well to dissolve the purple formazan formed. The colorimetric assay was measured and recorded at absorbance of 570 nm using a plate reader (EXL 800, USA). The relative cell viability in percentage was calculated as (A570 of treated samples/A570 of untreated sample) X 100.

2. *In vitro* VEGFR-2 kinase assay

All the synthesized compounds were tested for its inhibitory activity against VEGFR-2. Human VEGFR-2 ELISA kit (Enzyme-Linked Immunosorbent Assay) was utilized in this test. At first, specific antibody for VEGFR-2 was seeded on a 96-well plate and 100 µL of the standard solution or the tested compound was added, all were incubated at room temperature for 2.5 h. Then washed, 100 µL of the prepared biotin antibody was added, then incubated at room temperature for additional 1 h. and washed. Then, 100 µL of streptavidin solution was added and incubated for 45 min. at room temperature. Washed again, 100 µL of TMB Substrate reagent was added and incubated for 30 min. at room temperature. 50 µL of the stop solution was added, then read at 450 nm immediately. The standard curve was drawn, concentrations on the X-axis and the absorbance on the Y-axis.

3. Flow cytometry analysis for cell cycle

To determine the role of the synthesized compounds in cell cycle distribution, cell cycle analysis was performed using propidium iodide (PI) staining and flow cytometry analysis for compound **12l**. Flow Cytometry Kit for Cell Cycle Analysis (ab139418_Propidium Iodide Flow Cytometry Kit/BD) was used in this test. HepG2 cells were treated with compound **12l** (10.50 µM) for 24 h. Then, the cells were fixed in 70% ethanol at 4 °C for 12 h. After that, the cells were washed with cold PBS, incubated with 100 µl RNase A at 37 °C for 30 min, and stained with 400 µl PI in the dark at room temperature for further 30 min. The stained cells were measured using Epics XL-MCL™ Flow Cytometer (Beckman Coulter), and the data were analyzed using Flowing software (version 2.5.1, Turku Centre for Biotechnology, Turku, Finland).

4. **Flow cytometry analysis for apoptosis**

Flow cytometry cell apoptosis analysis was used to investigate the apoptotic effect of the synthesized compounds. HepG2 cells were treated with compound **12l** (10.50 µM) for 24 h, collected by trypsin, centrifuged, washed two successive times with PBS, suspended in 500 µl binding buffer, and double stained with 5 µl Annexin V-FITC and 5 µl PI in the dark at room temperature for 15 min. The stained cells were measured using Epics XL-MCL™ Flow Cytometer and analyzed using Flowing software.

5. **Western blot analysis**

The proteins expression of cleaved caspase-3, BAX, and Bcl-2 were determined using Western blot analysis. In brief, HepG2 cells were treated with control, or with the synthesized compounds. Then cells were lysed in 250 µL precold lysis buffer (pH 7.4: Tris-Base [10 mM], NaCl [100 mM], ethylenediaminetetraacetic acid [EDTA, 25 mM], ethylene glycol bis (2-aminoethyl) tetraacetic acid [EGTA, 25 mM], 1% [v/v] NP-40, and 1% [v/v] Triton X-100) supplemented with 1:350 protease:phosphatase inhibitors cocktail (Sigma). The cells were immediately frozen at −20°C for 1.5 hours for further lysis, collected by cell scraper, sonicated 3 × 10 seconds, and centrifuged (13000 rpm, 15 minutes). Total protein concentrations in the supernatant were determined colorimetrically using the Pierce 660 nm Protein Assay method (Thermo Fisher Scientific, Rockford, IL), with BSA as the standard. Equal amounts of protein (25 µg) samples were mixed with SDS-loading buffer (pH 6.8: Tris-HCl [700 mM], dithiothreitol [DTT, 600 mM], sodium dodecyl sulfate [SDS, 12%], glycerol [60%], and bromophenol blue [0.012%]), denatured by boiling at 95°C for 10 minutes, allowed to cool on ice for 15 minutes, vortexed vigorously for 30 seconds and loaded into SDS-polyacrylamide gel and separated by an electrophoresis unit (Cleaver Scientific Ltd, UK), transferred onto polyvinylidene fluoride membranes (Bio-Rad) for 35 minutes using a Trans-Blot SD semi-dry transfer cell (Bio-Rad) at 250 mA and 22 V. Membranes were blocked with 5% (w/v) blotting grade dry milk (Bio-Rad) in Tris-buffered saline/Tween-20 (TBS-T) (pH 7.5: Tris-base [20 mM], NaCl [150 mM], and 0.05% [v/v] Tween- 20) while shaking for 1.5 hour at RT, and then incubated with the corresponding primary antibody against cleaved caspase-3 antibody (1:000, #9661; Cell Signaling Technology), β-actin (1:2000, #A5060; Sigma), BAX (Biovision, USA) and Bcl-2(Bioimaging, system, syngene, UK) for 9-10 hours at 4°C in a

humidified chamber. The blots were washed with TBS-T three times for 15 minutes and incubated with matched horseradish peroxidase (HRP)-linked secondary antibodies (Dako, Denmark) for another 1 hour at RT, followed by washing 3 × 15 min with TBS-T. After membranes were incubated at RT with 1:1 reagent mixture of chemiluminescence Western Lightning ECL (Perkin Elmer, Waltham, MA) for 1 minute, the bands were visualized in Chemi-Doc imager (Bio-Rad). Means of the detected blot intensities were then quantified, analyzed by the combined Bio-Rad Image Lab software and their corresponding background subtracted, with normalization to the corresponding bands density of β-actin as the sampling loading control. Data were collected from three separate experiments.

Molecular docking

The docking studies were performed utilizing MOE.14 software to explore the binding mode of the synthesized compounds towards VEGFR-2. The crystal structure of VEGFR-2 was downloaded from the Protein Data Bank, http://www.rcsb.org/pdb (PDB ID: 4ASD, resolution: 2.03 Å). Sorafenib was used as reference ligand. To prepare the target protein, water molecules were removed, and the valances of atoms were corrected through protonation of the whole molecule. Then energy minimization was carried out by applying CHARMM and MMFF94 force fields. After that, the active binding site was defined and prepared for docking. The validation process was performed by redocking the co-crystallized ligand. The designed compounds together with sorafenib were drawn using ChemBioDraw Ultra 14.0 and saved as MDL-SD format. The sketched compounds were constructed from fragment libraries in MOE program, protonated, followed by energy minimization then prepared for docking. Docking process was carried through Triangle matcher placement inserted in compute window, and the scoring function was London dG. Ten conformers (poses) for each molecule were generated using genetic algorithm searches. The free energies and binding modes of the designed molecules against VEGFR-2 were determined. The most ideal pose was selected according to its binding free energy as well as its binding mode with target molecule.

IR Of Comp. 12a

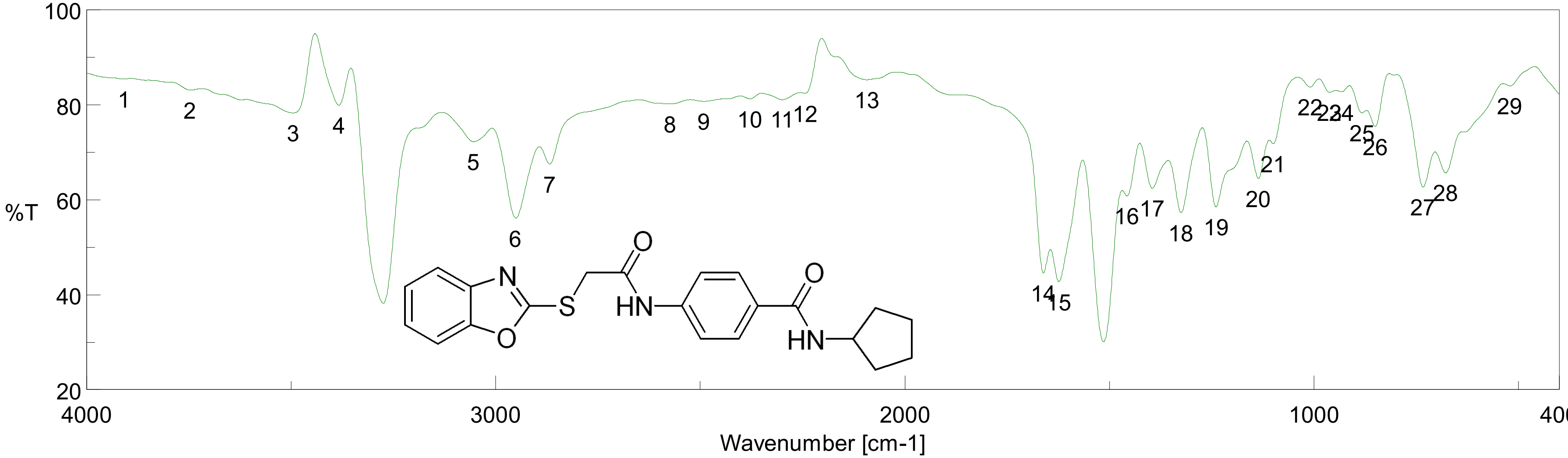

[Result of Peak Picking]

No.	Position	Intensity	No.	Position	Intensity	No.	Position	Intensity	No.	Position	Intensity
1	3908.04	85.3838	2	3747.98	83.0731	3	3495.35	78.2588	4	3383.5	79.8707
5	3054.69	72.2118	6	2951.52	56.1004	7	2868.59	67.4987	8	2573.54	80.1348
9	2491.58	80.6992	10	2378.76	81.2314	11	2298.73	81.0459	12	2243.77	82.3725
13	2092.39	85.2405	14	1661.37	44.5511	15	1623.77	42.7178	16	1456.96	60.806
17	1395.25	62.4006	18	1324.86	57.2485	19	1239.04	58.4815	20	1135.87	64.4438
21	1099.23	71.7807	22	1008.59	83.6986	23	961.341	82.5895	24	932.414	82.6969
25	882.274	78.2642	26	849.49	75.4079	27	732.817	62.6592	28	677.856	65.6478
29	520.686	83.9556									

^{1}H NMR of 12a

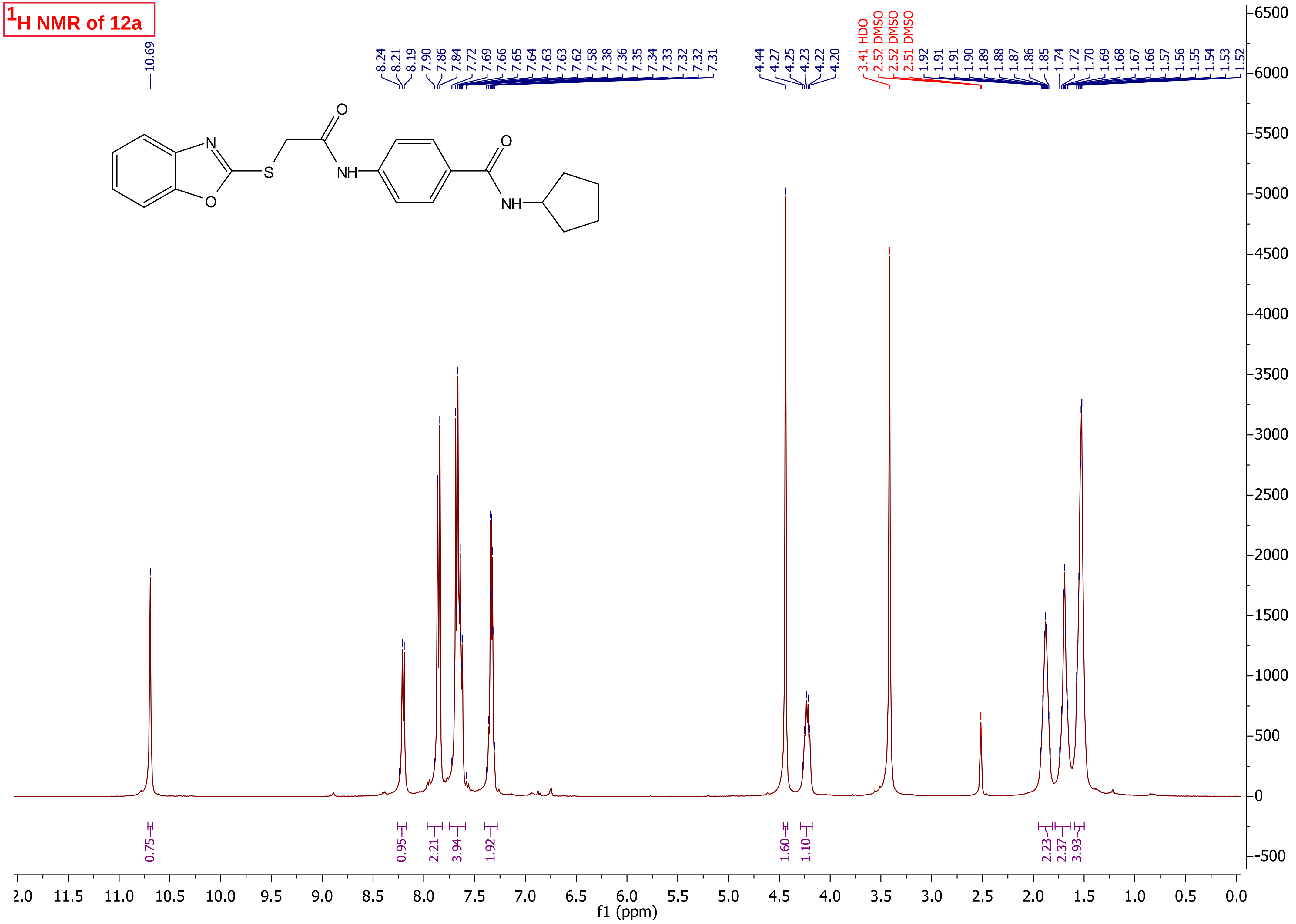
10.69
8.24
8.21
8.19
7.90
7.86
7.84
7.72
7.69
7.66
7.65
7.64
7.63
7.63
7.62
7.58
7.38
7.36
7.35
7.34
7.33
7.32
7.32
7.31
4.44
4.27
4.25
4.23
4.22
4.20
3.41 HDO
2.52 DMSO
2.52 DMSO
2.51 DMSO
1.92
1.91
1.91
1.90
1.89
1.88
1.87
1.86
1.85
1.74
1.72
1.70
1.69
1.68
1.67
1.66
1.57
1.56
1.55
1.54
1.53
1.52
O
N
S
NH
O
O
NH
0.75
0.95
2.21
3.94
1.92
1.60
1.10
2.23
2.37
3.93
f1 (ppm)

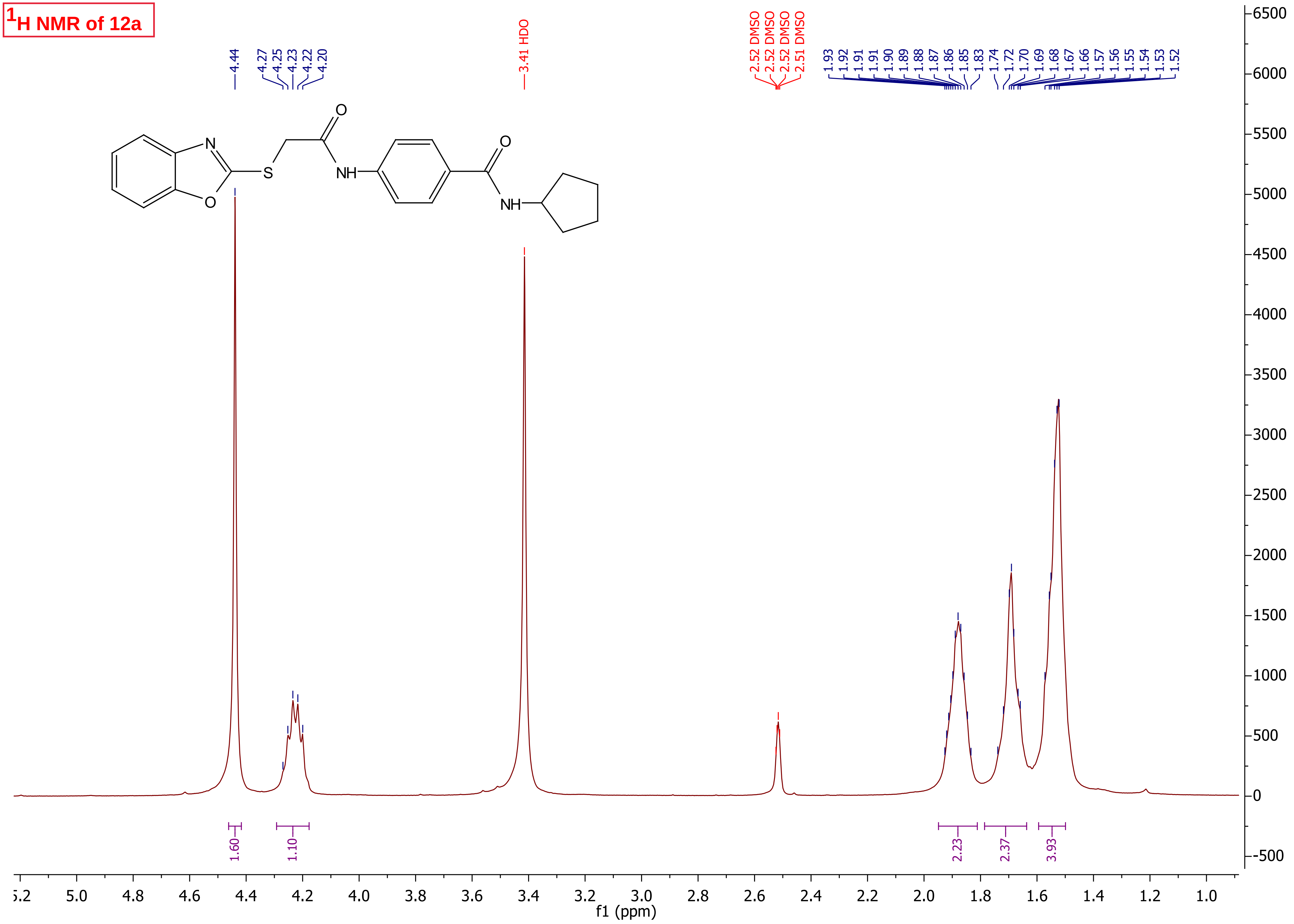

1H NMR of 12a
4.44
4.27
4.25
4.23
4.22
4.20
3.41 HDO
2.52 DMSO
2.52 DMSO
2.52 DMSO
2.51 DMSO
1.93
1.92
1.91
1.91
1.90
1.89
1.88
1.87
1.86
1.85
1.83
1.74
1.72
1.70
1.69
1.68
1.67
1.66
1.57
1.56
1.55
1.54
1.53
1.52
1.60
1.10
2.23
2.37
3.93
f1 (ppm)

^{1}H NMR of 12a

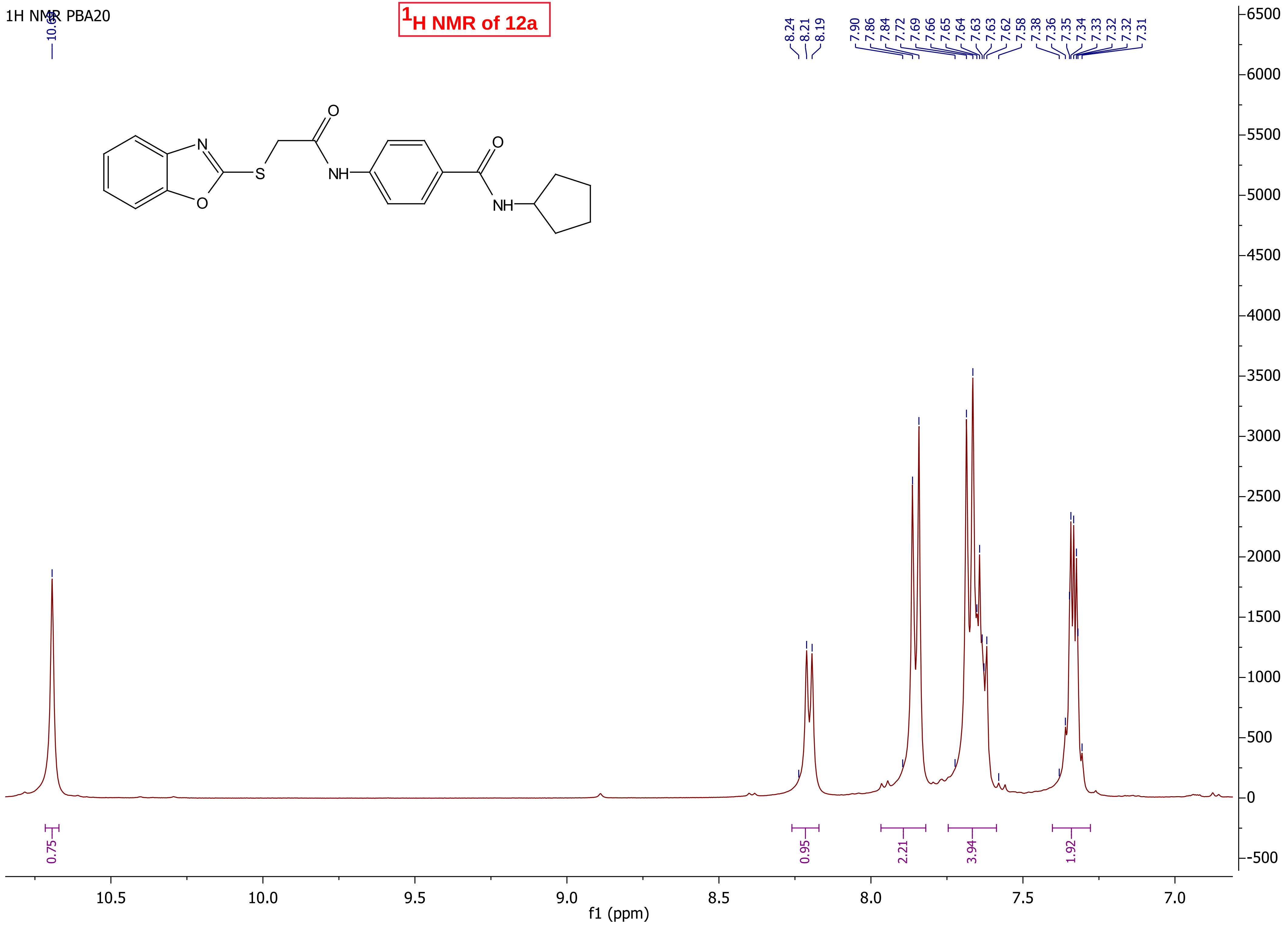

1H NMR PBA20
10.68
8.24
8.21
8.19
7.90
7.86
7.84
7.72
7.69
7.66
7.65
7.64
7.63
7.63
7.62
7.58
7.38
7.36
7.35
7.34
7.33
7.32
7.32
7.31
O
N
S
NH
O
NH
O
0.75
0.95
2.21
3.94
1.92
10.5
10.0
9.5
9.0
8.5
8.0
7.5
7.0
f1 (ppm)
6500
6000
5500
5000
4500
4000
3500
3000
2500
2000
1500
1000
500
0
-500

^{13}C NMR of 12a

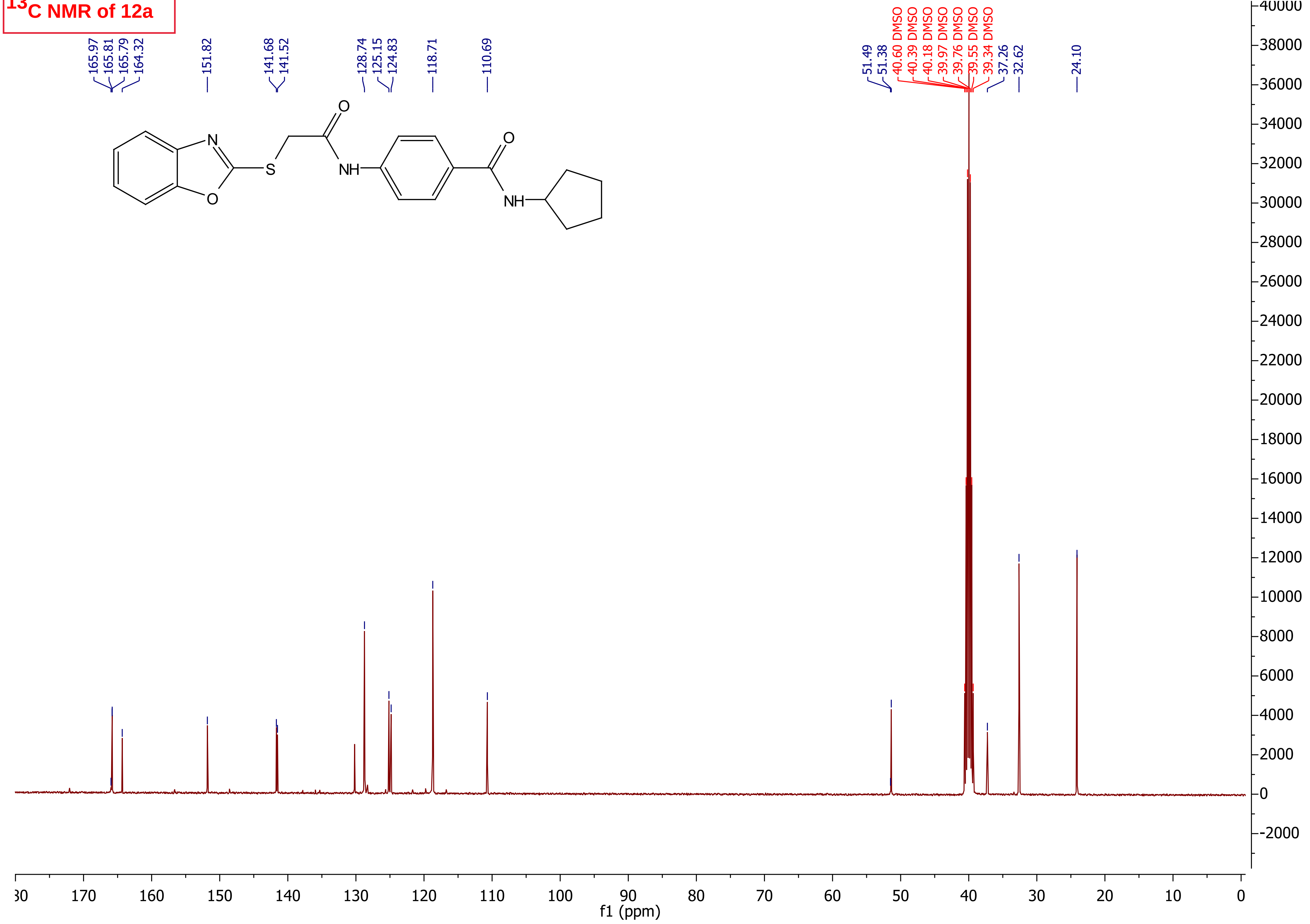

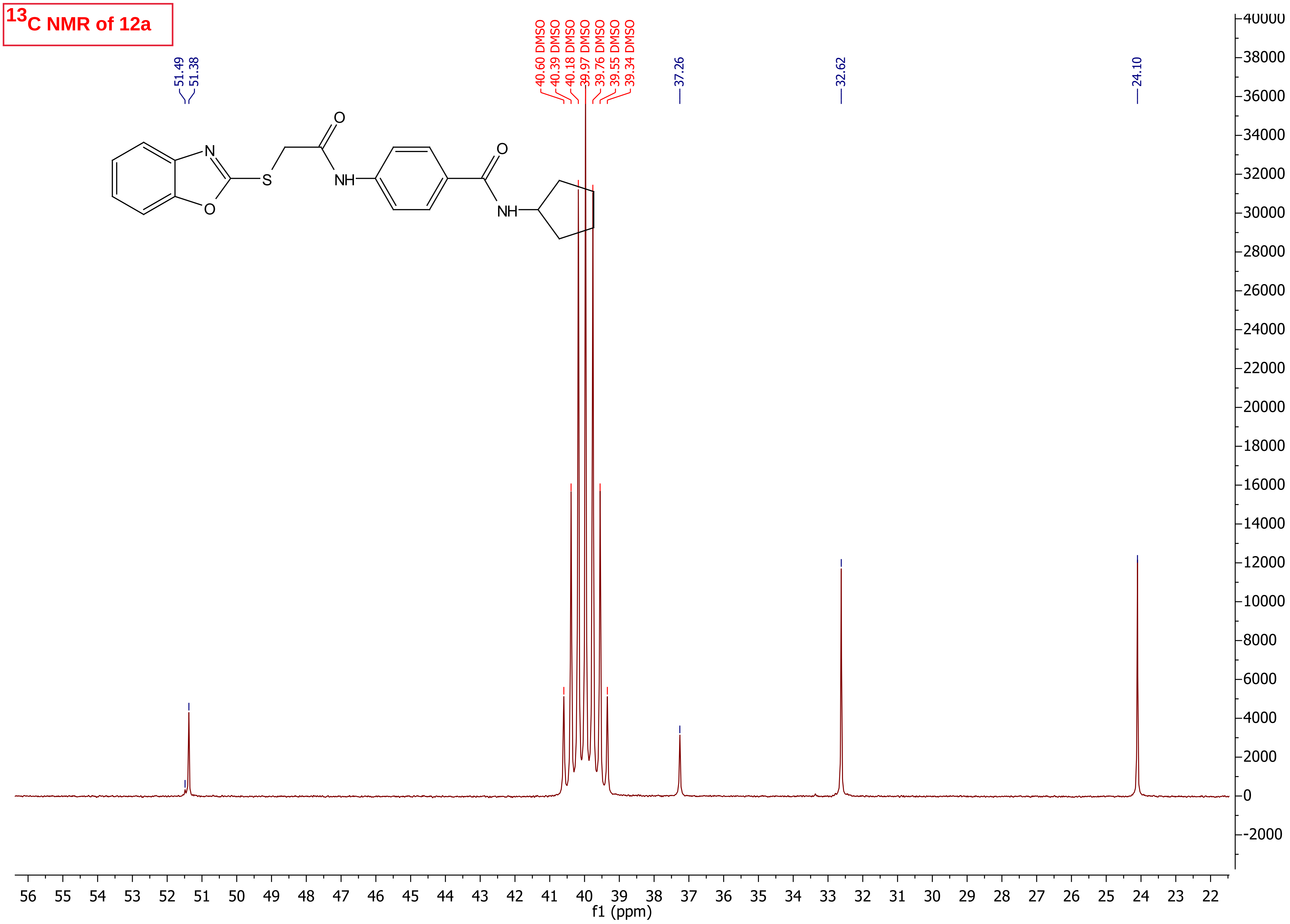

13C NMR of 12a
51.49
51.38
40.60 DMSO
40.39 DMSO
40.18 DMSO
39.97 DMSO
39.76 DMSO
39.55 DMSO
39.34 DMSO
37.26
32.62
24.10
f1 (ppm)

13C NMR of 12a

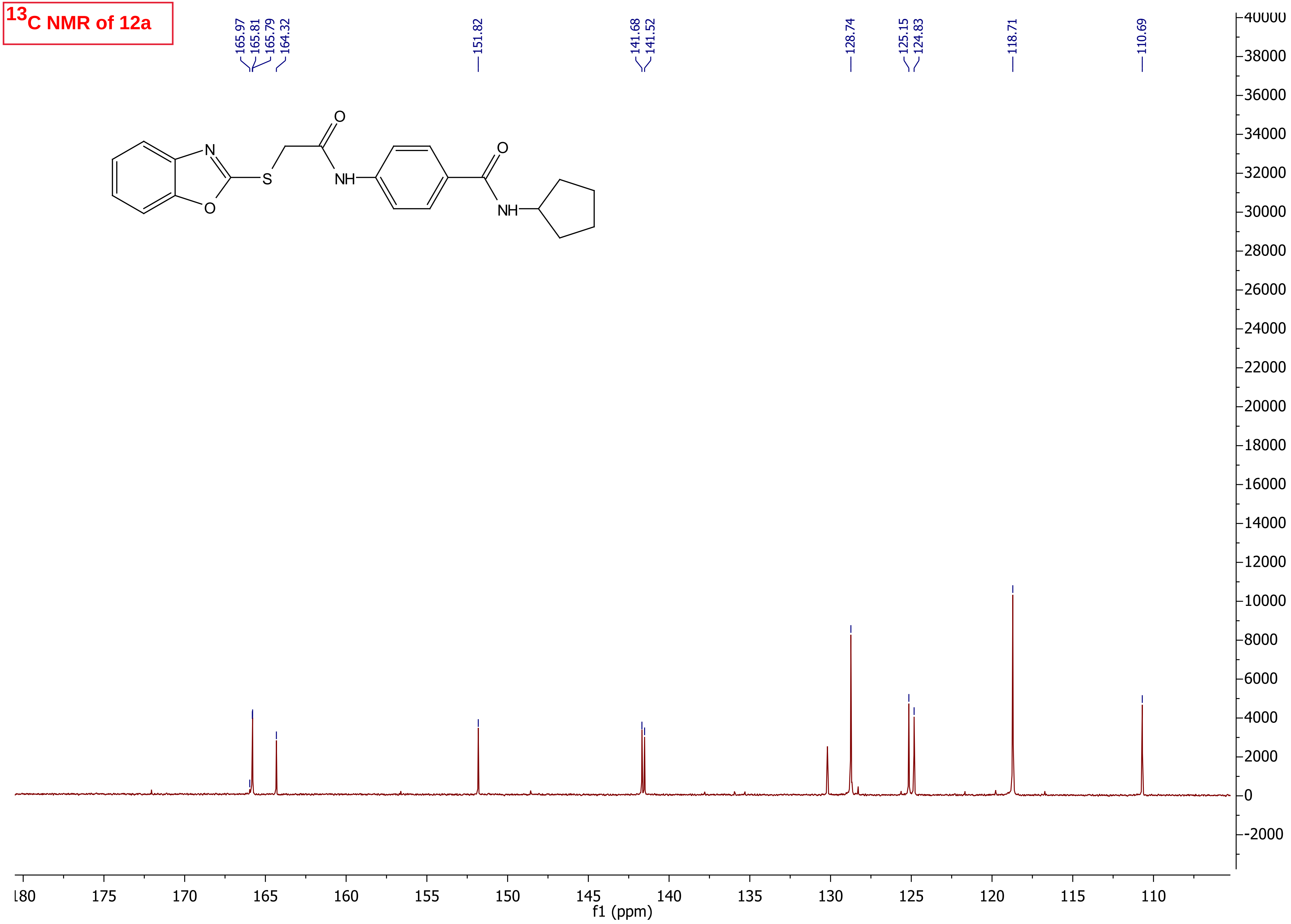

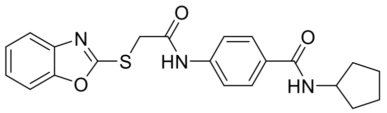

Mass spec. of 12a

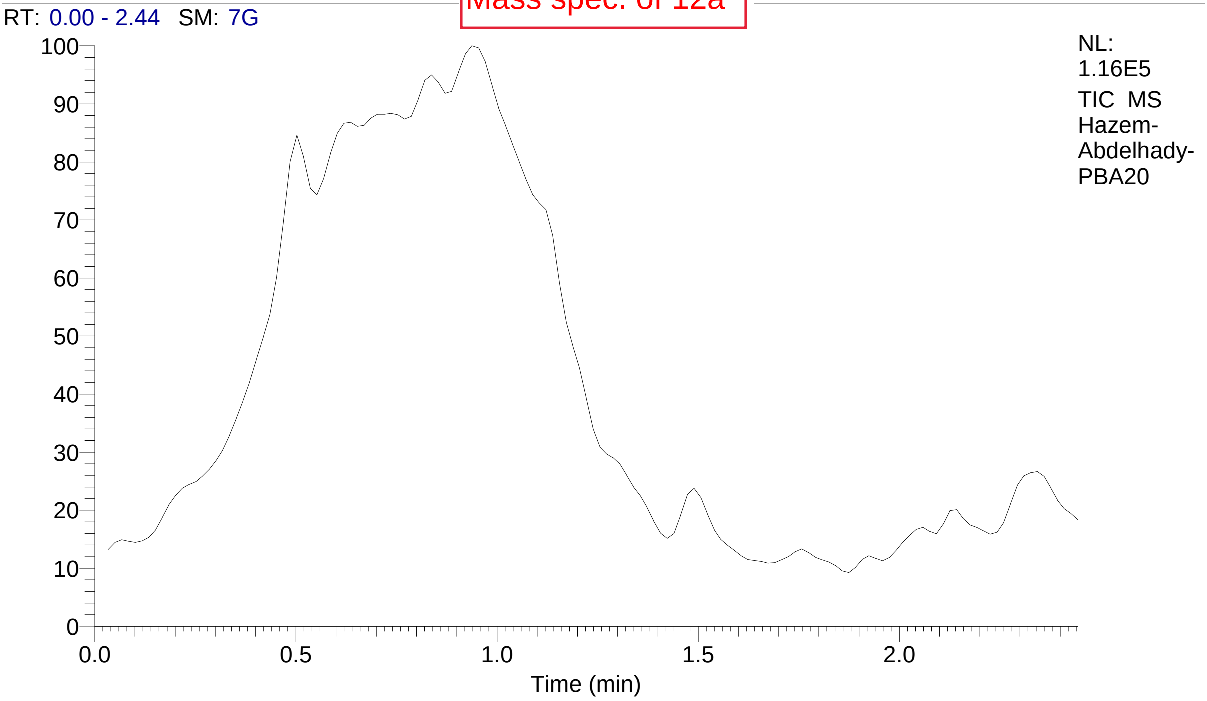

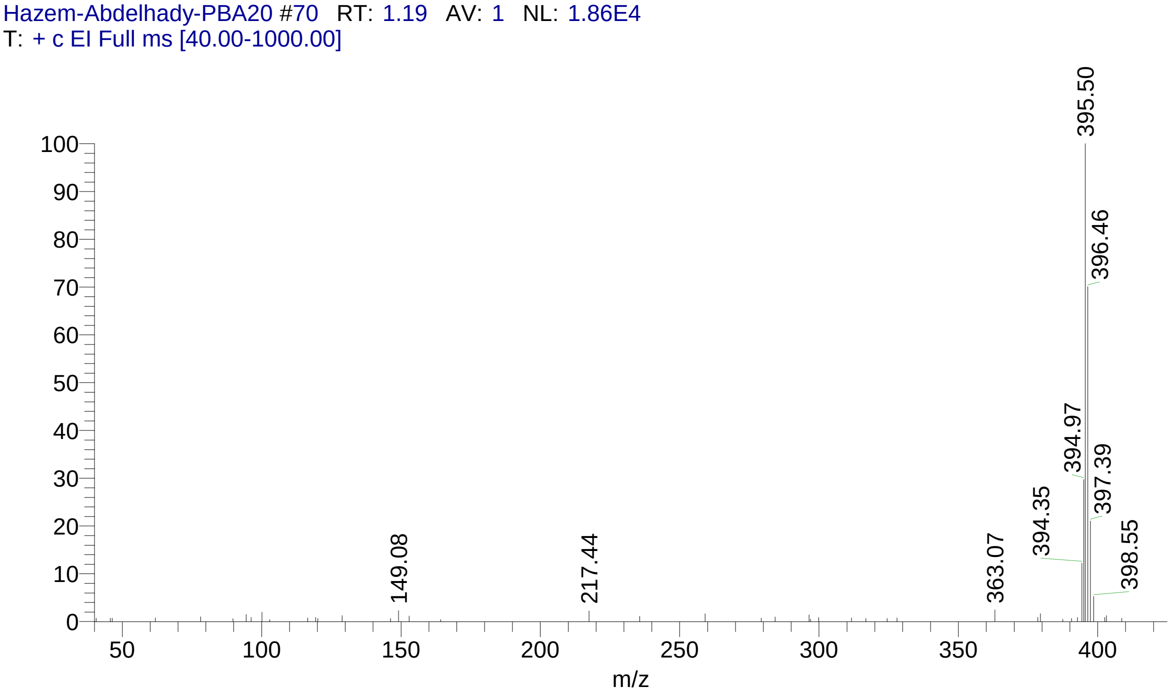

IR Of Comp. 12b

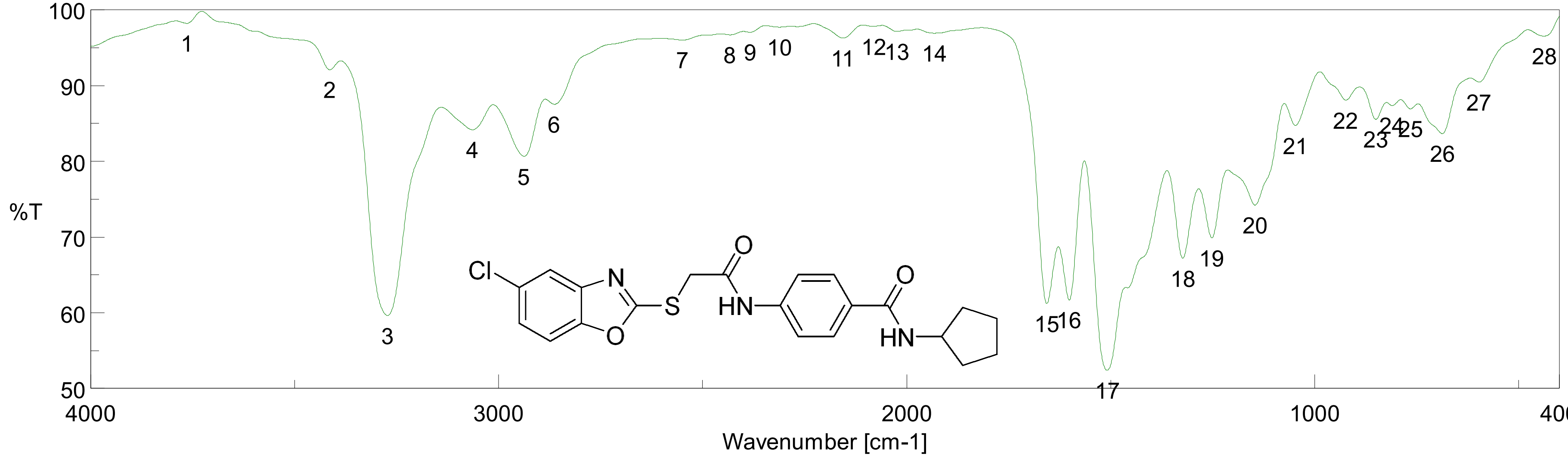

[Result of Peak Picking]

No.	Position	Intensity	No.	Position	Intensity	No.	Position	Intensity	No.	Position	Intensity
1	3764.37	98.1786	2	3414.35	92.0624	3	3272.61	59.6159	4	3064.33	84.1362
5	2938.02	80.6324	6	2863.77	87.5016	7	2549.43	95.9834	8	2432.76	96.6686
9	2383.59	97.0033	10	2311.27	97.6886	11	2156.03	96.2553	12	2080.82	97.7882
13	2023.93	97.1211	14	1932.32	96.8889	15	1656.55	61.1879	16	1601.59	61.6755
17	1508.06	52.3941	18	1322.93	67.1783	19	1251.58	69.8684	20	1145.51	74.1862
21	1046.19	84.7078	22	923.736	88.0384	23	849.49	85.5219	24	809.956	87.3371
25	764.637	86.9007	26	686.534	83.6398	27	596.861	90.4451	28	436.798	96.5154

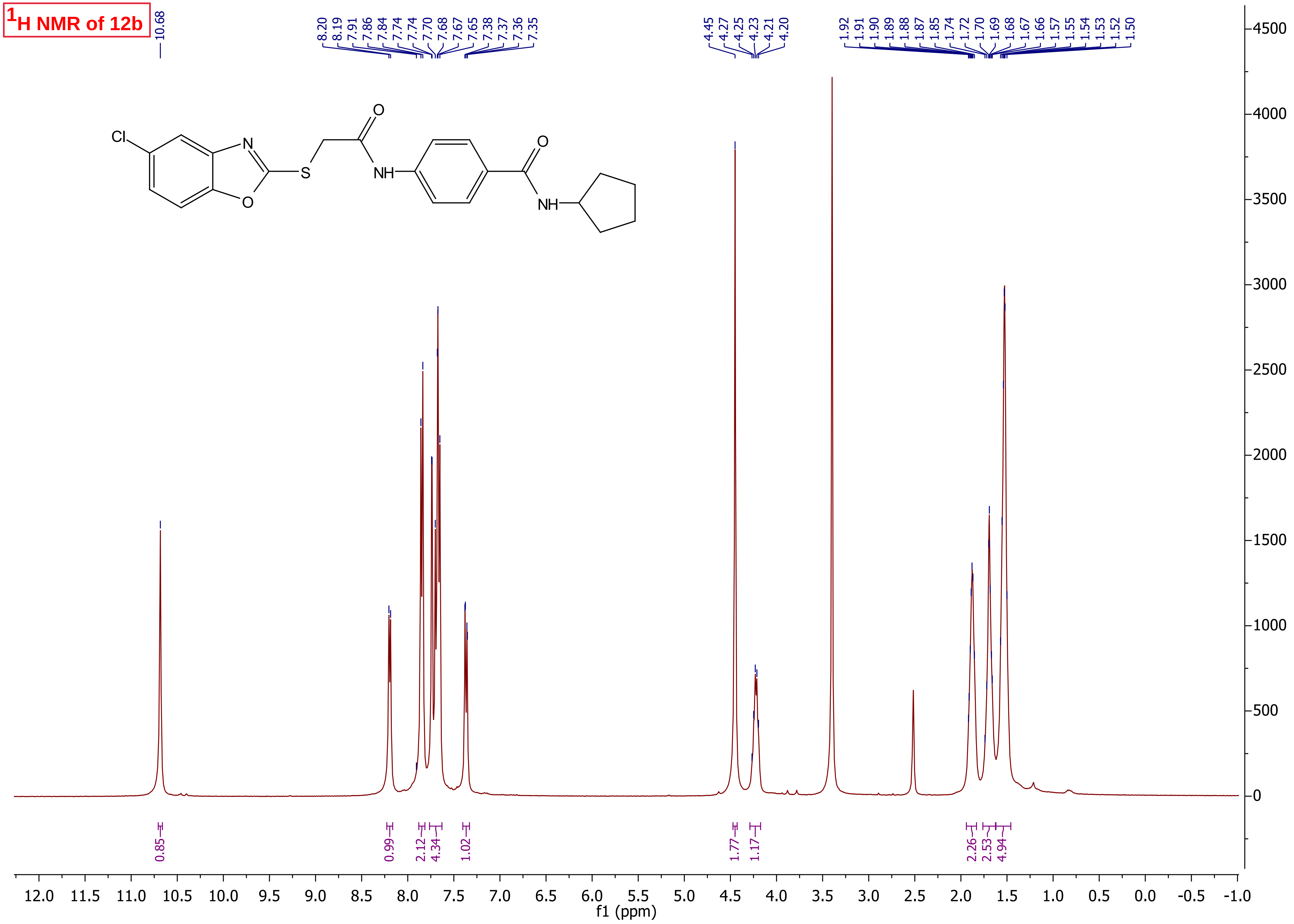

^{1}H NMR of 12b
10.68
8.20
8.19
7.91
7.86
7.84
7.74
7.74
7.70
7.68
7.67
7.65
7.38
7.37
7.36
7.35
4.45
4.27
4.25
4.23
4.21
4.20
1.92
1.91
1.90
1.89
1.88
1.87
1.85
1.74
1.72
1.70
1.69
1.68
1.67
1.66
1.57
1.55
1.54
1.53
1.52
1.50
Cl
N
O
S
O
NH
O
NH
0.85
0.99
2.12
4.34
1.02
1.77
1.17
2.26
2.53
4.94
f1 (ppm)

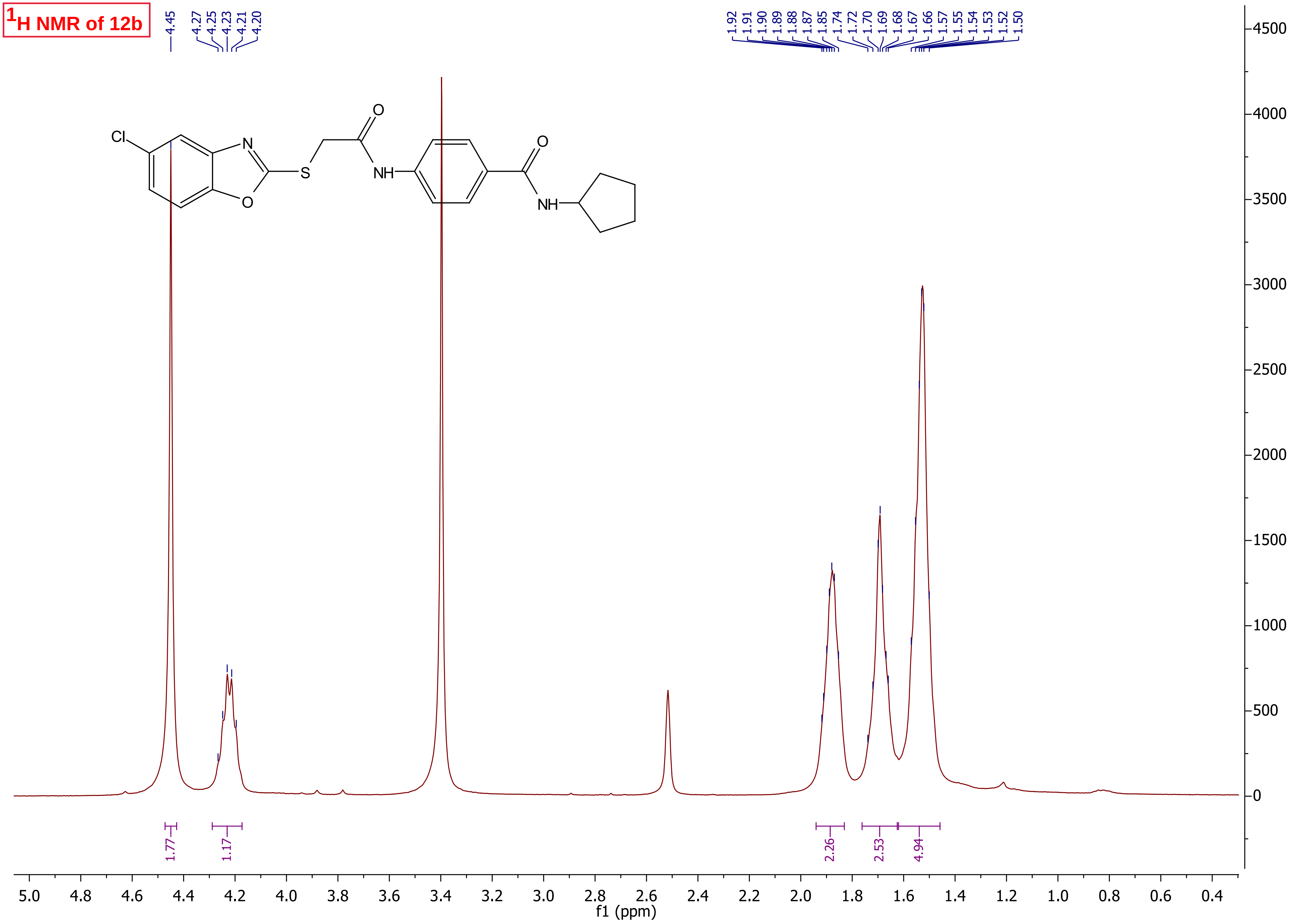

1H NMR of 12b
4.45
4.27
4.25
4.23
4.21
4.20
1.92
1.91
1.90
1.89
1.88
1.87
1.85
1.74
1.72
1.70
1.69
1.68
1.67
1.66
1.57
1.55
1.54
1.53
1.52
1.50
Cl
N
O
S
NH
O
NH
1.77
1.17
2.26
2.53
4.94
f1 (ppm)

^{1}H NMR of 12b

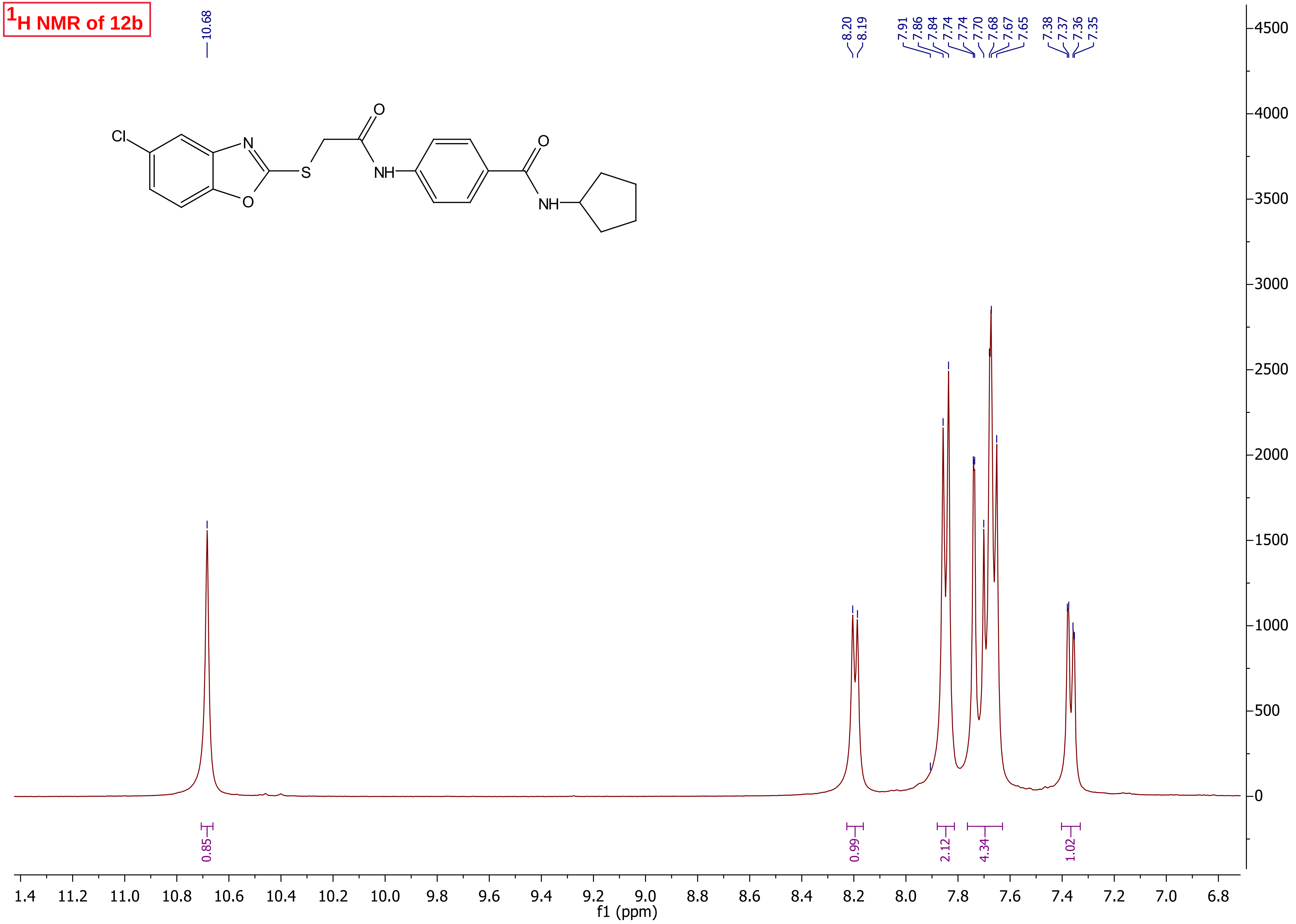
10.68
8.20
8.19
7.91
7.86
7.84
7.74
7.74
7.70
7.68
7.67
7.65
7.38
7.37
7.36
7.35
Cl
N
O
S
O
NH
O
NH
0.85
0.99
2.12
4.34
1.02
f1 (ppm)

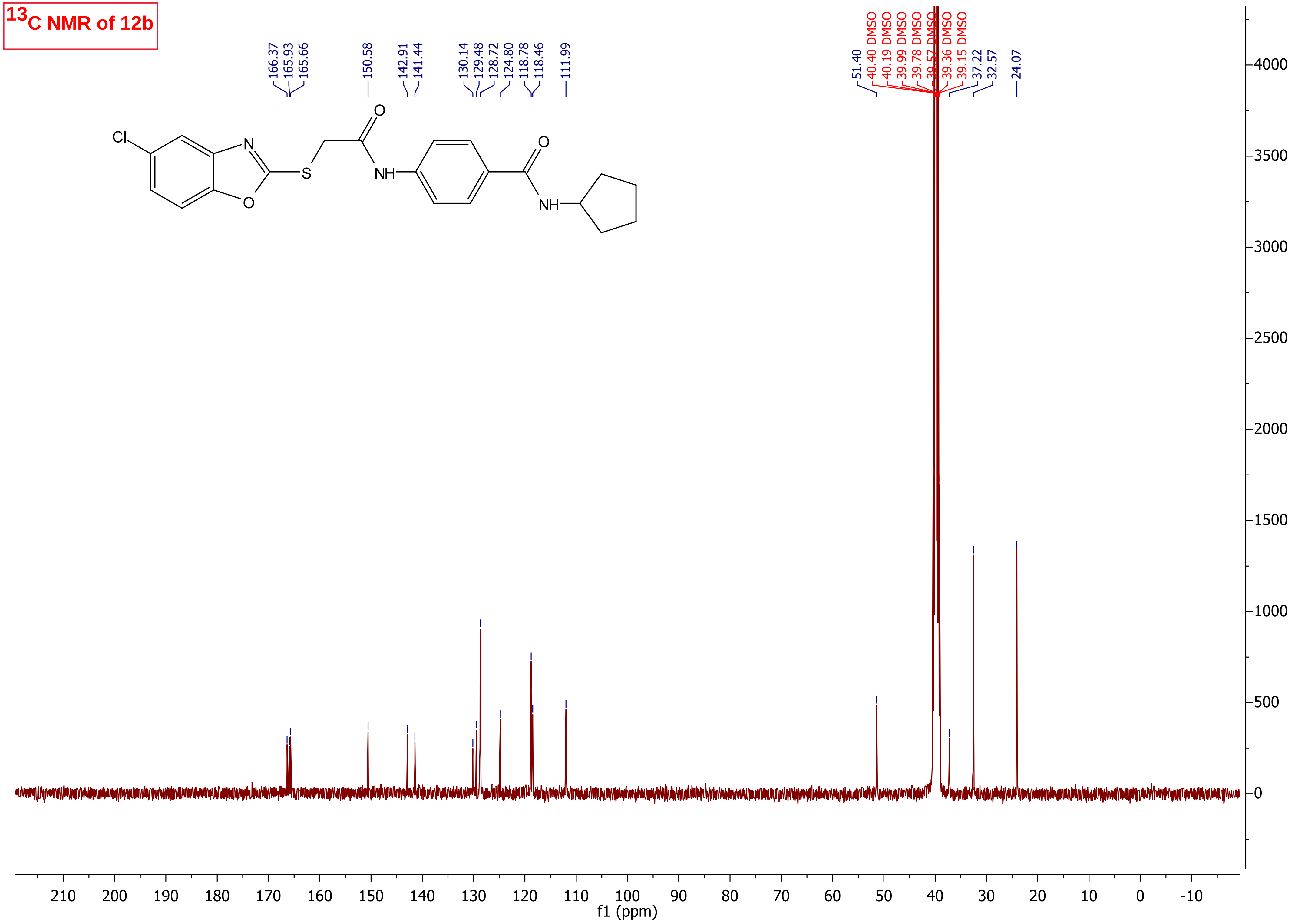
13C NMR of 12b
166.37
165.93
165.66
150.58
142.91
141.44
130.14
129.48
128.72
124.80
118.78
118.46
111.99
51.40
40.40 DMSO
40.19 DMSO
39.99 DMSO
39.78 DMSO
39.57 DMSO
39.36 DMSO
39.15 DMSO
37.22
32.57
24.07
Cl
N
O
S
NH
O
O
NH
f1 (ppm)

13C NMR of 12b

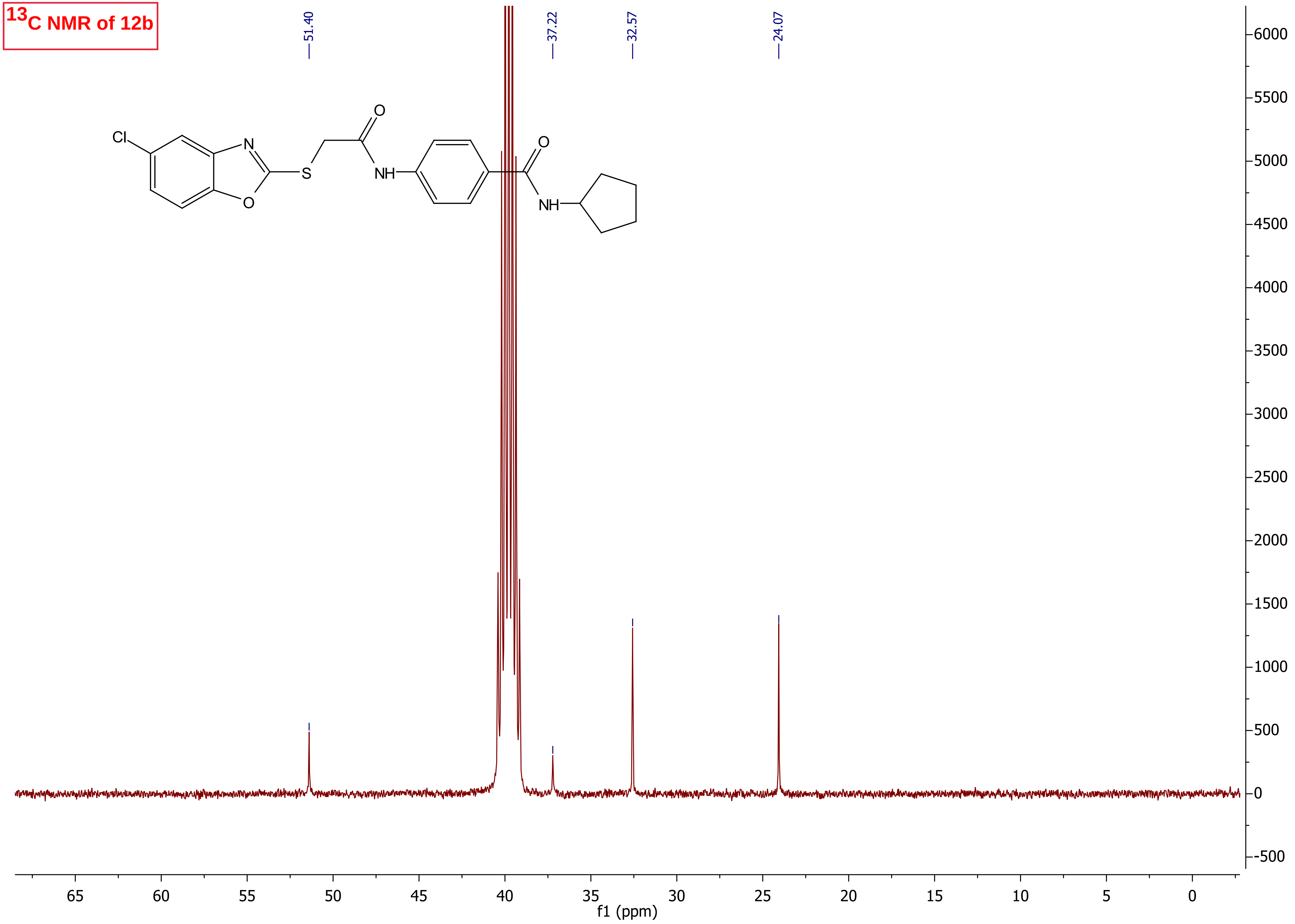

^{13}C NMR of 12b

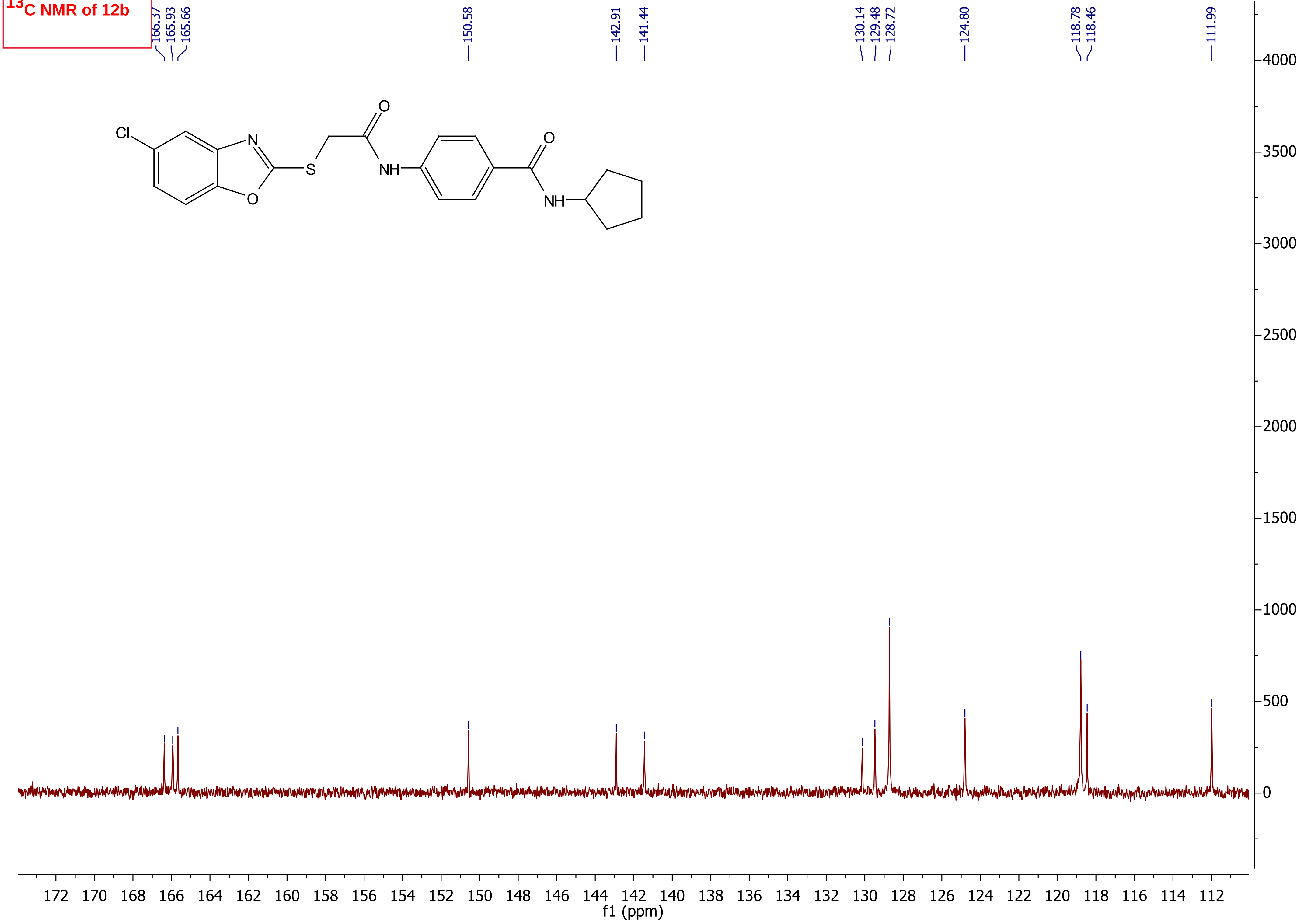

IR Of Comp. 12c

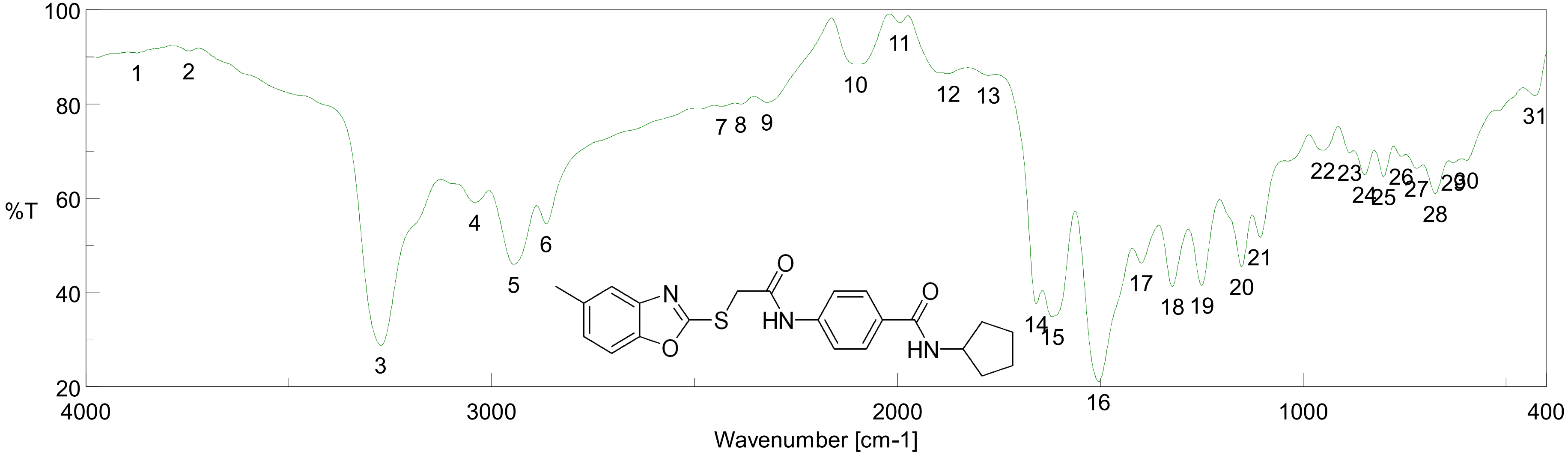

[Result of Peak Picking]

No.	Position	Intensity	No.	Position	Intensity	No.	Position	Intensity	No.	Position	Intensity
1	3874.29	90.7946	2	3746.05	91.2222	3	3273.57	28.7813	4	3041.19	59.0862
5	2945.73	45.9277	6	2865.7	54.5587	7	2433.73	79.4788	8	2385.51	79.9466
9	2320.91	80.3389	10	2102.99	88.4413	11	1994.03	97.25	12	1874.47	86.4445
13	1776.12	86.0541	14	1657.52	37.5794	15	1618.95	34.9054	16	1504.2	21.0482
17	1399.1	46.2319	18	1322.93	41.2278	19	1249.65	41.4699	20	1151.29	45.4441
21	1105.98	51.7113	22	950.734	70.1569	23	885.166	69.6454	24	848.525	64.971
25	801.278	64.4858	26	757.888	68.8886	27	719.318	66.3373	28	673.999	60.9708
29	629.644	67.5269	30	596.861	67.9861	31	428.12	81.7825			

^{1}H NMR of 12c

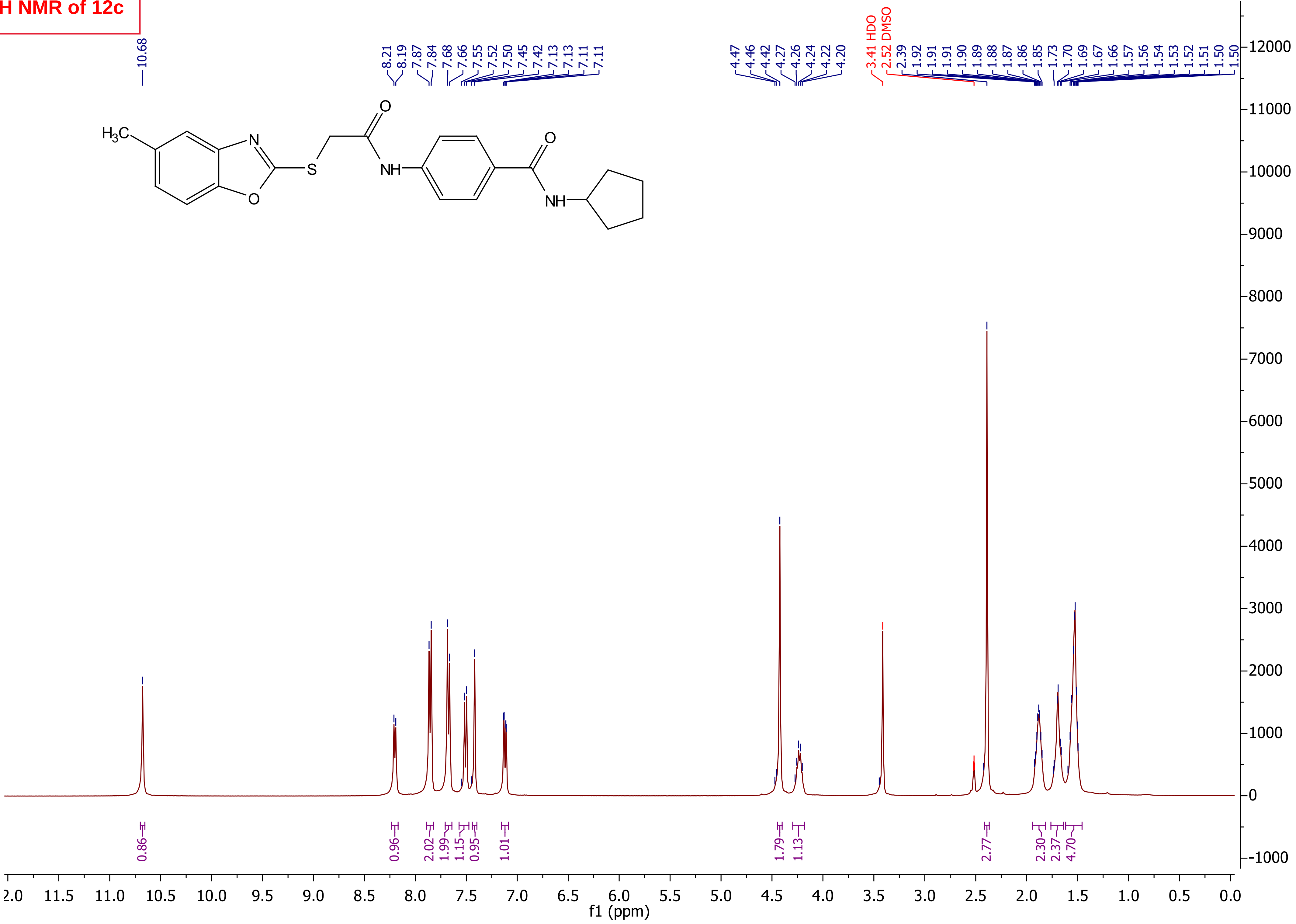

10.68
8.21
8.19
7.87
7.84
7.68
7.66
7.55
7.52
7.50
7.45
7.42
7.13
7.13
7.11
7.11
4.47
4.46
4.42
4.27
4.26
4.24
4.22
4.20
3.41 HDO
2.52 DMSO
2.39
1.92
1.91
1.91
1.90
1.89
1.88
1.87
1.86
1.85
1.73
1.70
1.69
1.67
1.66
1.57
1.56
1.54
1.53
1.52
1.51
1.50
1.50
H3C
N
O
S
O
NH
O
NH
0.86
0.96
2.02
1.99
1.15
0.95
1.01
1.79
1.13
2.77
2.30
2.37
4.70
f1 (ppm)

^{1}H NMR of 12c

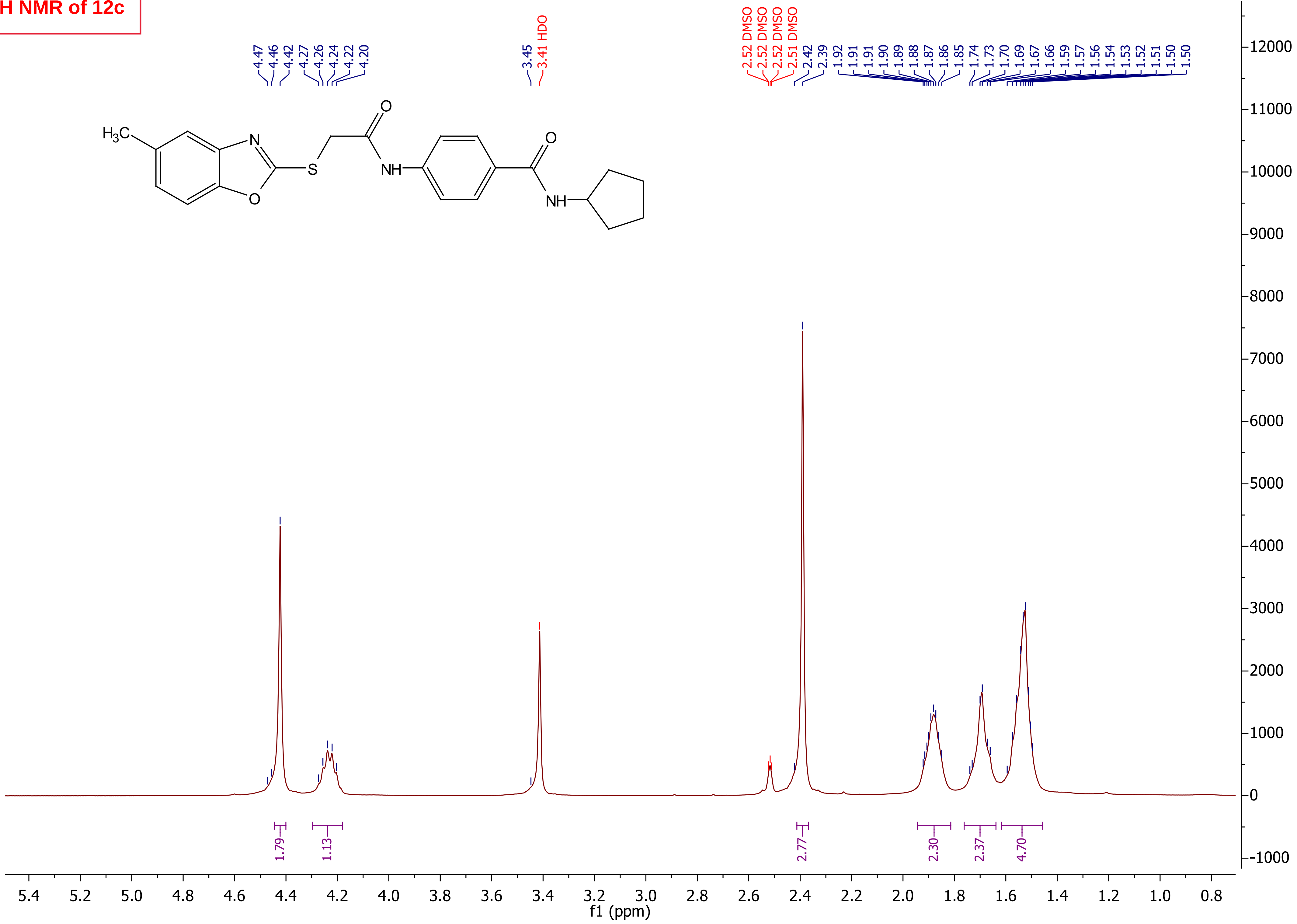

4.47
4.46
4.42
4.27
4.26
4.24
4.22
4.20
3.45
3.41 HDO
2.52 DMSO
2.52 DMSO
2.52 DMSO
2.51 DMSO
2.42
2.39
1.92
1.91
1.91
1.90
1.89
1.88
1.87
1.86
1.85
1.74
1.73
1.70
1.69
1.67
1.66
1.59
1.57
1.56
1.54
1.53
1.52
1.51
1.50
1.50
1.79
1.13
2.77
2.30
2.37
4.70
f1 (ppm)

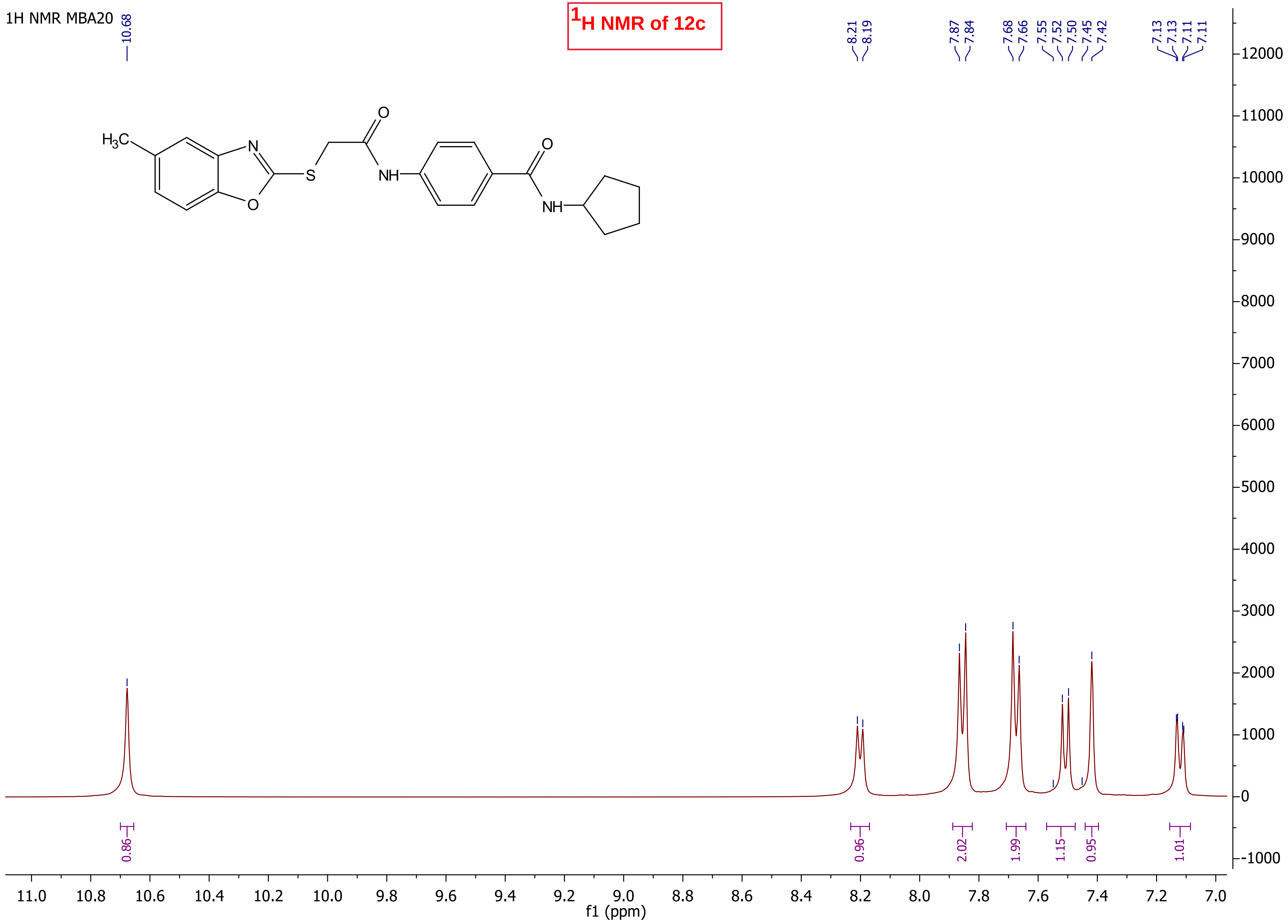
1H NMR MBA20
1H NMR of 12c
10.68
8.21
8.19
7.87
7.84
7.68
7.66
7.55
7.52
7.50
7.45
7.42
7.13
7.13
7.11
7.11
H3C
N
O
S
O
NH
O
NH
0.86
0.96
2.02
1.99
1.15
0.95
1.01
f1 (ppm)

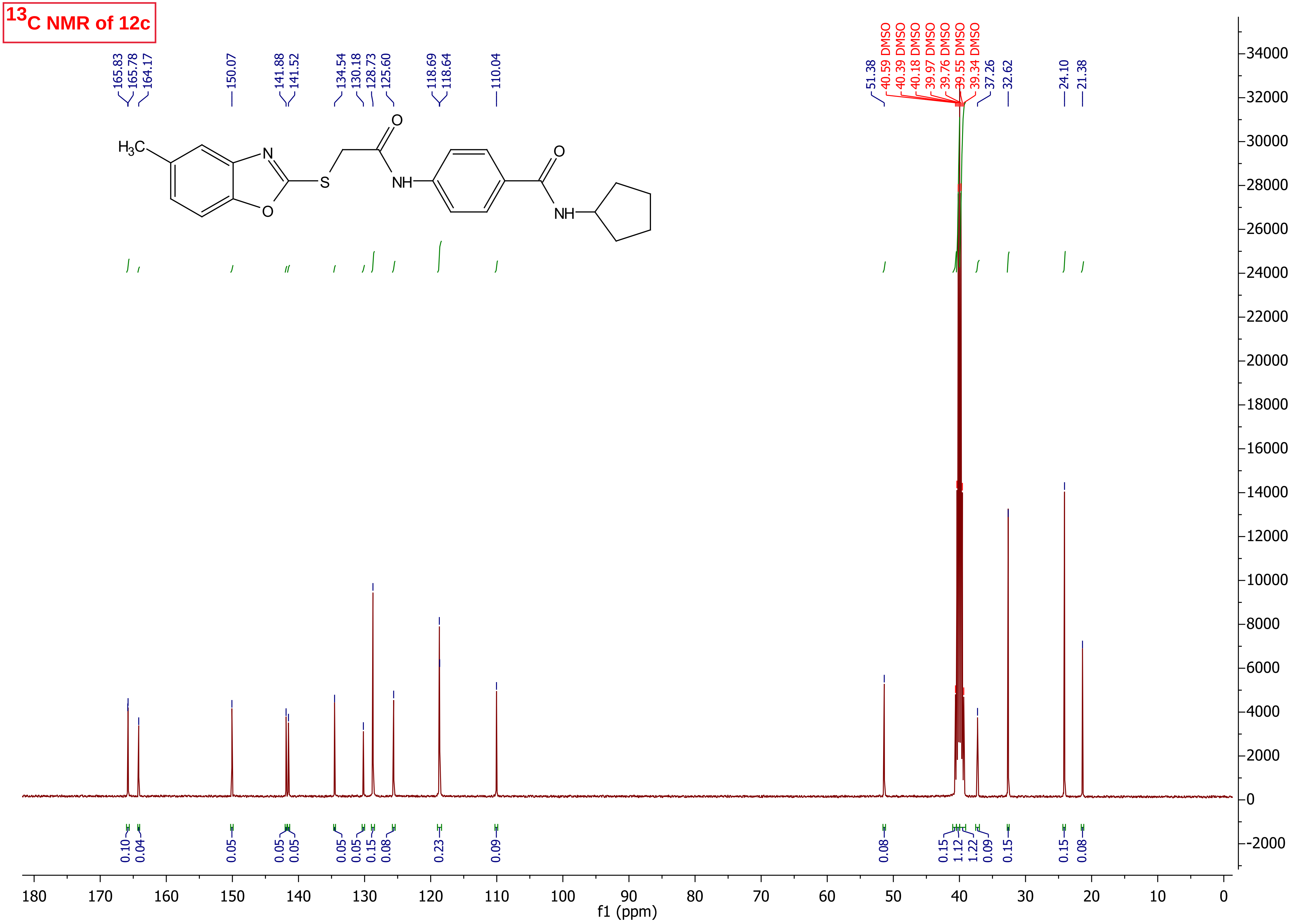

13C NMR of 12c
165.83
165.78
164.17
150.07
141.88
141.52
134.54
130.18
128.73
125.60
118.69
118.64
110.04
51.38
40.59 DMSO
40.39 DMSO
40.18 DMSO
39.97 DMSO
39.76 DMSO
39.55 DMSO
39.34 DMSO
37.26
32.62
24.10
21.38
H3C
N
O
S
O
NH
O
NH
0.10
0.04
0.05
0.05
0.05
0.05
0.05
0.15
0.08
0.23
0.09
0.08
0.15
1.12
1.22
0.09
0.15
0.15
0.08
f1 (ppm)

^{13}C NMR of 12c

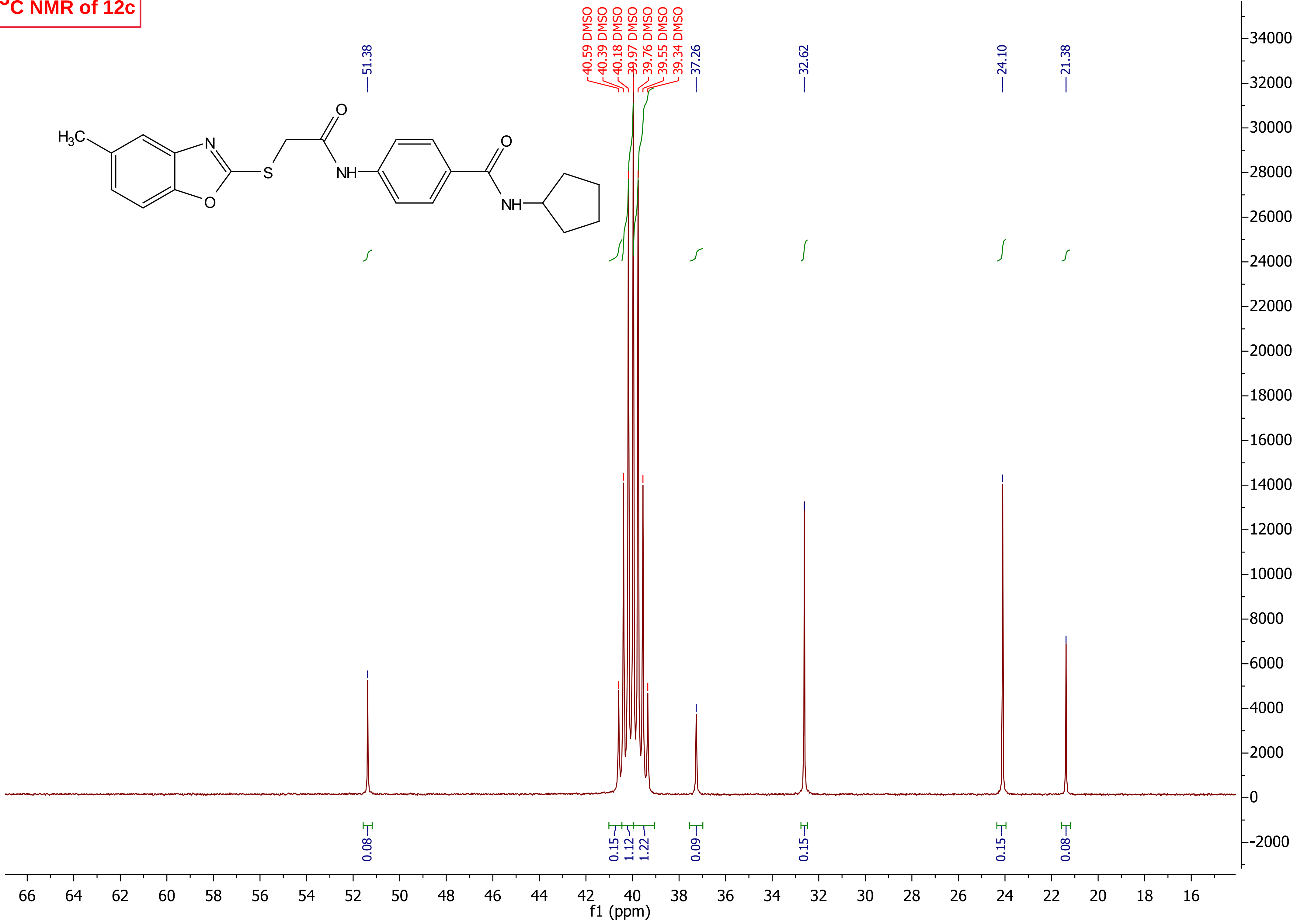

^{13}C NMR of 12c

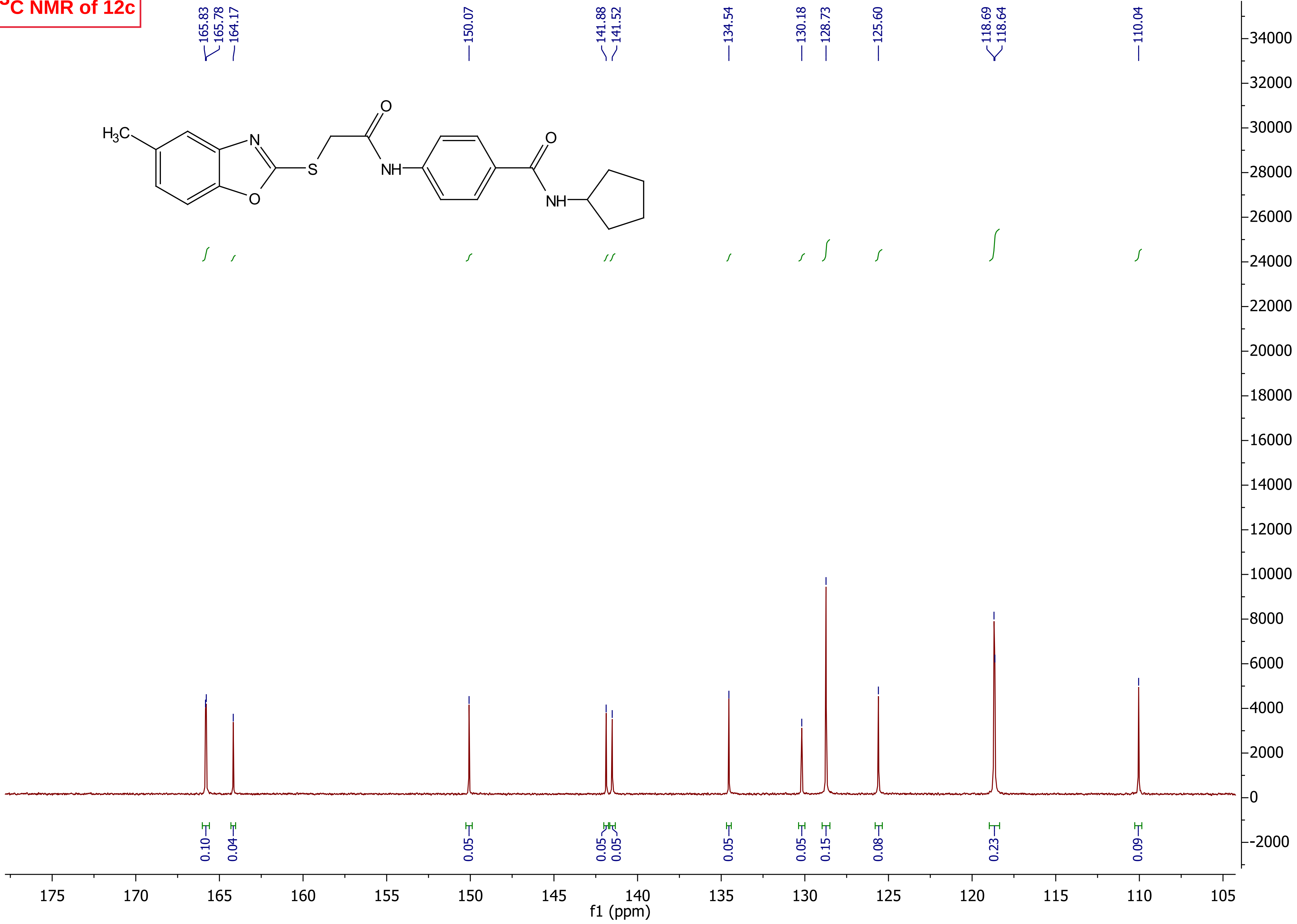

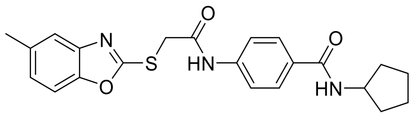

Mass spec. of 12c

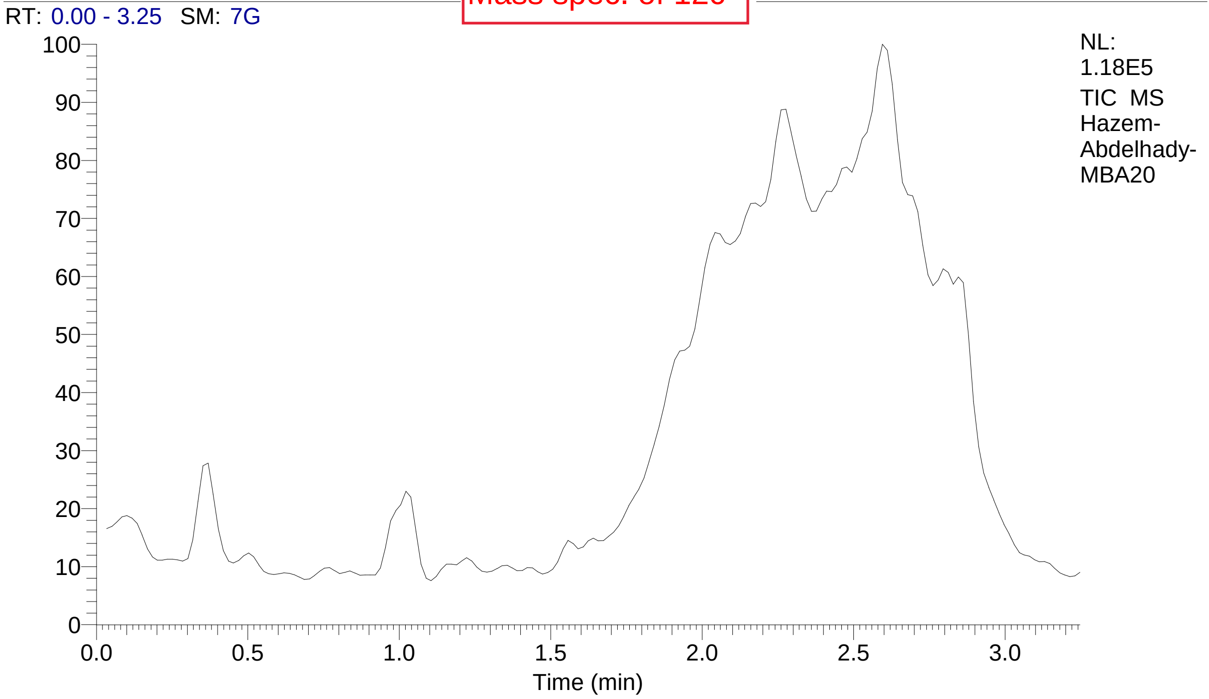

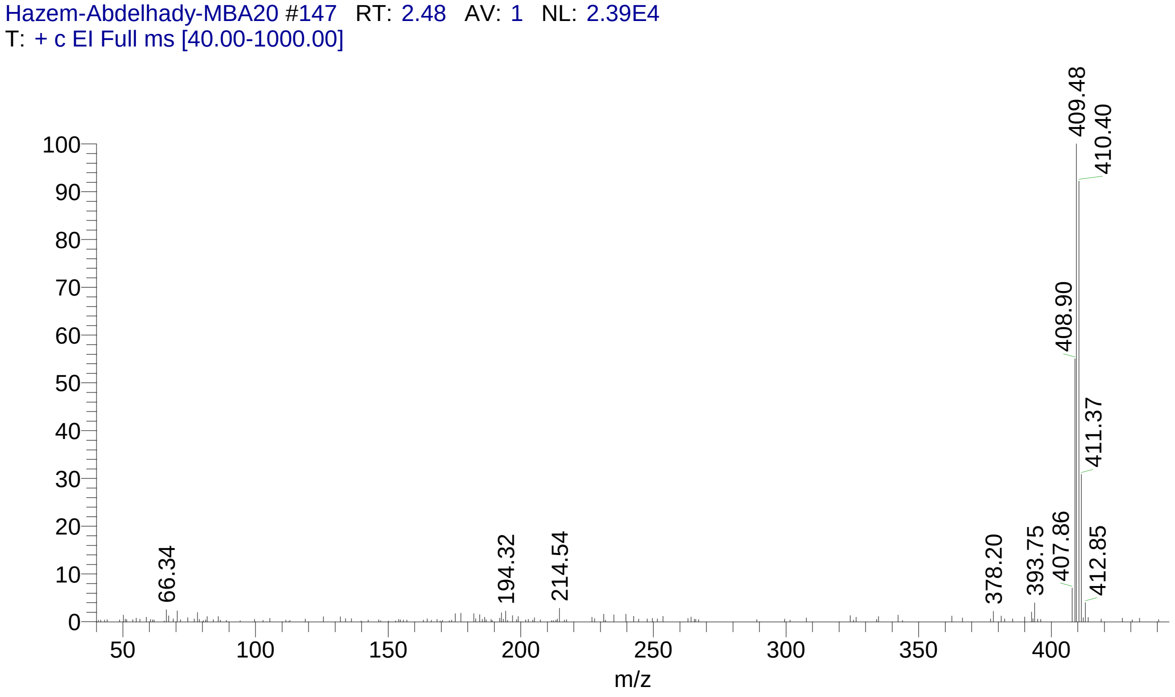

IR Of Comp. 12d

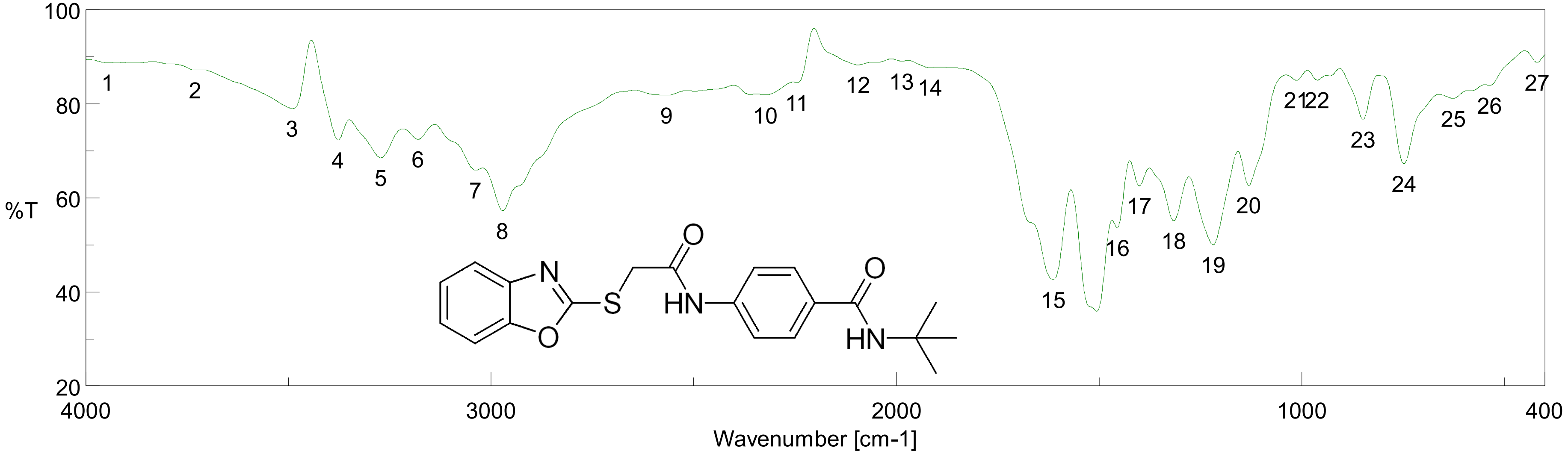

[Result of Peak Picking]

No.	Position	Intensity	No.	Position	Intensity	No.	Position	Intensity	No.	Position	Intensity
1	3945.64	88.7055	2	3730.62	87.1236	3	3490.53	78.9538	4	3377.71	72.2367
5	3272.61	68.484	6	3180.04	72.4134	7	3038.3	65.8248	8	2971.77	57.2148
9	2566.79	81.8072	10	2323.8	81.8976	11	2244.74	84.4556	12	2095.28	88.2464
13	1987.29	89.0325	14	1916.9	87.6549	15	1613.16	42.6012	16	1455.03	53.5738
17	1401.03	62.5312	18	1316.18	55.0999	19	1218.79	49.9762	20	1131.05	62.6221
21	1012.45	84.9699	22	961.341	84.9805	23	848.525	76.6698	24	747.281	67.2273
25	625.788	81.1076	26	536.114	83.8807	27	418.477	88.763			

^{1}H NMR of 12d

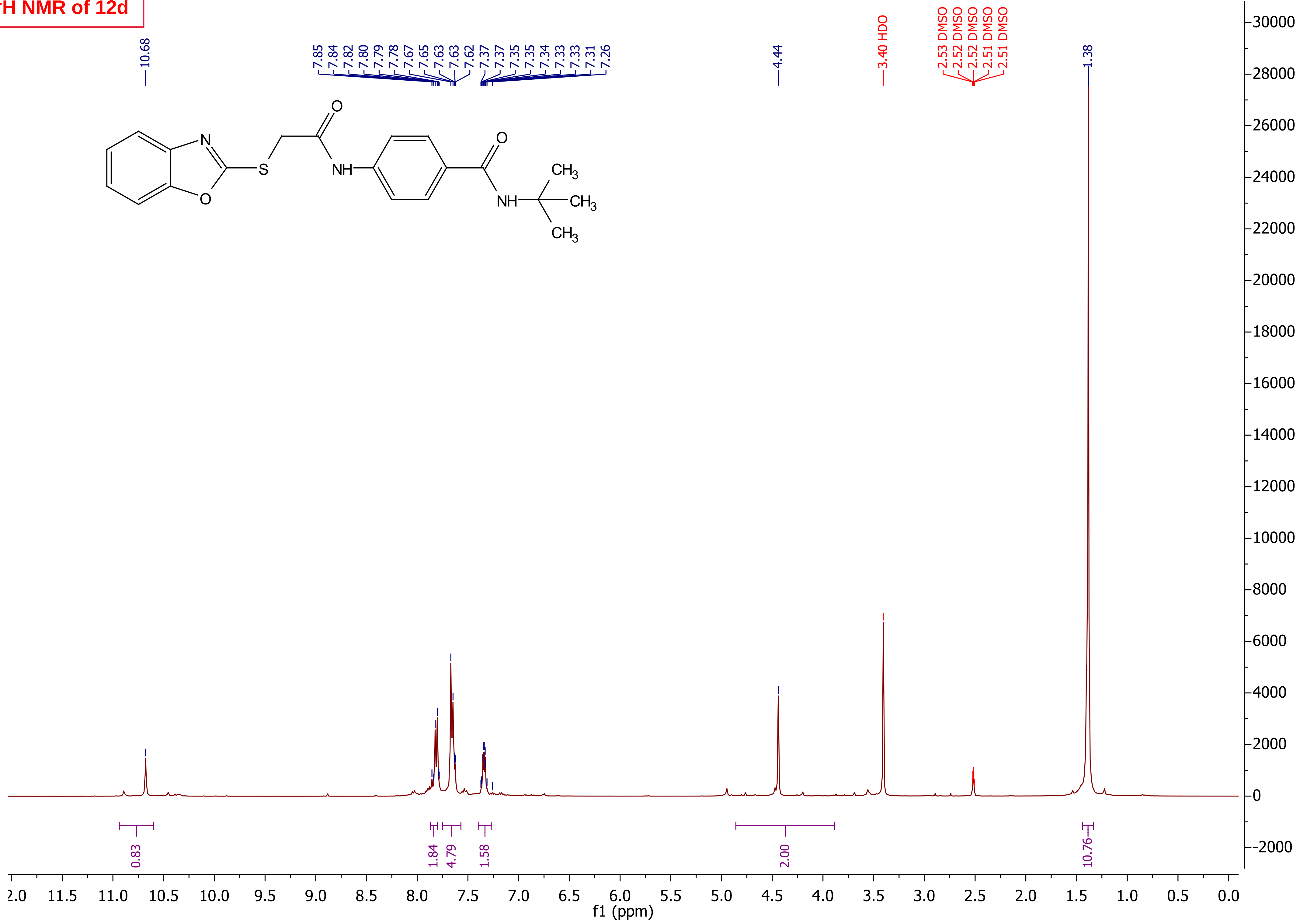

10.68
7.85 7.84 7.82 7.80 7.79 7.78 7.67 7.65 7.63 7.63 7.62 7.37 7.37 7.35 7.35 7.34 7.33 7.33 7.31 7.26
4.44
3.40 HDO
2.53 DMSO 2.52 DMSO 2.52 DMSO 2.51 DMSO 2.51 DMSO
1.38
O
N
S
NH
O
O
NH
CH3
CH3
CH3
0.83
1.84
4.79
1.58
2.00
10.76
f1 (ppm)

^{1}H NMR of 12d

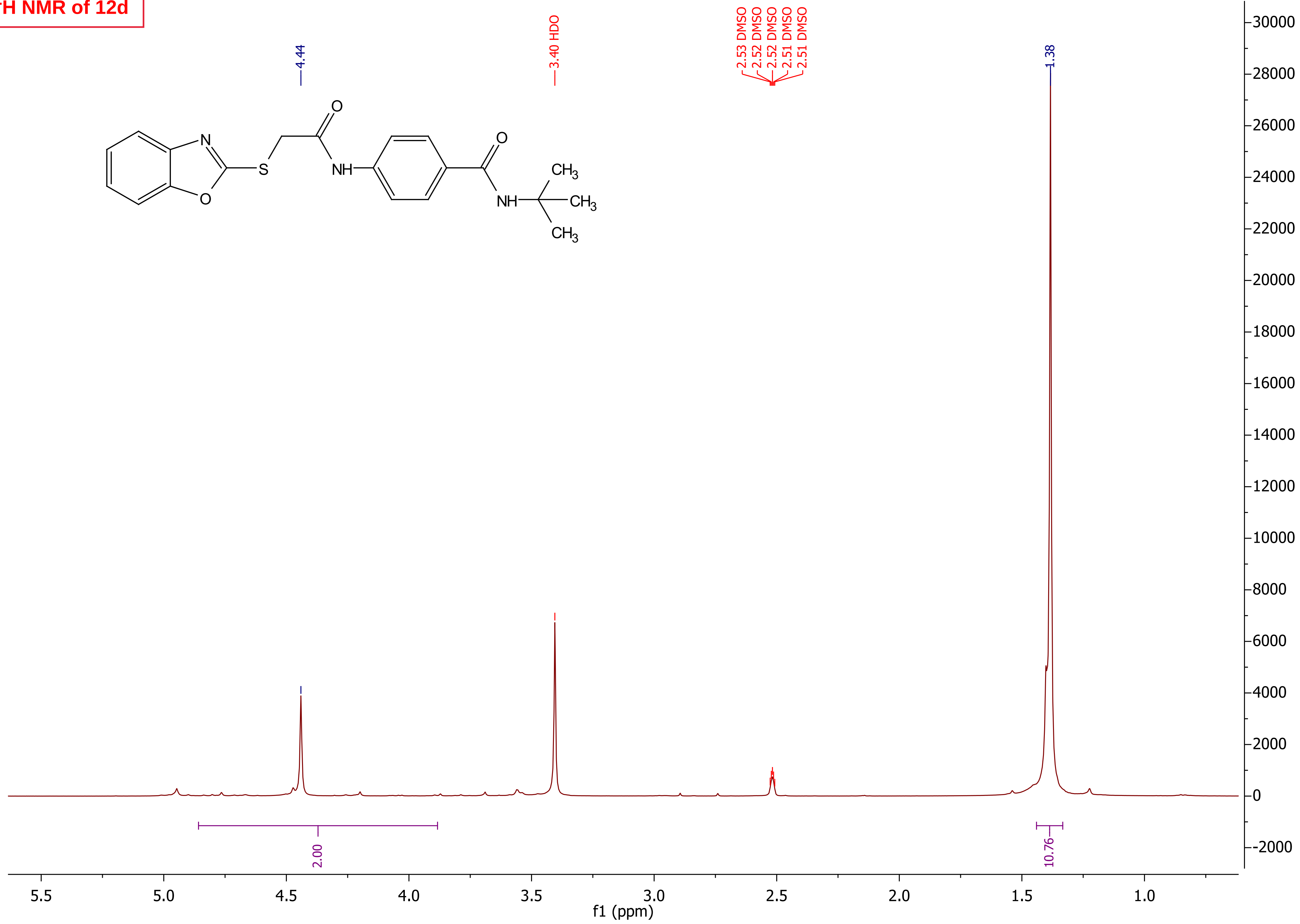

4.44
3.40 HDO
2.53 DMSO
2.52 DMSO
2.52 DMSO
2.51 DMSO
2.51 DMSO
1.38
O
N
S
NH
O
O
NH
CH3
CH3
CH3
2.00
10.76
f1 (ppm)

1H NMR PBA35

^{1}H NMR of 12d

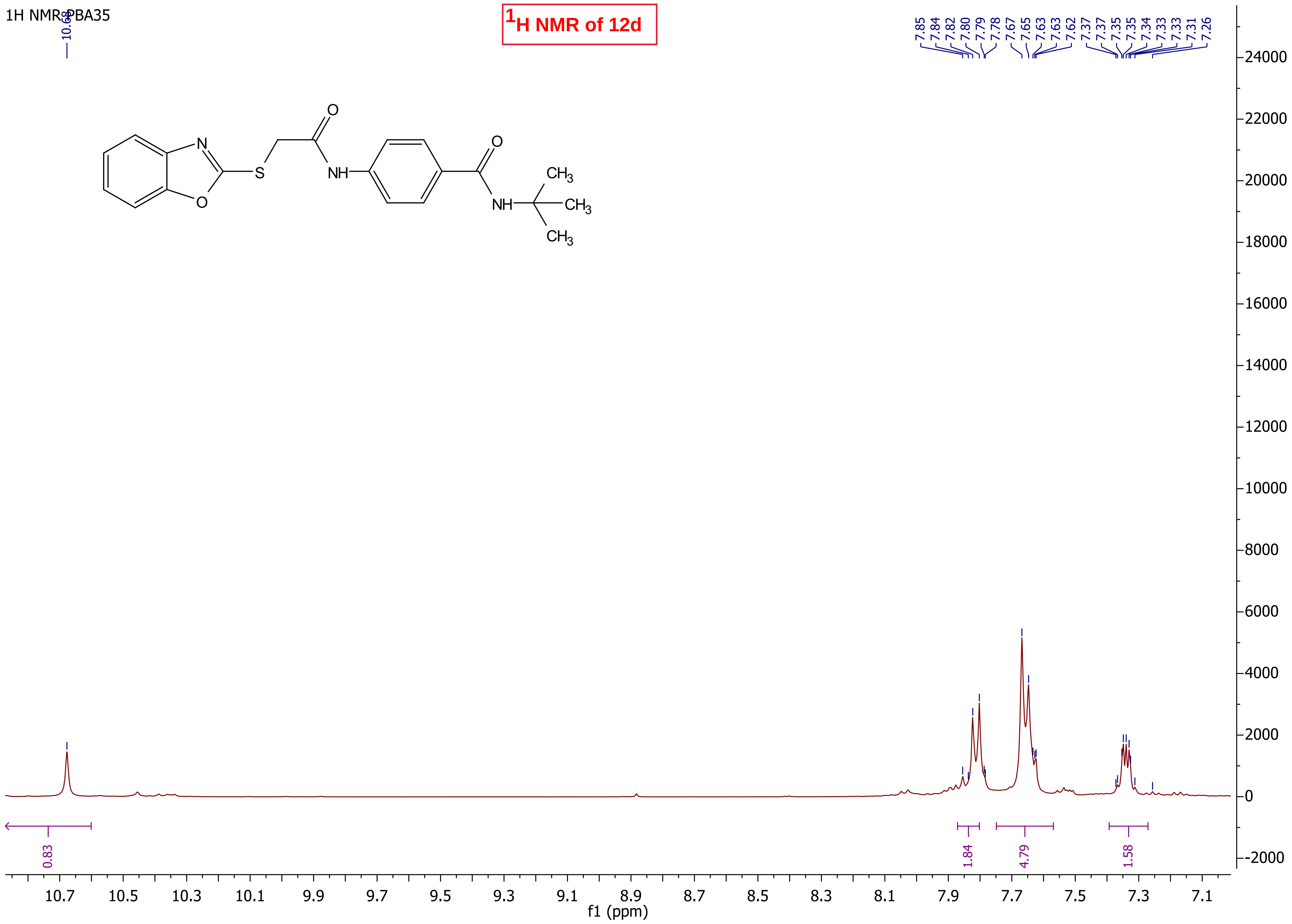

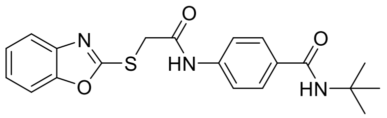

Mass spec. of 12d

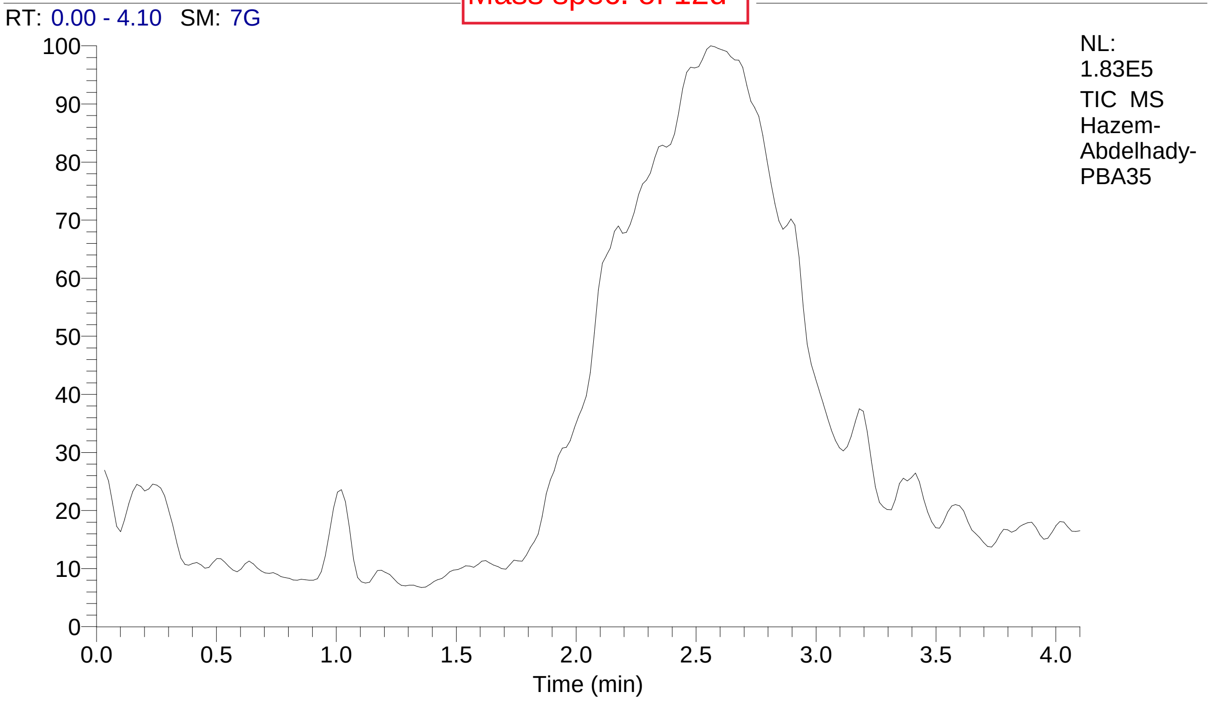

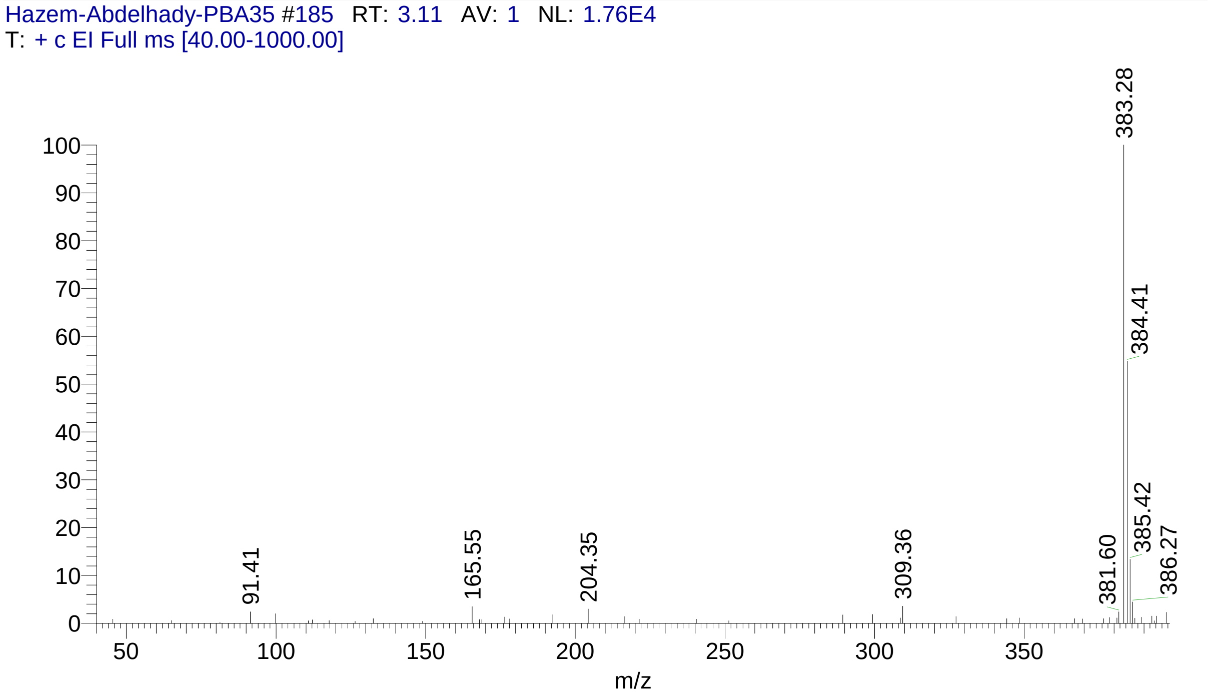

IR Of Comp. 12e

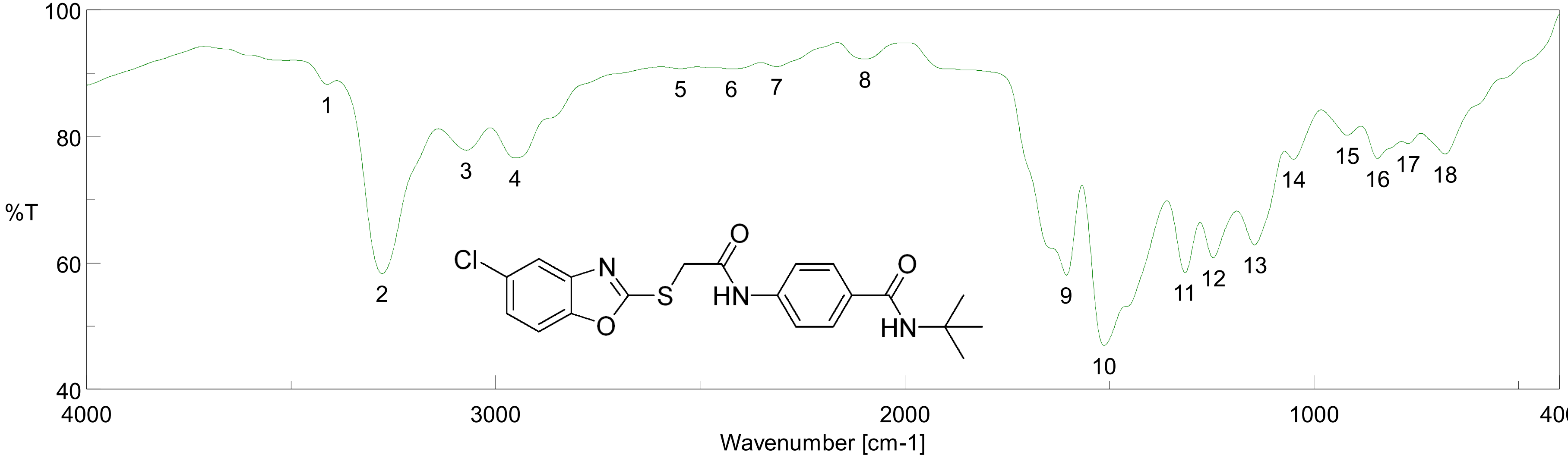

[Result of Peak Picking]

No.	Position	Intensity	No.	Position	Intensity	No.	Position	Intensity	No.	Position	Intensity
1	3412.42	88.1914	2	3277.43	58.2797	3	3072.05	77.7668	4	2951.52	76.5641
5	2548.47	90.6699	6	2423.12	90.6144	7	2313.2	91.0073	8	2097.21	92.1935
9	1604.48	58.0365	10	1512.88	46.9154	11	1315.21	58.44	12	1245.79	60.7719
13	1144.55	62.8167	14	1049.09	76.3545	15	917.95	80.1353	16	844.669	76.5009
17	769.458	78.8302	18	678.82	77.1604						

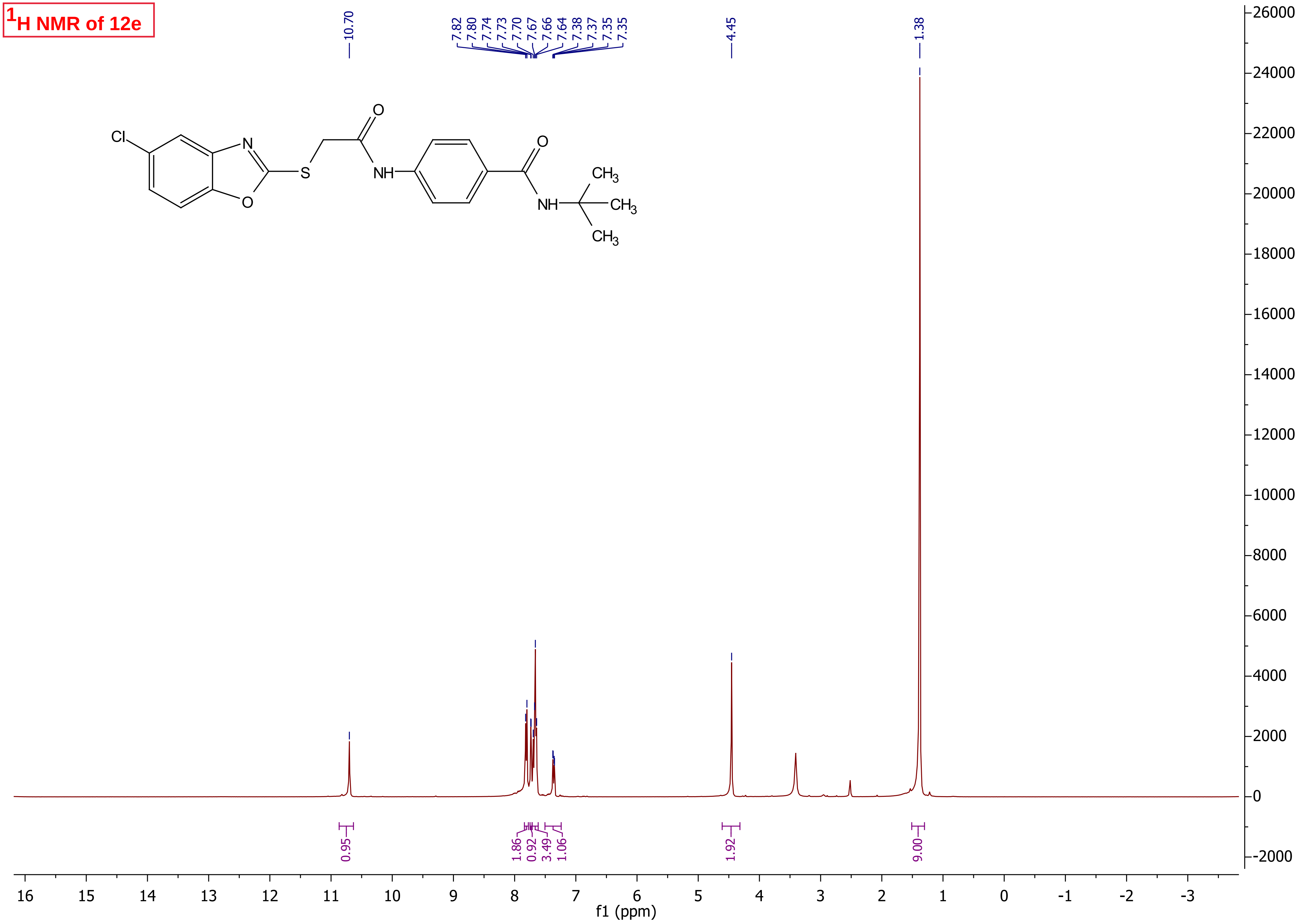

1H NMR of 12e
10.70
7.82
7.80
7.74
7.73
7.70
7.67
7.66
7.64
7.38
7.37
7.35
7.35
4.45
1.38
0.95
1.86
0.92
3.49
1.06
1.92
9.00
f1 (ppm)

1H NMR of 12e

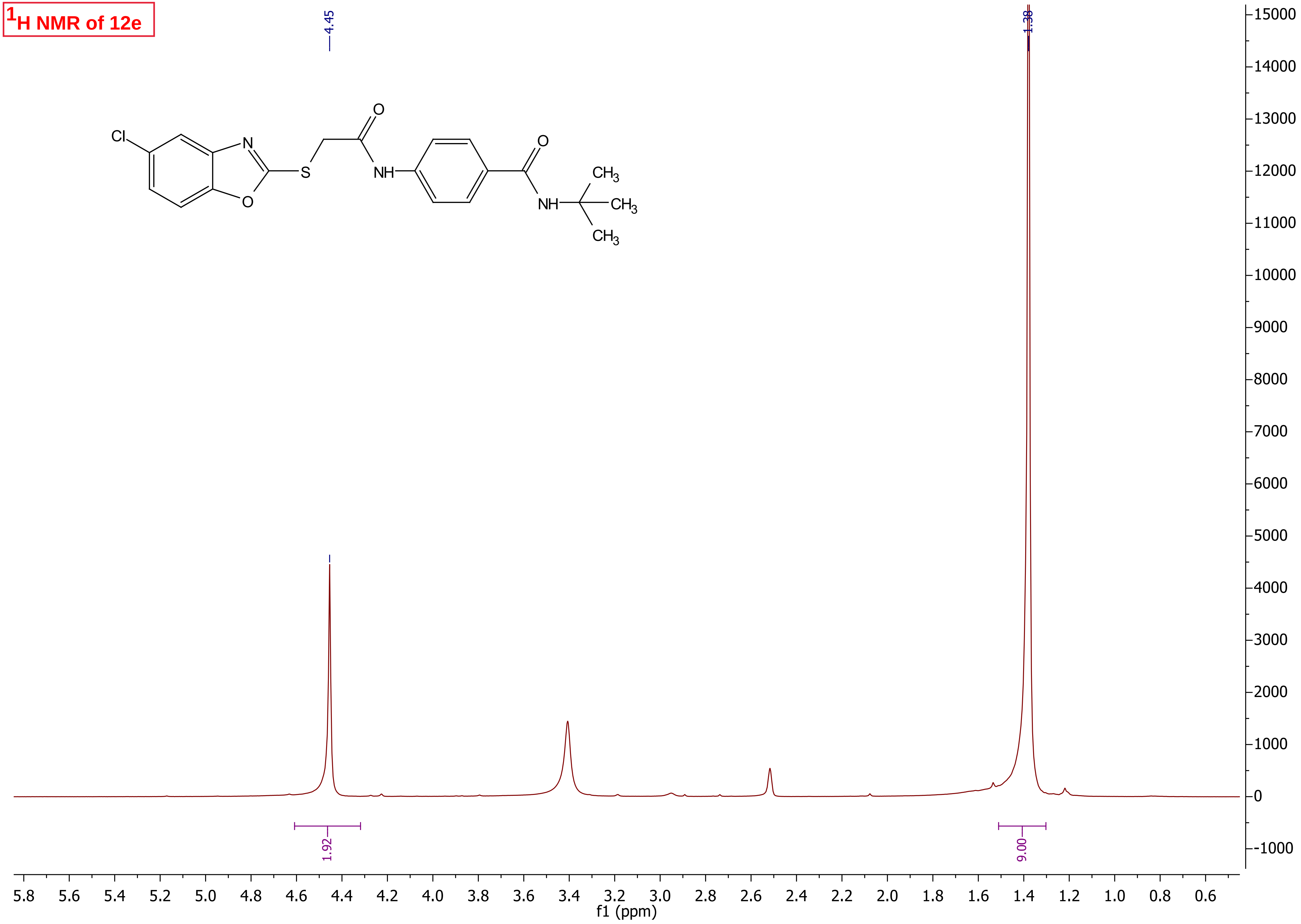
4.45
1.38
Cl
N
O
S
NH
O
NH
CH3
CH3
CH3
1.92
9.00
f1 (ppm)
5.8
5.6
5.4
5.2
5.0
4.8
4.6
4.4
4.2
4.0
3.8
3.6
3.4
3.2
3.0
2.8
2.6
2.4
2.2
2.0
1.8
1.6
1.4
1.2
1.0
0.8
0.6
15000
14000
13000
12000
11000
10000
9000
8000
7000
6000
5000
4000
3000
2000
1000
0
-1000

1H NMR of 12e

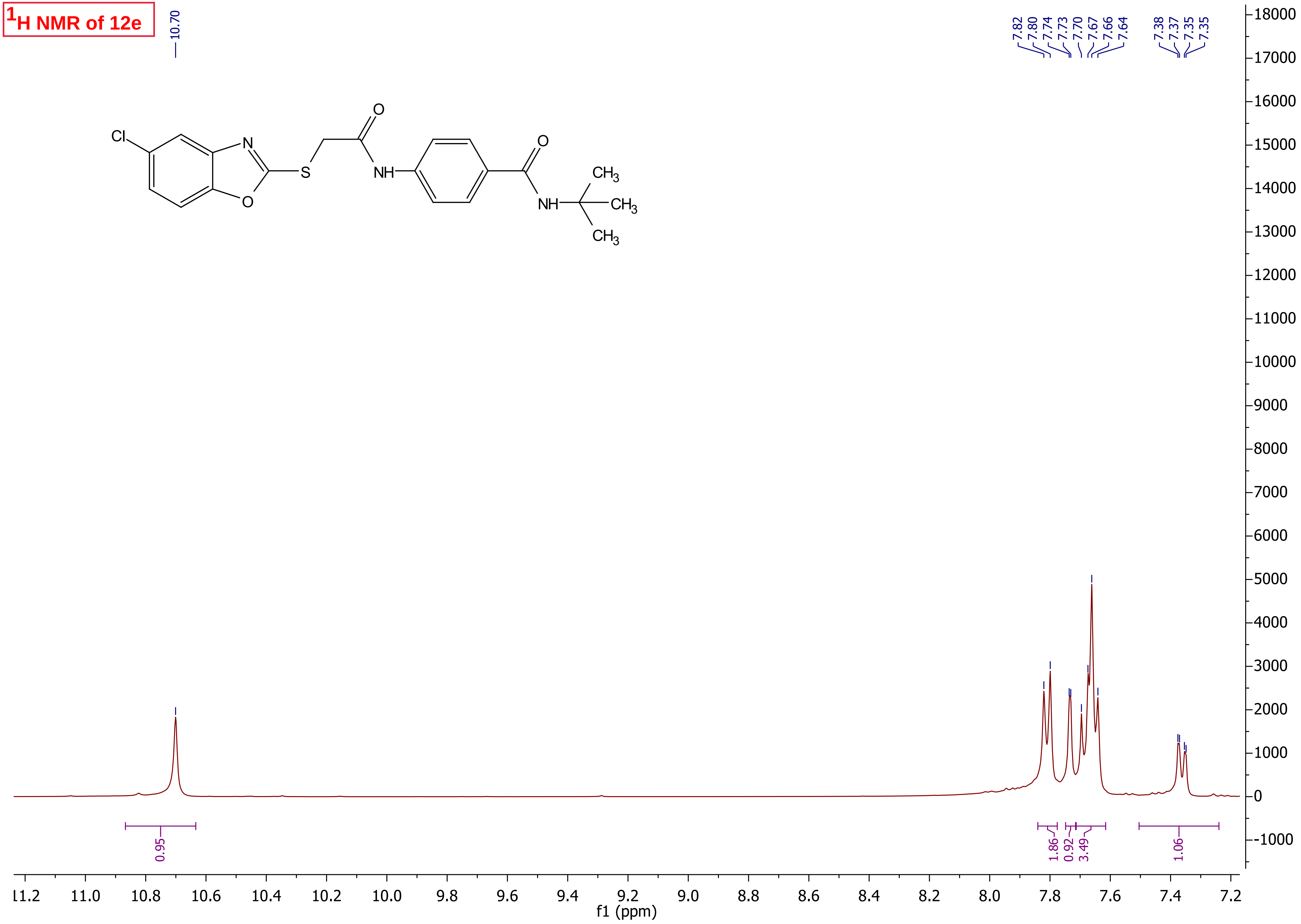

10.70
7.82
7.80
7.74
7.73
7.70
7.67
7.66
7.64
7.38
7.37
7.35
7.35
Cl
N
O
S
NH
O
O
NH
CH3
CH3
CH3
0.95
1.86
0.92
3.49
1.06
11.2
11.0
10.8
10.6
10.4
10.2
10.0
9.8
9.6
9.4
9.2
9.0
8.8
8.6
8.4
8.2
8.0
7.8
7.6
7.4
7.2
f1 (ppm)
18000
17000
16000
15000
14000
13000
12000
11000
10000
9000
8000
7000
6000
5000
4000
3000
2000
1000
0
-1000

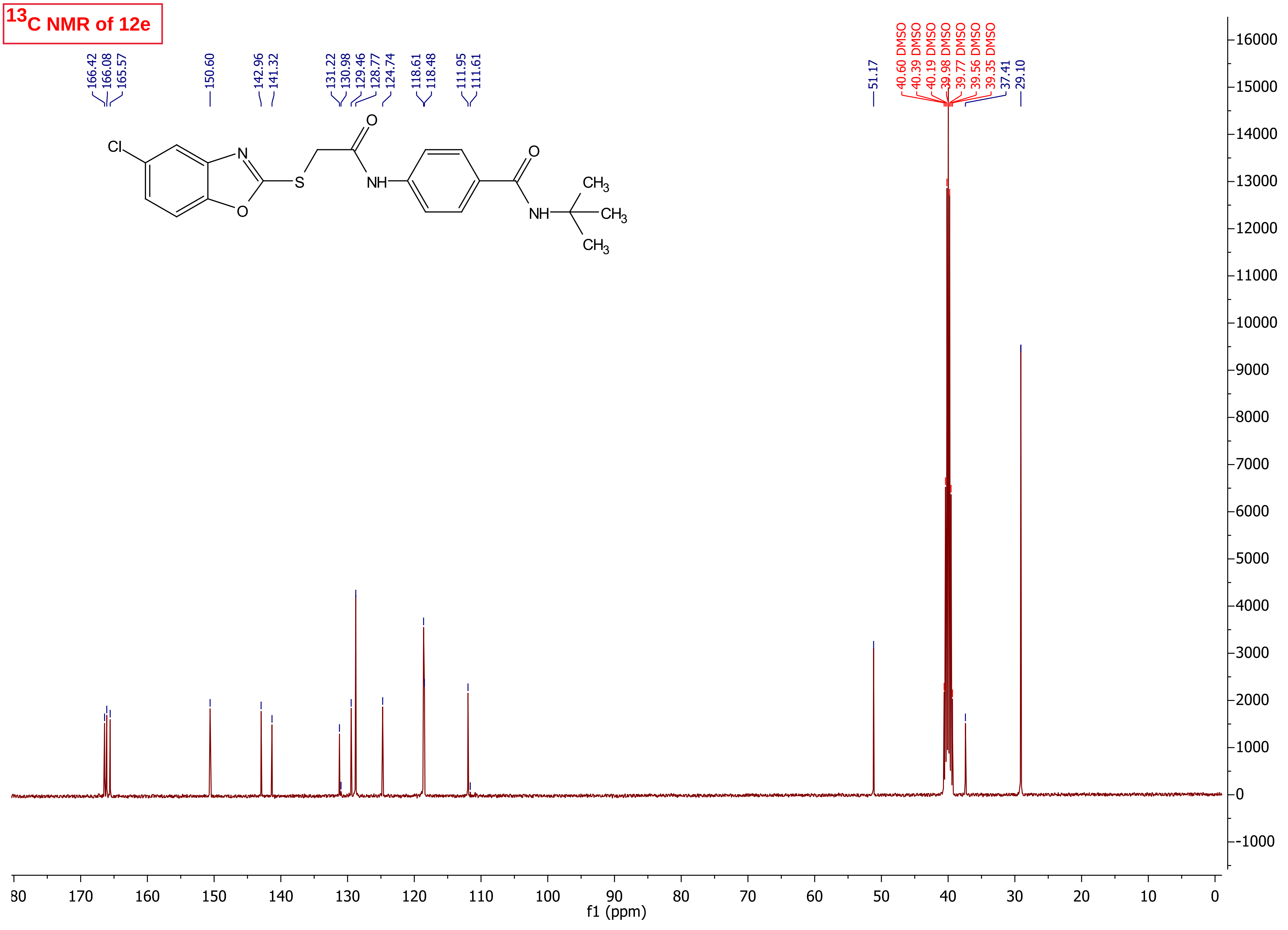
13C NMR of 12e
166.42
166.08
165.57
150.60
142.96
141.32
131.22
130.98
129.46
128.77
124.74
118.61
118.48
111.95
111.61
51.17
40.60 DMSO
40.39 DMSO
40.19 DMSO
39.98 DMSO
39.77 DMSO
39.56 DMSO
39.35 DMSO
37.41
29.10
Cl
N
O
S
O
NH
O
NH
CH3
CH3
CH3
f1 (ppm)

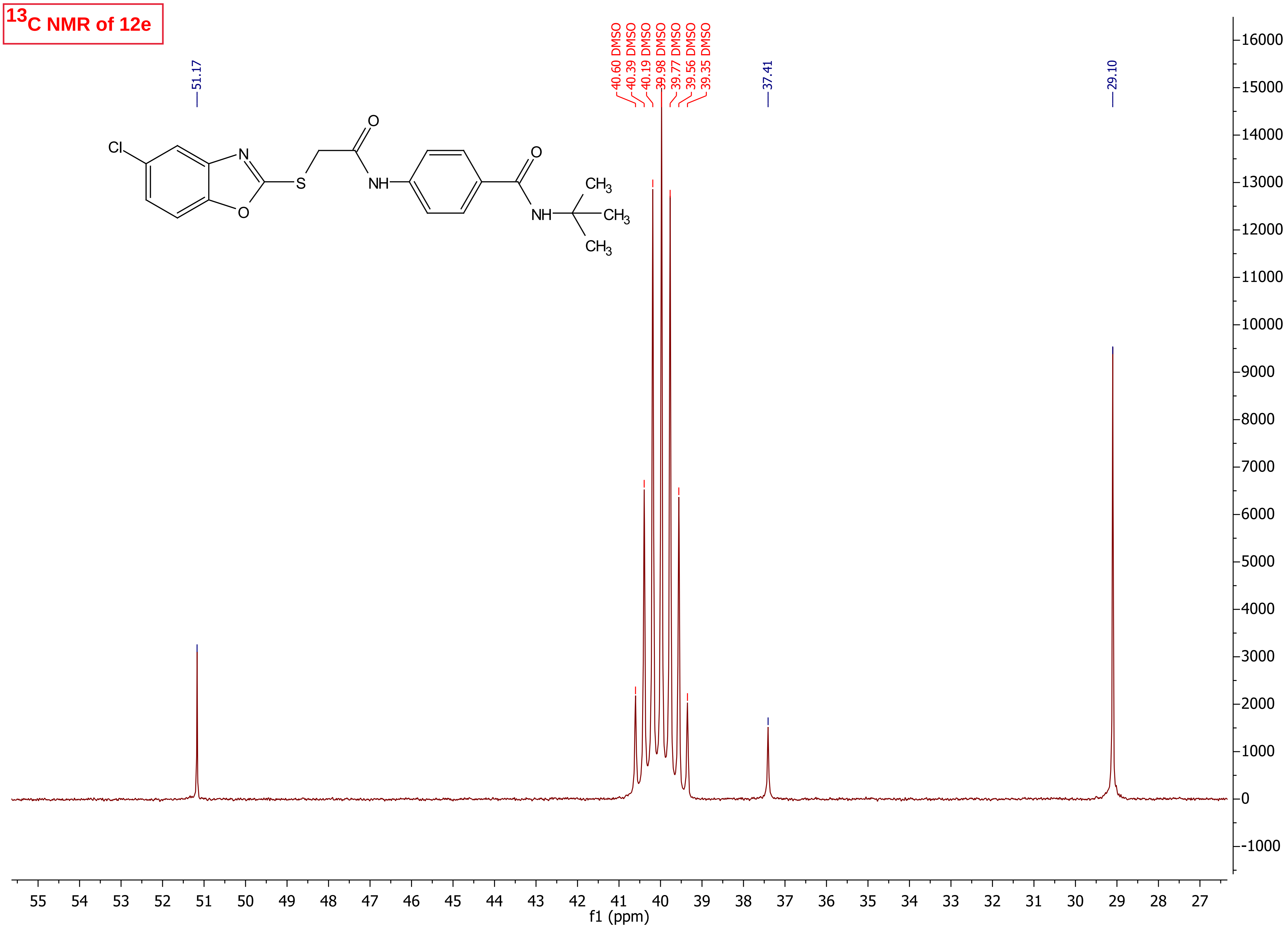

13C NMR of 12e
51.17
40.60 DMSO
40.39 DMSO
40.19 DMSO
39.98 DMSO
39.77 DMSO
39.56 DMSO
39.35 DMSO
37.41
29.10
Cl
N
O
S
O
NH
O
NH
CH3
CH3
CH3
f1 (ppm)

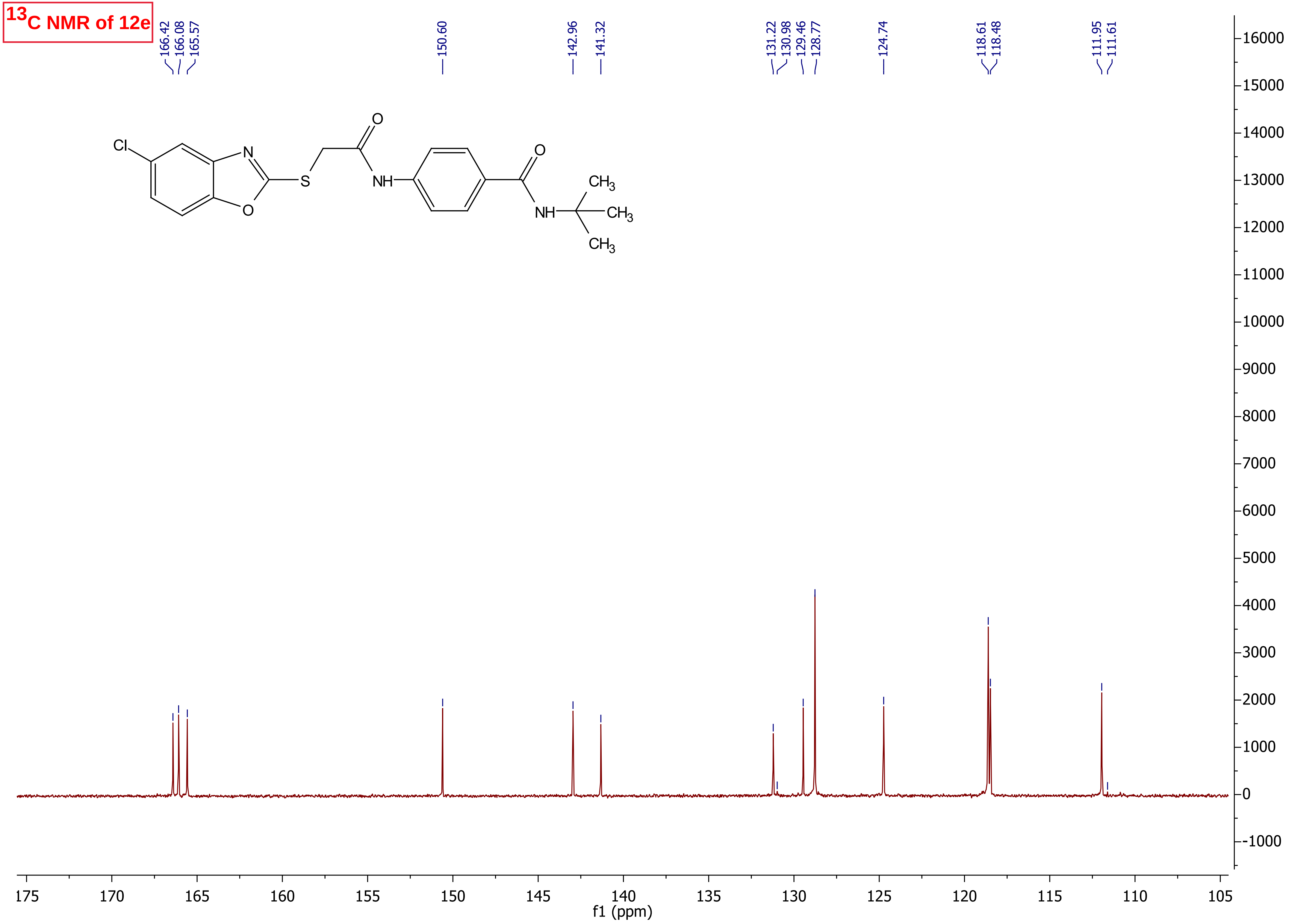

13C NMR of 12e
166.42
166.08
165.57
150.60
142.96
141.32
131.22
130.98
129.46
128.77
124.74
118.61
118.48
111.95
111.61
Cl
N
O
S
O
NH
O
NH
CH3
CH3
CH3
16000
15000
14000
13000
12000
11000
10000
9000
8000
7000
6000
5000
4000
3000
2000
1000
0
-1000
175
170
165
160
155
150
145
140
135
130
125
120
115
110
105
f1 (ppm)

IR Of Comp. 12f

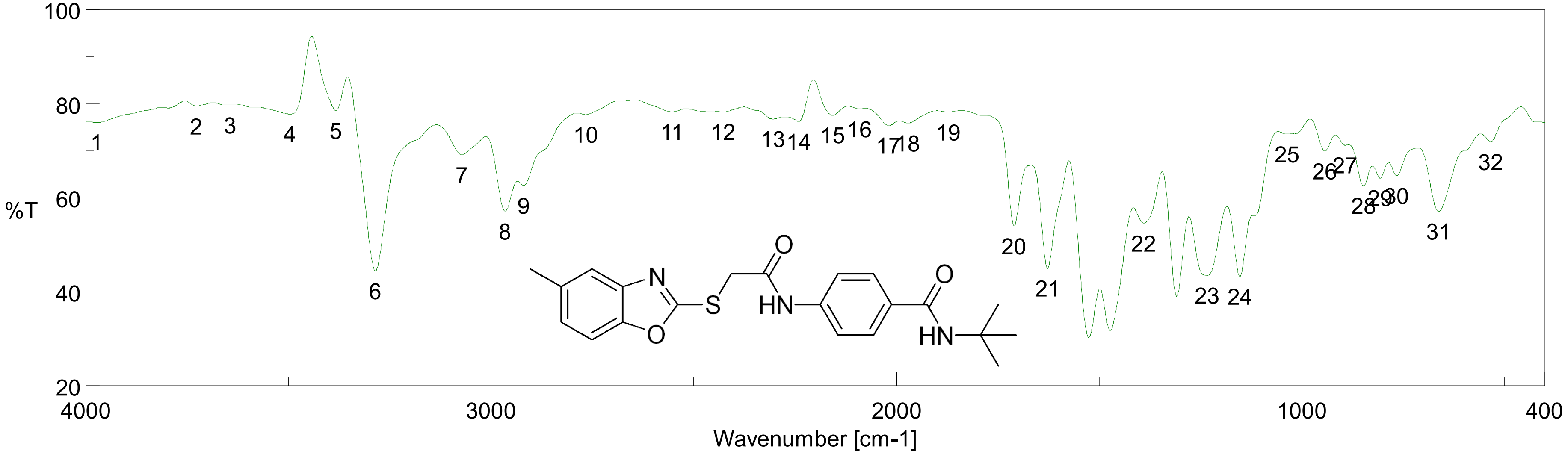

[Result of Peak Picking]

No.	Position	Intensity	No.	Position	Intensity	No.	Position	Intensity	No.	Position	Intensity
1	3973.61	76.0057	2	3726.76	79.4803	3	3643.84	79.6679	4	3496.31	77.7767
5	3383.5	78.5136	6	3286.11	44.4572	7	3072.05	69.1118	8	2965.98	57.1068
9	2919.7	62.6076	10	2766.39	77.6778	11	2553.29	78.2467	12	2427.94	78.2195
13	2304.52	76.727	14	2241.84	76.2221	15	2157.95	77.5339	16	2091.42	78.9065
17	2019.1	75.3331	18	1971.86	75.8492	19	1872.54	78.199	20	1709.59	53.994
21	1626.66	44.9623	22	1389.46	54.5873	23	1233.25	43.4485	24	1152.26	43.242
25	1036.55	73.5466	26	943.02	69.9137	27	892.88	71.1529	28	846.597	62.5262
29	806.099	64.142	30	765.601	64.6793	31	661.464	57.0638	32	533.221	71.9242

^{1}H NMR of 12f

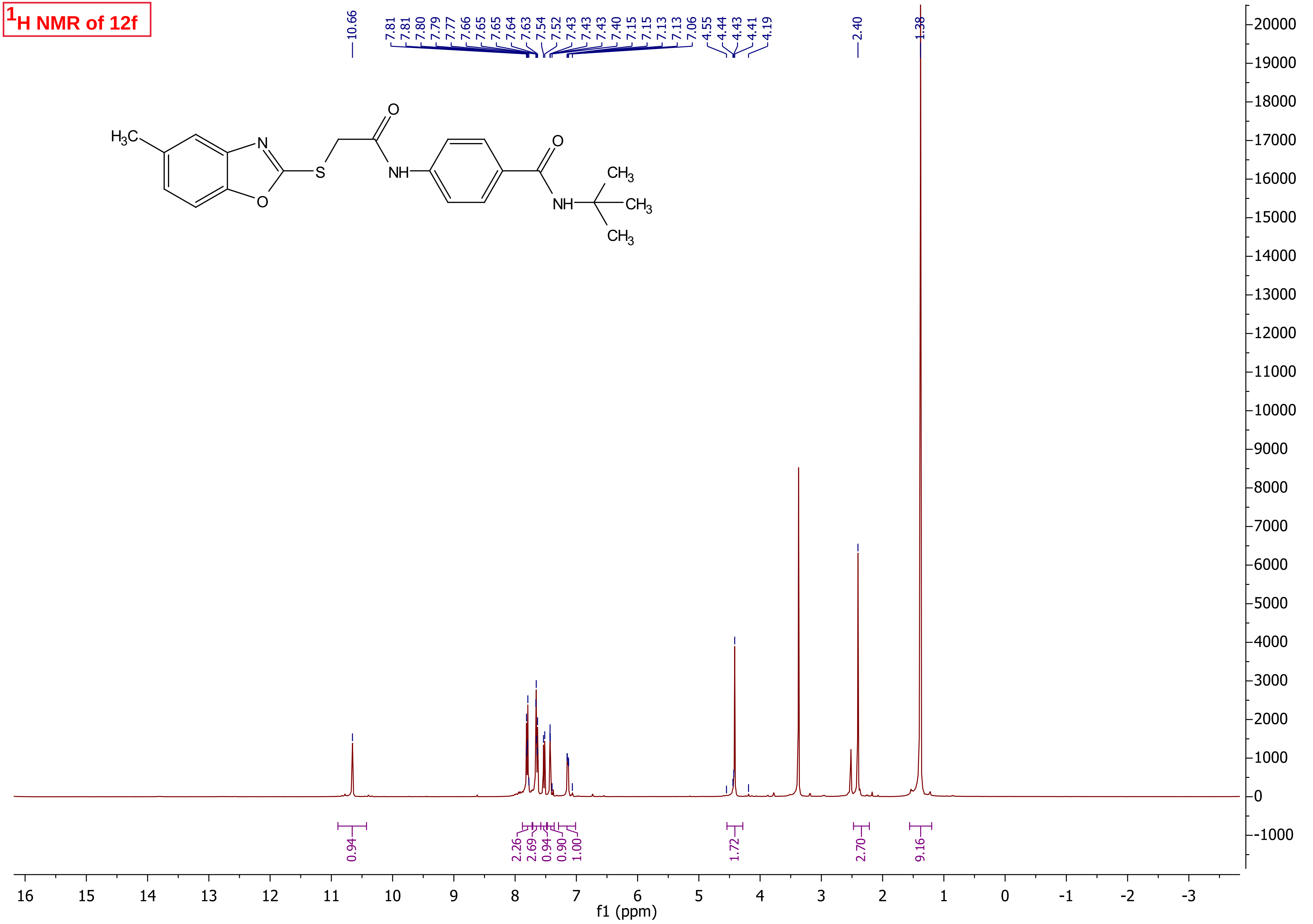
10.66
7.81
7.81
7.80
7.79
7.77
7.66
7.65
7.65
7.64
7.63
7.54
7.52
7.43
7.43
7.43
7.40
7.15
7.15
7.13
7.13
7.06
4.55
4.44
4.43
4.41
4.19
2.40
1.38
H3C
N
O
S
O
NH
O
NH
CH3
CH3
CH3
0.94
2.26
2.69
0.94
0.90
1.00
1.72
2.70
9.16
f1 (ppm)

^{1}H NMR of 12f

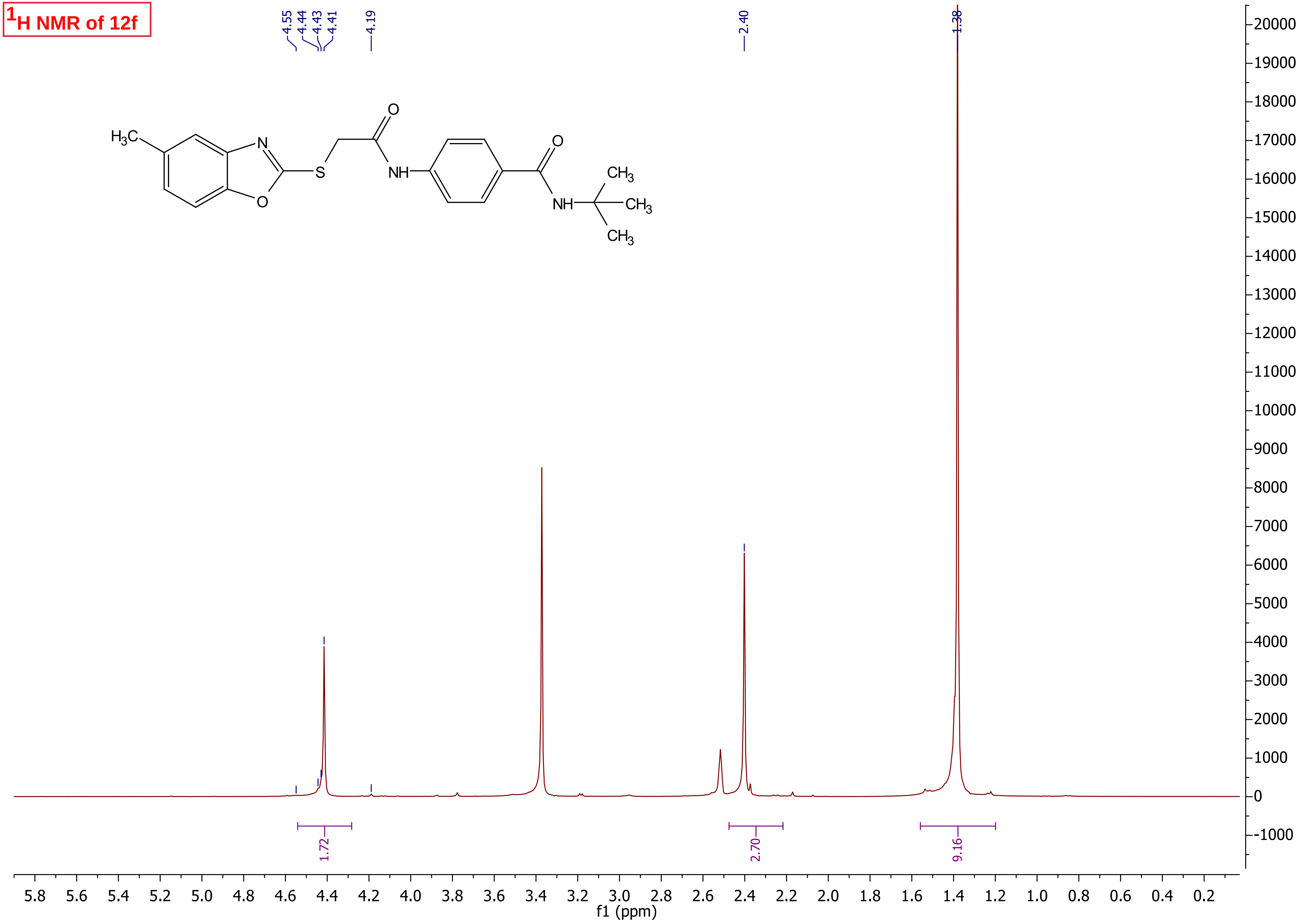

4.55
4.44
4.43
4.41
4.19
2.40
1.38
H3C
N
O
S
O
NH
O
NH
CH3
CH3
CH3
1.72
2.70
9.16
f1 (ppm)

^{1}H NMR of 12f

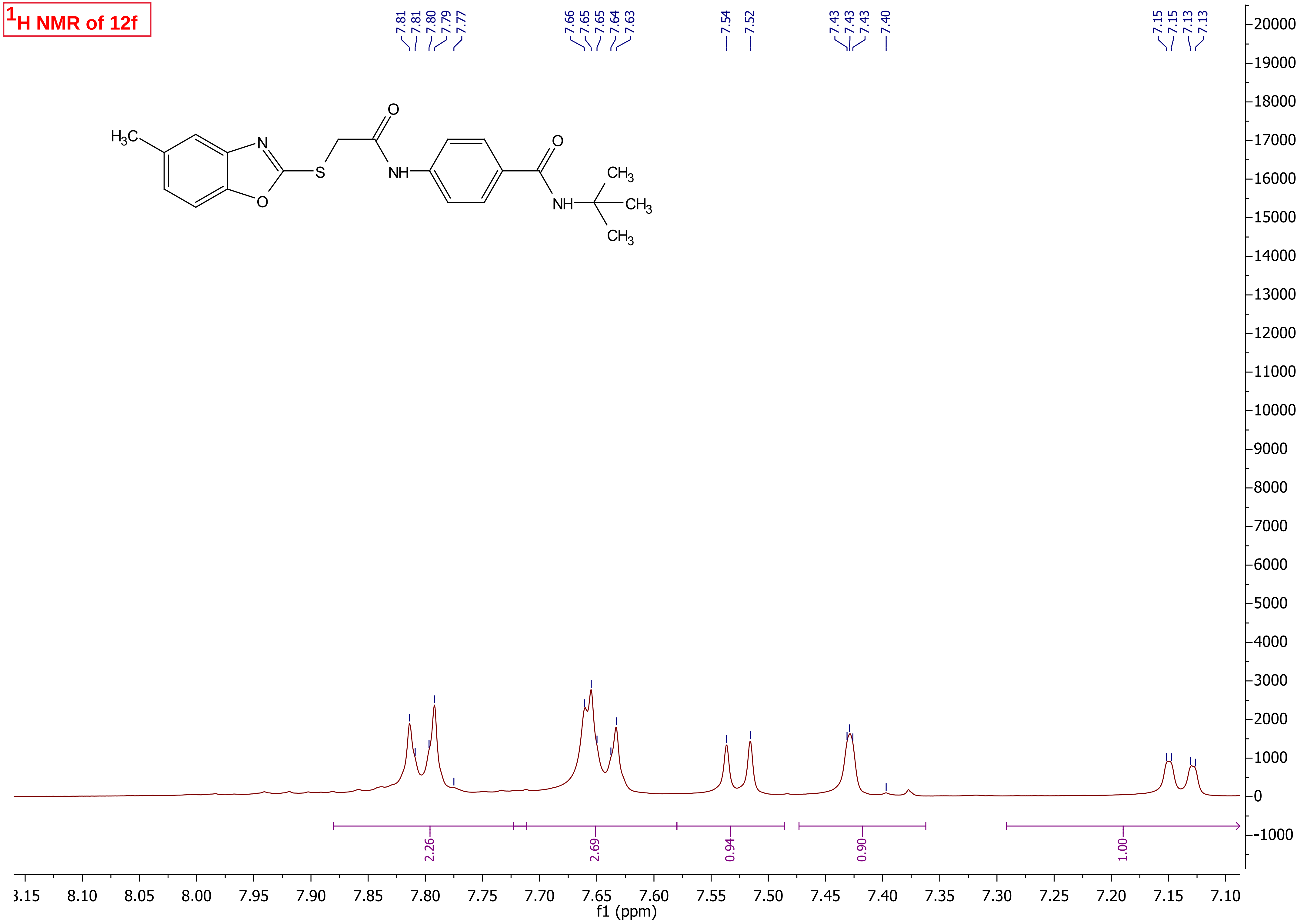
7.81
7.81
7.80
7.79
7.77
7.66
7.65
7.65
7.64
7.63
7.54
7.52
7.43
7.43
7.43
7.40
7.15
7.15
7.13
7.13
H3C
N
O
S
O
NH
O
NH
CH3
CH3
CH3
2.26
2.69
0.94
0.90
1.00
f1 (ppm)

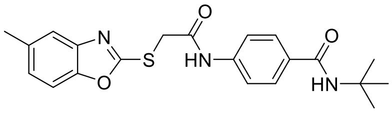

Mass spec. of 12f

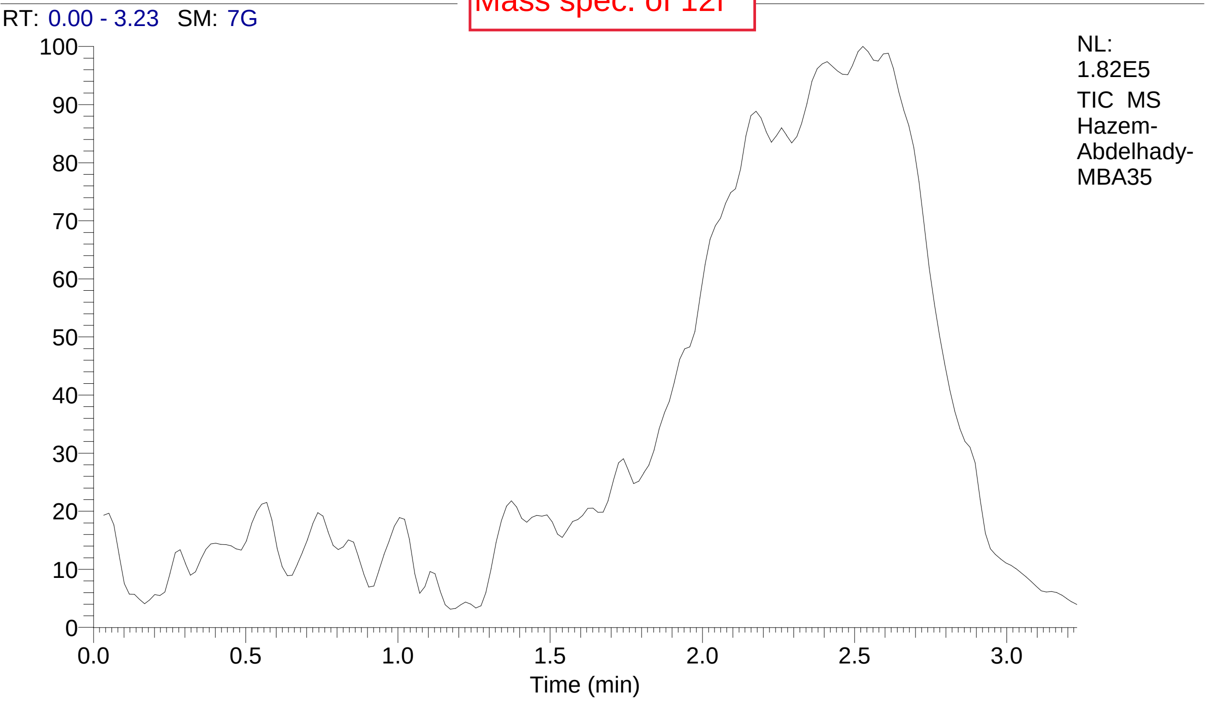

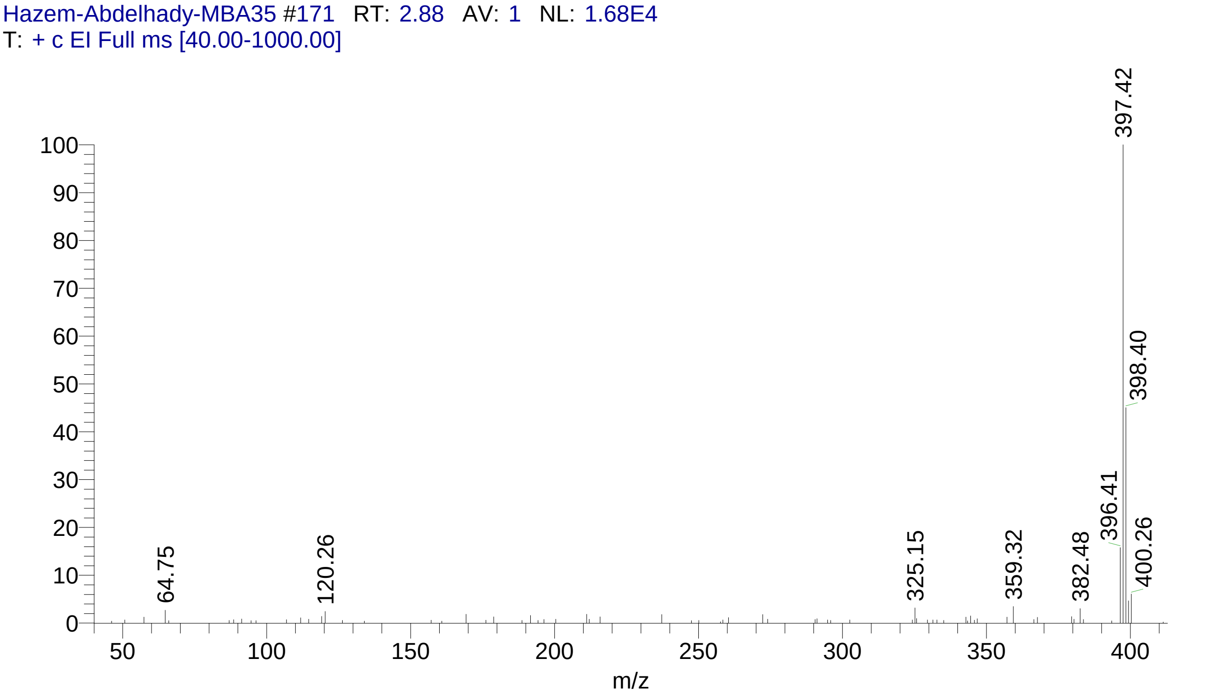

IR Of Comp. 12g

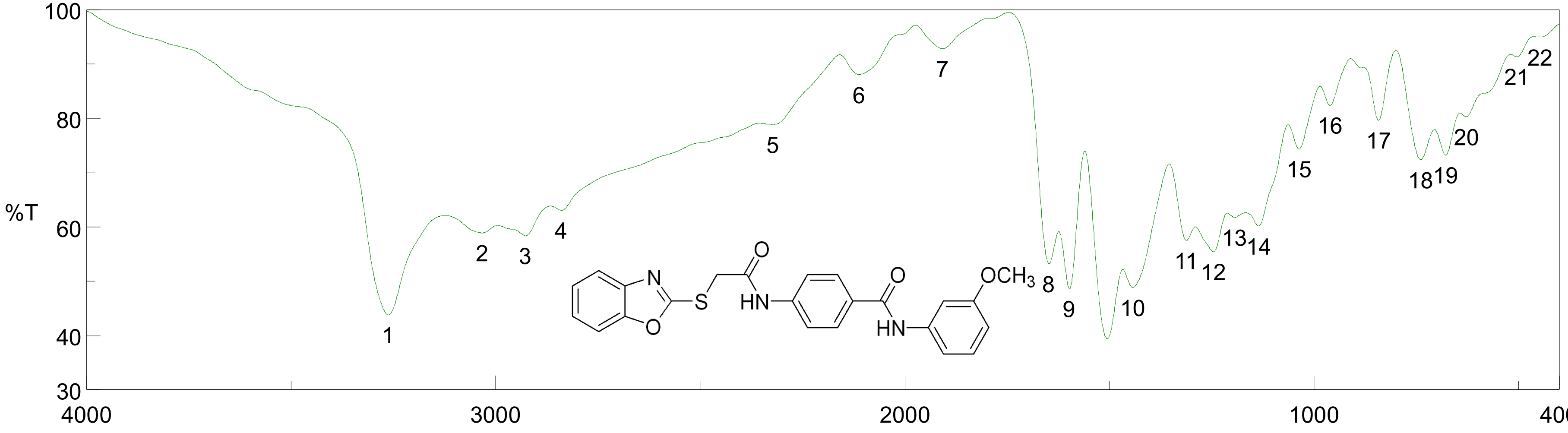

[Result of Peak Picking]

No.	Position	Intensity	No.	Position	Intensity	No.	Position	Intensity	No.	Position	Intensity
1	3262.97	43.7759	2	3033.48	58.8719	3	2927.41	58.3647	4	2839.67	63.038
5	2321.87	78.8344	6	2111.67	88.059	7	1907.25	92.8002	8	1647.88	53.2061
9	1597.73	48.5754	10	1442.49	48.7845	11	1311.36	57.4677	12	1245.79	55.4101
13	1193.72	61.7122	14	1134.9	60.1479	15	1035.59	74.3341	16	960.377	82.3621
17	841.776	79.6231	18	738.603	72.3662	19	677.856	73.2267	20	626.752	80.2756
21	503.33	91.3321	22	447.404	94.9492						

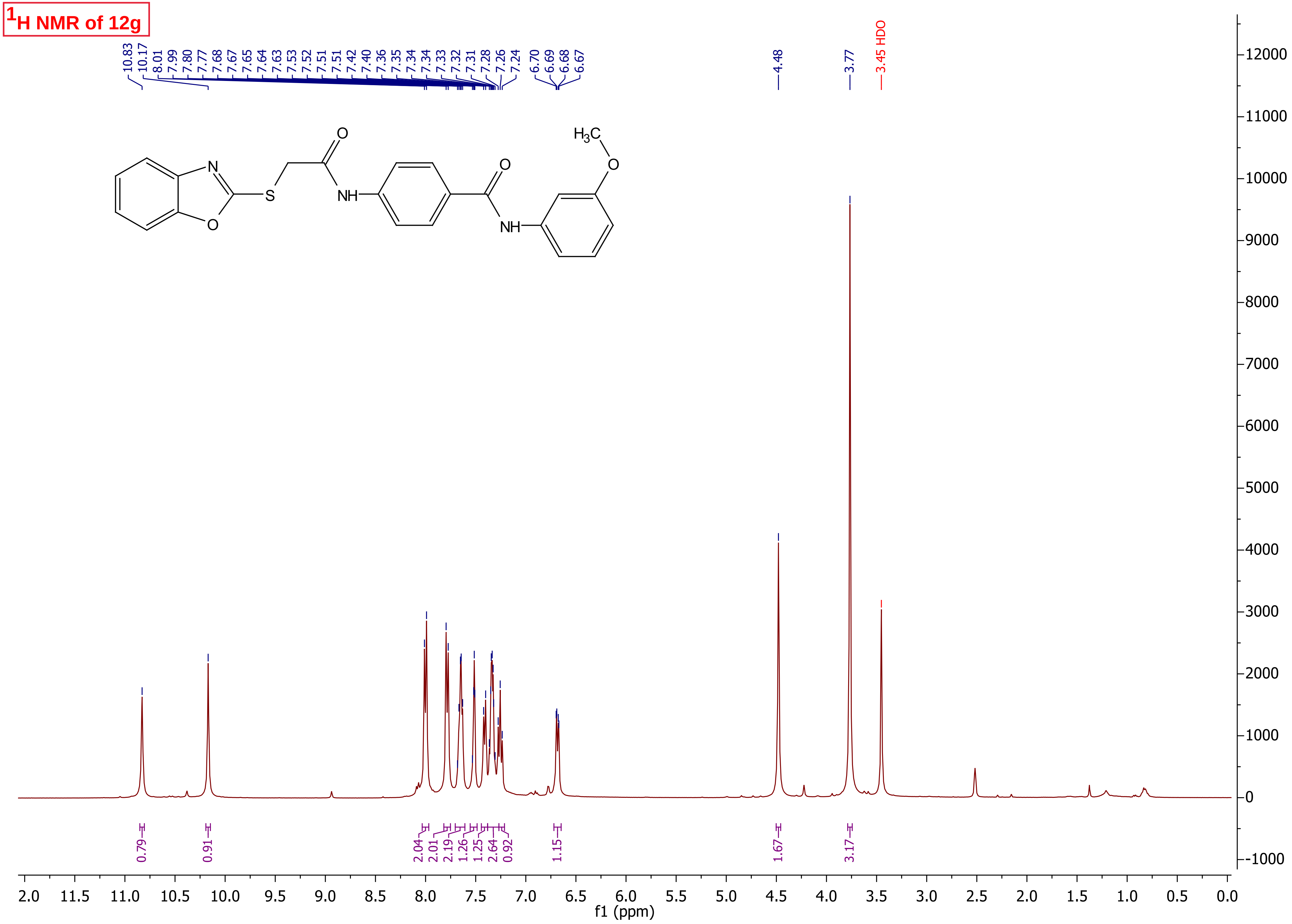

1H NMR of 12g
10.83
10.17
8.01
7.99
7.80
7.77
7.68
7.67
7.65
7.64
7.63
7.53
7.52
7.51
7.51
7.42
7.40
7.36
7.35
7.34
7.34
7.33
7.32
7.31
7.28
7.26
7.24
6.70
6.69
6.68
6.67
4.48
3.77
3.45 HDO
0.79
0.91
2.04
2.01
2.19
1.26
1.25
2.64
0.92
1.15
1.67
3.17
f1 (ppm)

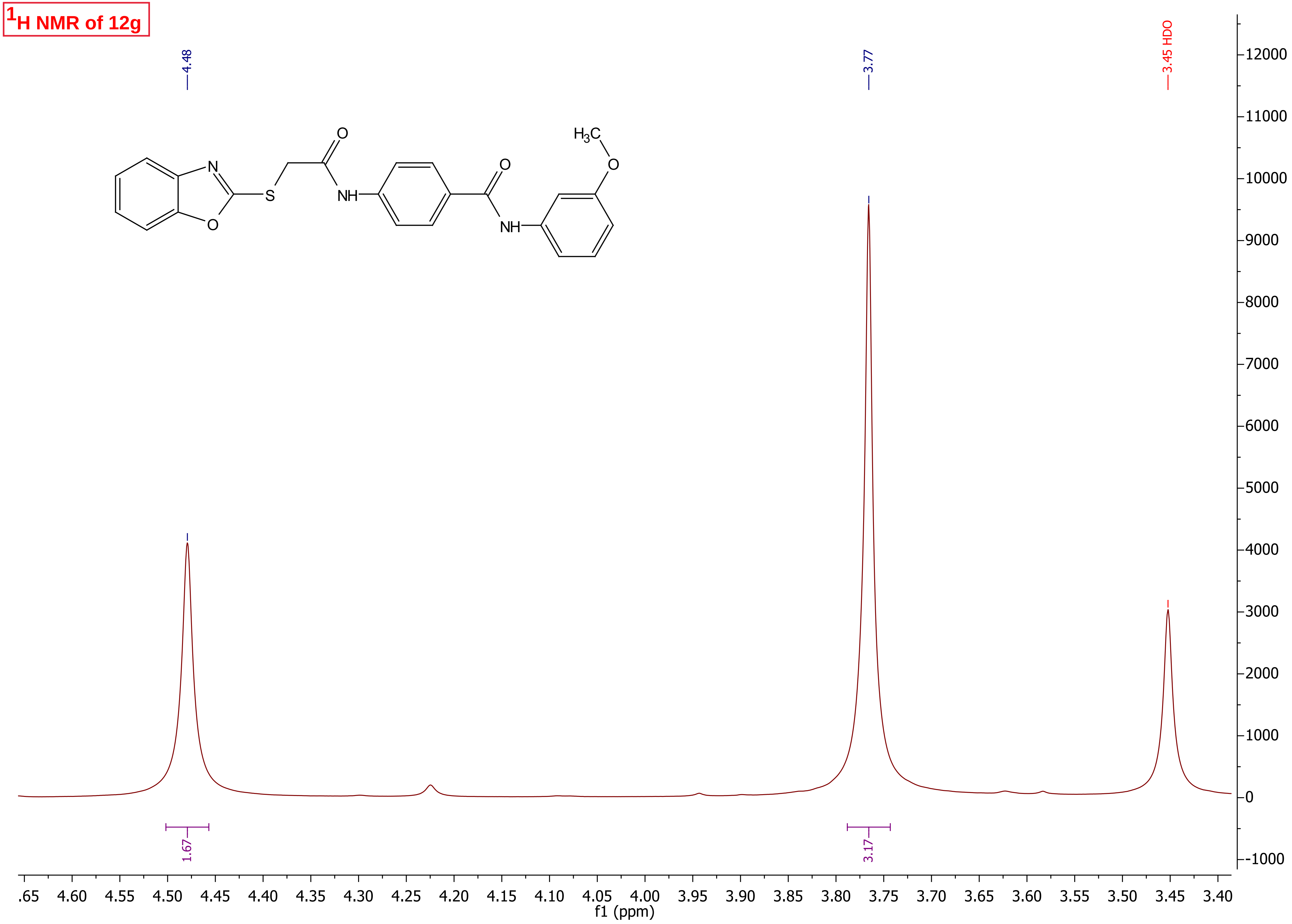

1H NMR of 12g
4.48
3.77
3.45 HDO
H3C
O
N
S
NH
O
NH
O
O
1.67
3.17
f1 (ppm)

^{1}H NMR of 12g

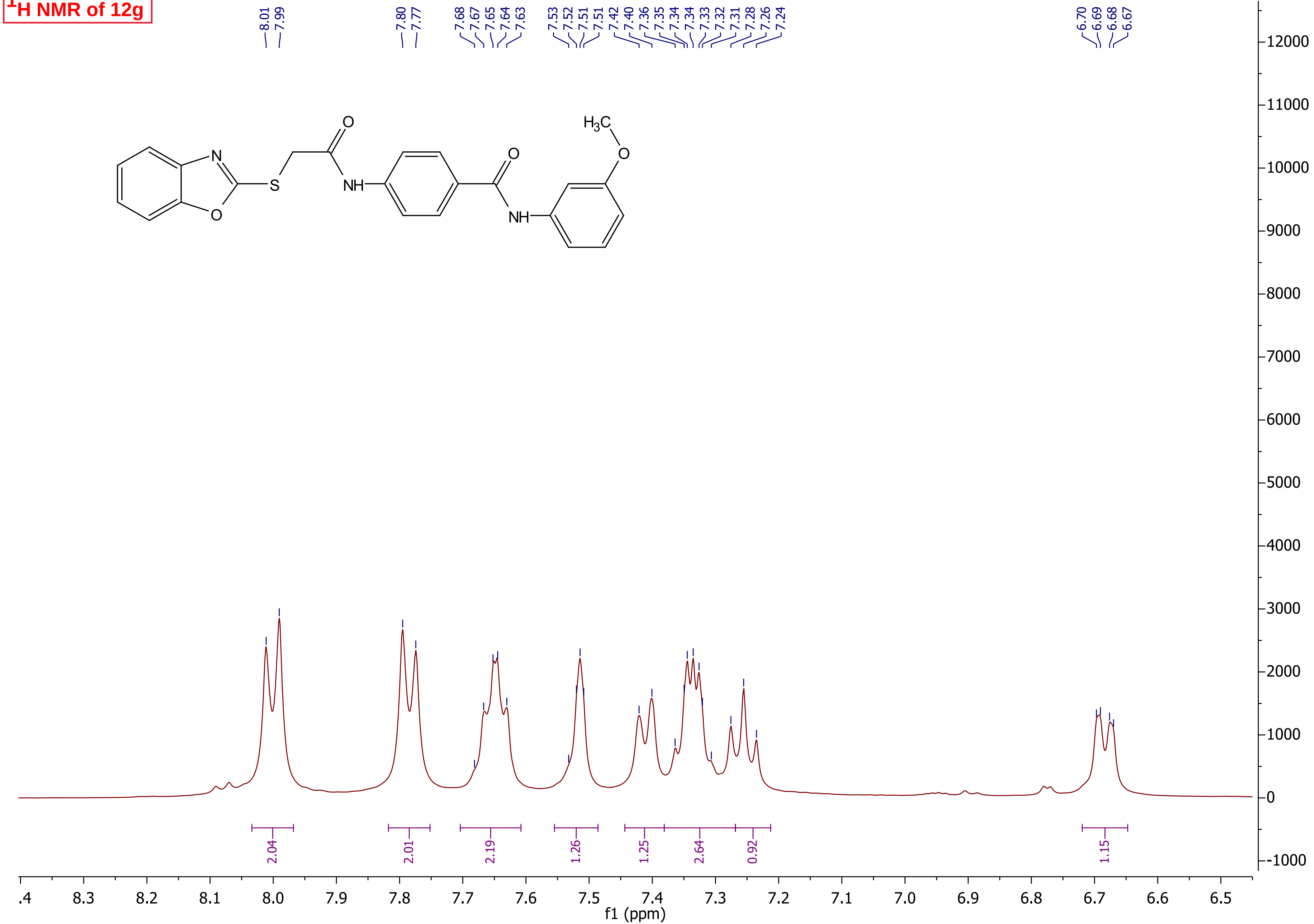
8.01
7.99
7.80
7.77
7.68
7.67
7.65
7.64
7.63
7.53
7.52
7.51
7.51
7.42
7.40
7.36
7.35
7.34
7.34
7.33
7.32
7.31
7.28
7.26
7.24
6.70
6.69
6.68
6.67
2.04
2.01
2.19
1.26
1.25
2.64
0.92
1.15
f1 (ppm)

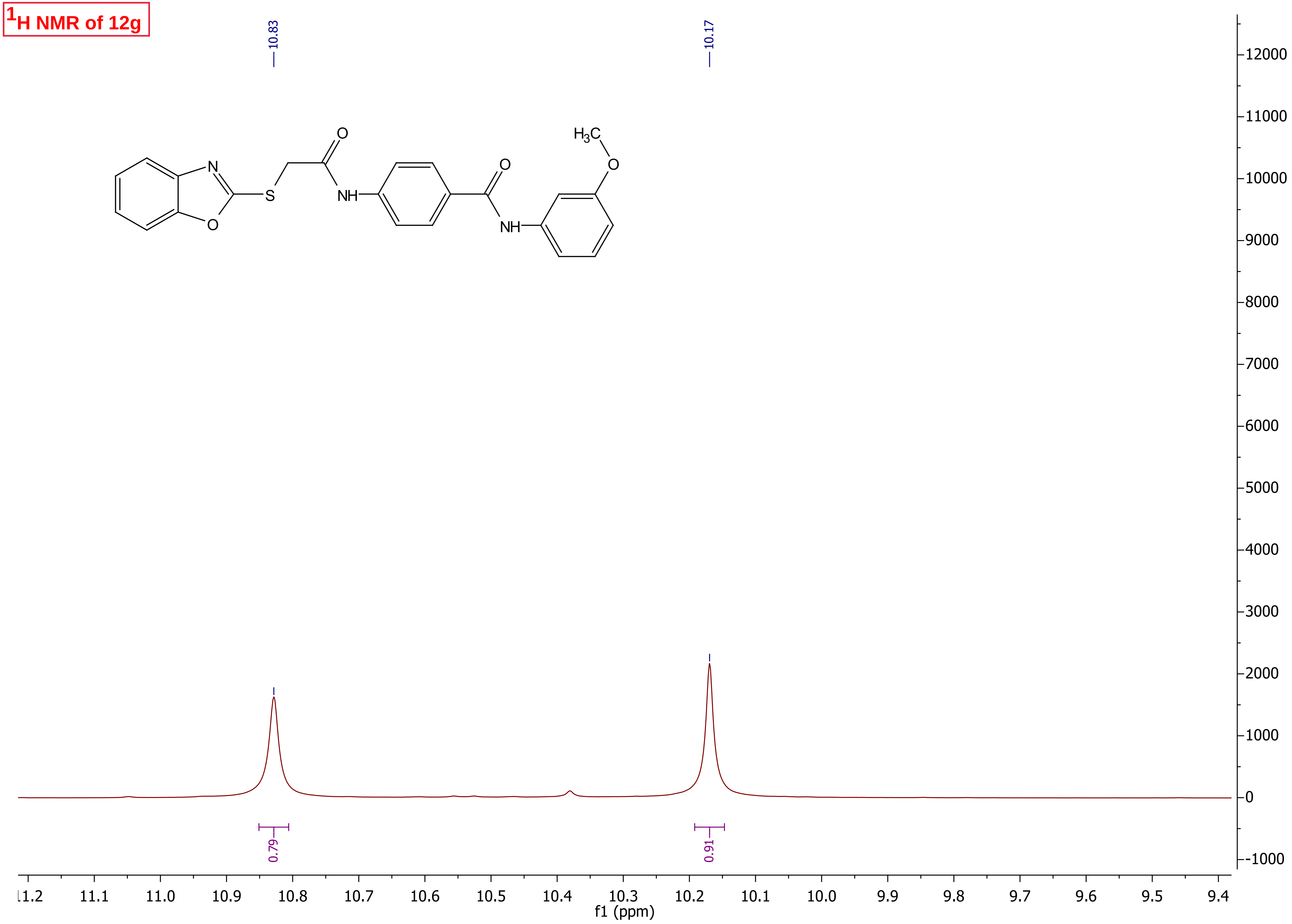
1H NMR of 12g
10.83
10.17
0.79
0.91
f1 (ppm)

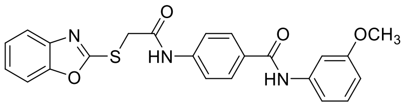

Mass spec. of 12g

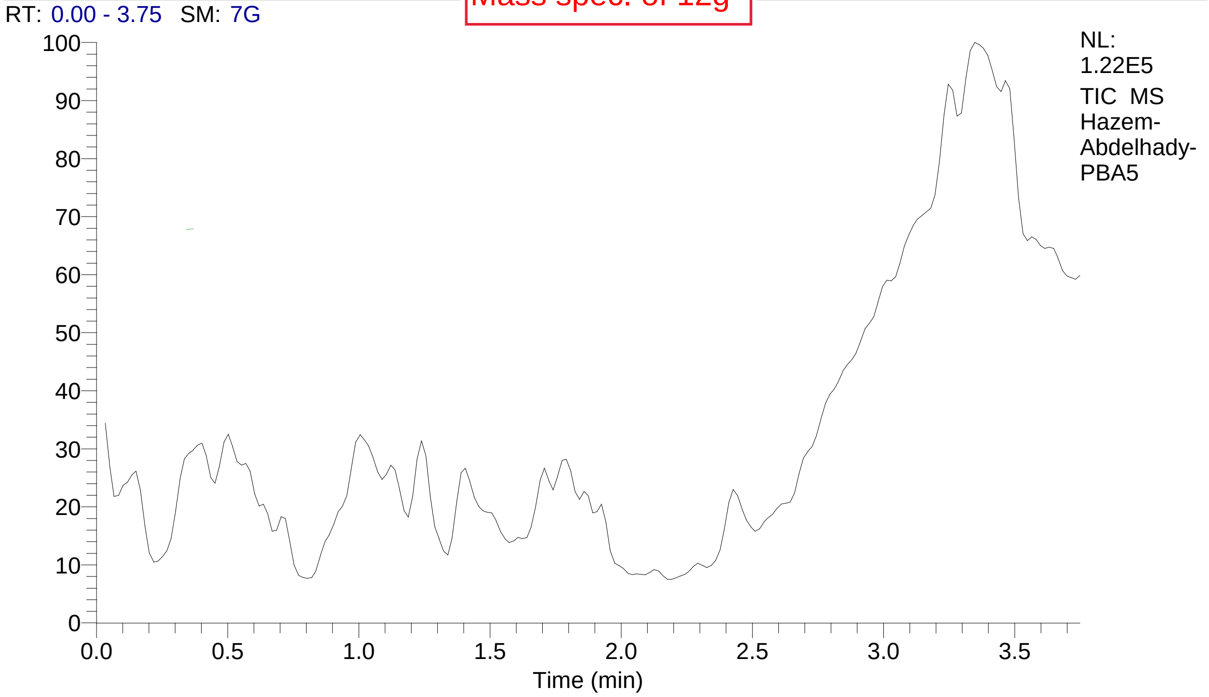

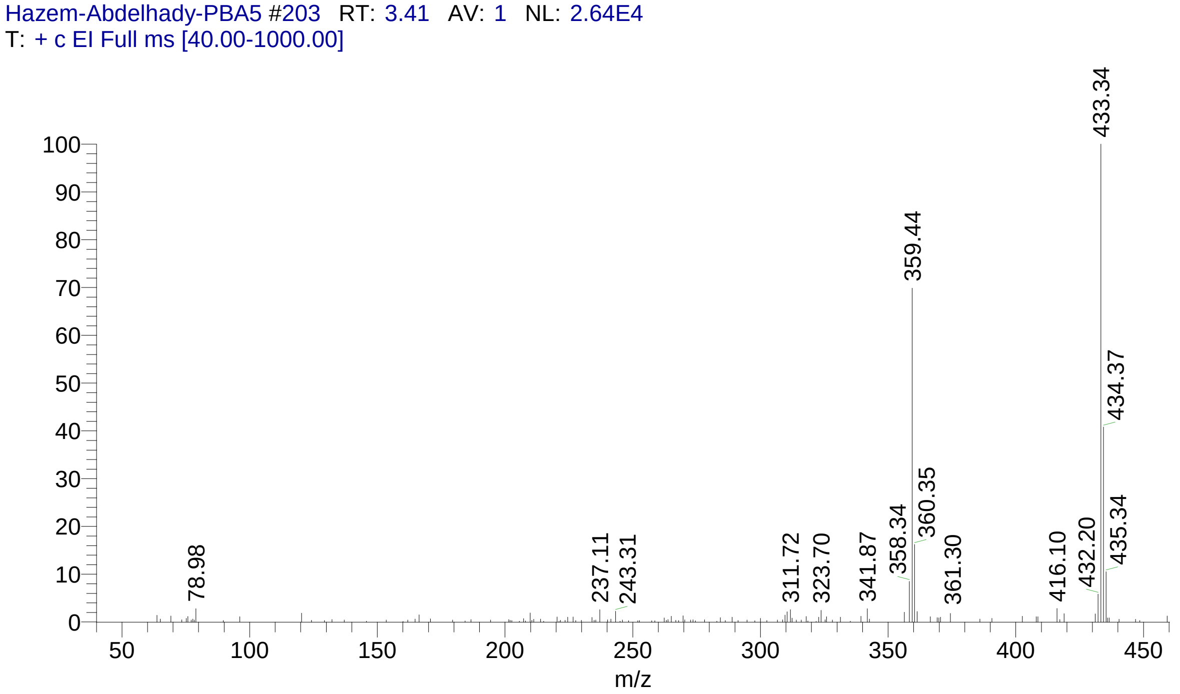

IR Of Comp. 12h

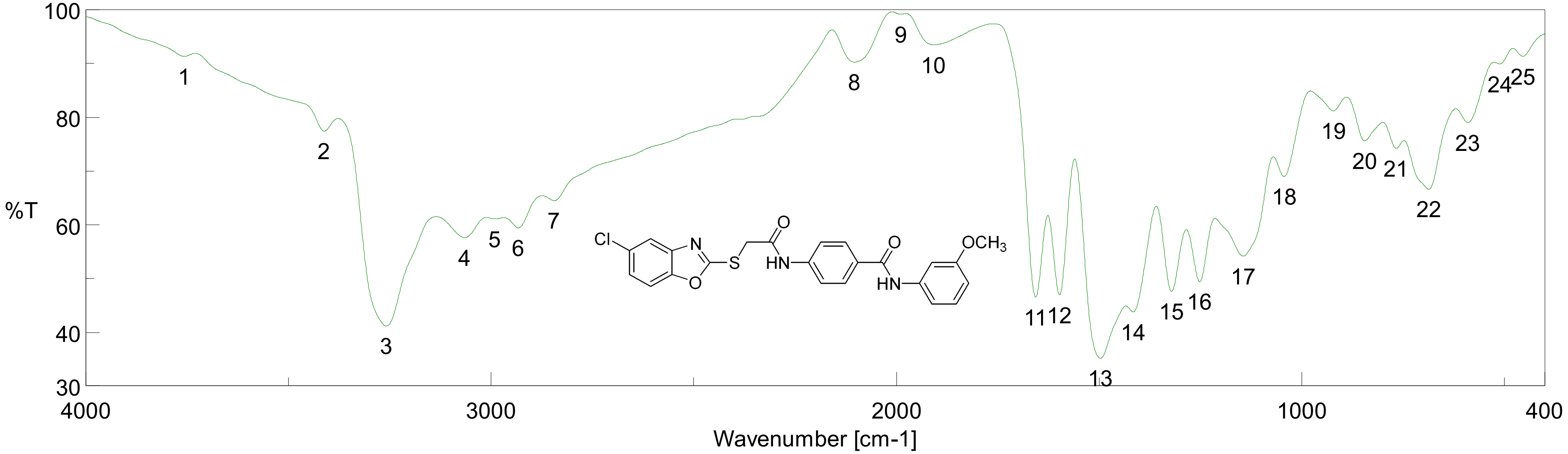

[Result of Peak Picking]

No.	Position	Intensity	No.	Position	Intensity	No.	Position	Intensity	No.	Position	Intensity
1	3757.62	91.2874	2	3412.42	77.3799	3	3259.11	41.1458	4	3065.3	57.5724
5	2991.05	61.071	6	2933.2	59.3891	7	2844.49	64.4634	8	2102.99	90.2239
9	1989.21	99.1409	10	1908.22	93.445	11	1656.55	46.5189	12	1597.73	46.913
13	1496.49	35.1202	14	1415.49	43.7665	15	1321	47.572	16	1251.58	49.393
17	1144.55	54.1192	18	1043.3	68.9323	19	921.807	81.1612	20	844.669	75.5803
21	766.566	74.1774	22	686.534	66.5846	23	590.111	78.9814	24	511.044	89.8717
25	454.154	91.3038									

^{1}H NMR of 12h

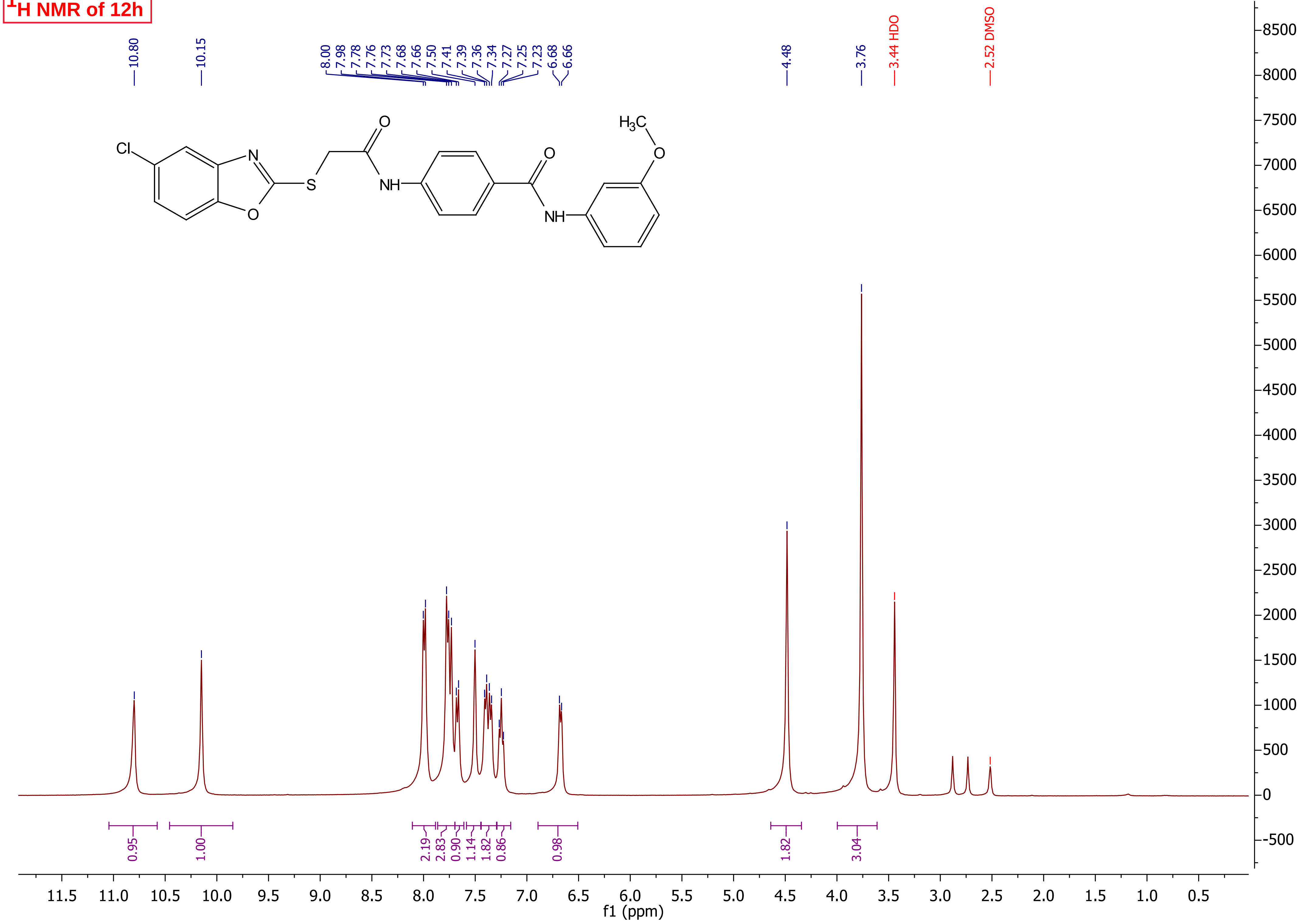
10.80
10.15
8.00
7.98
7.78
7.76
7.73
7.68
7.66
7.50
7.41
7.39
7.36
7.34
7.27
7.25
7.23
6.68
6.66
4.48
3.76
3.44 HDO
2.52 DMSO
Cl
N
O
S
O
NH
O
NH
H3C
O
0.95
1.00
2.19
2.83
0.90
1.14
1.82
0.86
0.98
1.82
3.04
11.5
11.0
10.5
10.0
9.5
9.0
8.5
8.0
7.5
7.0
6.5
6.0
5.5
5.0
4.5
4.0
3.5
3.0
2.5
2.0
1.5
1.0
0.5
f1 (ppm)
8500
8000
7500
7000
6500
6000
5500
5000
4500
4000
3500
3000
2500
2000
1500
1000
500
0
-500

^{1}H NMR of 12h

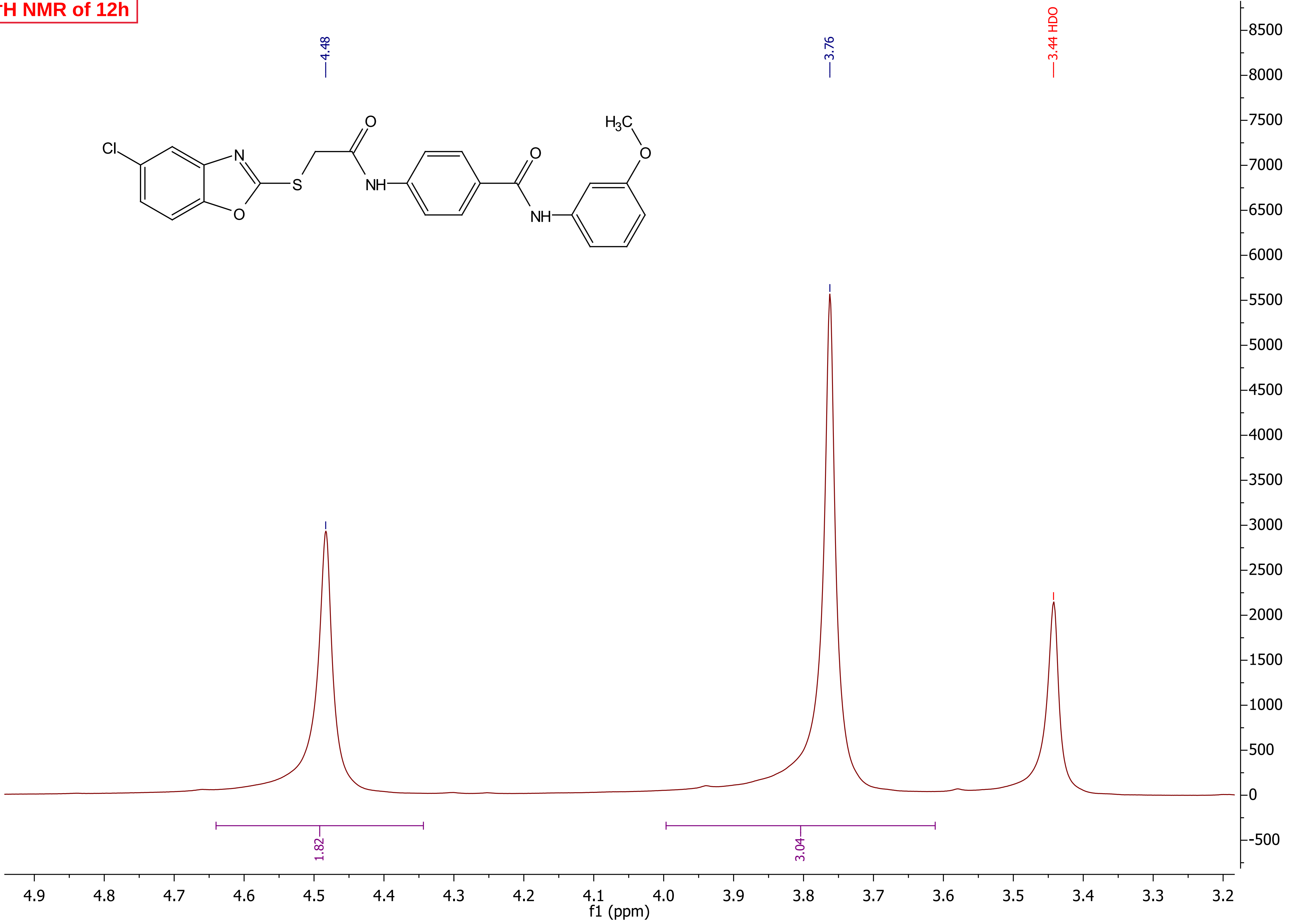

4.48
3.76
3.44 HDO
Cl
N
O
S
O
NH
O
NH
H3C
O
1.82
3.04
4.9
4.8
4.7
4.6
4.5
4.4
4.3
4.2
4.1
4.0
3.9
3.8
3.7
3.6
3.5
3.4
3.3
3.2
f1 (ppm)
8500
8000
7500
7000
6500
6000
5500
5000
4500
4000
3500
3000
2500
2000
1500
1000
500
0
-500

^{1}H NMR of 12h

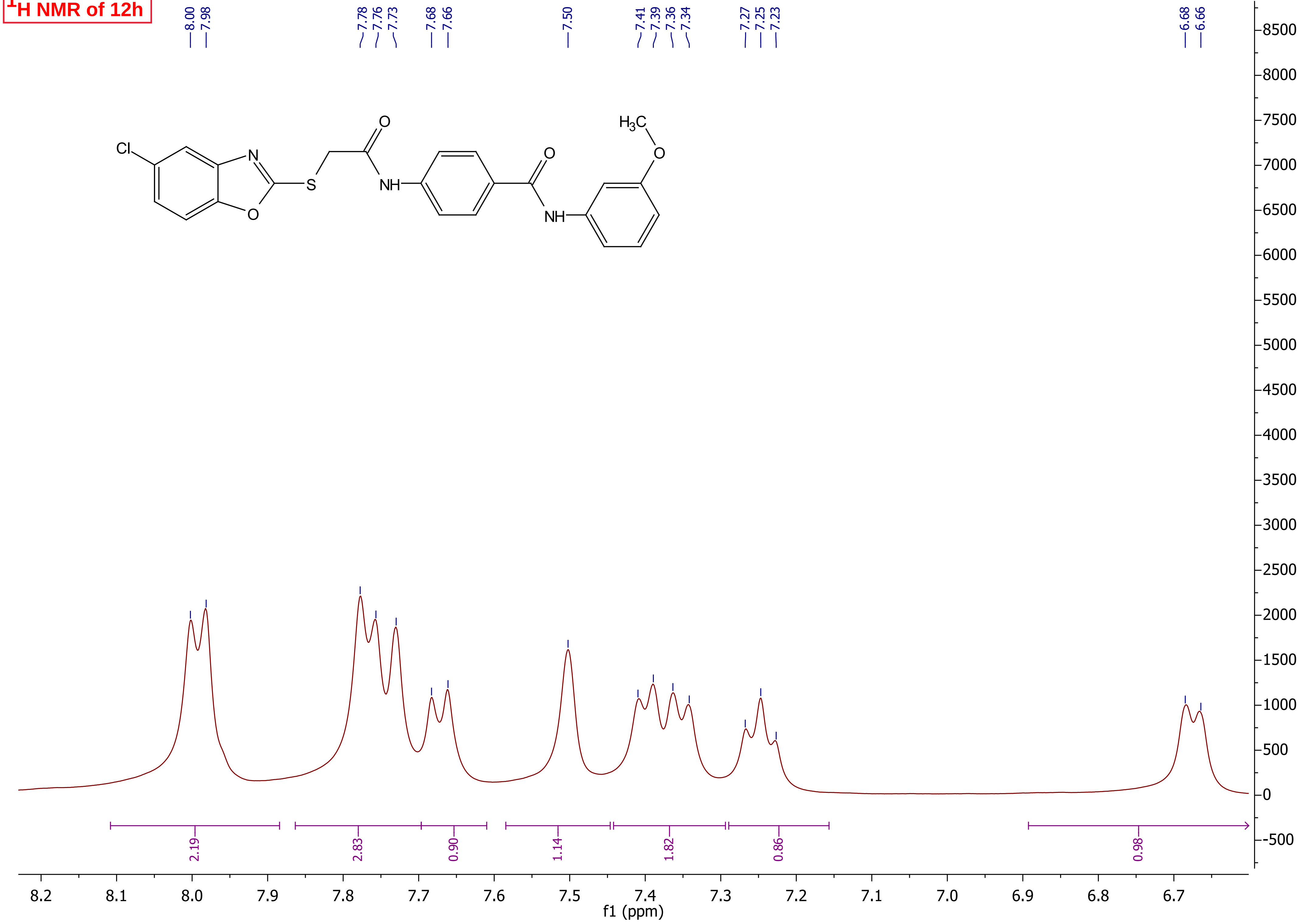

8.00
7.98
7.78
7.76
7.73
7.68
7.66
7.50
7.41
7.39
7.36
7.34
7.27
7.25
7.23
6.68
6.66
Cl
N
O
S
O
NH
O
NH
H3C
O
2.19
2.83
0.90
1.14
1.82
0.86
0.98
f1 (ppm)

^{1}H NMR of 12h

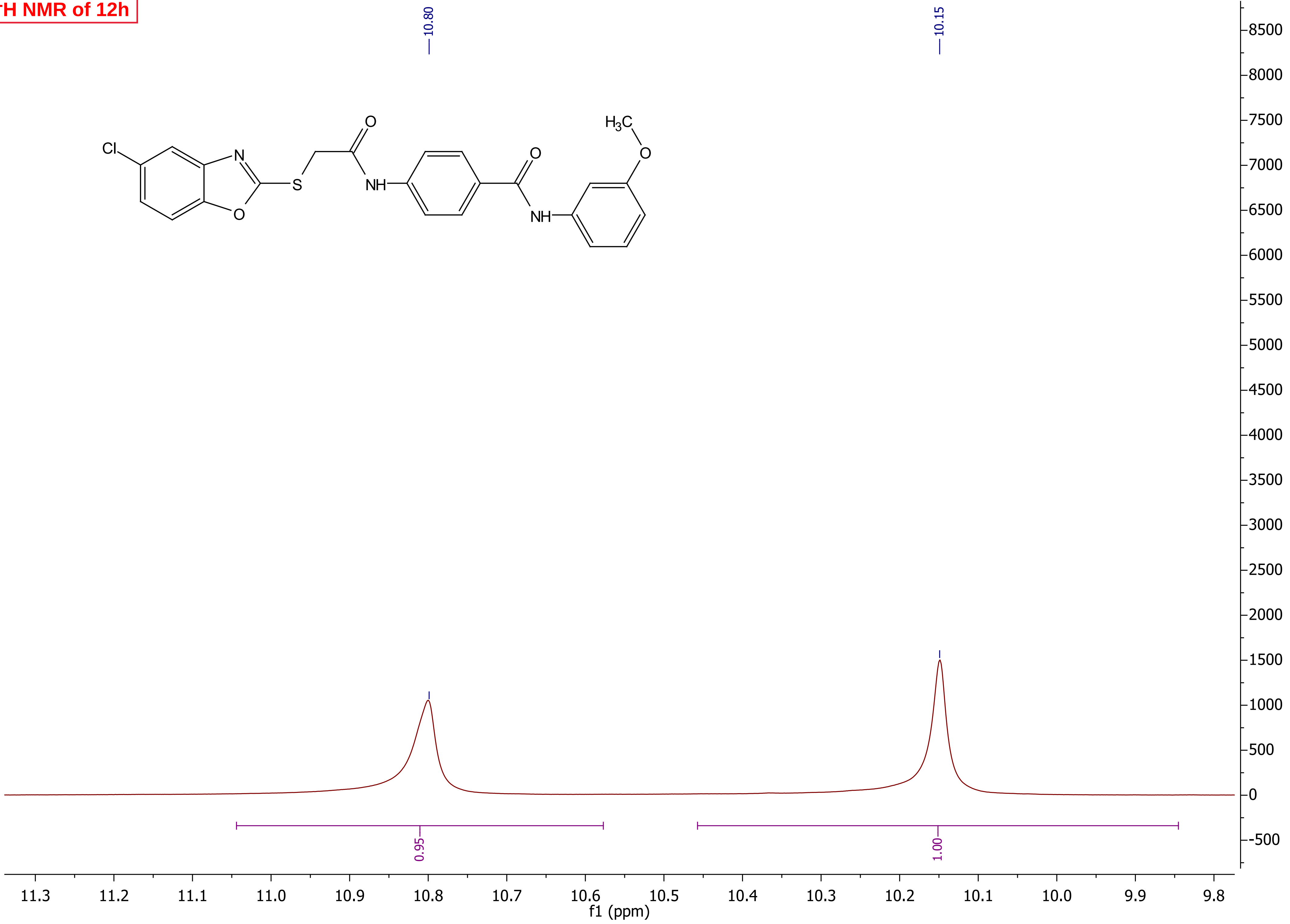
10.80
10.15
Cl
N
O
S
O
NH
O
NH
H_3C
O
0.95
1.00
11.3
11.2
11.1
11.0
10.9
10.8
10.7
10.6
10.5
10.4
10.3
10.2
10.1
10.0
9.9
9.8
f1 (ppm)
8500
8000
7500
7000
6500
6000
5500
5000
4500
4000
3500
3000
2500
2000
1500
1000
500
0
-500

IR Of Comp. 12i

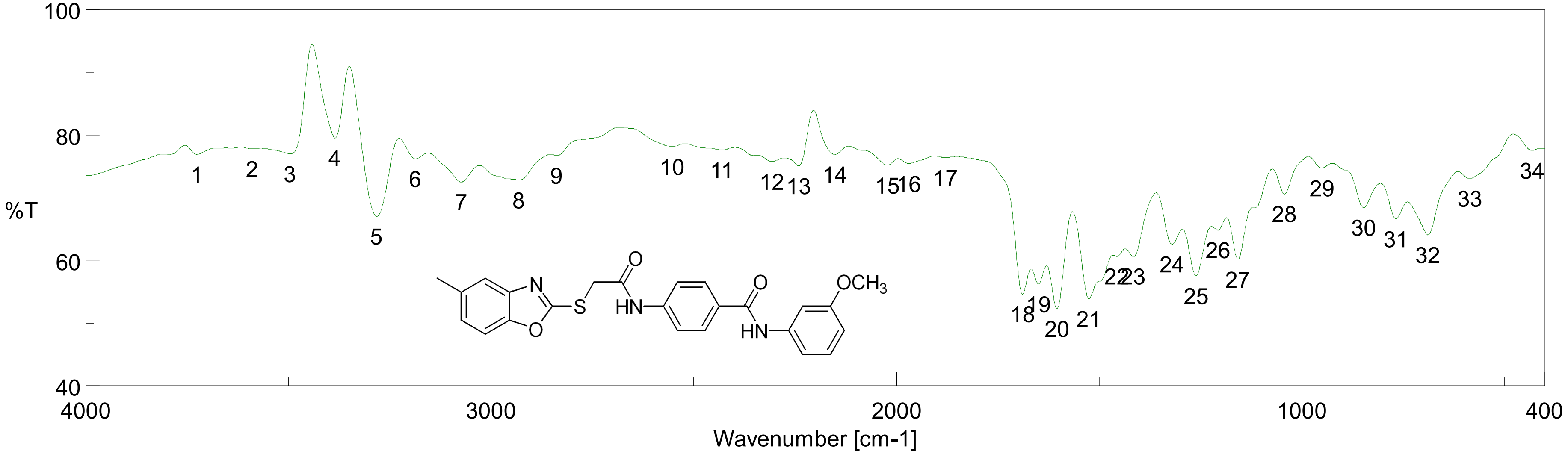

[Result of Peak Picking]

No.	Position	Intensity	No.	Position	Intensity	No.	Position	Intensity	No.	Position	Intensity
1	3725.8	76.886	2	3589.84	77.7685	3	3496.31	77.0158	4	3385.42	79.507
5	3282.25	66.9992	6	3186.79	76.1775	7	3073.98	72.4882	8	2931.27	72.8111
9	2837.74	76.7254	10	2553.29	78.1583	11	2430.83	77.6224	12	2307.41	75.7794
13	2240.88	75.0879	14	2152.17	76.8674	15	2022.96	75.1694	16	1968.96	75.438
17	1879.29	76.3926	18	1688.37	54.6311	19	1648.84	56.2265	20	1603.52	52.2829
21	1525.42	53.8957	22	1455.99	60.6483	23	1415.49	60.5682	24	1320.04	62.5676
25	1260.25	57.5393	26	1206.26	64.8379	27	1157.08	60.1462	28	1042.34	70.5748
29	949.77	74.7438	30	846.597	68.4009	31	766.566	66.6274	32	688.463	64.0647
33	585.29	73.0553	34	431.012	77.5389						

^{1}H NMR of 12i

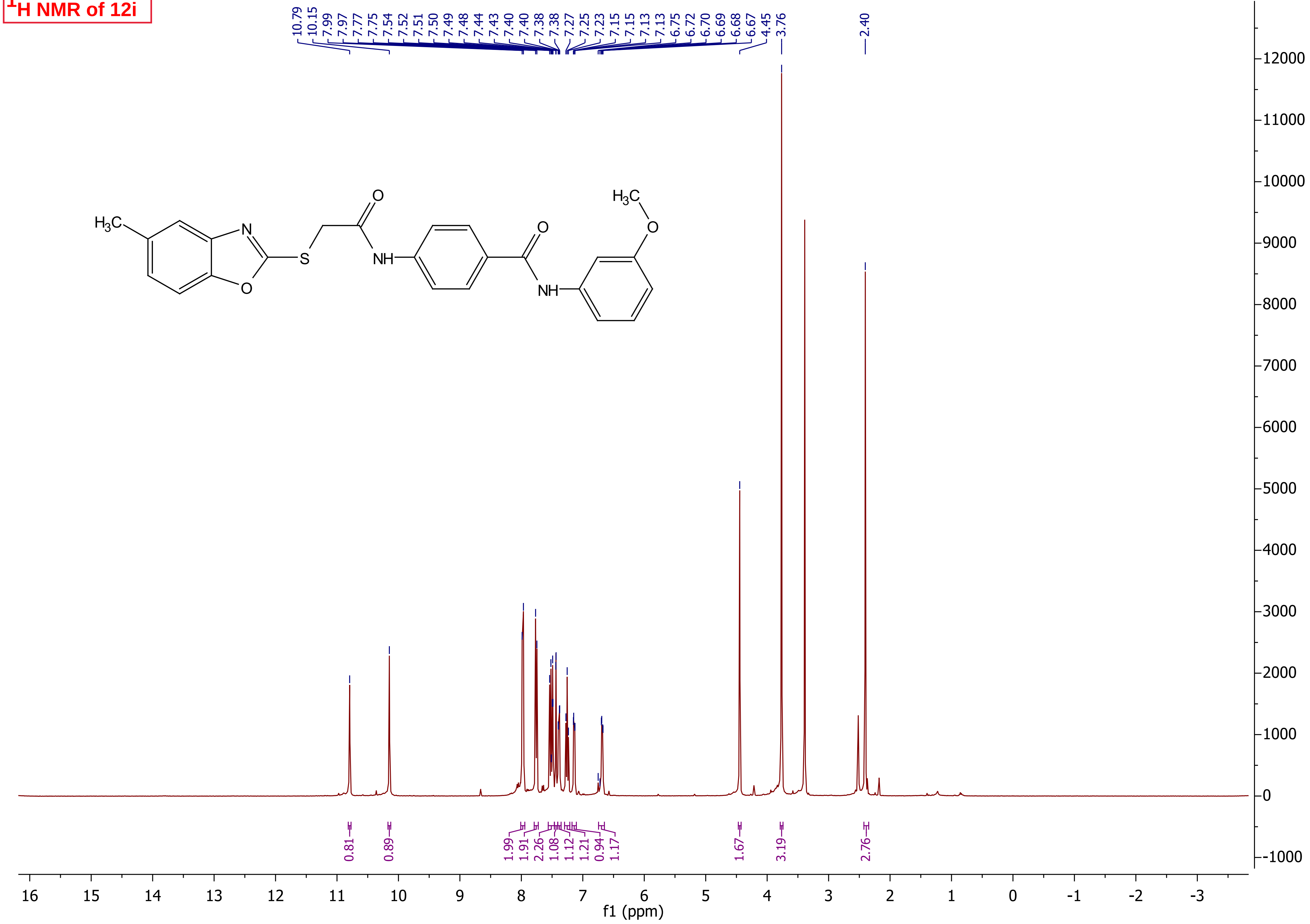
10.79
10.15
7.99
7.97
7.77
7.75
7.54
7.52
7.51
7.50
7.49
7.48
7.44
7.43
7.40
7.40
7.38
7.38
7.27
7.25
7.23
7.15
7.15
7.13
7.13
6.75
6.72
6.70
6.69
6.68
6.67
4.45
3.76
2.40
0.81
0.89
1.99
1.91
2.26
1.08
1.12
1.21
0.94
1.17
1.67
3.19
2.76
f1 (ppm)

^{1}H NMR of 12i

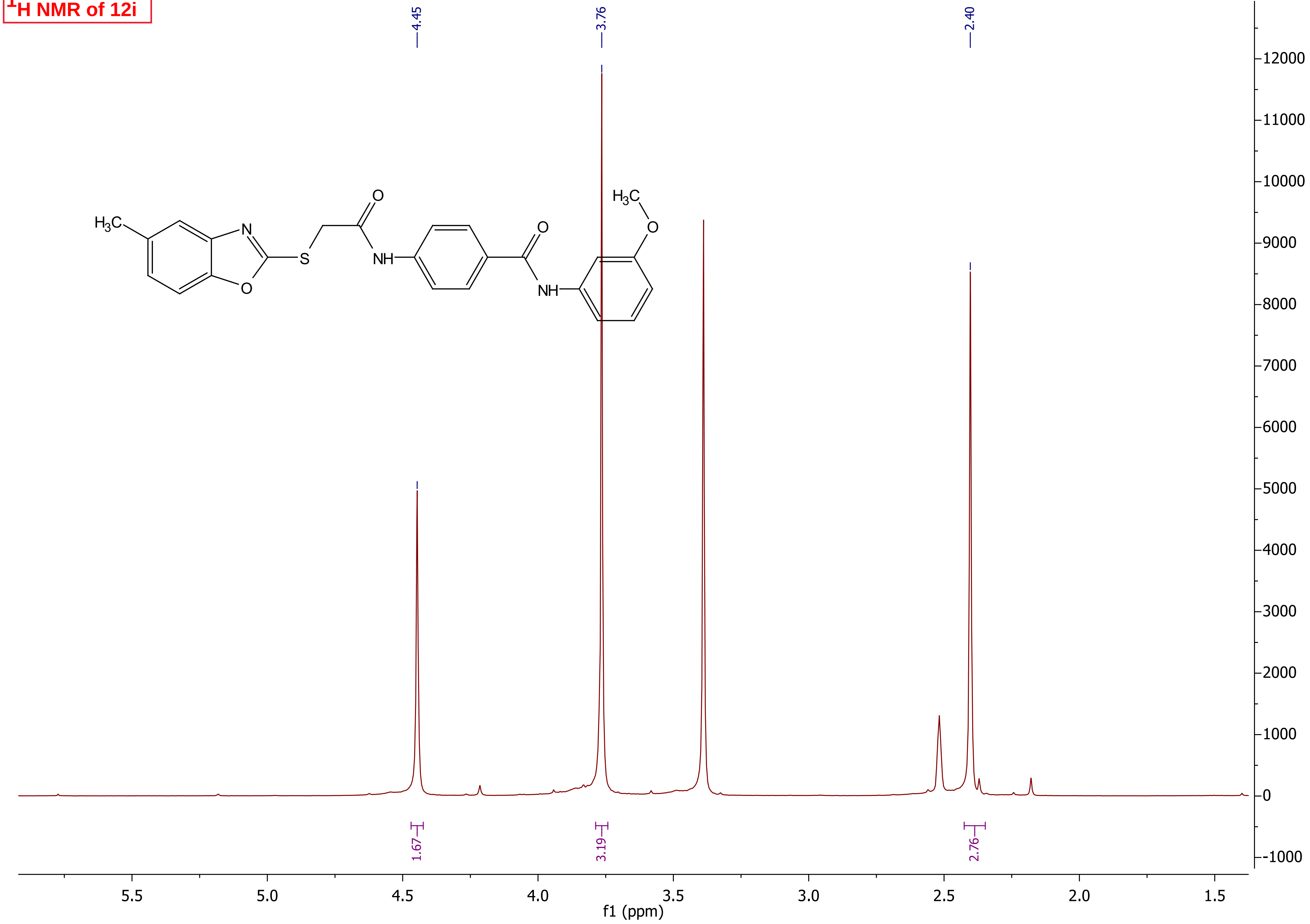

4.45
3.76
2.40
H_3C
N
O
S
NH
O
NH
H_3C
O
O
1.67
3.19
2.76
12000
11000
10000
9000
8000
7000
6000
5000
4000
3000
2000
1000
0
-1000
5.5
5.0
4.5
4.0
3.5
3.0
2.5
2.0
1.5
f1 (ppm)

^{1}H NMR of 12i

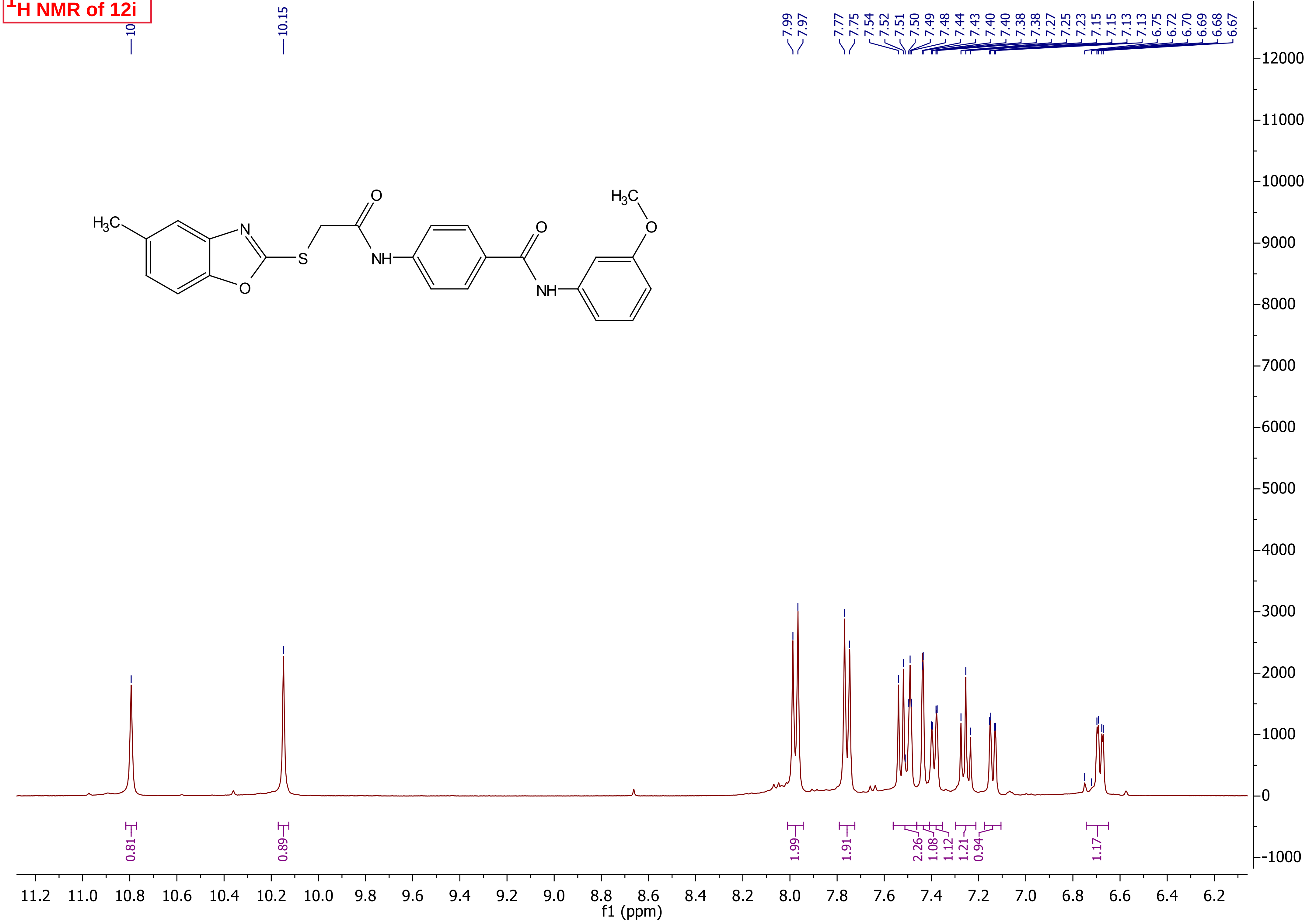
10.80
10.15
7.99
7.97
7.77
7.75
7.54
7.52
7.51
7.50
7.49
7.48
7.44
7.43
7.40
7.40
7.38
7.38
7.27
7.25
7.23
7.15
7.15
7.13
7.13
6.75
6.72
6.70
6.69
6.68
6.67
H3C
N
O
S
O
NH
O
NH
H3C
O
0.81
0.89
1.99
1.91
2.26
1.08
1.12
1.21
0.94
1.17
f1 (ppm)

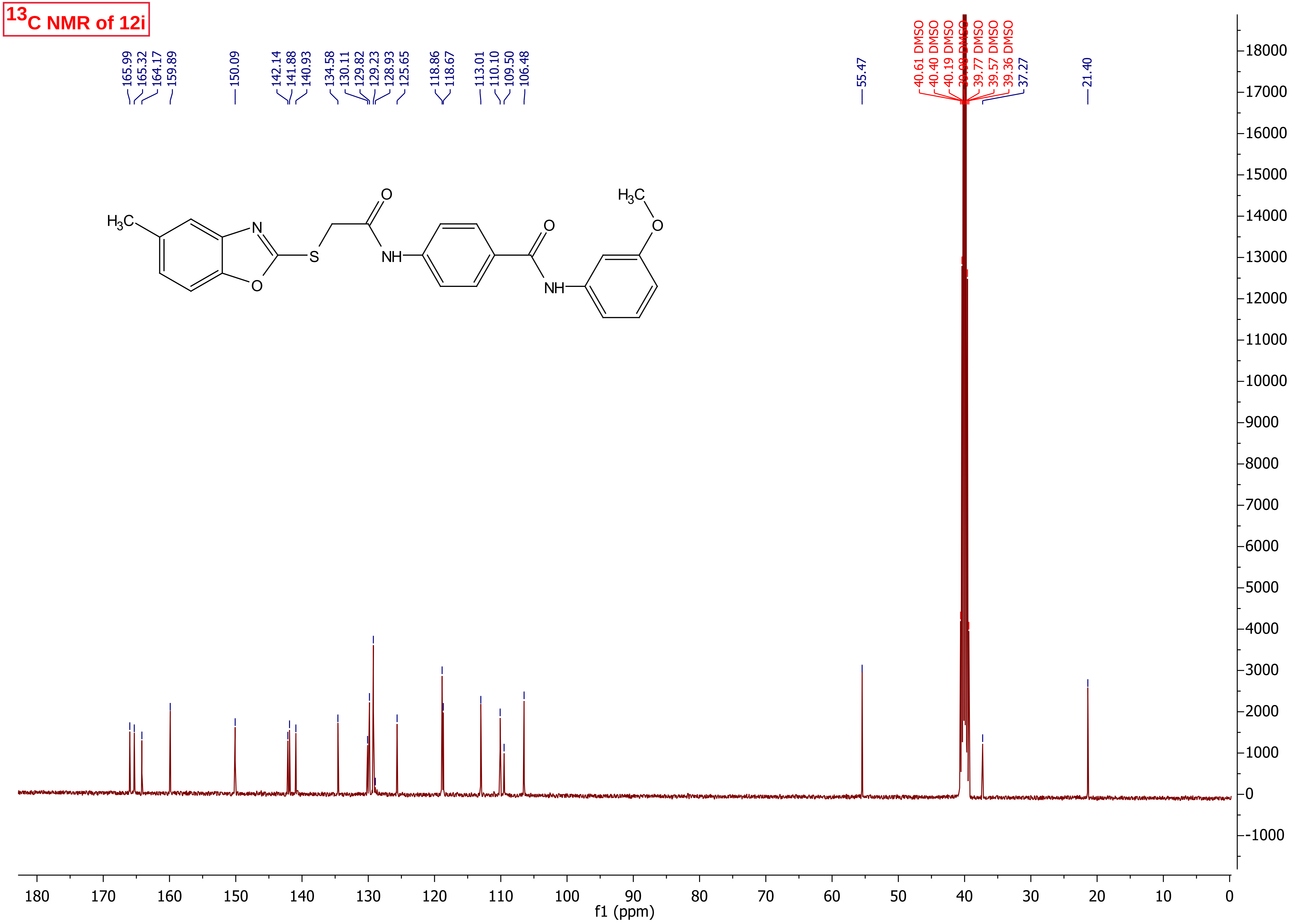
13C NMR of 12i
165.99
165.32
164.17
159.89
150.09
142.14
141.88
140.93
134.58
130.11
129.82
129.23
128.93
125.65
118.86
118.67
113.01
110.10
109.50
106.48
55.47
40.61 DMSO
40.40 DMSO
40.19 DMSO
39.98 DMSO
39.77 DMSO
39.57 DMSO
39.36 DMSO
37.27
21.40
H3C
N
O
S
NH
O
NH
O
H3C
O
f1 (ppm)

^{13}C NMR of 12i

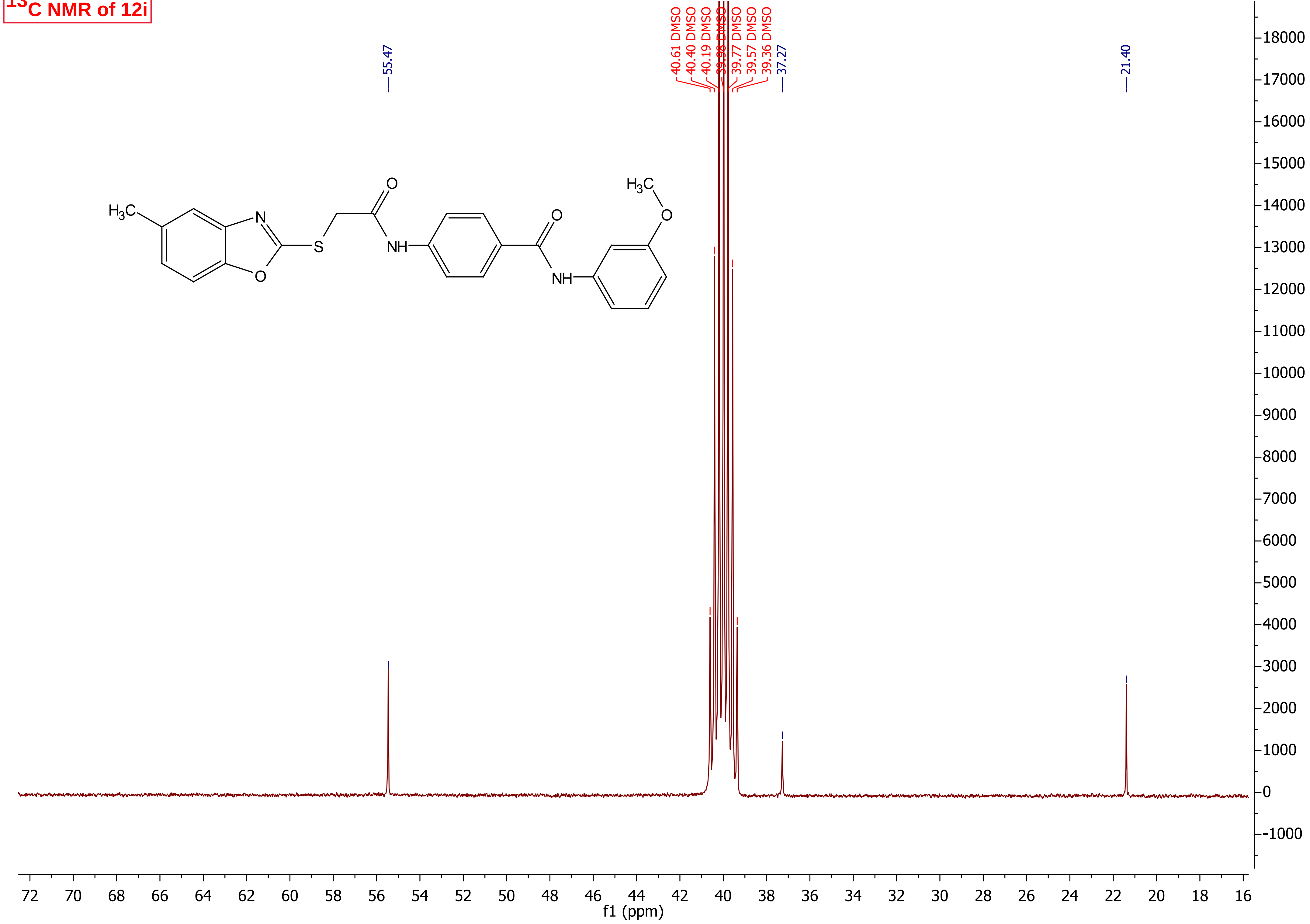

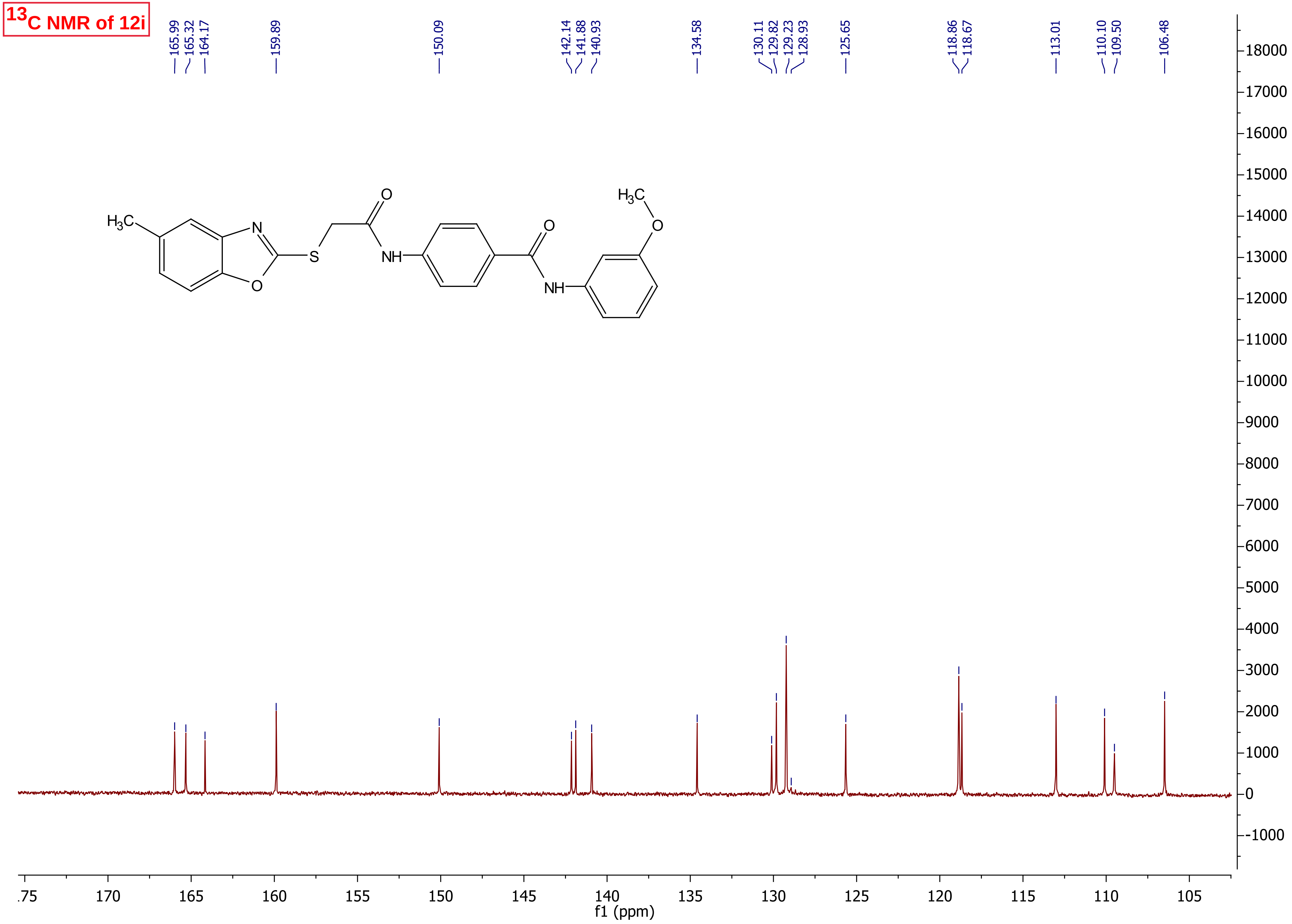

13C NMR of 12i
165.99
165.32
164.17
159.89
150.09
142.14
141.88
140.93
134.58
130.11
129.82
129.23
128.93
125.65
118.86
118.67
113.01
110.10
109.50
106.48
H3C
N
O
S
O
NH
O
NH
H3C
O
f1 (ppm)

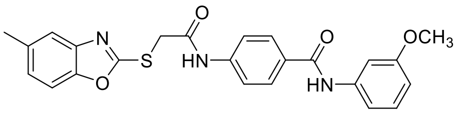

Mass spec. of 12i

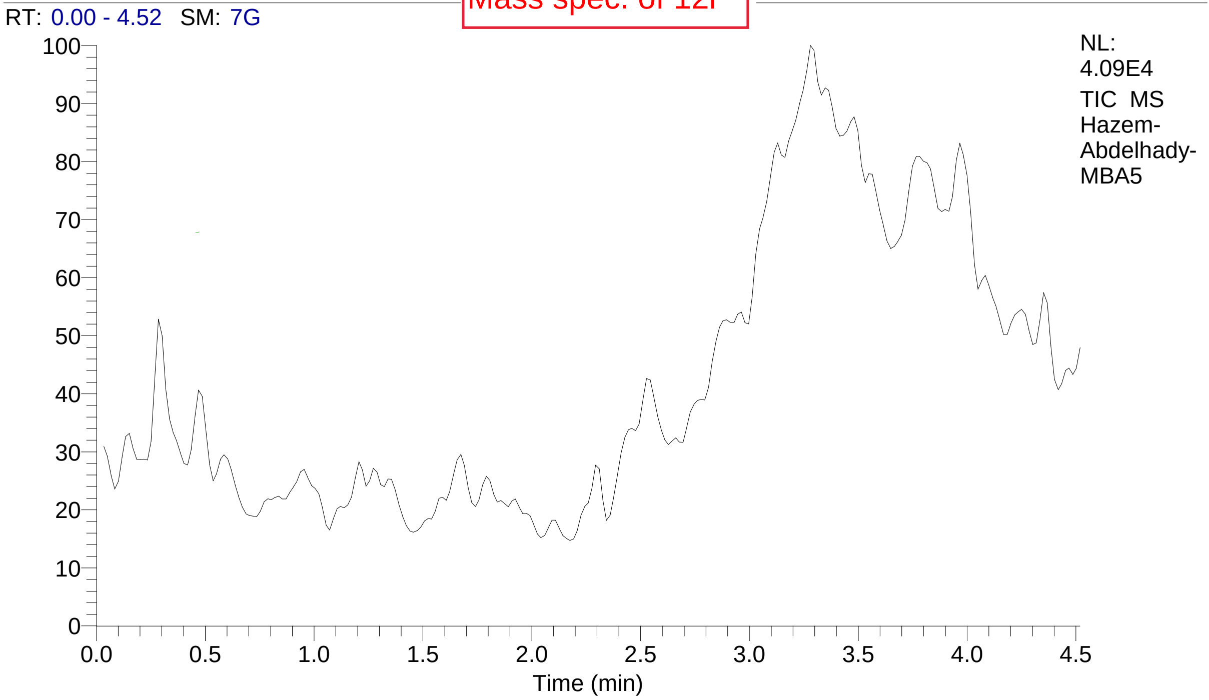

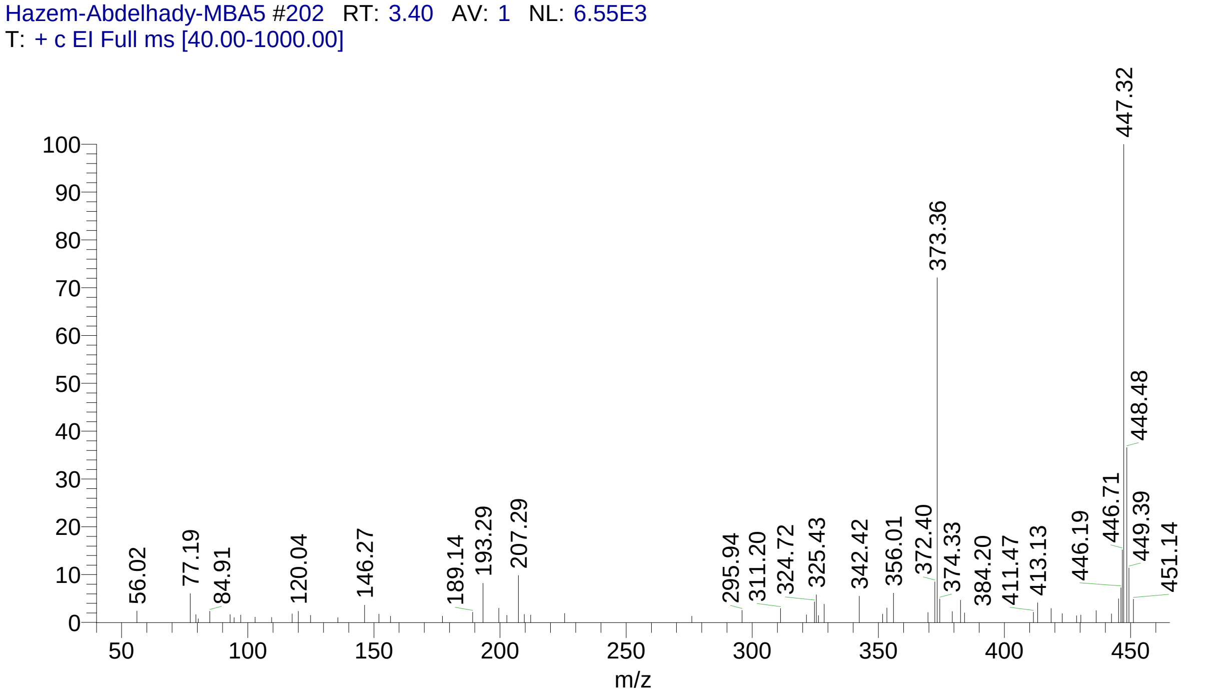

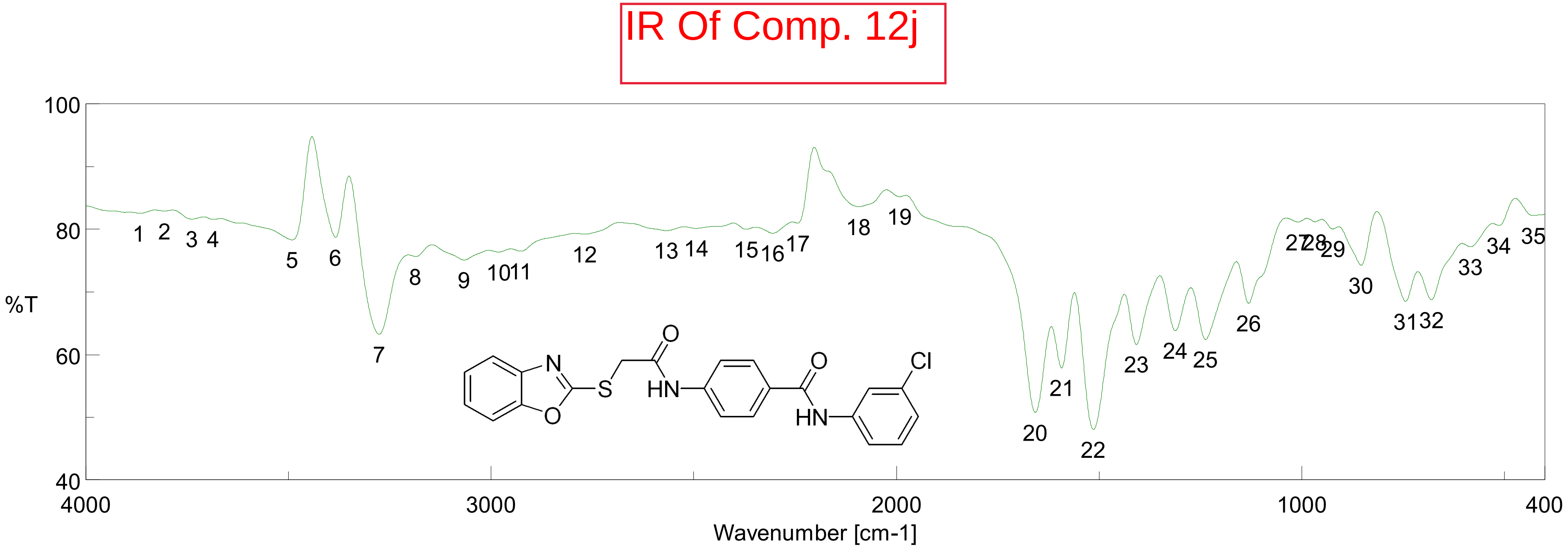

[Result of Peak Picking]

No.	Position	Intensity	No.	Position	Intensity	No.	Position	Intensity	No.	Position	Intensity
1	3869.47	82.5123	2	3805.83	82.8307	3	3738.33	81.5663	4	3685.3	81.5565
5	3491.49	78.2667	6	3384.46	78.6759	7	3276.47	63.2115	8	3186.79	75.6017
9	3066.26	75.0539	10	2981.41	76.326	11	2927.41	76.4633	12	2769.28	79.1763
13	2567.75	79.7397	14	2494.47	80.1086	15	2371.05	79.966	16	2305.48	79.3266
17	2243.77	80.9249	18	2093.35	83.5628	19	1992.11	85.1515	20	1657.52	50.7472
21	1591.95	57.8468	22	1513.85	48.0363	23	1407.78	61.5913	24	1312.32	63.8029
25	1237.11	62.3758	26	1131.05	68.1626	27	1009.55	81.1209	28	967.126	81.1564
29	922.771	80.0238	30	853.347	74.2327	31	743.424	68.4675	32	679.785	68.7035
33	583.361	77.1587	34	512.972	80.5657	35	428.12	82.1351			

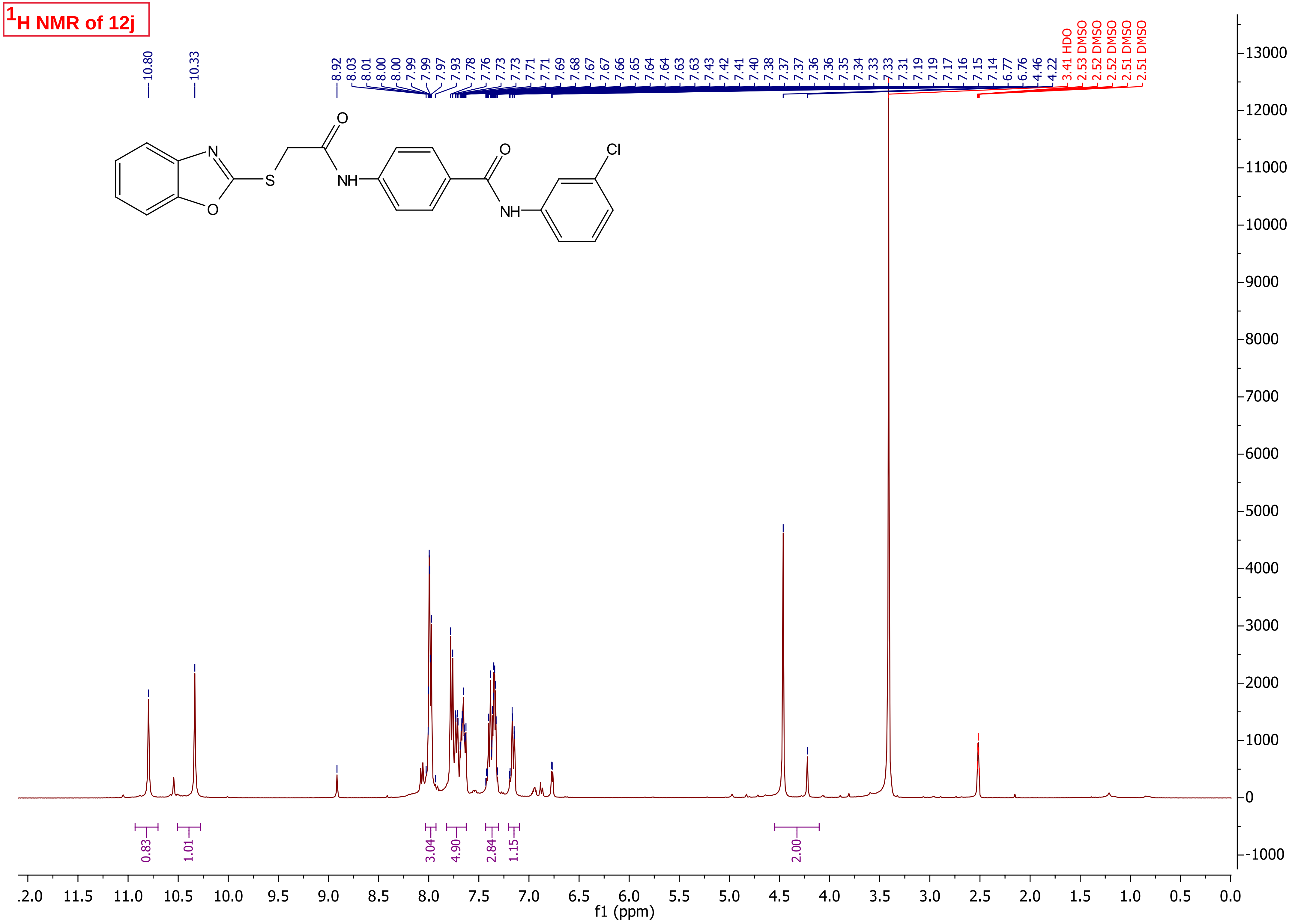

1H NMR of 12j
10.80
10.33
8.92
8.03
8.01
8.00
8.00
7.99
7.99
7.97
7.93
7.78
7.76
7.73
7.73
7.71
7.71
7.69
7.68
7.67
7.67
7.66
7.65
7.64
7.64
7.63
7.63
7.43
7.42
7.41
7.40
7.38
7.37
7.37
7.36
7.36
7.35
7.34
7.33
7.33
7.31
7.19
7.19
7.17
7.16
7.15
7.14
6.77
6.76
4.46
4.22
3.41 HDO
2.53 DMSO
2.52 DMSO
2.52 DMSO
2.51 DMSO
2.51 DMSO
N
O
S
NH
O
NH
Cl
0.83
1.01
3.04
4.90
2.84
1.15
2.00
f1 (ppm)

^{1}H NMR of 12j

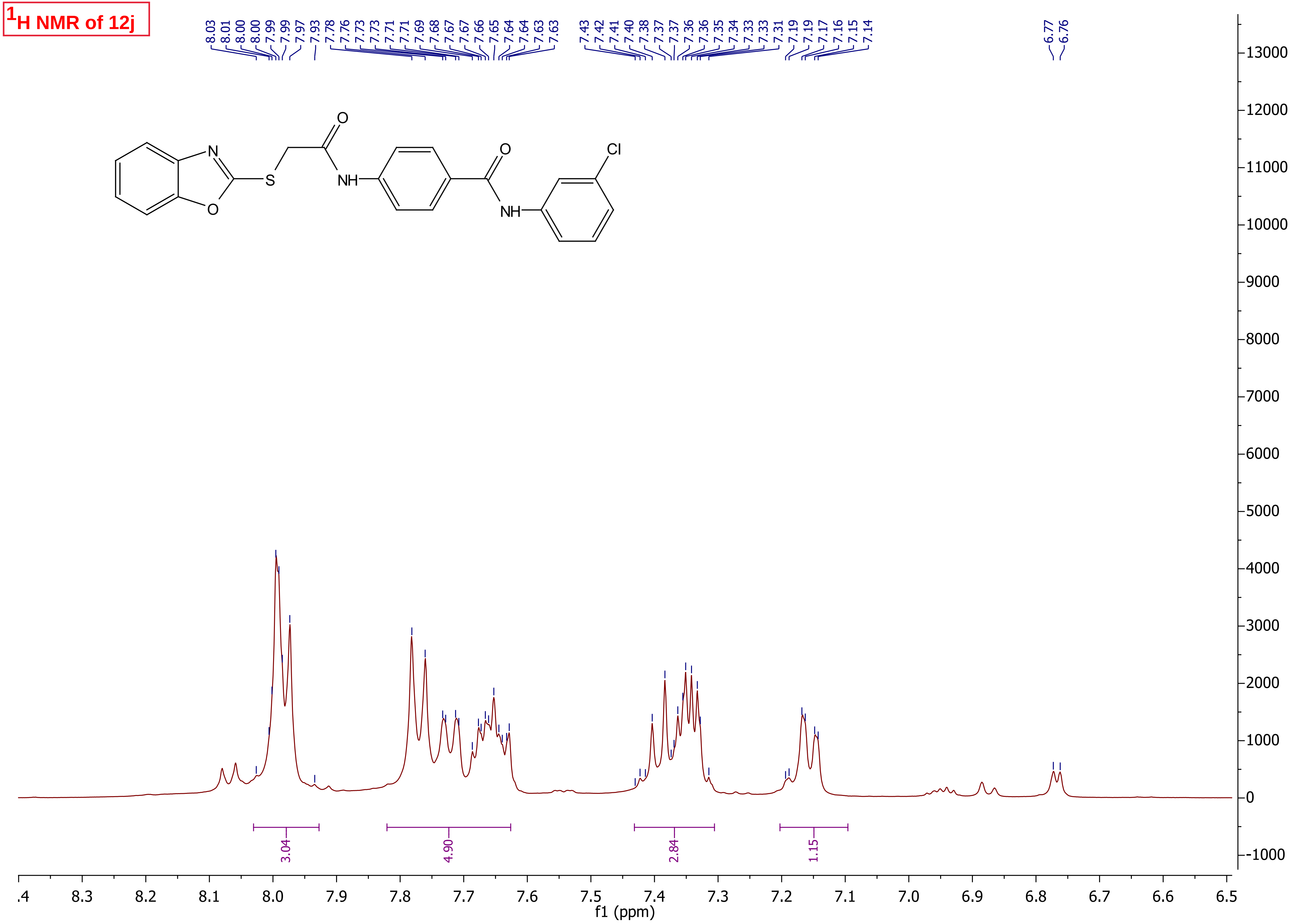

8.03 8.01 8.00 8.00 7.99 7.99 7.97 7.93 7.78 7.76 7.73 7.73 7.71 7.71 7.69 7.68 7.67 7.67 7.66 7.65 7.64 7.64 7.63 7.63
7.43 7.42 7.41 7.40 7.38 7.37 7.37 7.36 7.36 7.35 7.34 7.33 7.33 7.31 7.19 7.19 7.17 7.16 7.15 7.14
6.77 6.76
O
N
S
NH
O
Cl
O
NH
3.04
4.90
2.84
1.15
f1 (ppm)

^{1}H NMR of 12j

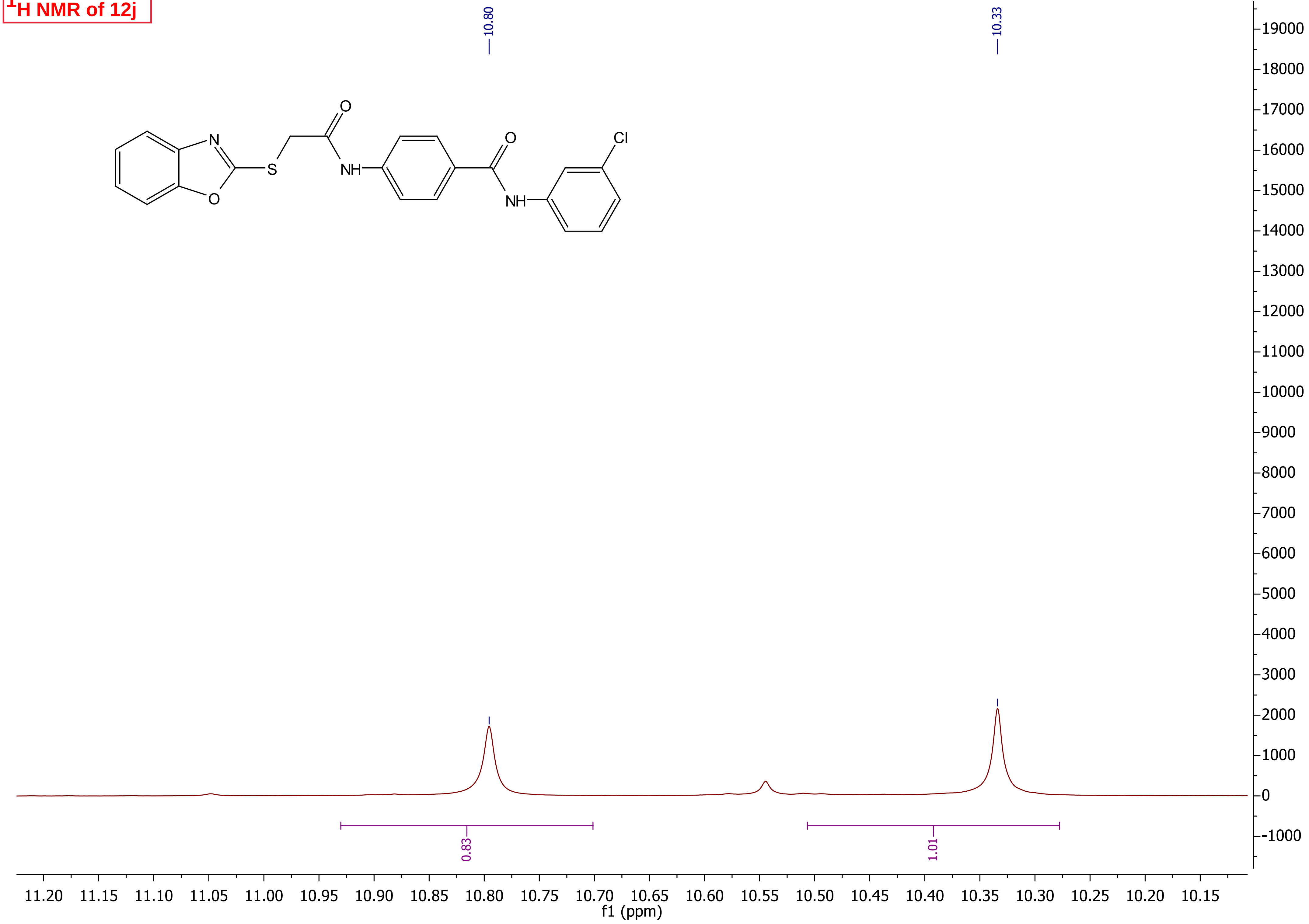

13C NMR of 12j

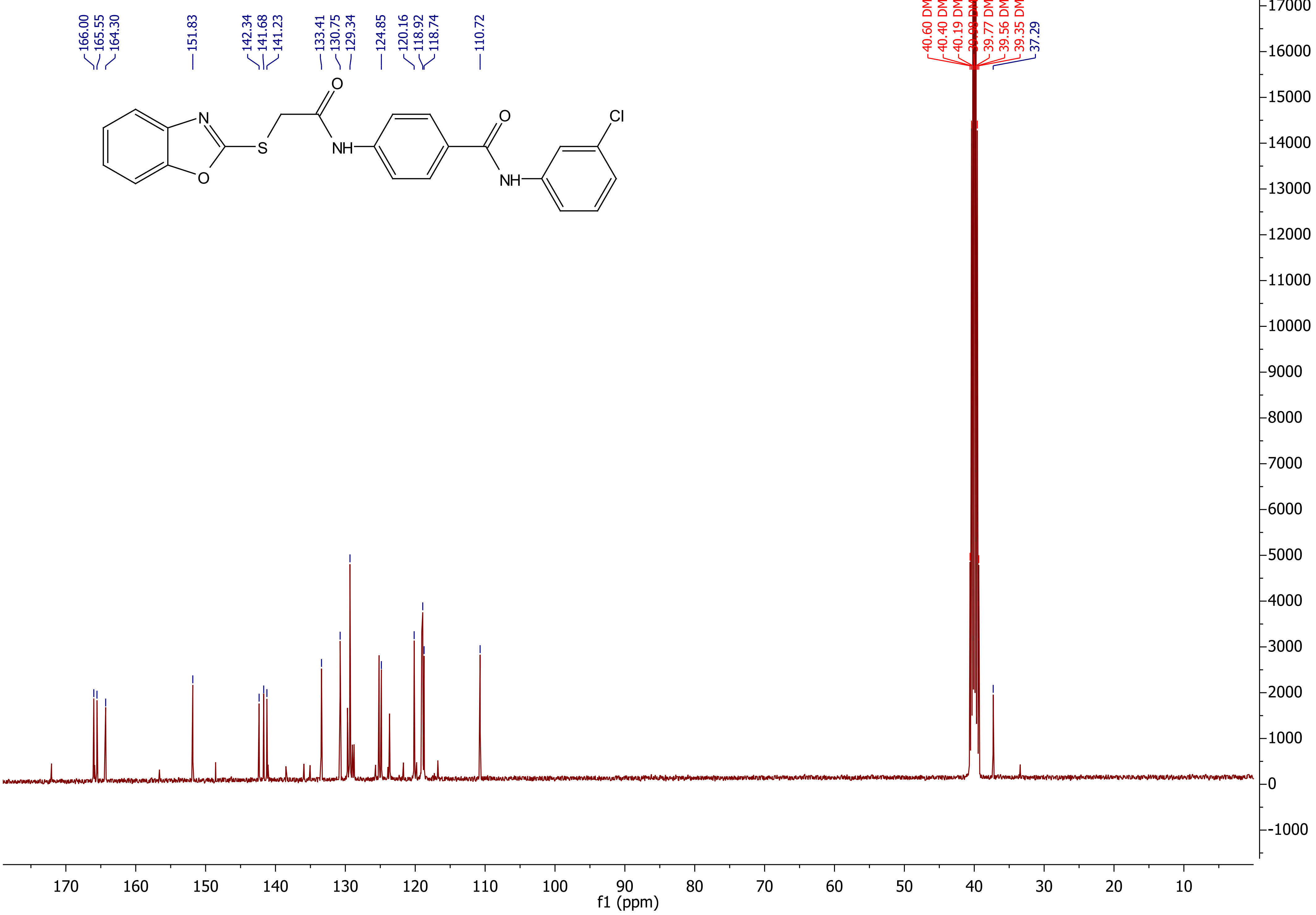

^{13}C NMR of 12j

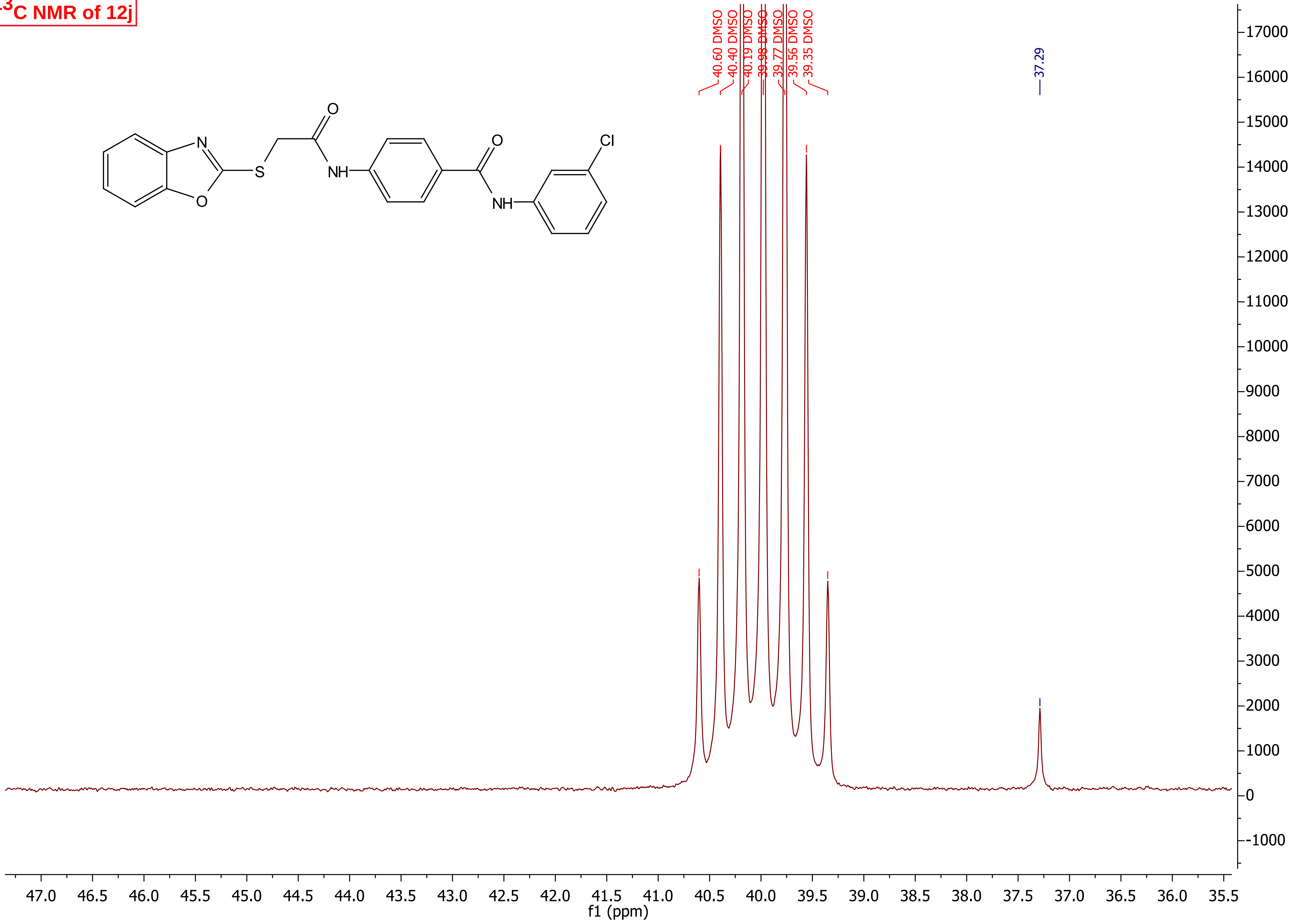

^{13}C NMR of 12j

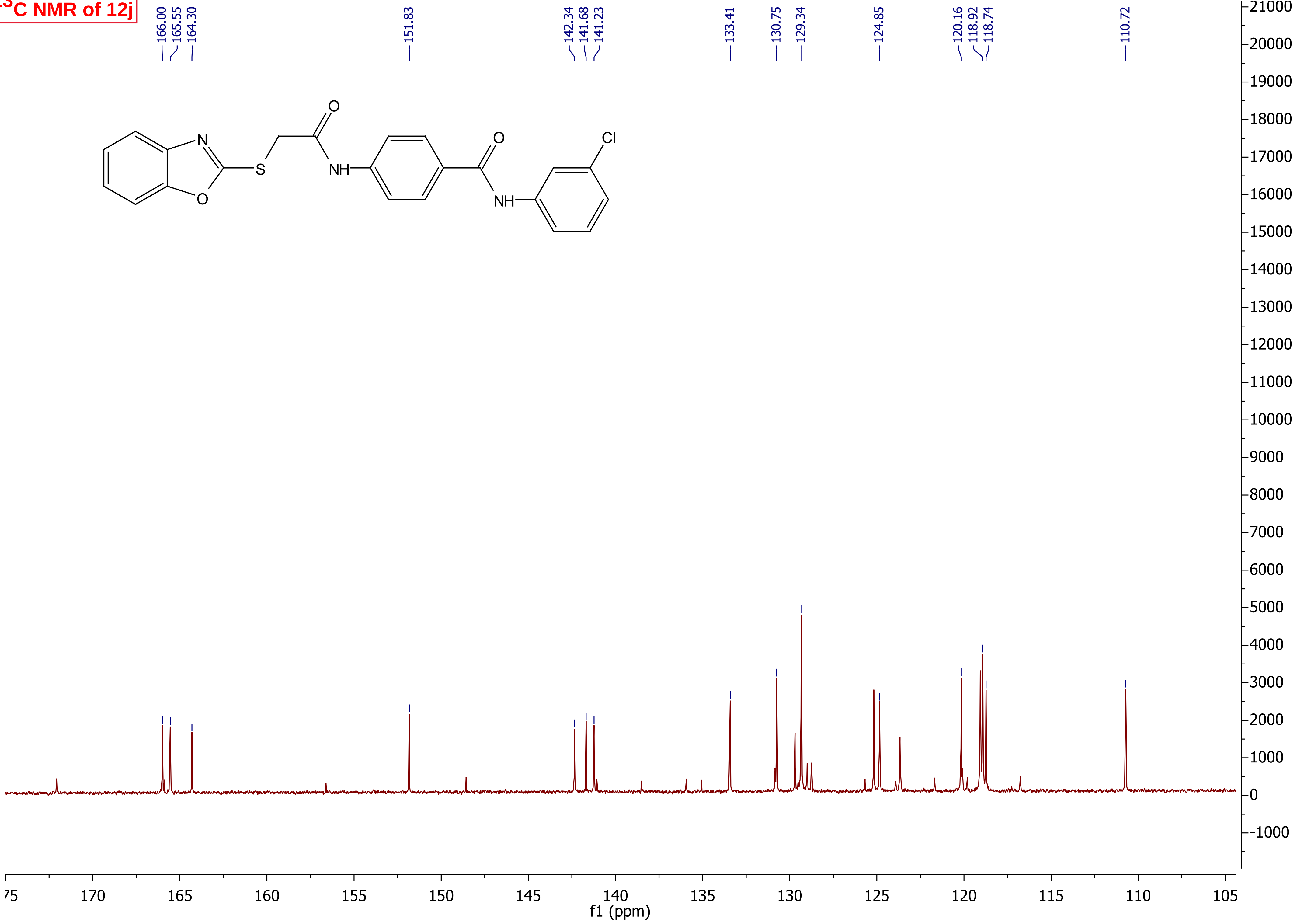

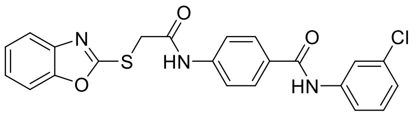

Mass spec. of 12j

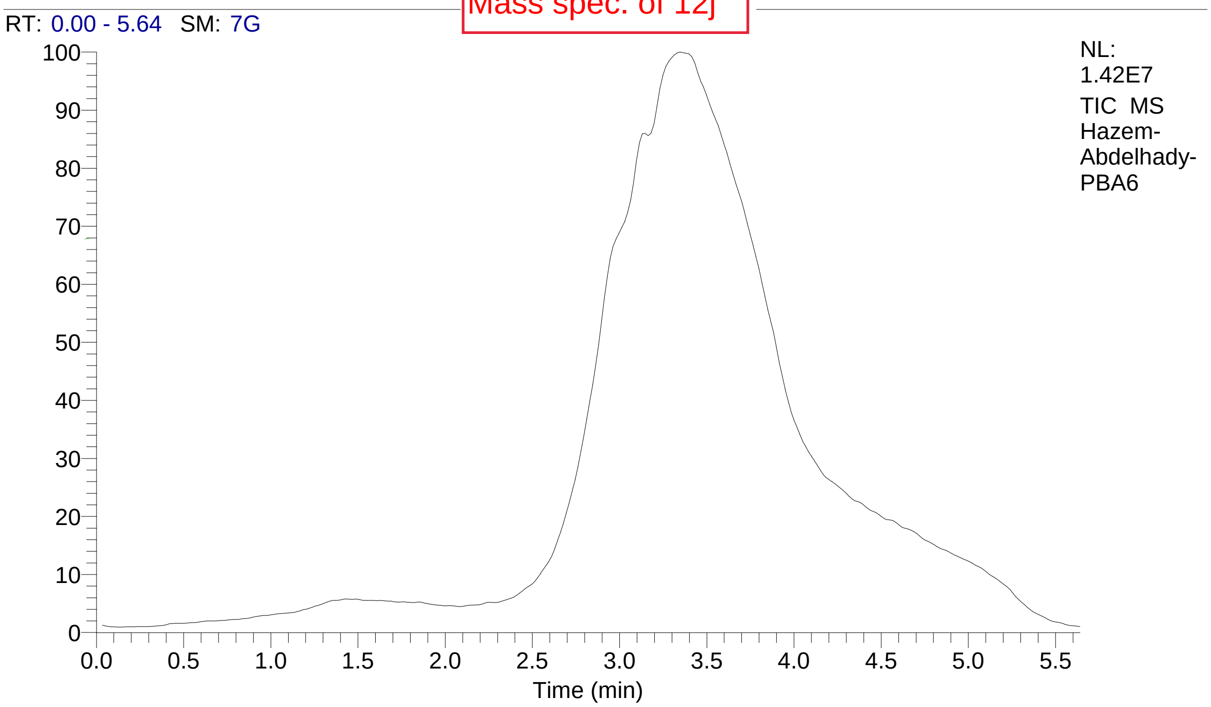

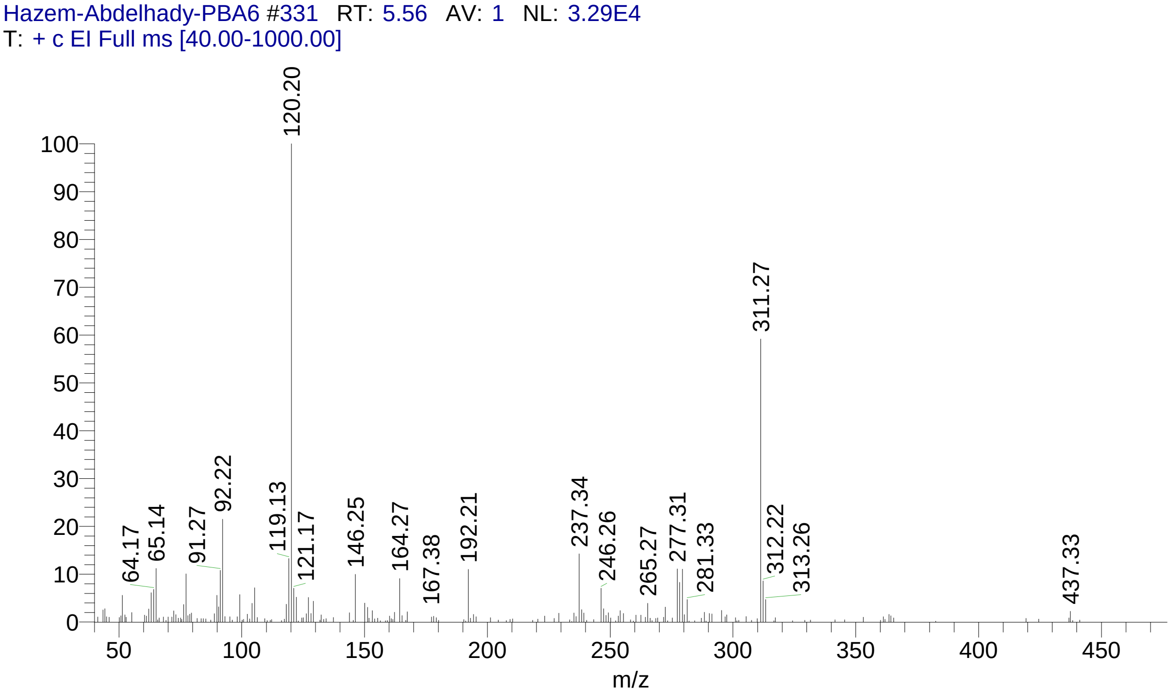

IR Of Comp. 12k

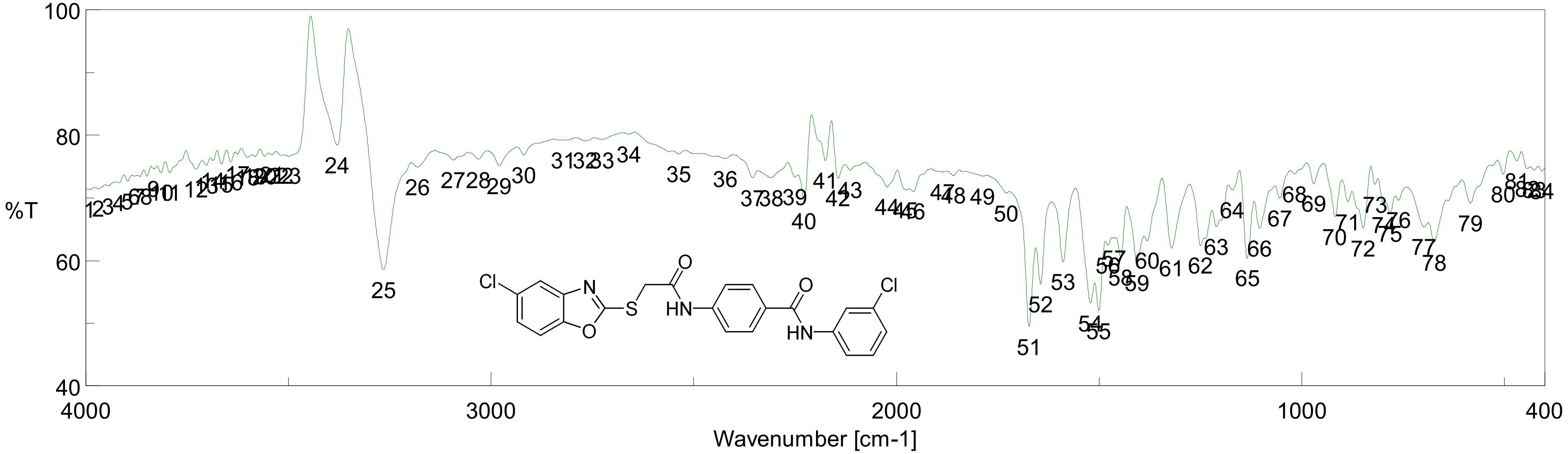

[Result of Peak Picking]

No.	Position	Intensity	No.	Position	Intensity	No.	Position	Intensity	No.	Position	Intensity
1	3990.96	71.294	2	3968.78	71.4774	3	3944.68	71.8733	4	3917.68	72.3984
5	3897.43	72.6204	6	3880.08	73.4161	7	3867.54	73.3792	8	3850.18	73.4256
9	3832.83	74.6036	10	3814.51	73.9844	11	3793.29	73.9895	12	3728.69	74.5611
13	3702.66	75.1883	14	3686.26	75.9155	15	3666.02	75.3447	16	3642.87	75.6807
17	3626.48	76.974	18	3601.41	76.4374	19	3583.09	76.617	20	3558.99	76.5981
21	3541.63	76.8869	22	3517.52	76.7221	23	3499.2	76.6079	24	3379.64	78.3773
25	3265.86	58.4985	26	3181.97	74.8806	27	3093.26	76.0101	28	3031.55	76.1391
29	2980.45	75.0701	30	2919.7	76.8003	31	2822.31	79.2055	32	2768.31	79.0742
33	2726.85	79.273	34	2660.32	80.1573	35	2536.9	76.9775	36	2422.15	76.2309
37	2354.66	73.1455	38	2309.34	73.1563	39	2250.52	73.3083	40	2227.38	69.5185
41	2175.31	75.8916	42	2143.49	73.102	43	2114.56	74.414	44	2022.96	71.7356
45	1977.64	71.2394	46	1958.36	71.0097	47	1886.04	74.0972	48	1859.04	73.5419
49	1786.72	73.4185	50	1728.87	70.7394	51	1672.95	49.4077	52	1644.02	56.2157
53	1589.06	59.7484	54	1520.6	53.1785	55	1500.35	51.9745	56	1478.17	62.4255
57	1462.74	63.5256	58	1446.35	60.4771	59	1405.85	59.5895	60	1380.78	63.1502
61	1321	61.9584	62	1249.65	62.3983	63	1210.11	65.402	64	1170.58	71.2338
65	1133.94	60.3787	66	1104.05	65.1168	67	1053.91	69.8816	68	1017.27	73.7799

[Result of Peak Picking]

No.	Position	Intensity	No.	Position	Intensity	No.	Position	Intensity	No.	Position	Intensity
69	970.019	72.2312	70	917.95	66.9467	71	886.131	69.3421	72	848.525	65.1434
73	818.634	72.1225	74	797.421	68.8782	75	781.993	67.491	76	760.78	69.6227
77	698.105	65.3448	78	673.035	62.8721	79	583.361	69.1268	80	502.366	73.7165
81	468.617	75.861	82	443.547	74.6276	83	425.227	74.4485	84	406.907	74.2537

^{1}H NMR of 12k

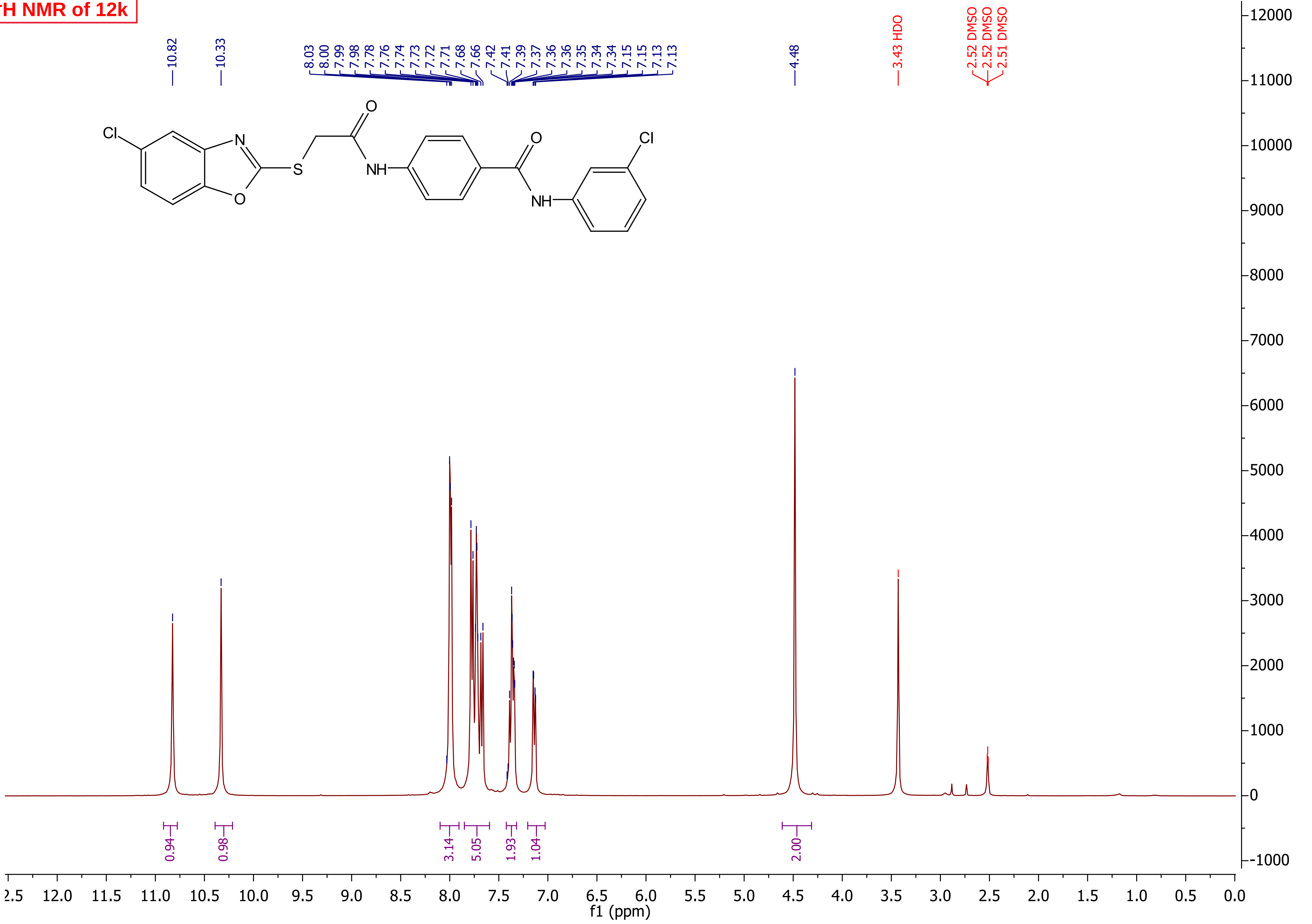
10.82
10.33
8.03
8.00
7.99
7.98
7.78
7.76
7.74
7.73
7.72
7.71
7.68
7.66
7.42
7.41
7.39
7.37
7.36
7.35
7.34
7.34
7.15
7.15
7.13
7.13
4.48
3.43 HDO
2.52 DMSO
2.52 DMSO
2.51 DMSO
Cl
N
O
S
O
NH
O
Cl
NH
0.94
0.98
3.14
5.05
1.93
1.04
2.00
f1 (ppm)

^{1}H NMR of 12k

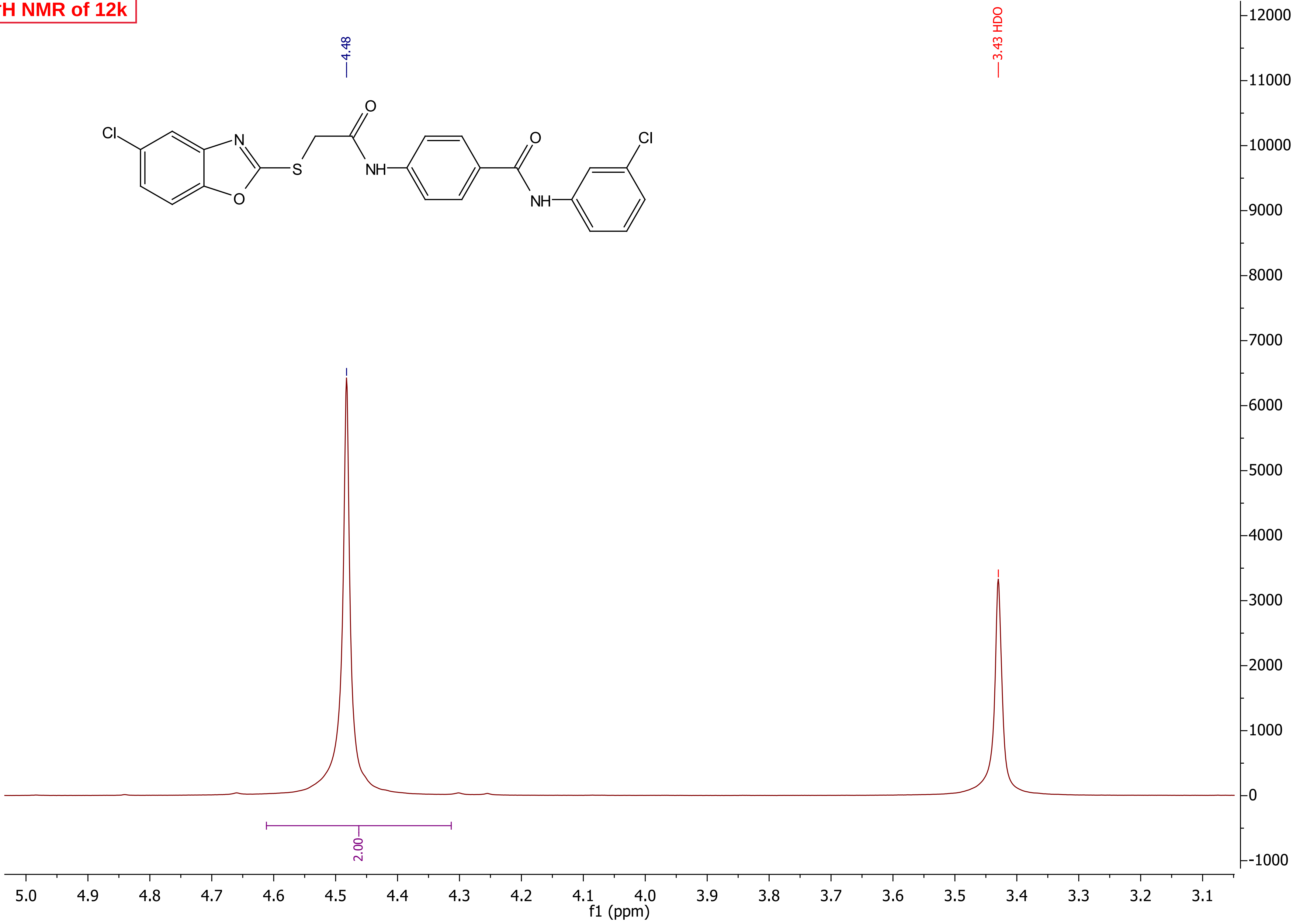
4.48
3.43 HDO
Cl
N
O
S
O
NH
O
Cl
NH
2.00
5.0
4.9
4.8
4.7
4.6
4.5
4.4
4.3
4.2
4.1
4.0
3.9
3.8
3.7
3.6
3.5
3.4
3.3
3.2
3.1
f1 (ppm)
12000
11000
10000
9000
8000
7000
6000
5000
4000
3000
2000
1000
0
-1000

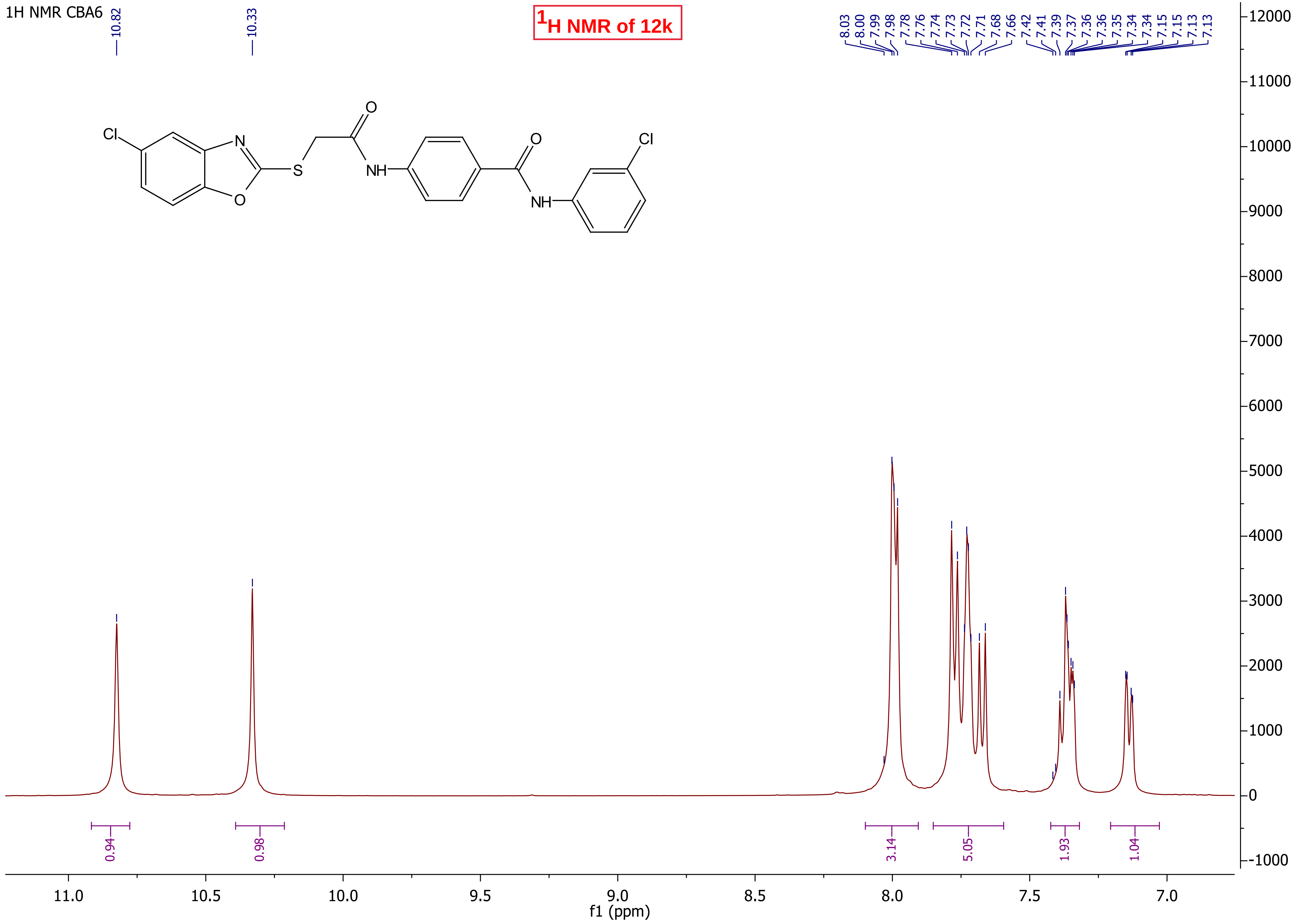
1H NMR CBA6
1H NMR of 12k
10.82
10.33
8.03 8.00 7.99 7.98 7.78 7.76 7.74 7.73 7.72 7.71 7.68 7.66 7.42 7.41 7.39 7.37 7.36 7.36 7.35 7.34 7.34 7.15 7.15 7.13 7.13
Cl
N
S
O
NH
O
Cl
NH
O
0.94
0.98
3.14
5.05
1.93
1.04
f1 (ppm)

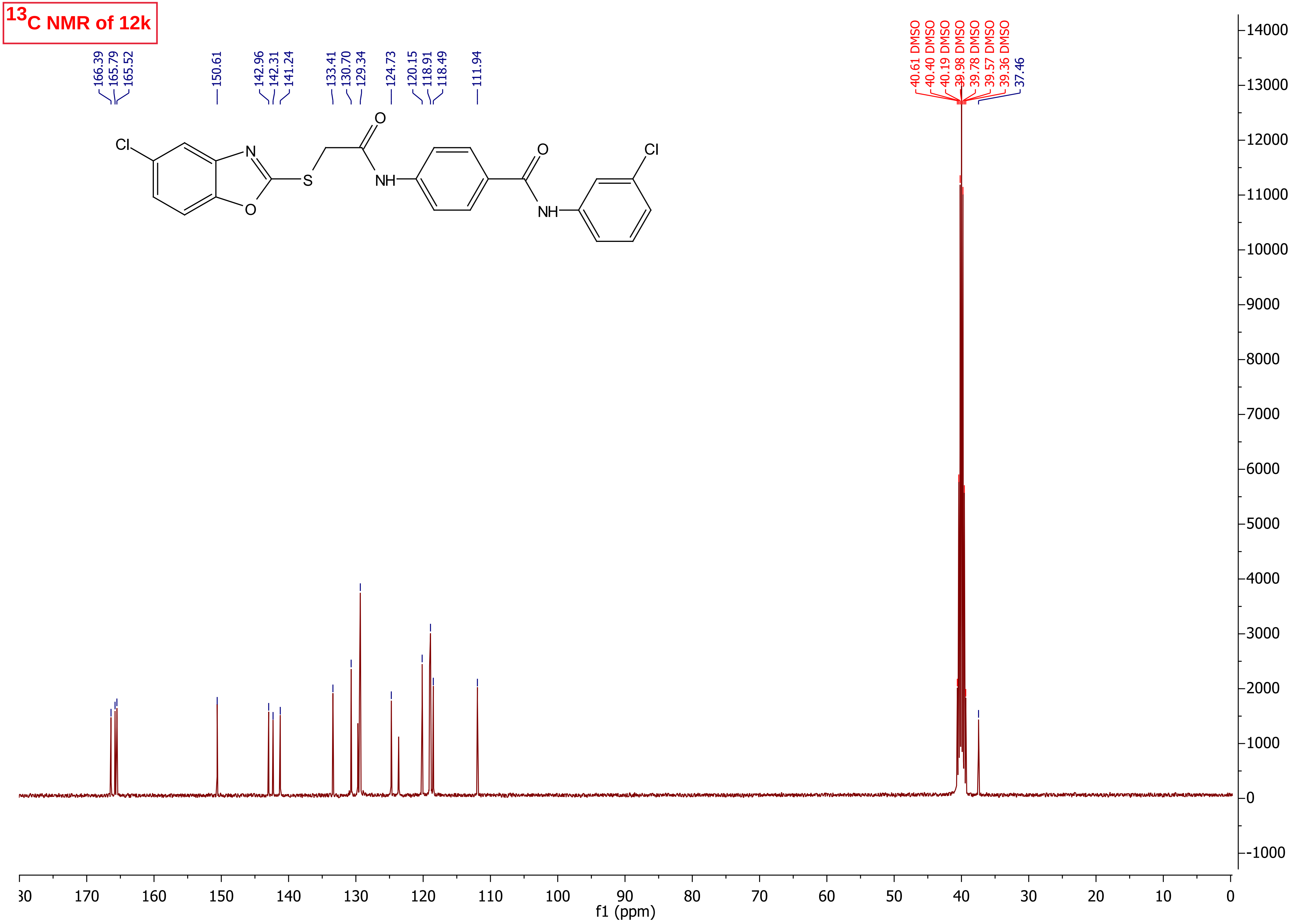

13C NMR of 12k
166.39
165.79
165.52
150.61
142.96
142.31
141.24
133.41
130.70
129.34
124.73
120.15
118.91
118.49
111.94
40.61 DMSO
40.40 DMSO
40.19 DMSO
39.98 DMSO
39.78 DMSO
39.57 DMSO
39.36 DMSO
37.46
Cl
N
S
O
NH
O
O
Cl
NH
f1 (ppm)

13C NMR of 12k

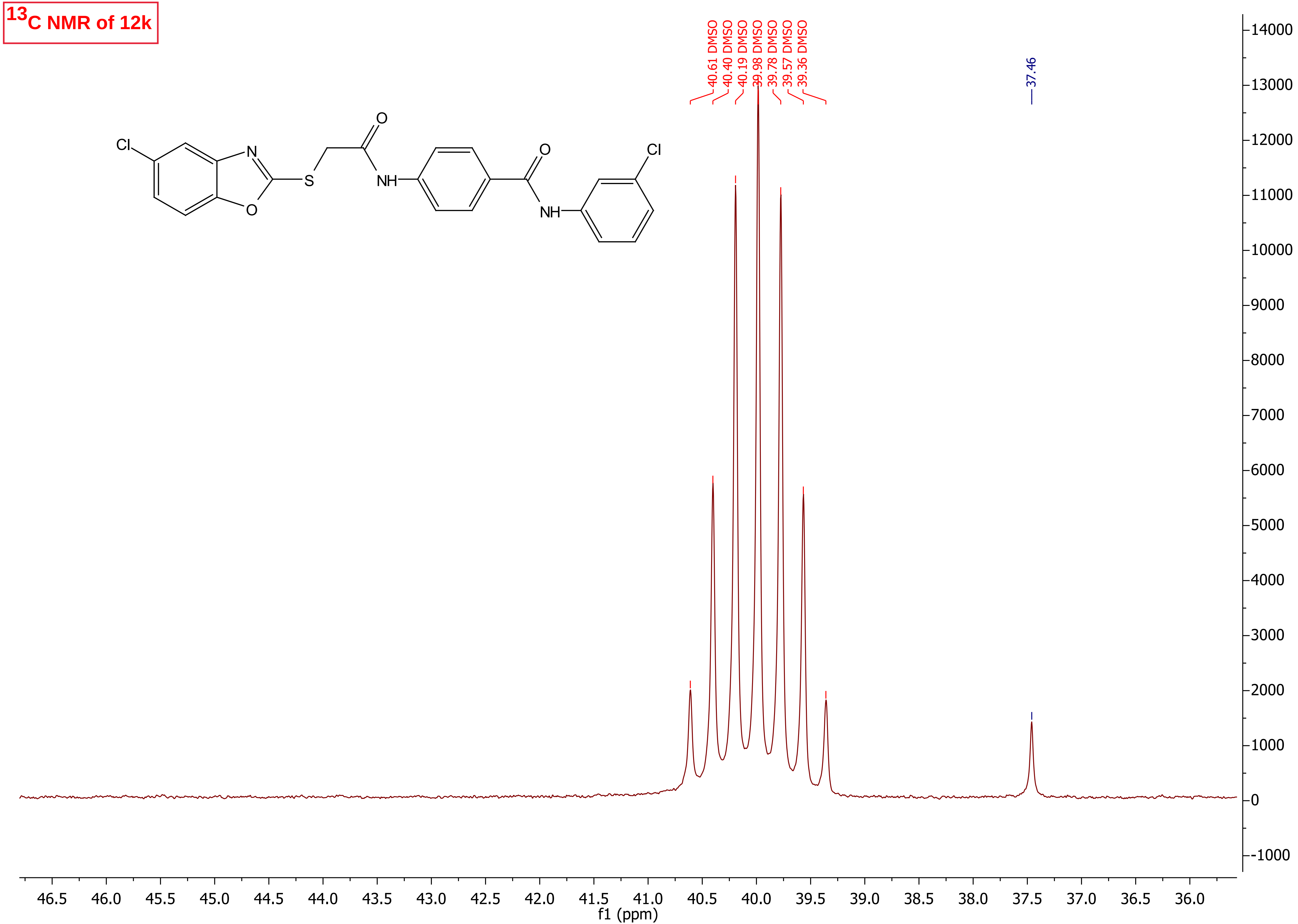

13C NMR of 12k

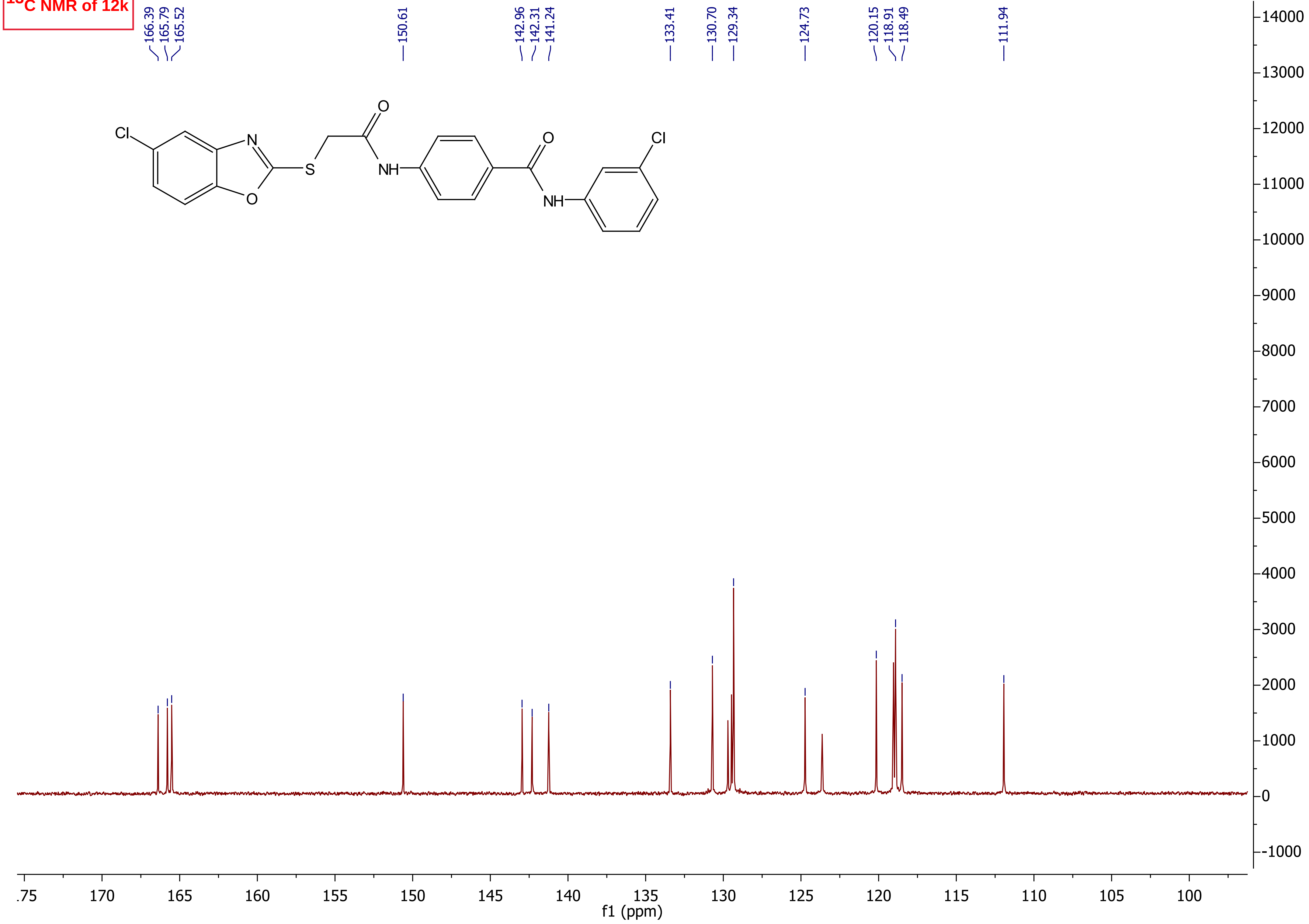

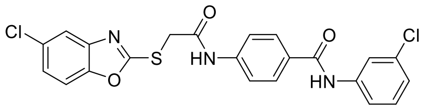

Mass spec. of 12k

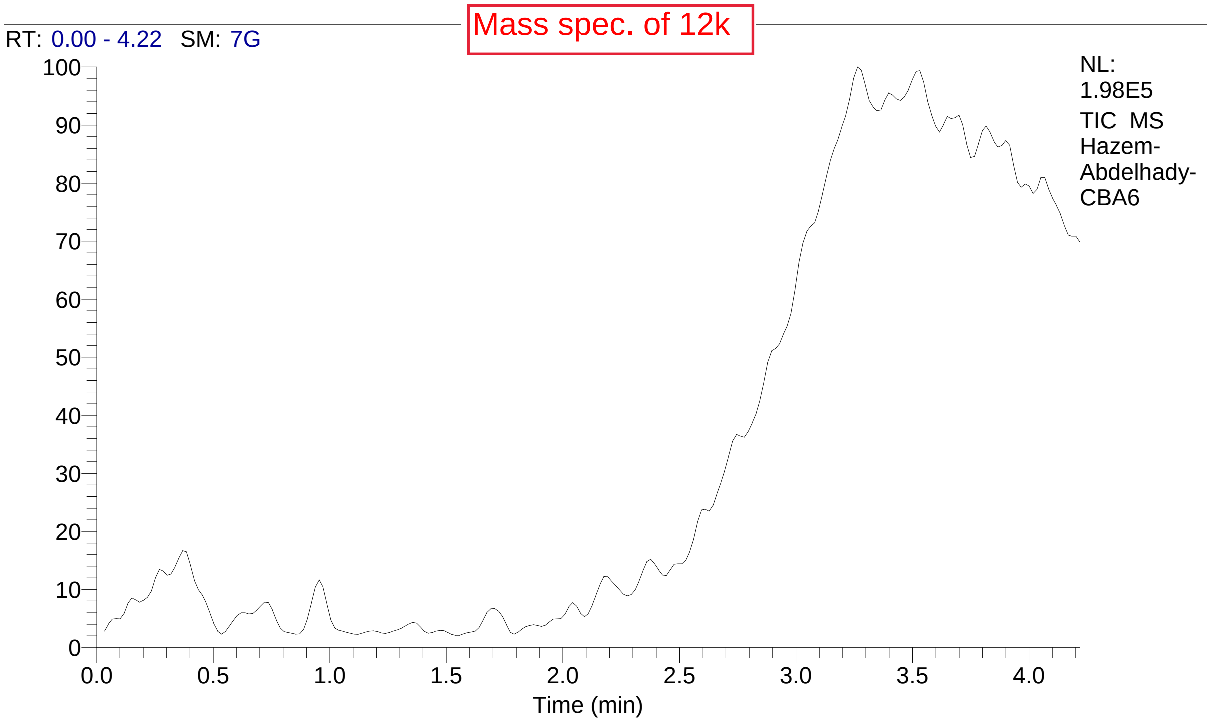

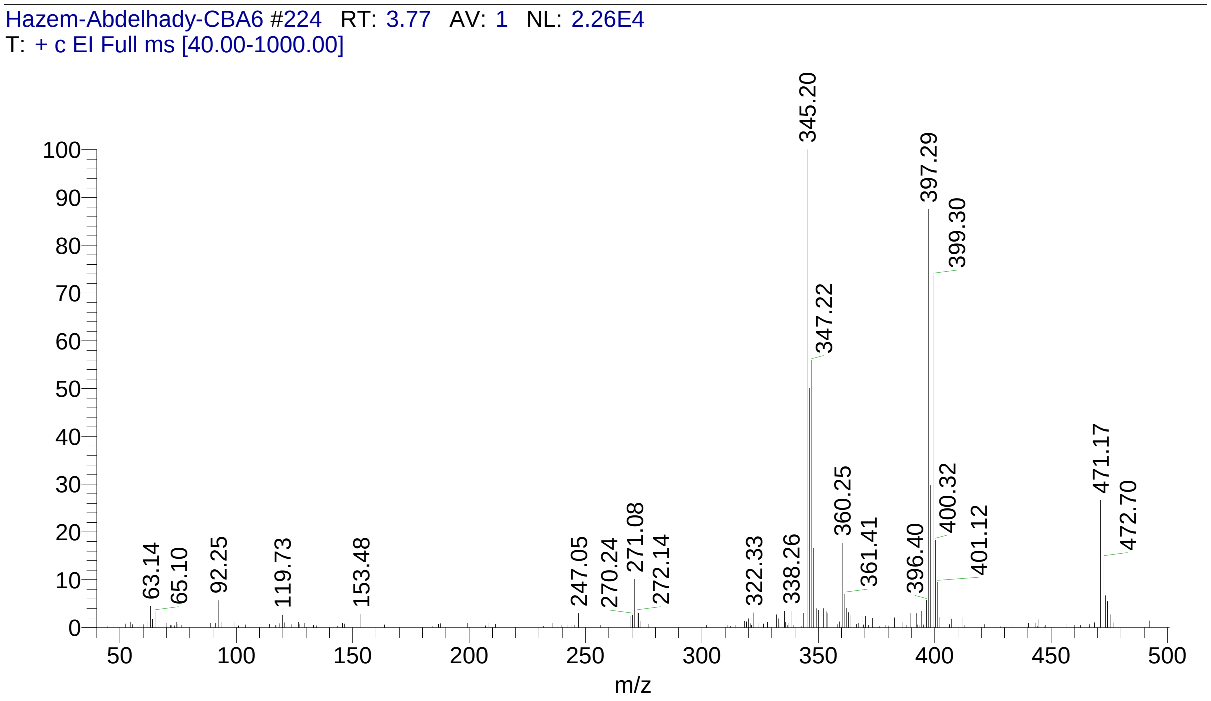

IR Of Comp. 12l

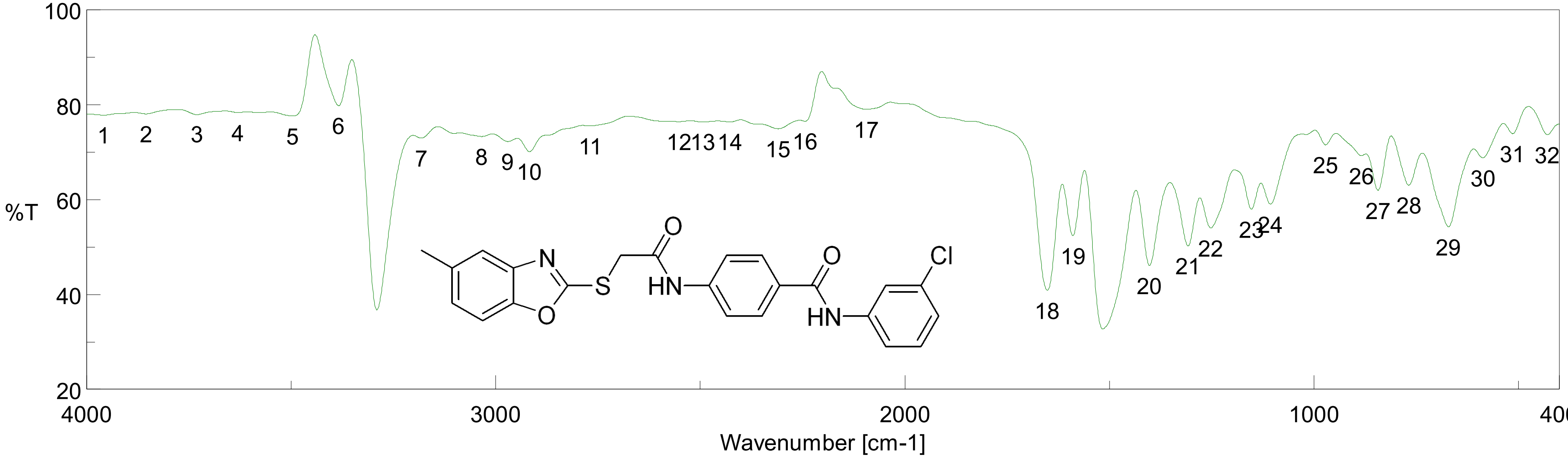

[Result of Peak Picking]

No.	Position	Intensity	No.	Position	Intensity	No.	Position	Intensity	No.	Position	Intensity
1	3958.18	77.7682	2	3855.01	78.0284	3	3730.62	77.8773	4	3630.34	78.3132
5	3496.31	77.6183	6	3384.46	79.7644	7	3181.97	72.9196	8	3034.44	73.2628
9	2970.8	72.2057	10	2917.77	70.0713	11	2768.31	75.4914	12	2552.33	76.3884
13	2493.51	76.2938	14	2427.94	76.3189	15	2310.3	74.8923	16	2243.77	76.4835
17	2094.32	79	18	1651.73	40.8537	19	1589.06	52.3312	20	1402	46.0928
21	1307.5	50.2137	22	1251.58	53.9942	23	1152.26	57.9257	24	1105.98	59.0099
25	970.983	71.5076	26	883.238	69.2085	27	842.74	61.9167	28	767.53	63.0025
29	671.106	54.239	30	586.254	68.7772	31	513.936	73.8434	32	429.084	73.6242

^{1}H NMR of 12l

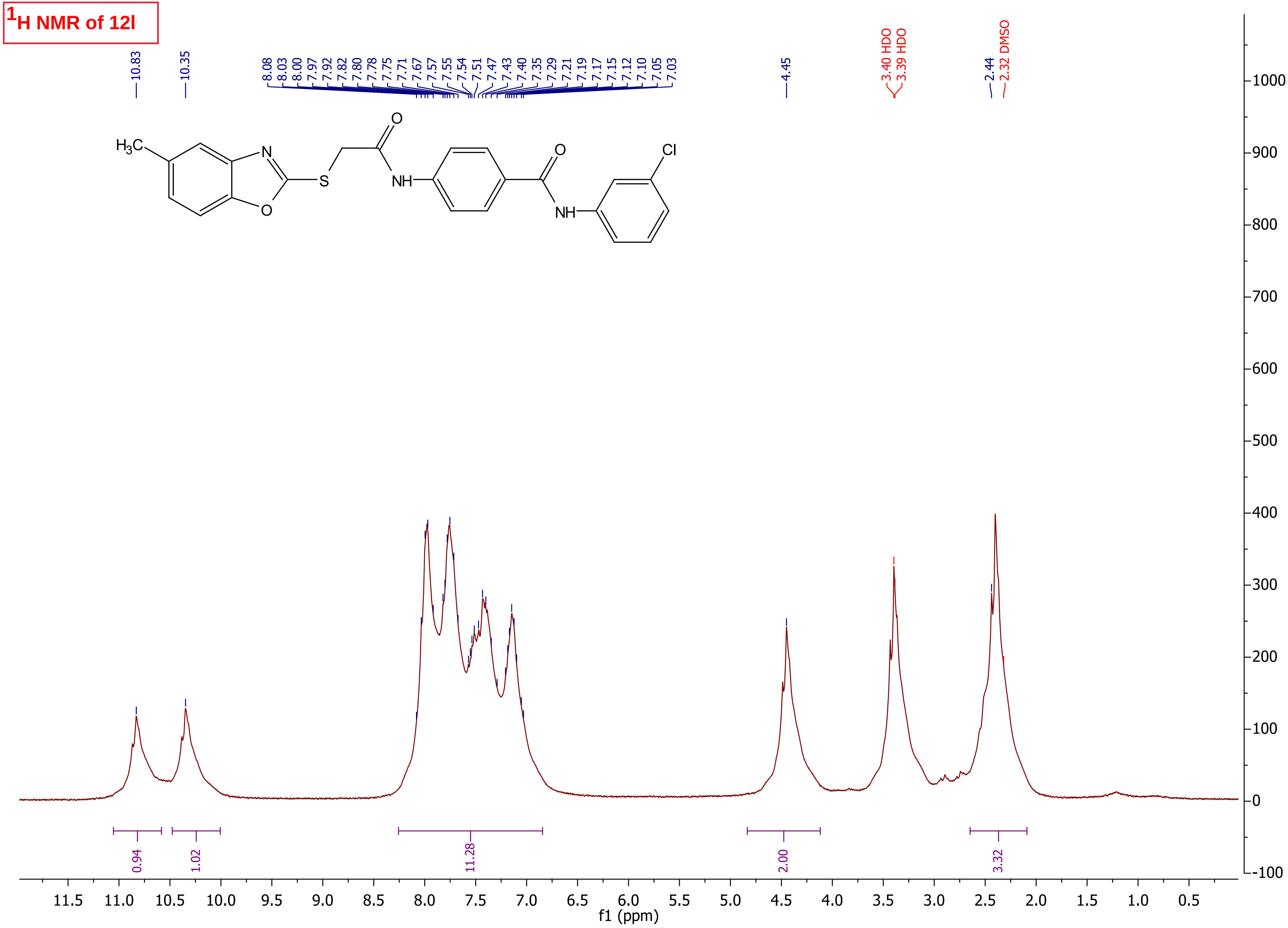

10.83
10.35
8.08
8.03
8.00
7.97
7.92
7.82
7.80
7.78
7.75
7.71
7.67
7.57
7.55
7.54
7.51
7.47
7.43
7.40
7.35
7.29
7.21
7.19
7.17
7.15
7.12
7.10
7.05
7.03
4.45
3.40 HDO
3.39 HDO
2.44
2.32 DMSO
H3C
N
O
S
NH
O
NH
Cl
0.94
1.02
11.28
2.00
3.32
f1 (ppm)

^{1}H NMR of 12l

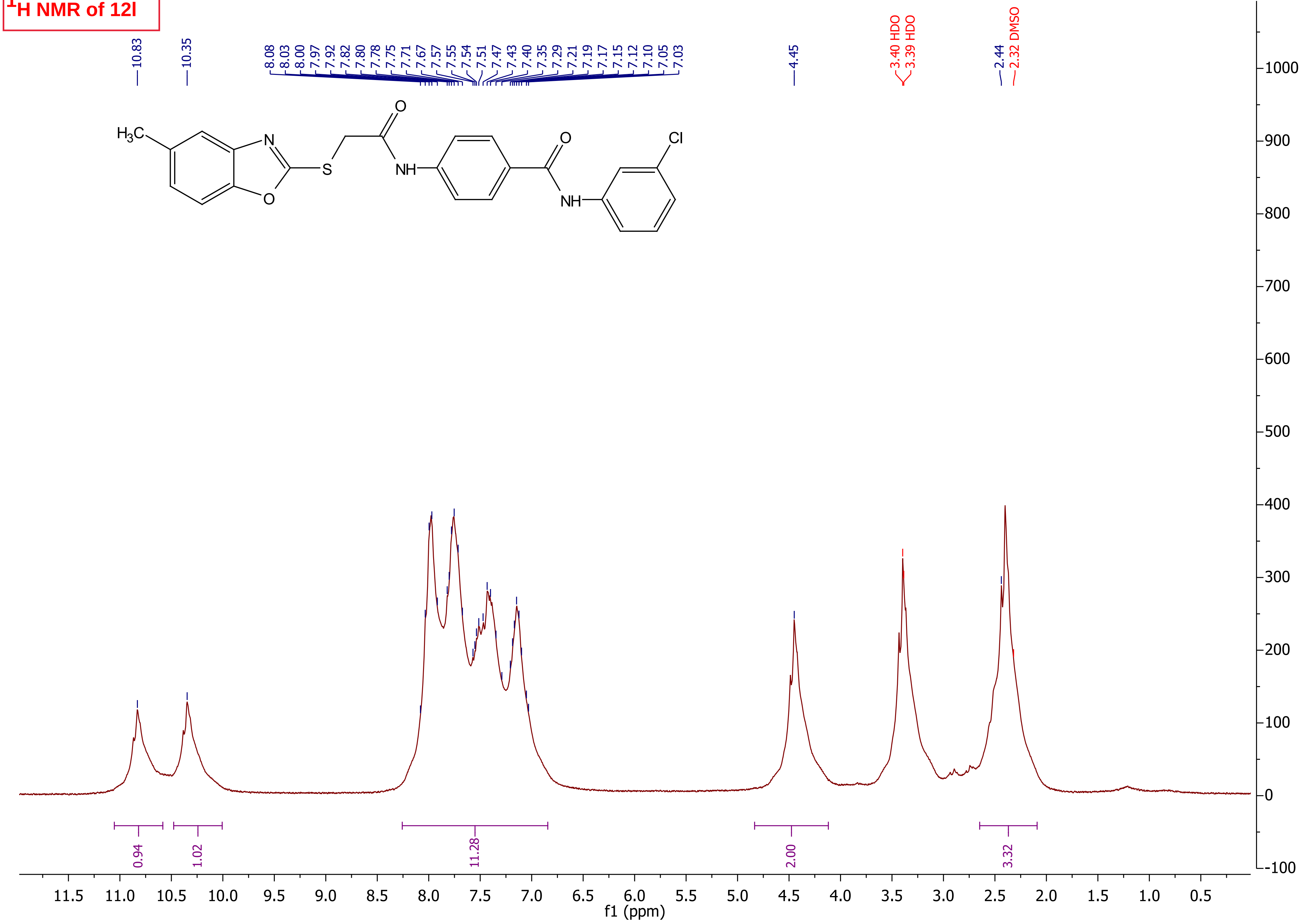

10.83
10.35
8.08
8.03
8.00
7.97
7.92
7.82
7.80
7.78
7.75
7.71
7.67
7.57
7.55
7.54
7.51
7.47
7.43
7.40
7.35
7.29
7.21
7.19
7.17
7.15
7.12
7.10
7.05
7.03
4.45
3.40 HDO
3.39 HDO
2.44
2.32 DMSO
H_3C
N
O
S
NH
O
NH
Cl
0.94
1.02
11.28
2.00
3.32
f1 (ppm)

^{1}H NMR of 12l

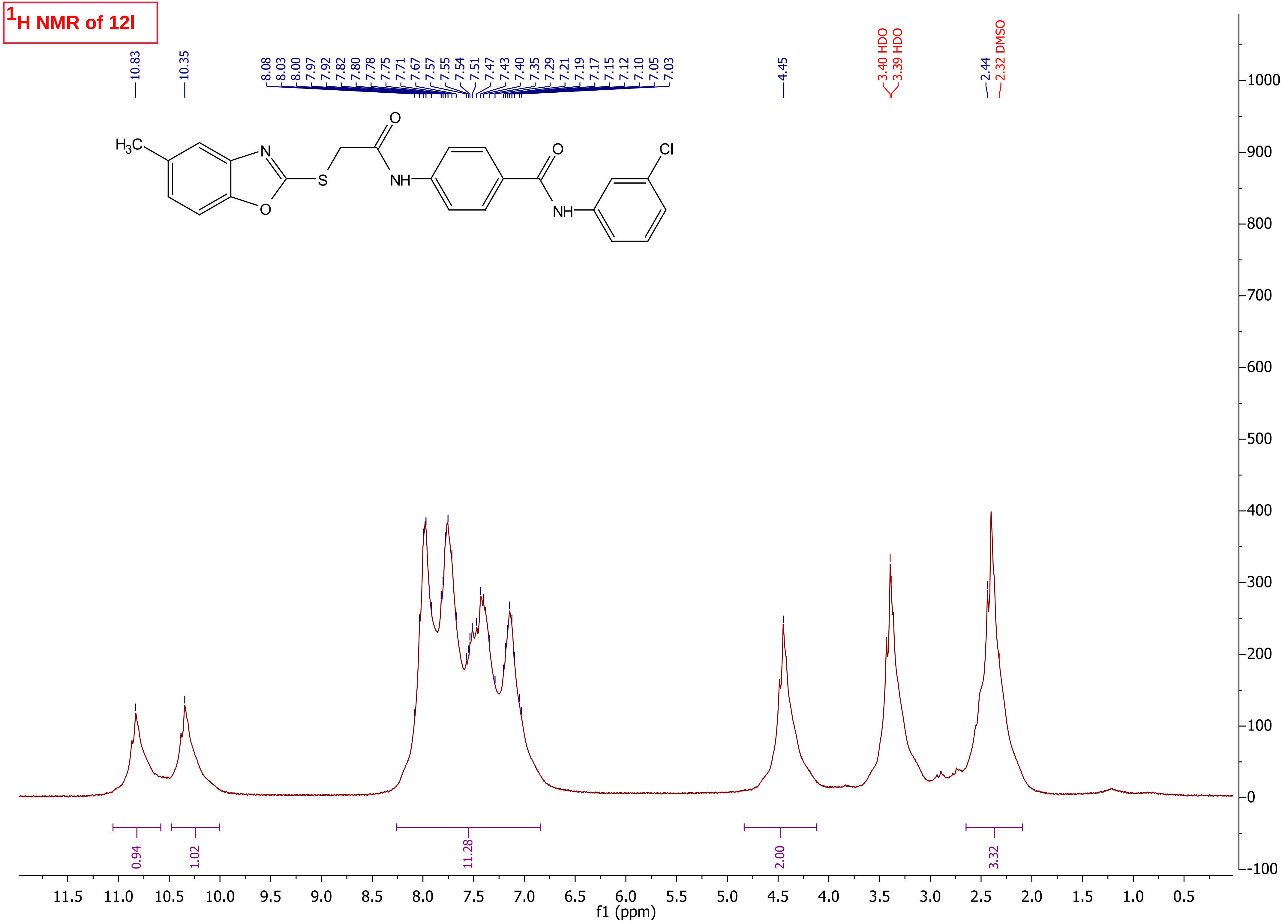
10.83
10.35
8.08 8.03 8.00 7.97 7.92 7.82 7.80 7.78 7.75 7.71 7.67 7.57 7.55 7.54 7.51 7.47 7.43 7.40 7.35 7.29 7.21 7.19 7.17 7.15 7.12 7.10 7.05 7.03
4.45
3.40 HDO
3.39 HDO
2.44
2.32 DMSO
H3C
N
O
S
O
NH
O
NH
Cl
0.94
1.02
11.28
2.00
3.32
f1 (ppm)

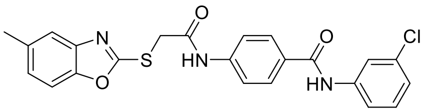

Mass spec. of 12l

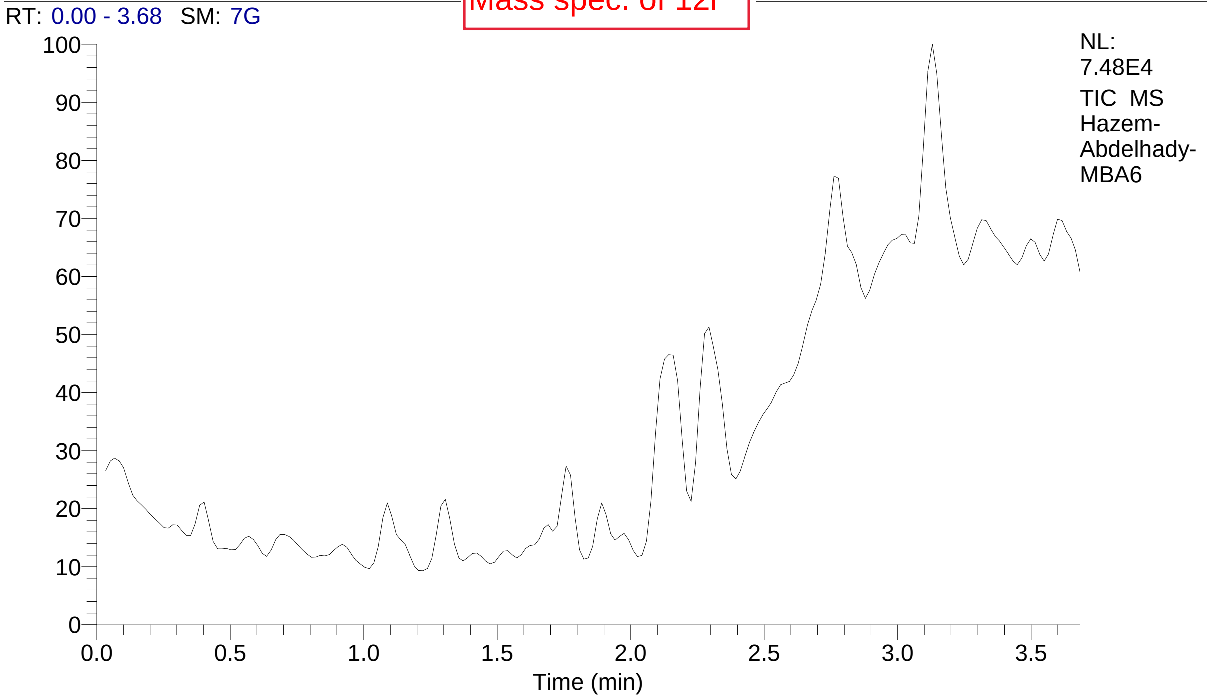

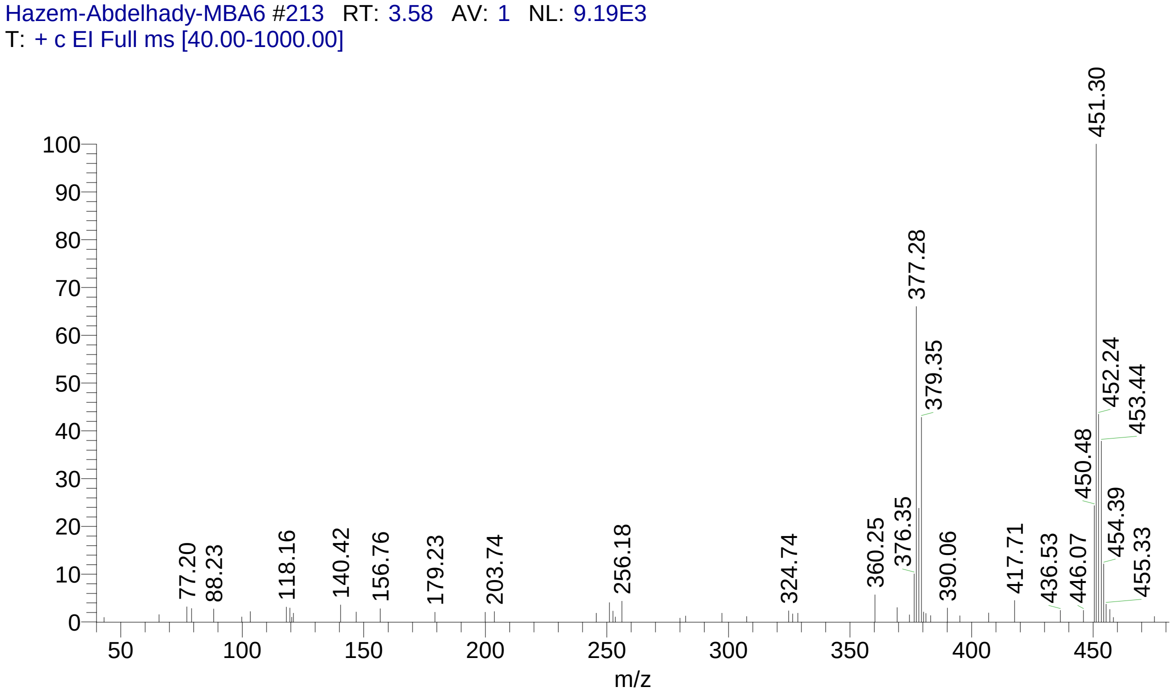

IR Of Comp. 13a

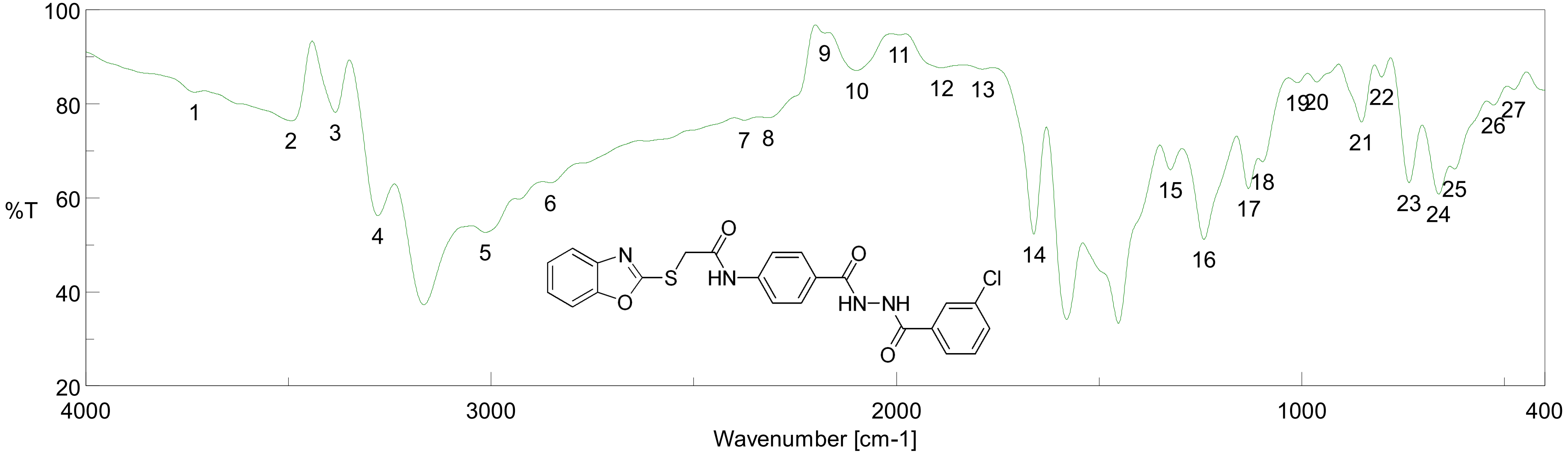

[Result of Peak Picking]

No.	Position	Intensity	No.	Position	Intensity	No.	Position	Intensity	No.	Position	Intensity
1	3731.58	82.4289	2	3493.42	76.3569	3	3384.46	78.15	4	3279.36	56.1766
5	3014.19	52.6266	6	2853.17	63.1626	7	2374.91	76.4734	8	2315.12	77.024
9	2176.27	95.0101	10	2098.17	87.0362	11	1992.11	94.6323	12	1888.93	87.6181
13	1787.69	87.3307	14	1660.41	52.2607	15	1323.89	65.9342	16	1240.97	51.1355
17	1131.05	61.9383	18	1096.33	67.6558	19	1010.52	84.4586	20	963.269	84.6237
21	852.382	76.1033	22	802.242	85.6725	23	734.746	63.1955	24	661.464	60.7727
25	621.931	66.1394	26	526.471	79.7055	27	476.331	83.0287			

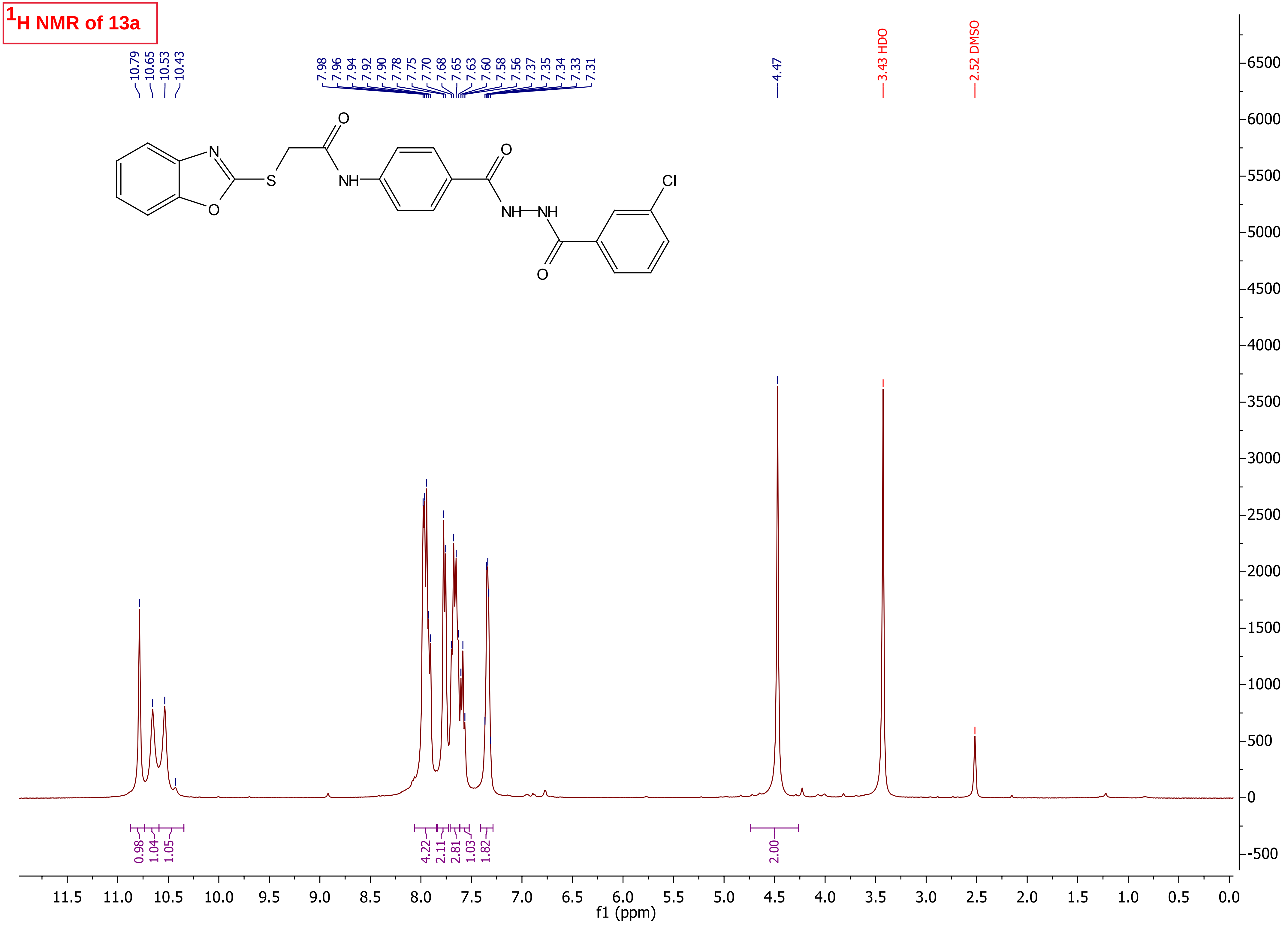

1H NMR of 13a
10.79
10.65
10.53
10.43
7.98
7.96
7.94
7.92
7.90
7.78
7.75
7.70
7.68
7.65
7.63
7.60
7.58
7.56
7.37
7.35
7.34
7.33
7.31
4.47
3.43 HDO
2.52 DMSO
0.98
1.04
1.05
4.22
2.11
2.81
1.03
1.82
2.00
f1 (ppm)

^{1}H NMR of 13a

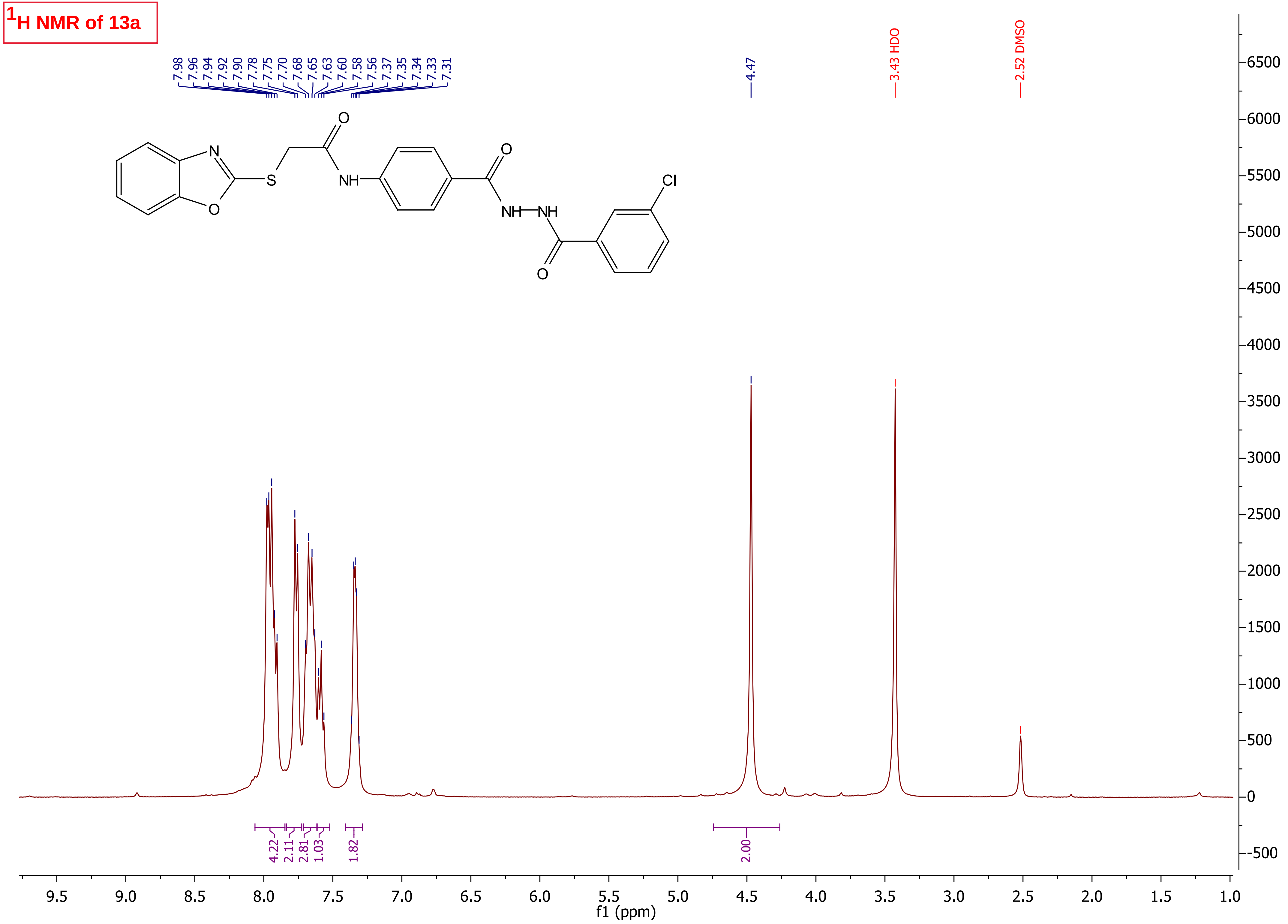

7.98
7.96
7.94
7.92
7.90
7.78
7.75
7.70
7.68
7.65
7.63
7.60
7.58
7.56
7.37
7.35
7.34
7.33
7.31
4.47
3.43 HDO
2.52 DMSO
O
N
S
NH
O
NH
NH
Cl
O
O
4.22
2.11
2.81
1.03
1.82
2.00
f1 (ppm)

^{1}H NMR of 13a

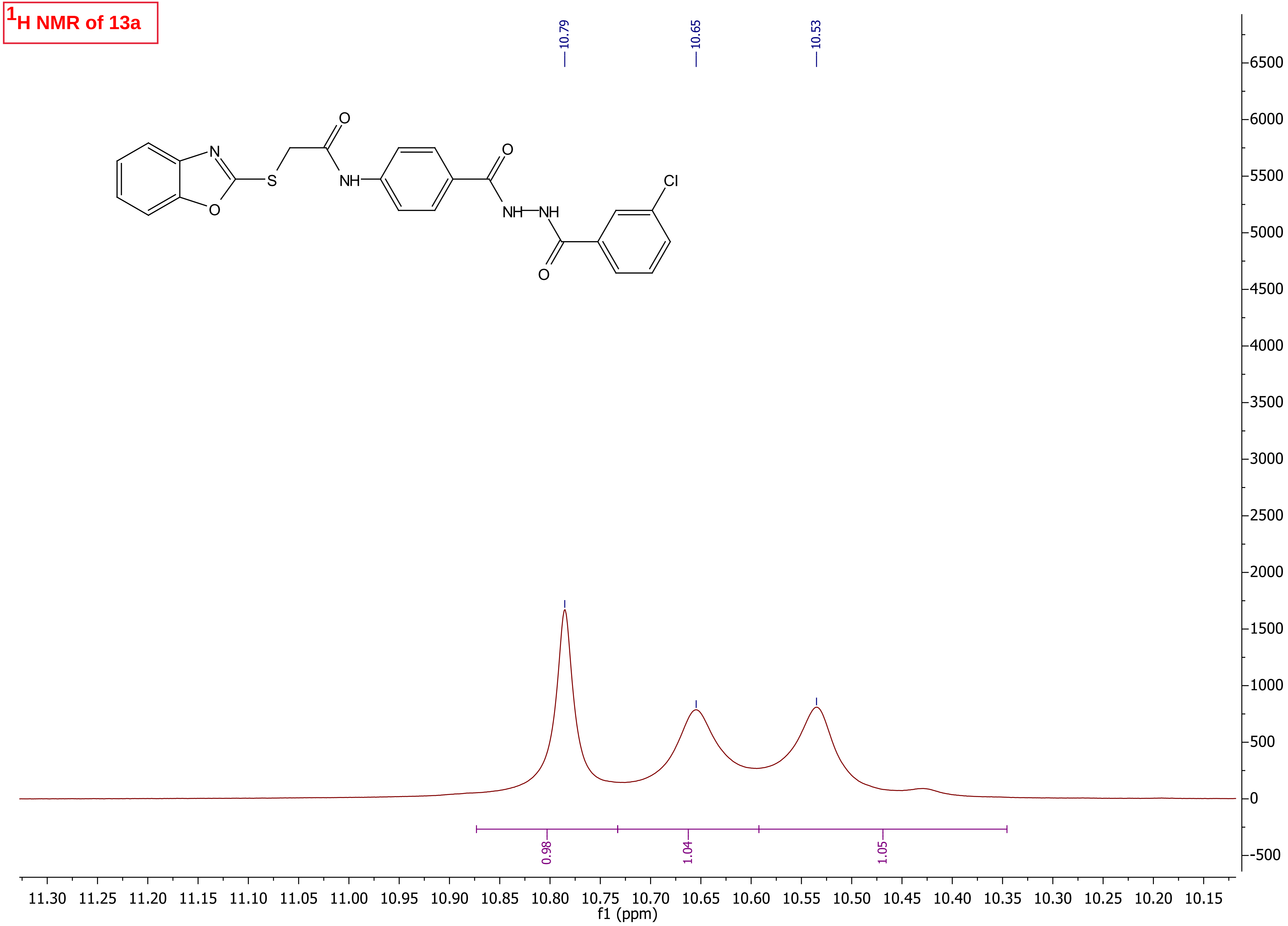

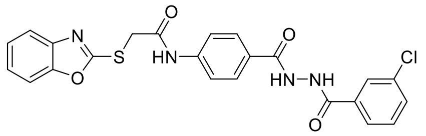

Mass spec. of 13a

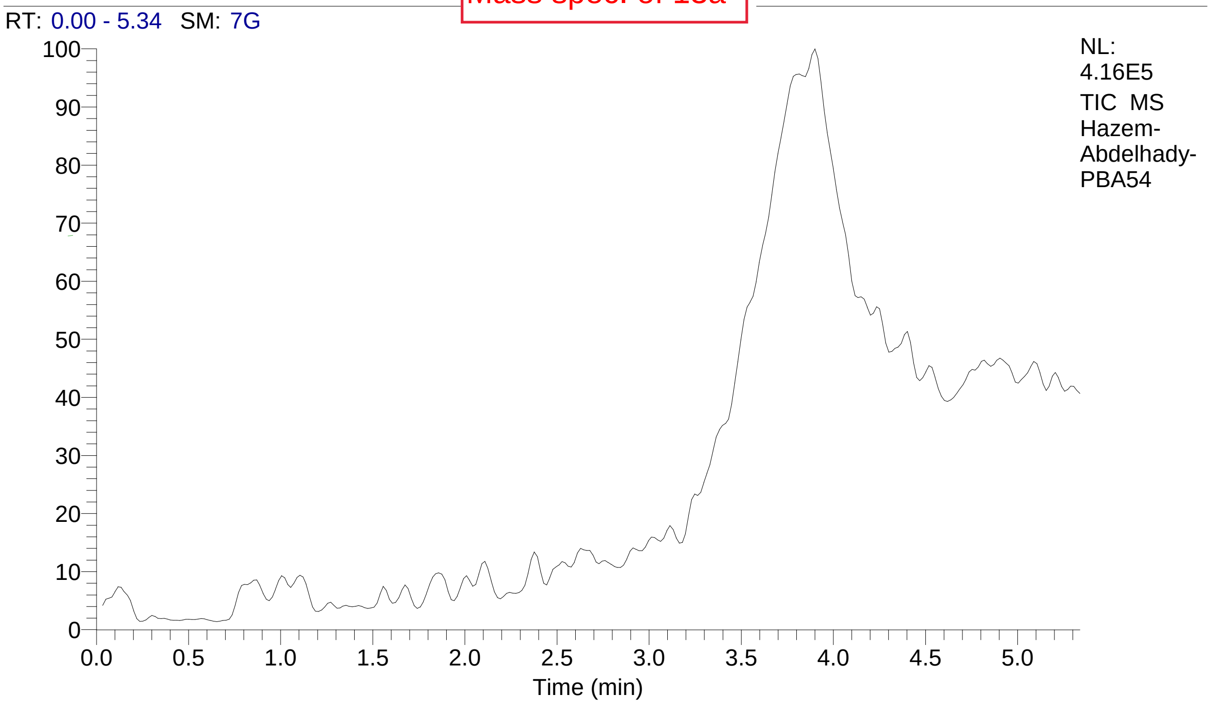

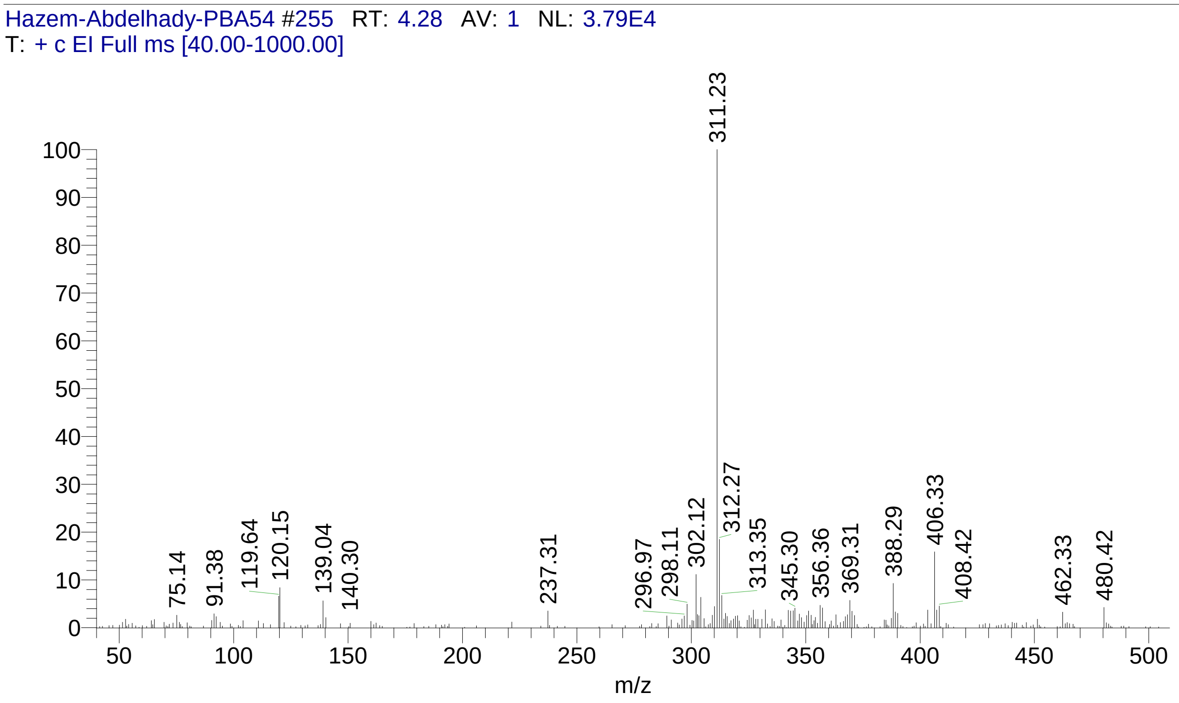

IR Of Comp. 13b

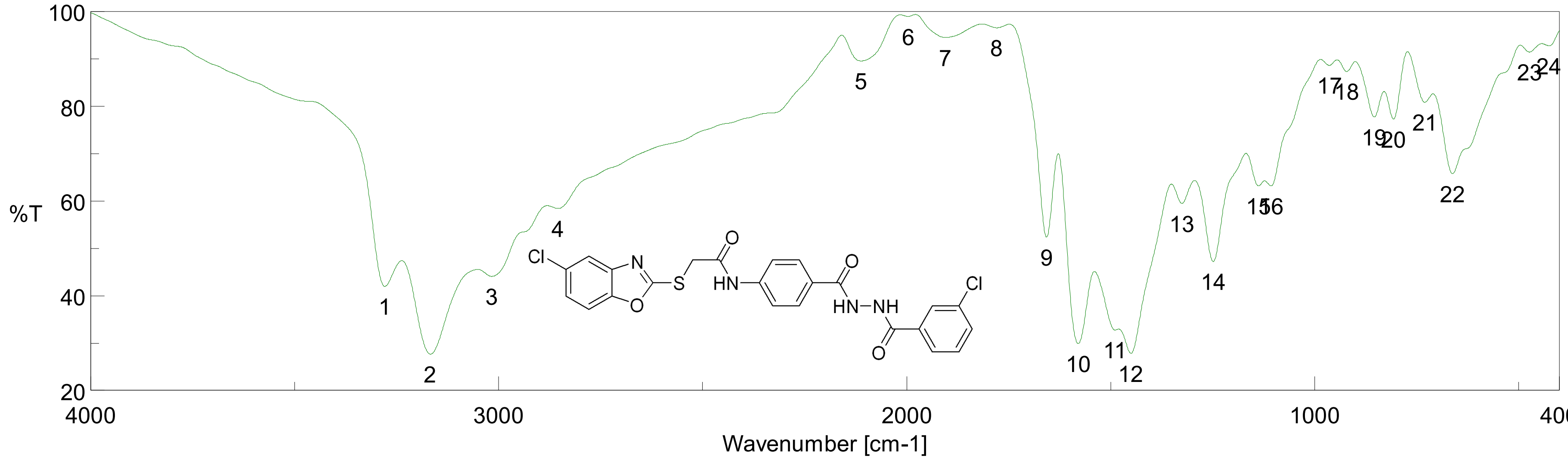

[Result of Peak Picking]

No.	Position	Intensity	No.	Position	Intensity	No.	Position	Intensity	No.	Position	Intensity
1	3279.36	41.9412	2	3167.51	27.6324	3	3017.09	44.0511	4	2855.1	58.3746
5	2111.67	89.5313	6	1995.96	98.9196	7	1904.36	94.4655	8	1779.01	96.5311
9	1656.55	52.3258	10	1579.41	29.8748	11	1488.78	32.7287	12	1450.21	27.805
13	1324.86	59.4584	14	1248.68	47.1907	15	1136.83	63.1437	16	1106.94	63.1547
17	963.269	88.6103	18	920.843	87.3138	19	853.347	77.6958	20	806.099	77.2693
21	729.925	80.8051	22	661.464	65.7331	23	472.474	91.4163	24	425.227	92.7091

1H NMR of 13b

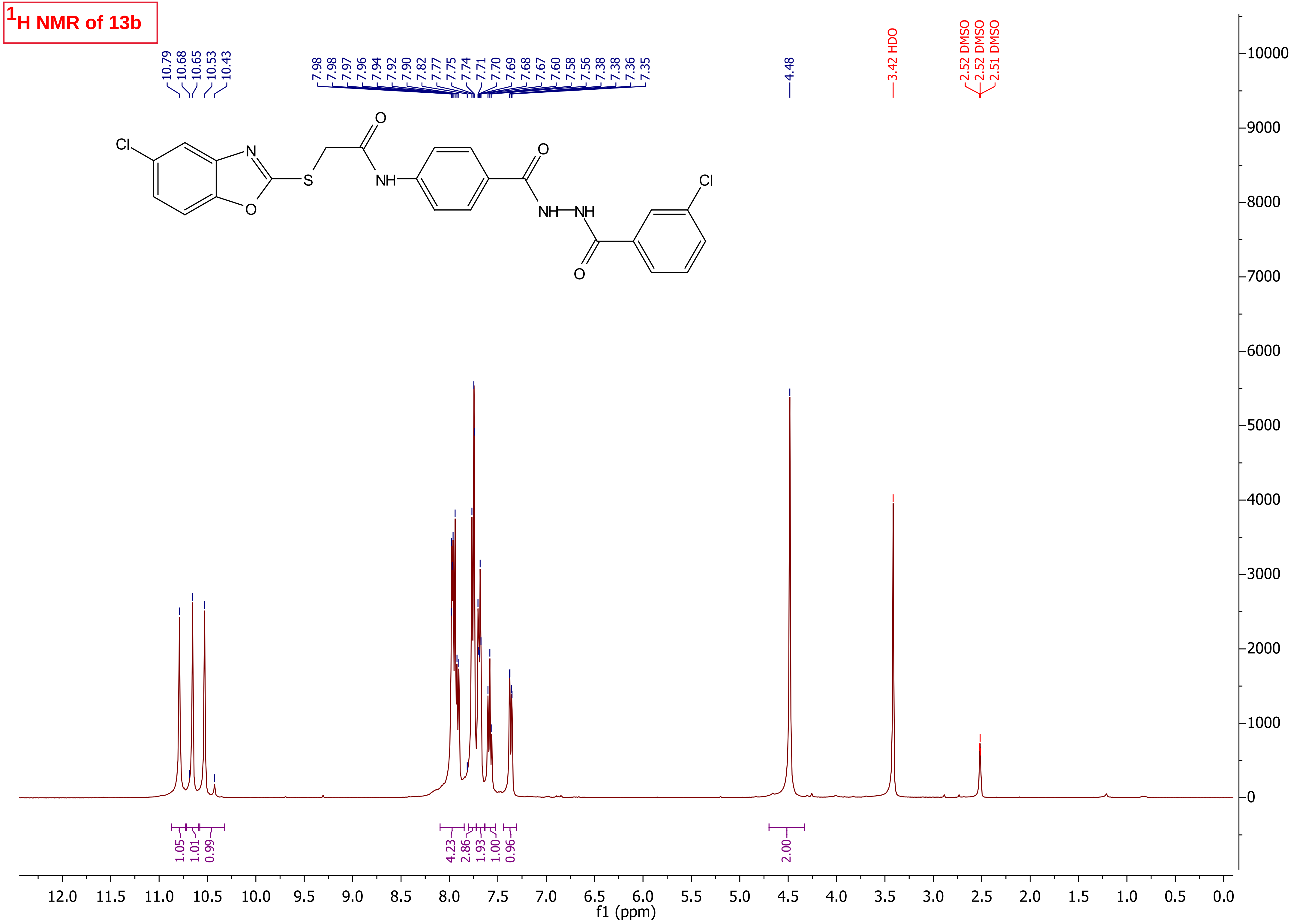

^{1}H NMR of 13b

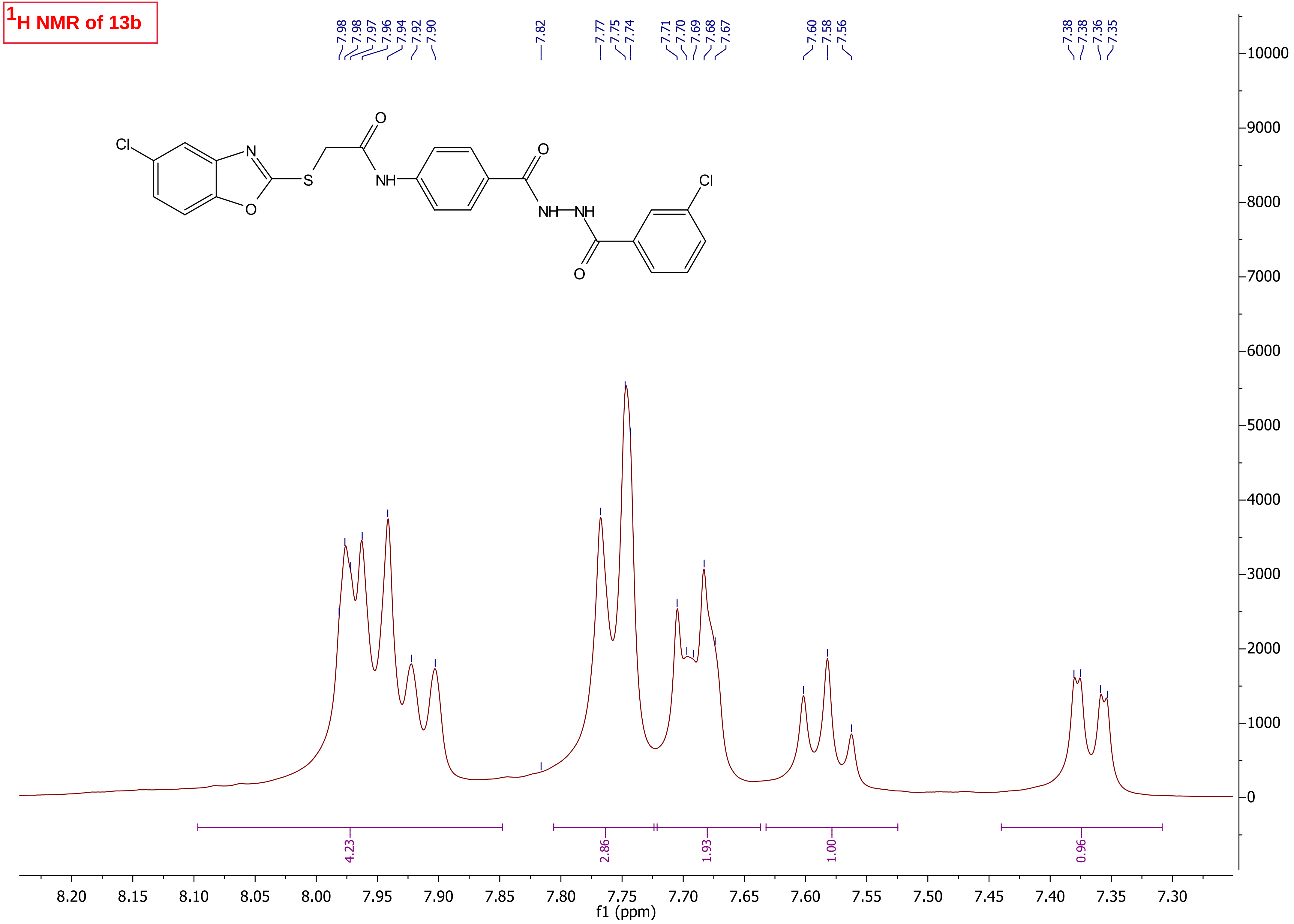
7.98
7.98
7.97
7.96
7.94
7.92
7.90
7.82
7.77
7.75
7.74
7.71
7.70
7.69
7.68
7.67
7.60
7.58
7.56
7.38
7.38
7.36
7.35
Cl
N
O
S
O
NH
O
NH
NH
Cl
O
10000
9000
8000
7000
6000
5000
4000
3000
2000
1000
0
4.23
2.86
1.93
1.00
0.96
8.20
8.15
8.10
8.05
8.00
7.95
7.90
7.85
7.80
7.75
7.70
7.65
7.60
7.55
7.50
7.45
7.40
7.35
7.30
f1 (ppm)

^{1}H NMR of 13b

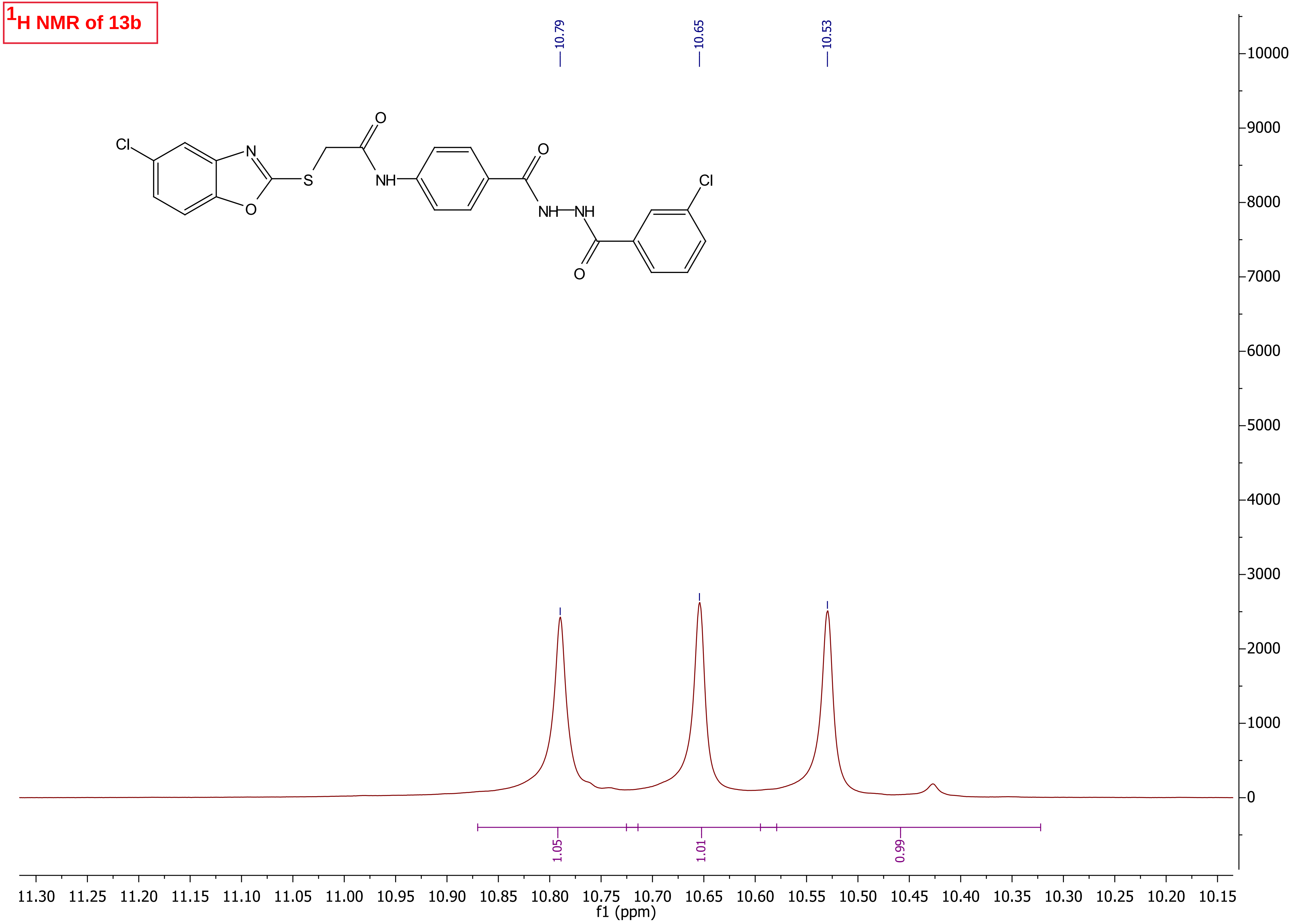

^{13}C NMR of 13b

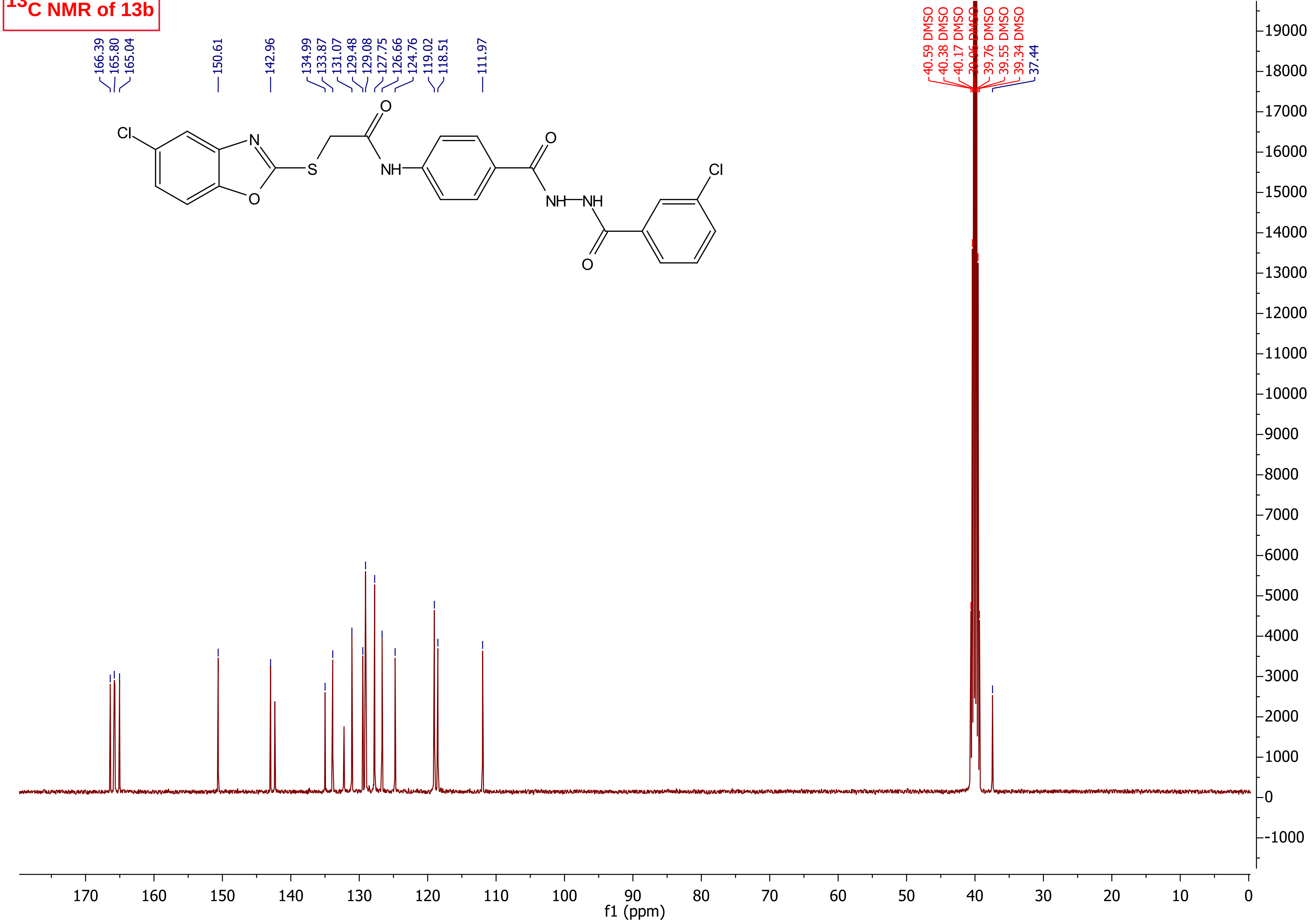

^{13}C NMR of 13b

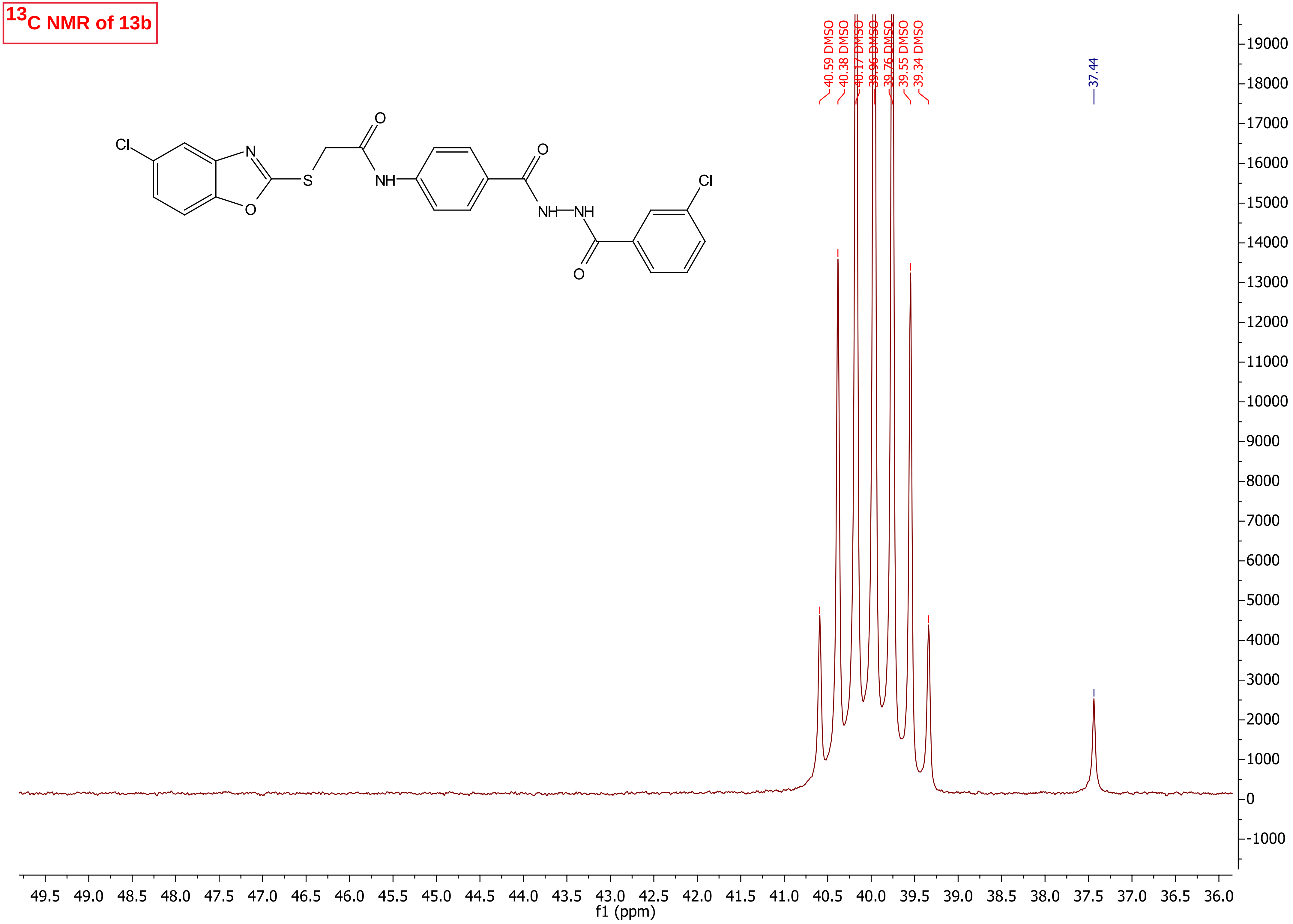

^{13}C NMR of 13b

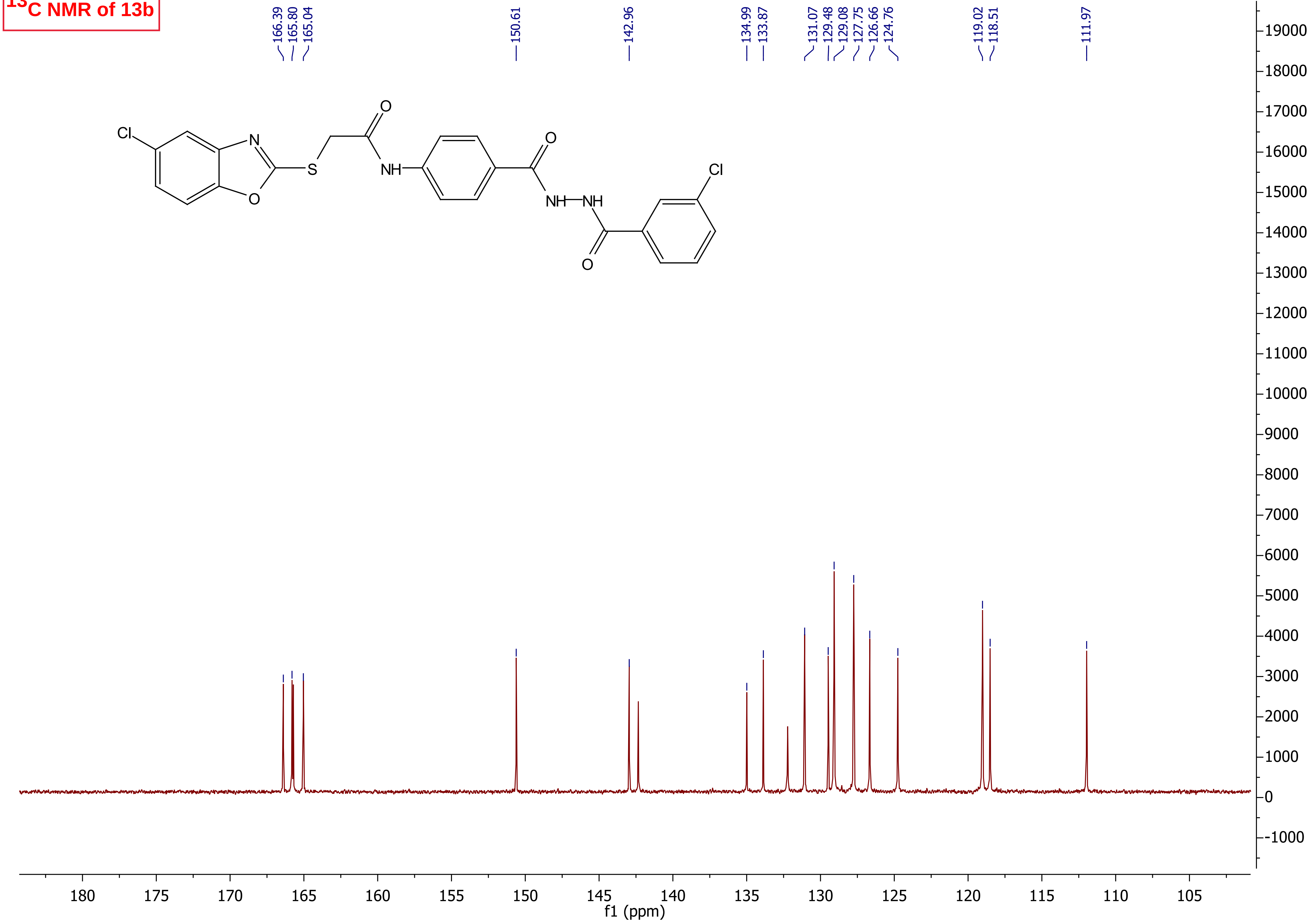

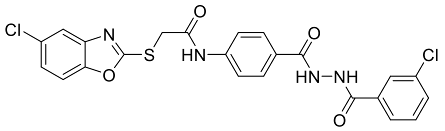

Mass spec. of 13b

RT: 0.00 - 5.51 SM: 7G

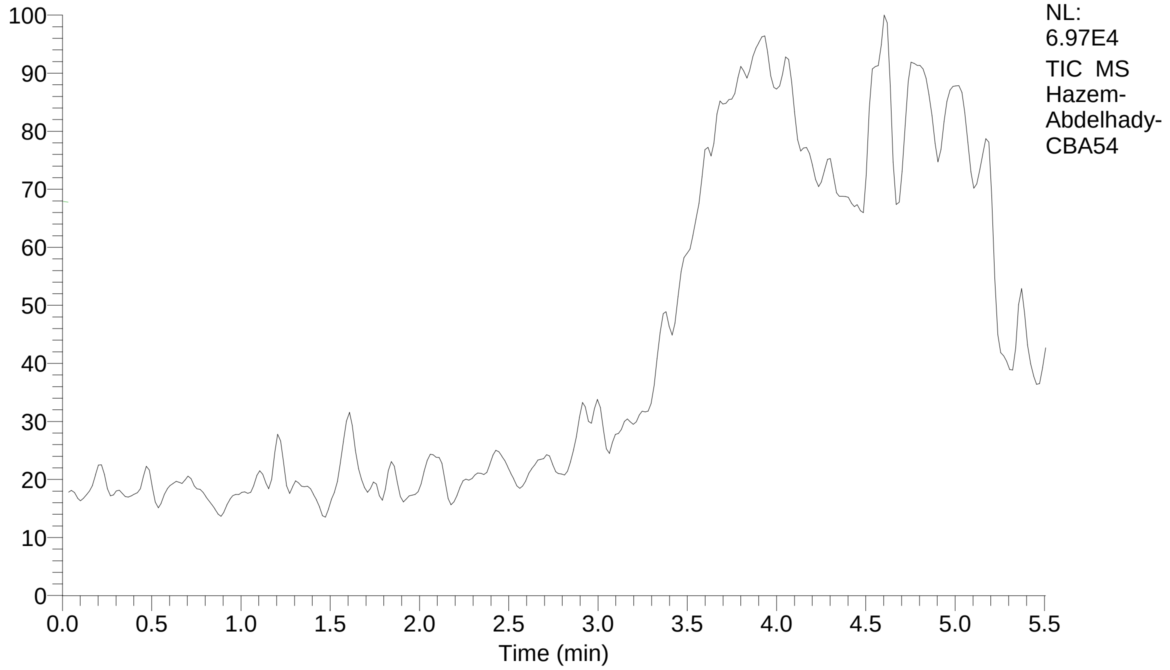

Hazem-Abdelhady-CBA54 #320-326 RT: 5.37-5.47 AV: 7 NL: 1.86E3
T: + c EI Full ms [40.00-1000.00]

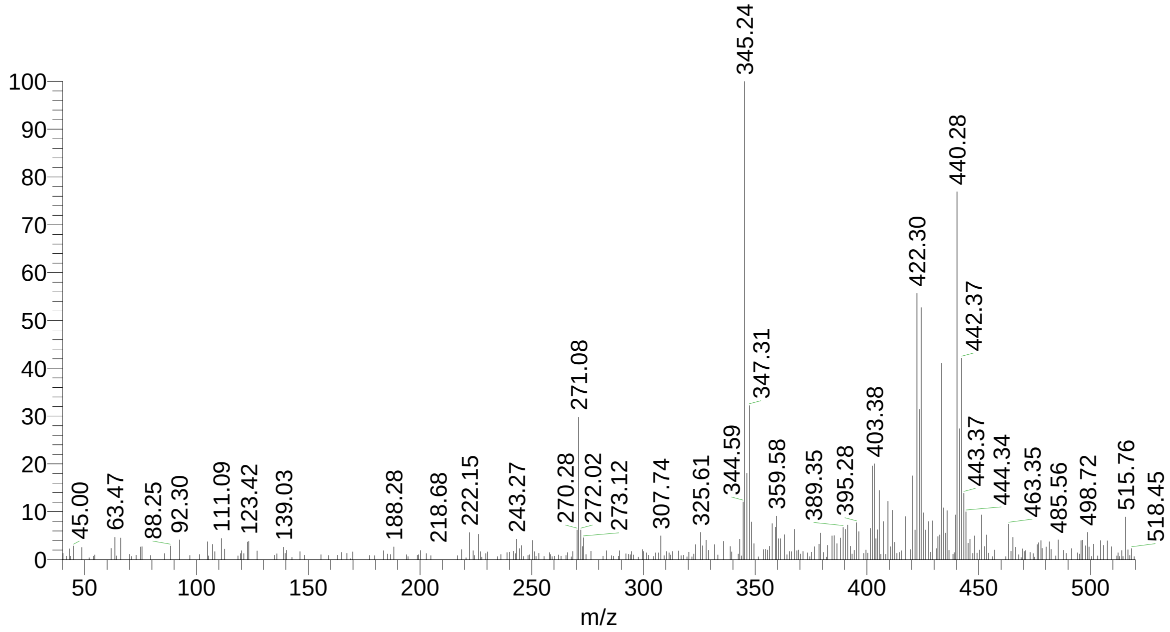

IR Of Comp. 13c

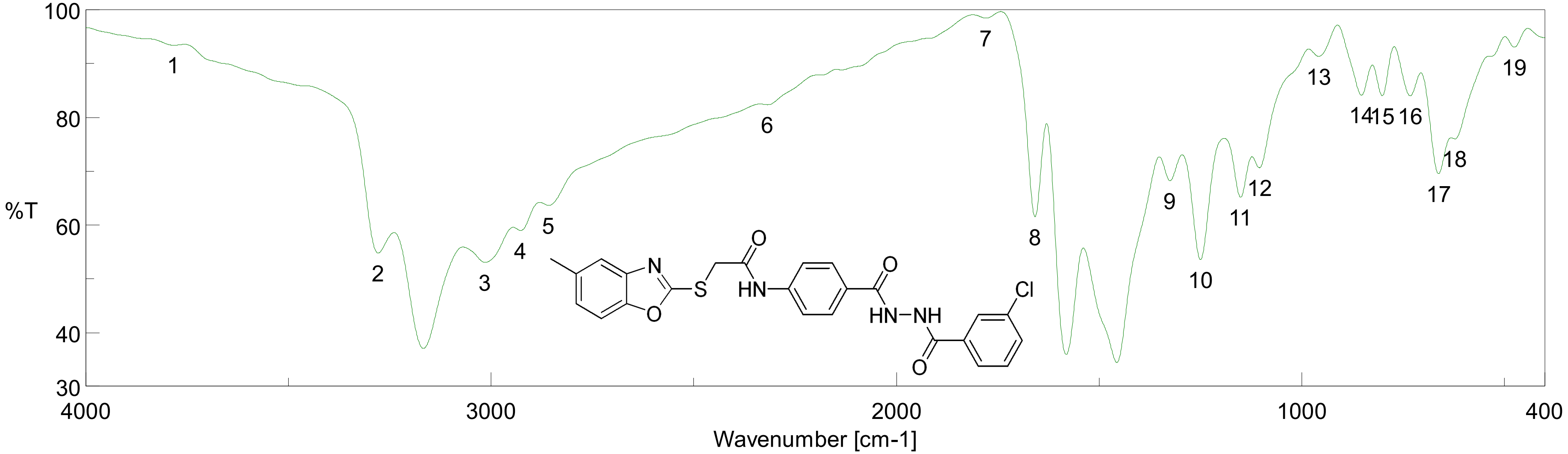

[Result of Peak Picking]

No.	Position	Intensity	No.	Position	Intensity	No.	Position	Intensity	No.	Position	Intensity
1	3784.62	93.3564	2	3279.36	54.7318	3	3015.16	52.9912	4	2927.41	58.9292
5	2857.99	63.6048	6	2318.02	82.3079	7	1779.01	98.4079	8	1657.52	61.4531
9	1324.86	68.1845	10	1249.65	53.495	11	1150.33	65.1047	12	1104.05	70.5942
13	957.484	91.281	14	852.382	84.1031	15	801.278	83.9659	16	731.853	83.954
17	662.428	69.4879	18	621.931	75.9664	19	475.367	93.0056			

^{1}H NMR of 13c

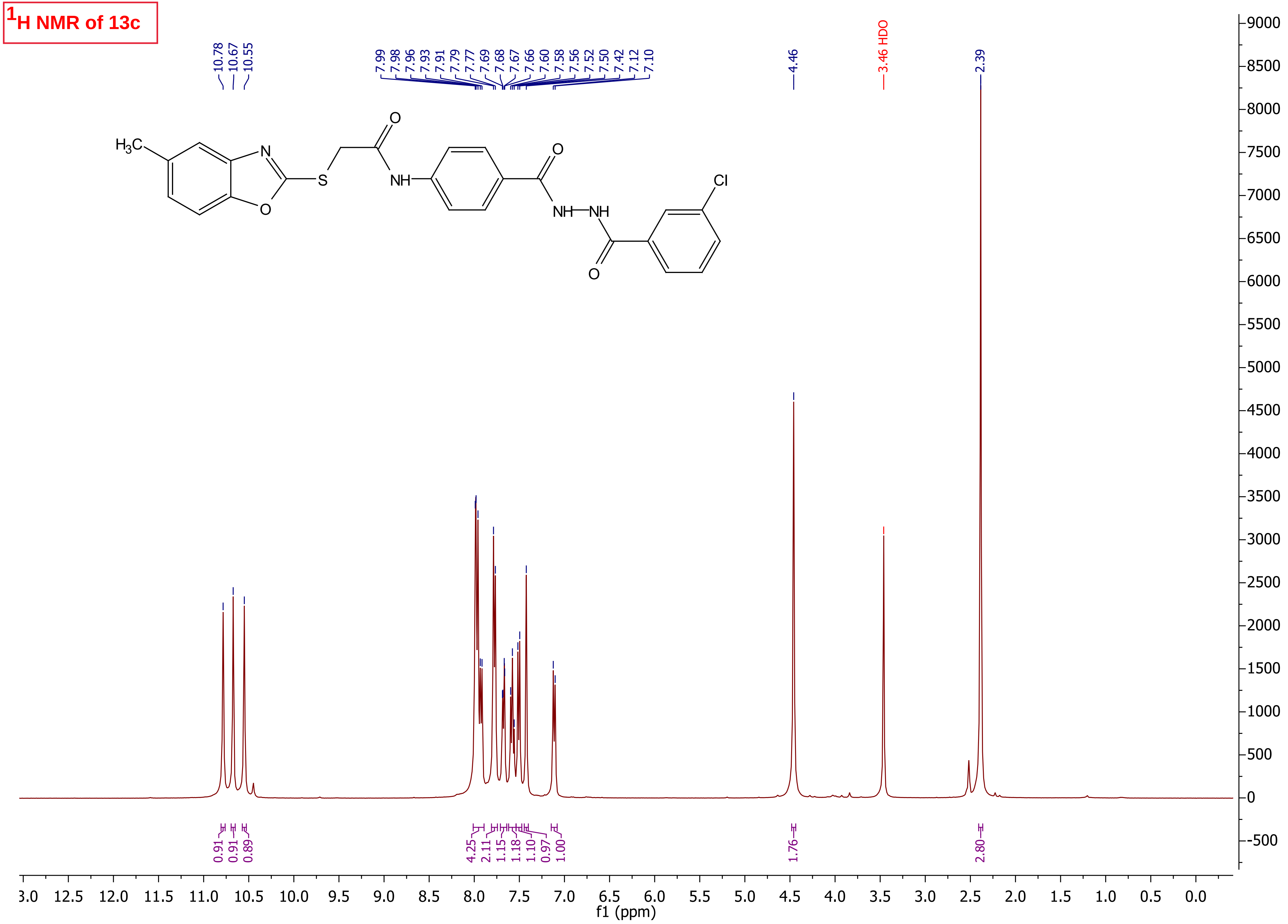

10.78
10.67
10.55
7.99
7.98
7.96
7.93
7.91
7.79
7.77
7.69
7.68
7.67
7.66
7.60
7.58
7.56
7.52
7.50
7.42
7.12
7.10
4.46
3.46 HDO
2.39
H3C
N
O
S
O
NH
O
NH—NH
Cl
O
0.91
0.91
0.89
4.25
2.11
1.15
1.18
1.10
0.97
1.00
1.76
2.80
f1 (ppm)

^{1}H NMR of 13c

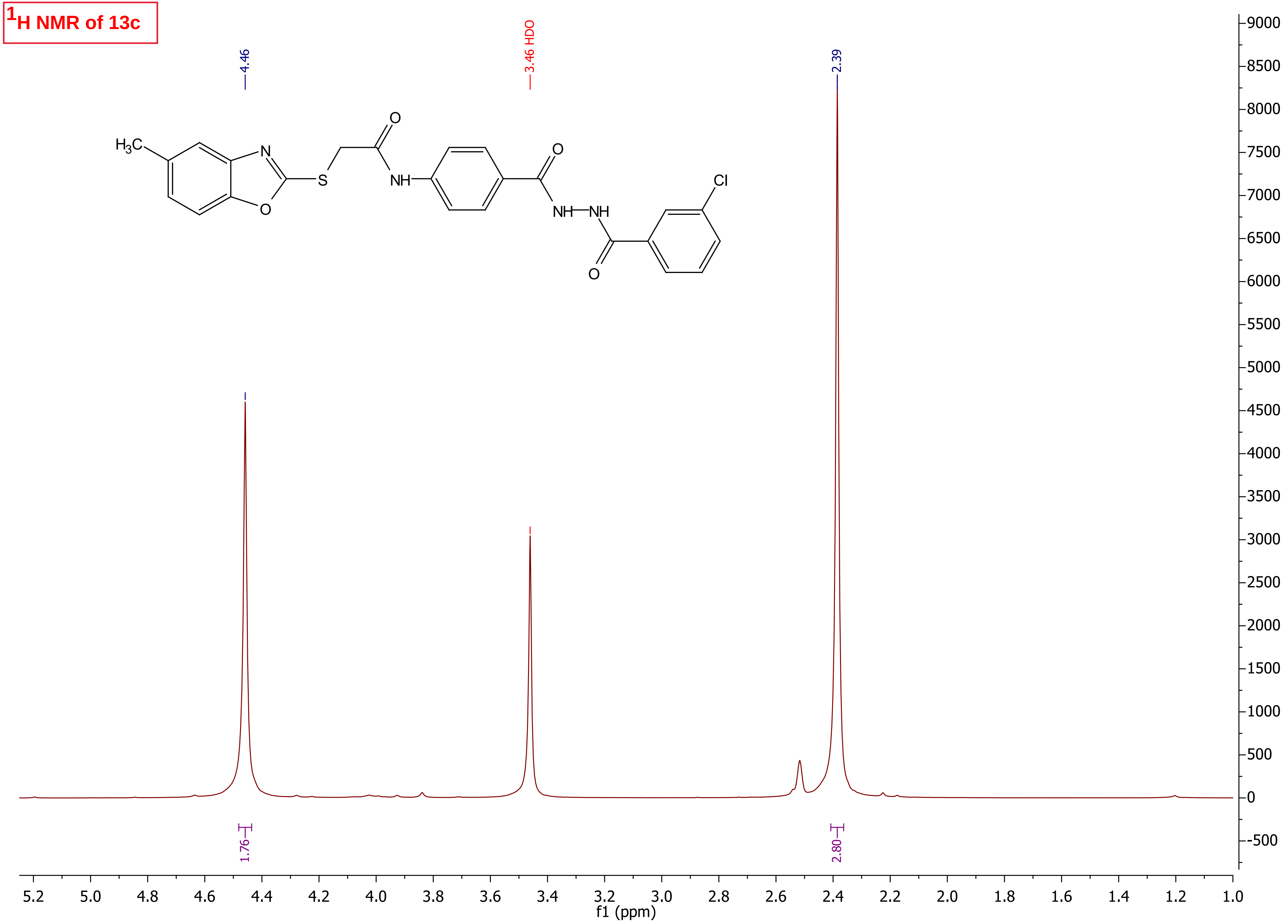

^{1}H NMR of 13c

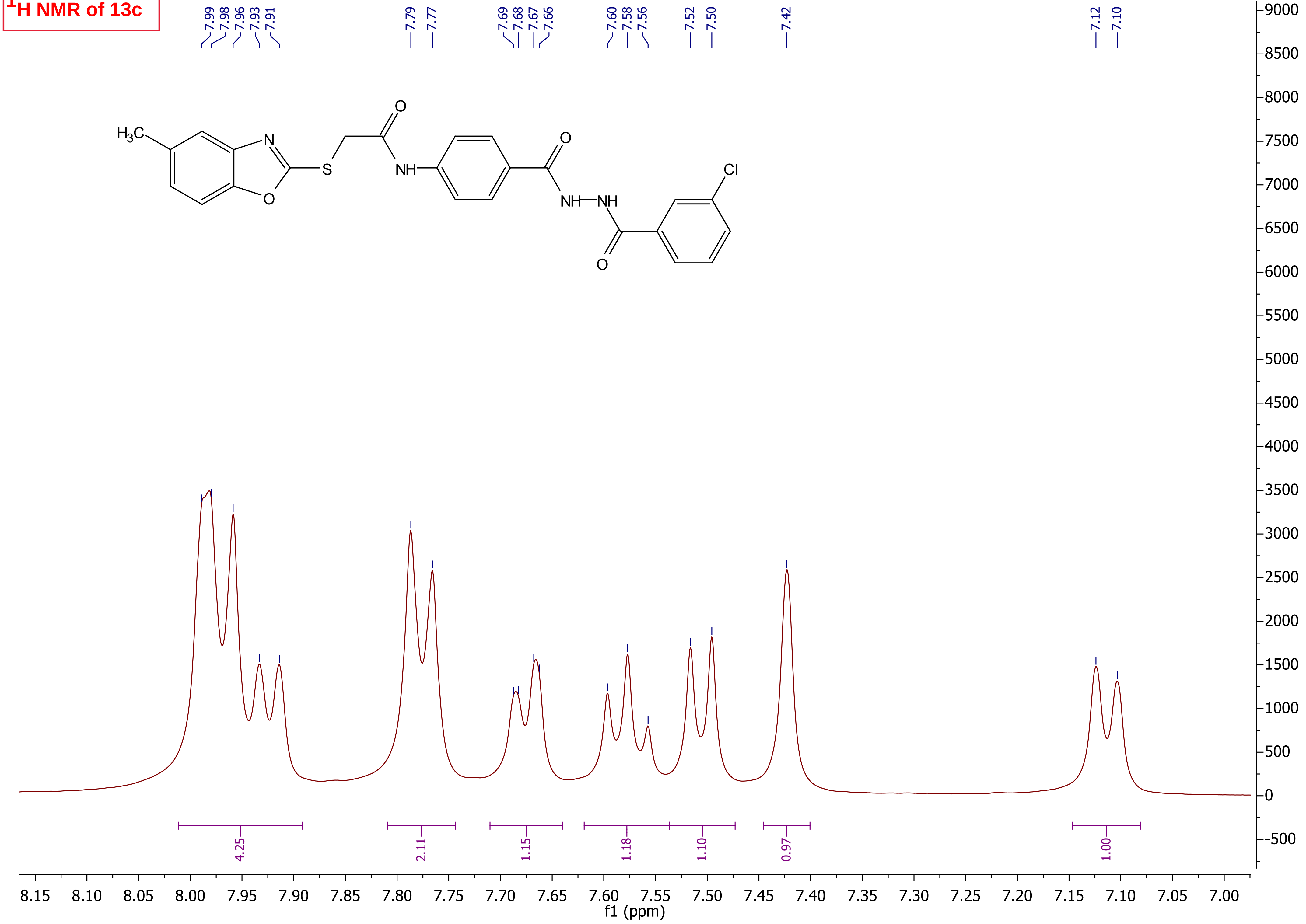

7.99
7.98
7.96
7.93
7.91
7.79
7.77
7.69
7.68
7.67
7.66
7.60
7.58
7.56
7.52
7.50
7.42
7.12
7.10
H3C
N
O
S
O
NH
O
NH
NH
Cl
O
4.25
2.11
1.15
1.18
1.10
0.97
1.00
f1 (ppm)

^{1}H NMR of 13c

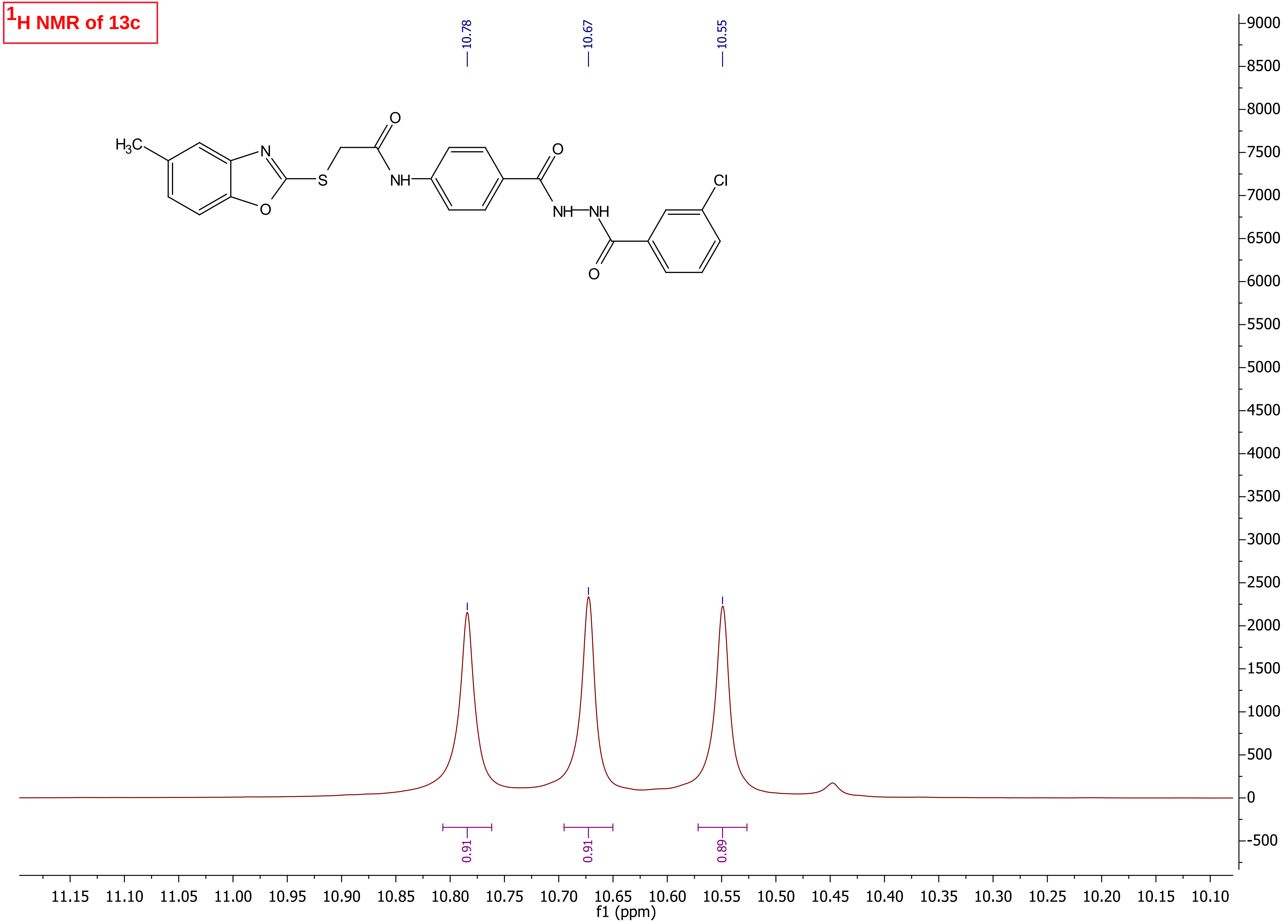
10.78
10.67
10.55
H3C
N
O
S
O
NH
O
NH
NH
Cl
O
0.91
0.91
0.89
11.15
11.10
11.05
11.00
10.95
10.90
10.85
10.80
10.75
10.70
10.65
10.60
10.55
10.50
10.45
10.40
10.35
10.30
10.25
10.20
10.15
10.10
f1 (ppm)
9000
8500
8000
7500
7000
6500
6000
5500
5000
4500
4000
3500
3000
2500
2000
1500
1000
500
0
-500

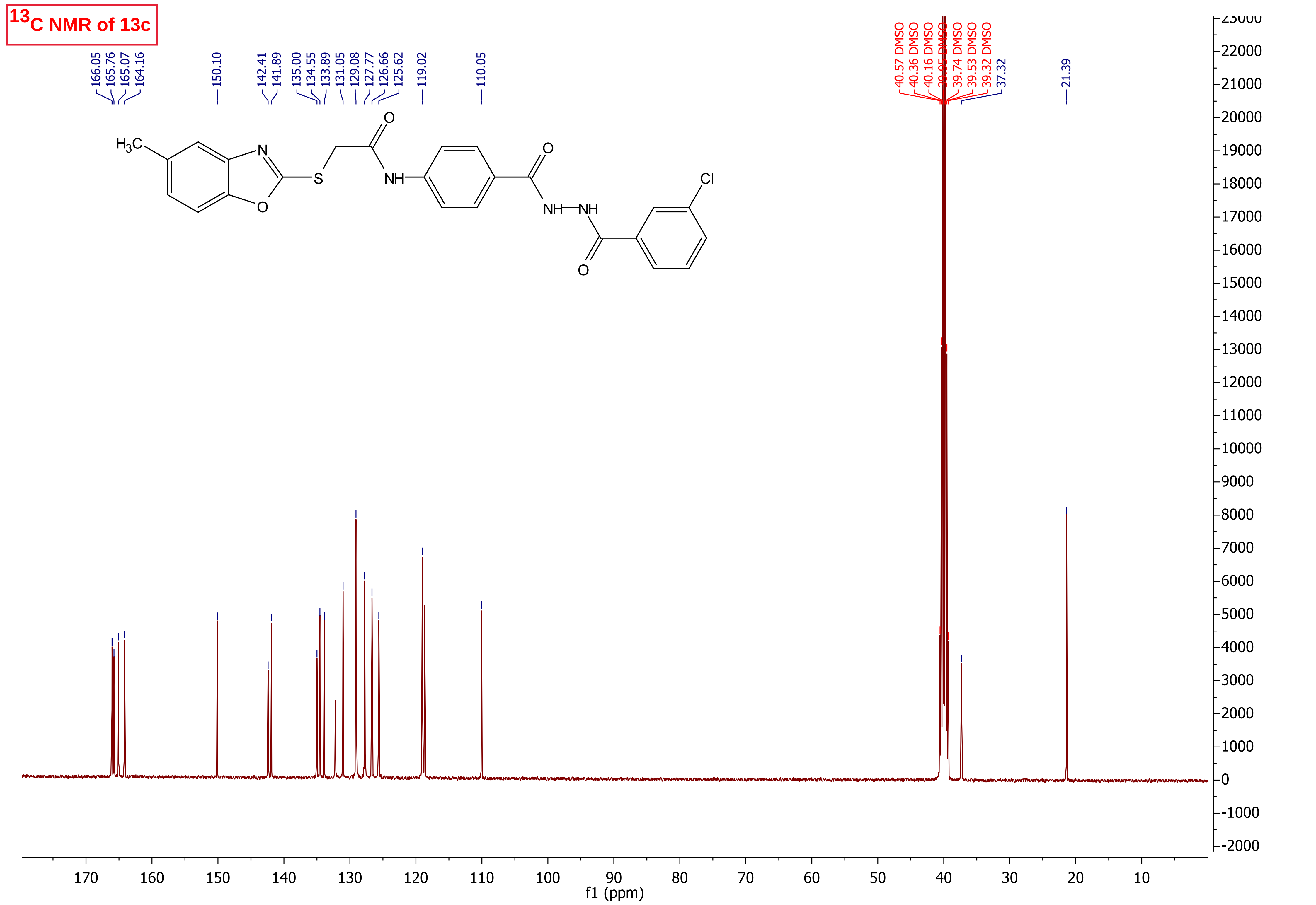

13C NMR of 13c
166.05
165.76
165.07
164.16
150.10
142.41
141.89
135.00
134.55
133.89
131.05
129.08
127.77
126.66
125.62
119.02
110.05
40.57 DMSO
40.36 DMSO
40.16 DMSO
39.95 DMSO
39.74 DMSO
39.53 DMSO
39.32 DMSO
37.32
21.39
H3C
N
O
S
O
NH
O
NH
NH
Cl
O
f1 (ppm)

^{13}C NMR of 13c

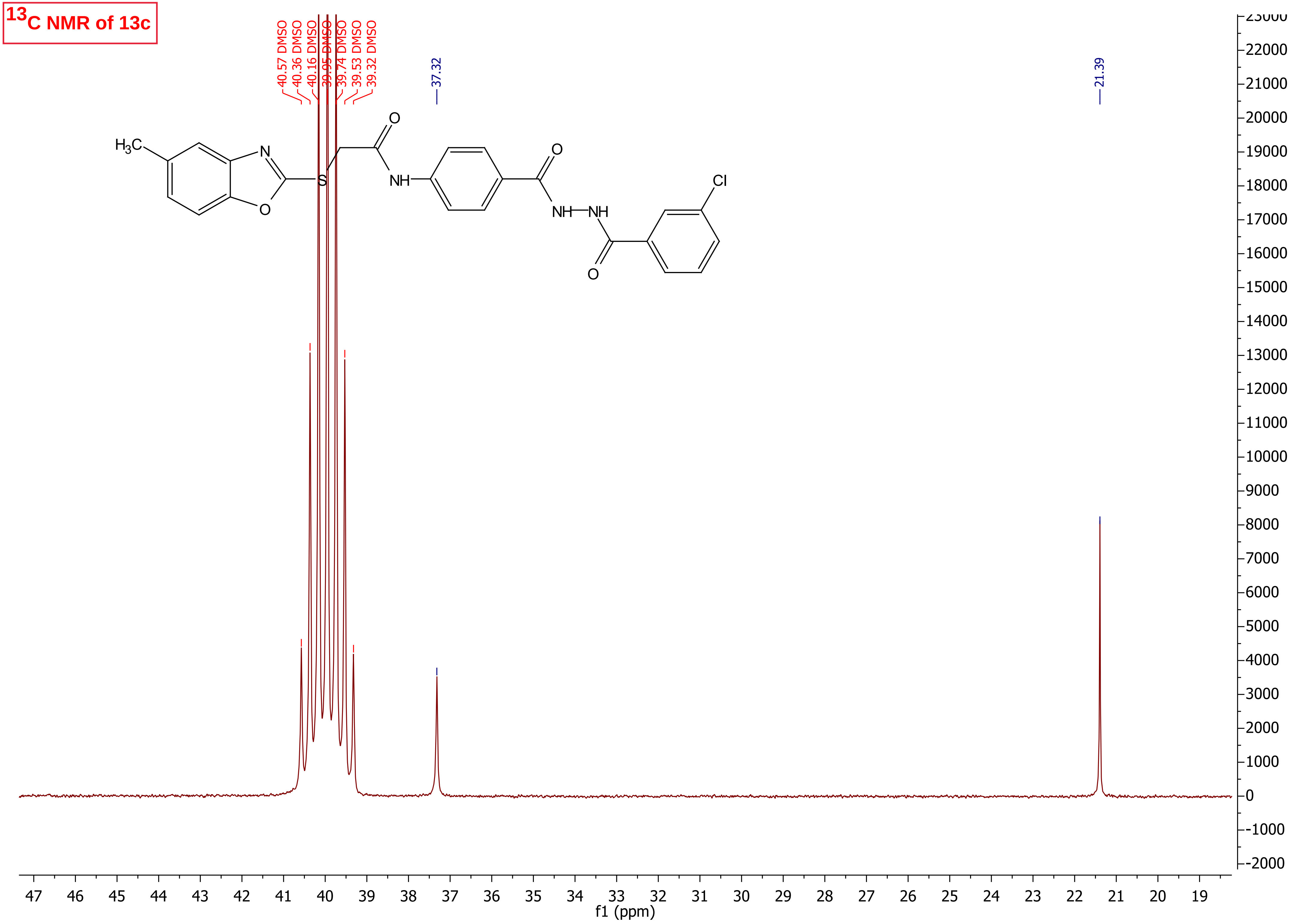

^{13}C NMR of 13c

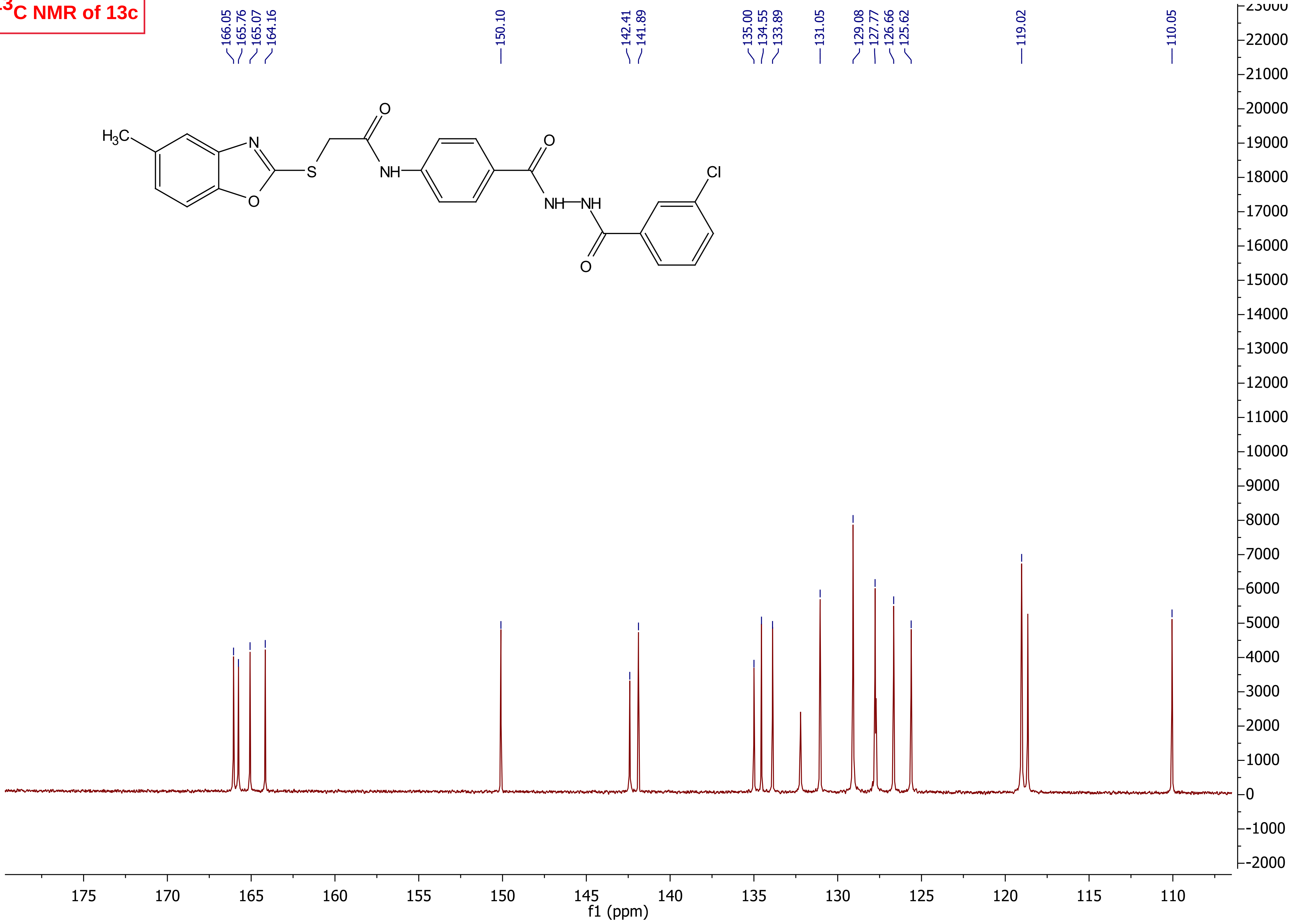

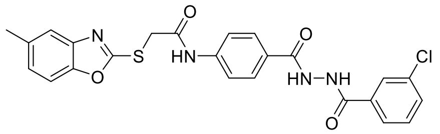

Mass spec. of 13c

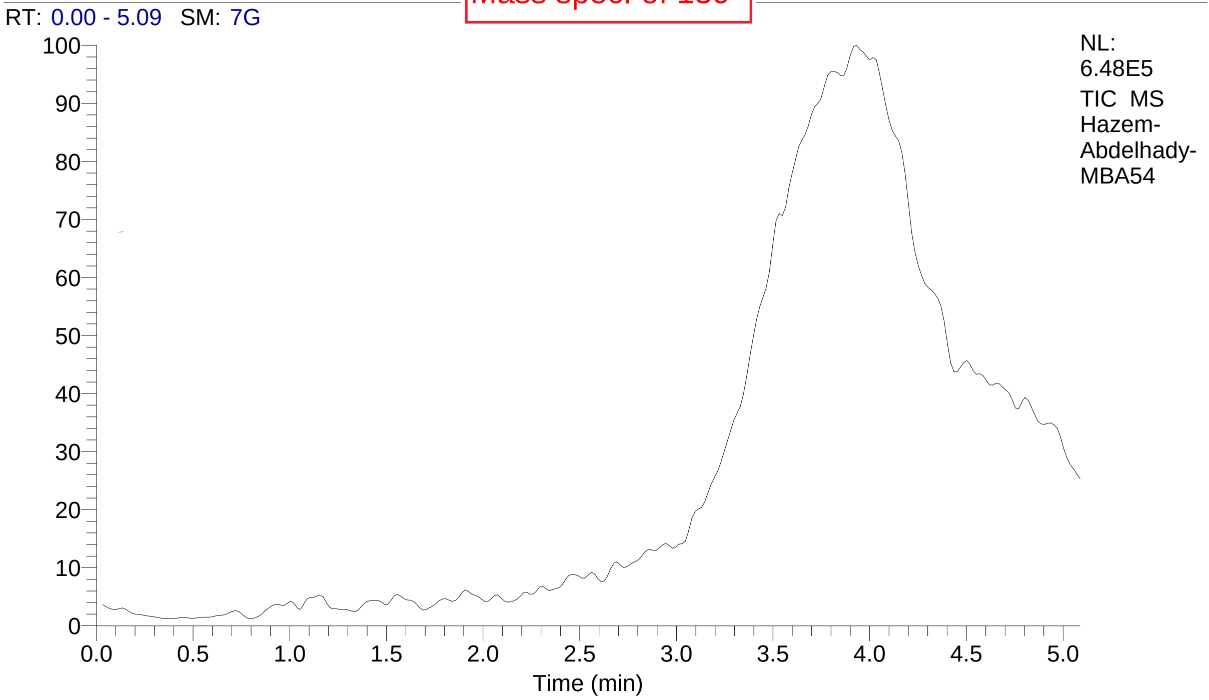

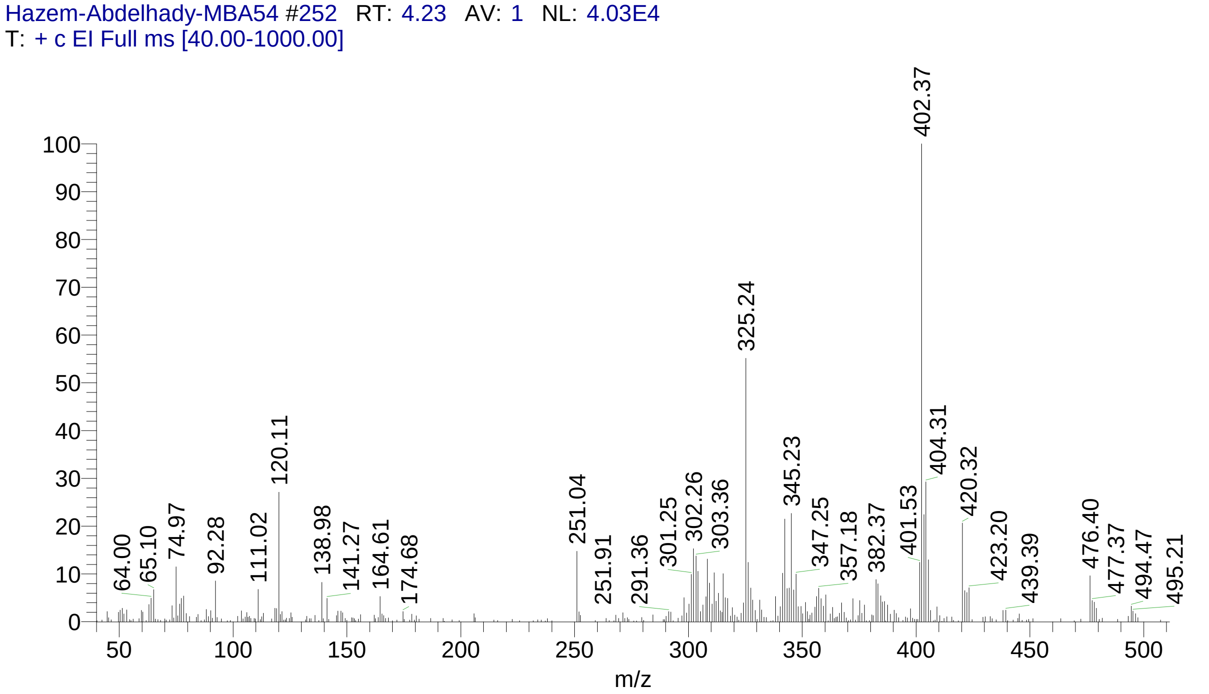

Raw data of the cytotoxicity of the synthesized compounds against HepG2 and MCF-7 cell lines

Cytotoxicity of 12a against HepG2 cell line

Best-fit values	
LogIC50	1.589
HillSlope	-0.4109
IC50	38.83

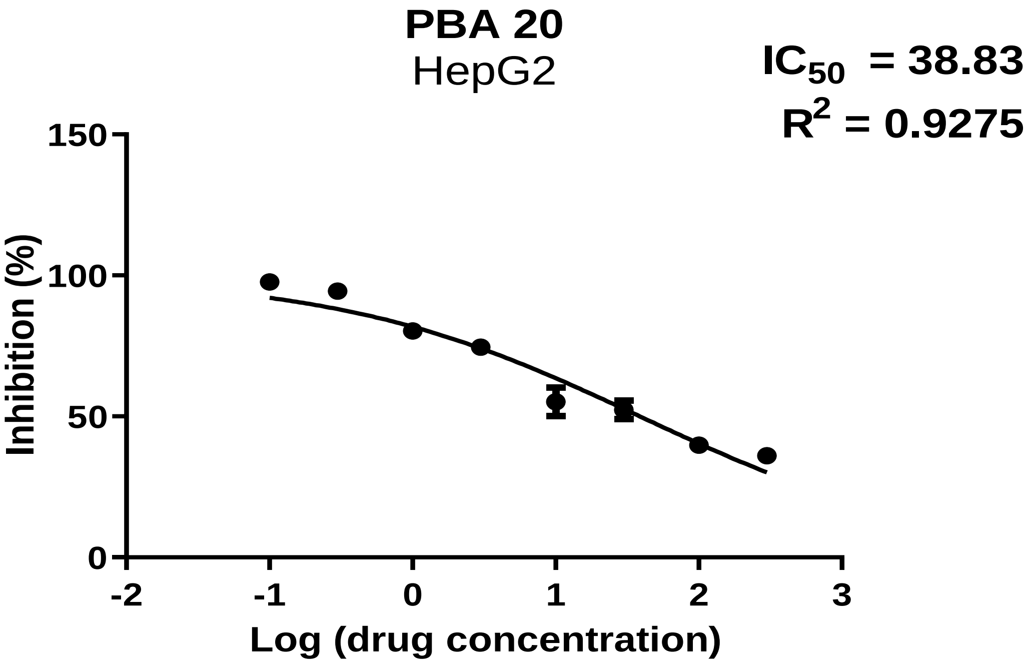

Cytotoxicity of 12b against HepG2 cell line

Best-fit values	
LogIC50	1.807
HillSlope	-0.4183
IC50	64.16

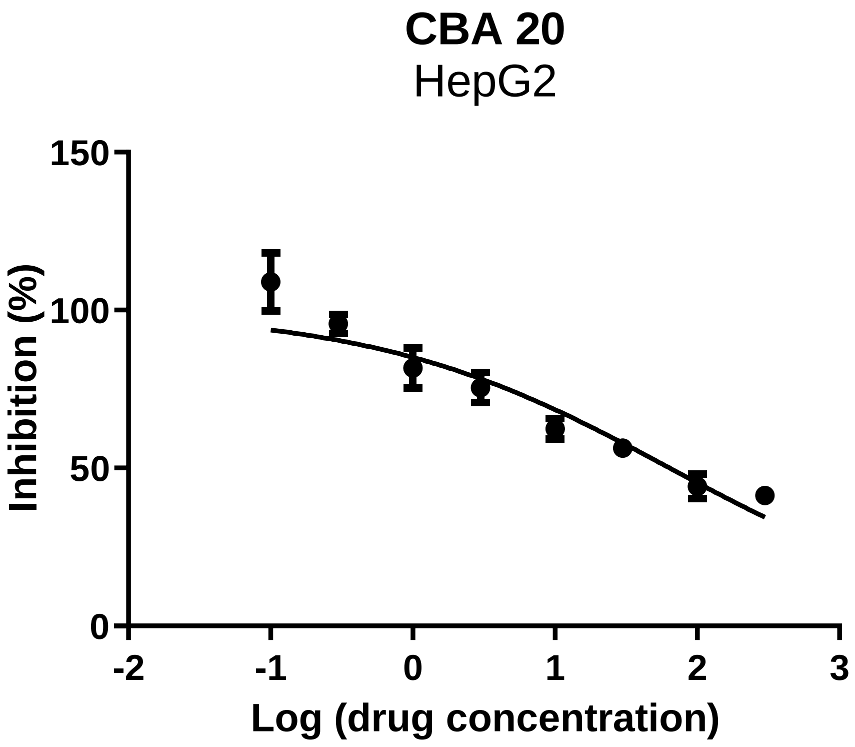

Cytotoxicity of 12c against HepG2 cell line

Best-fit values	
LogIC50	1.871
HillSlope	-0.4624
IC50	74.30

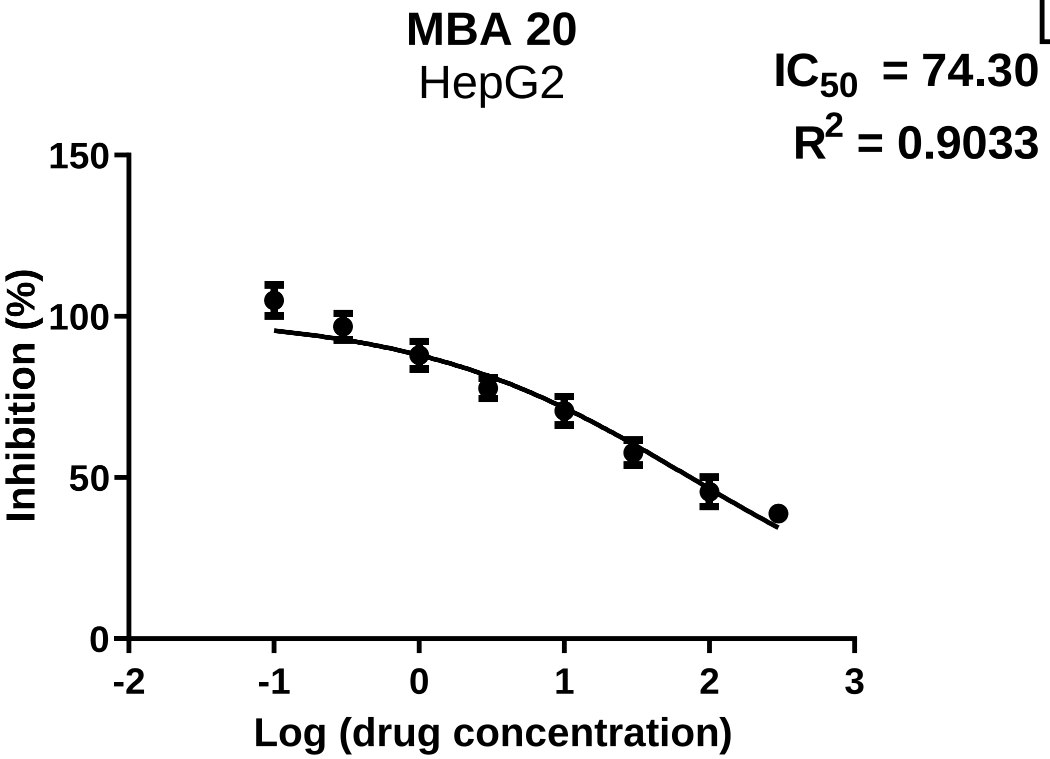

Cytotoxicity of 12d against HepG2 cell line

Best-fit values	
LogIC50	1.373
HillSlope	-0.3615
IC50	23.61

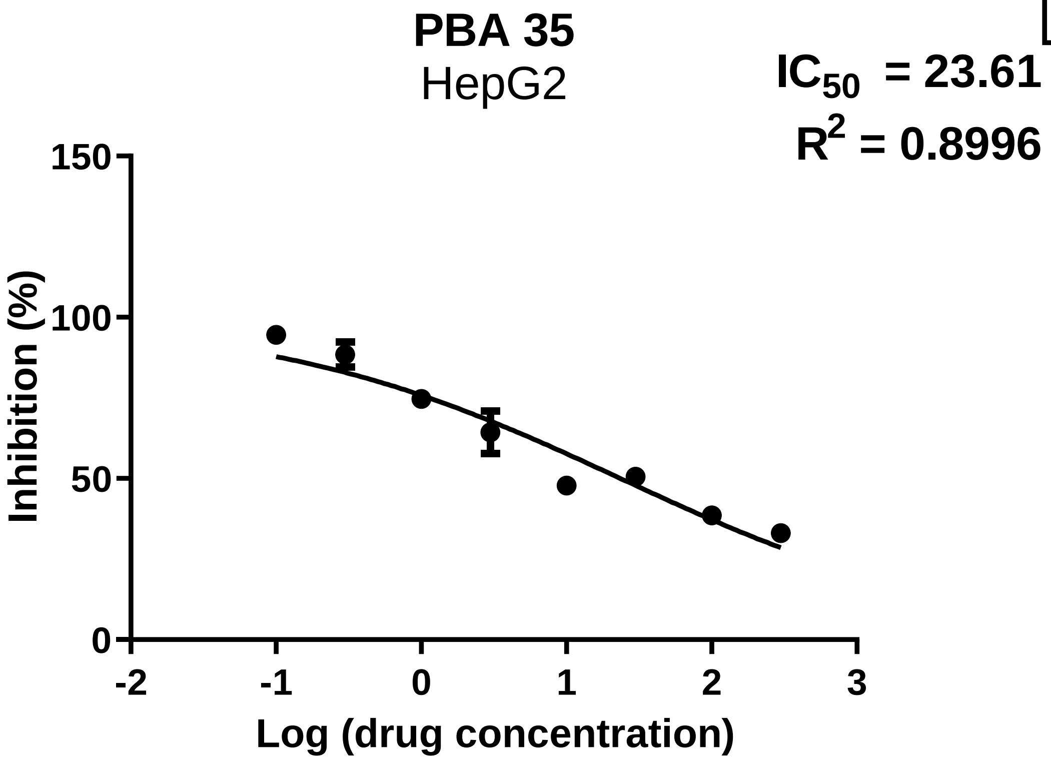

Cytotoxicity of 12e against HepG2 cell line

Best-fit values	
LogIC50	1.855
HillSlope	-0.3212
IC50	71.59

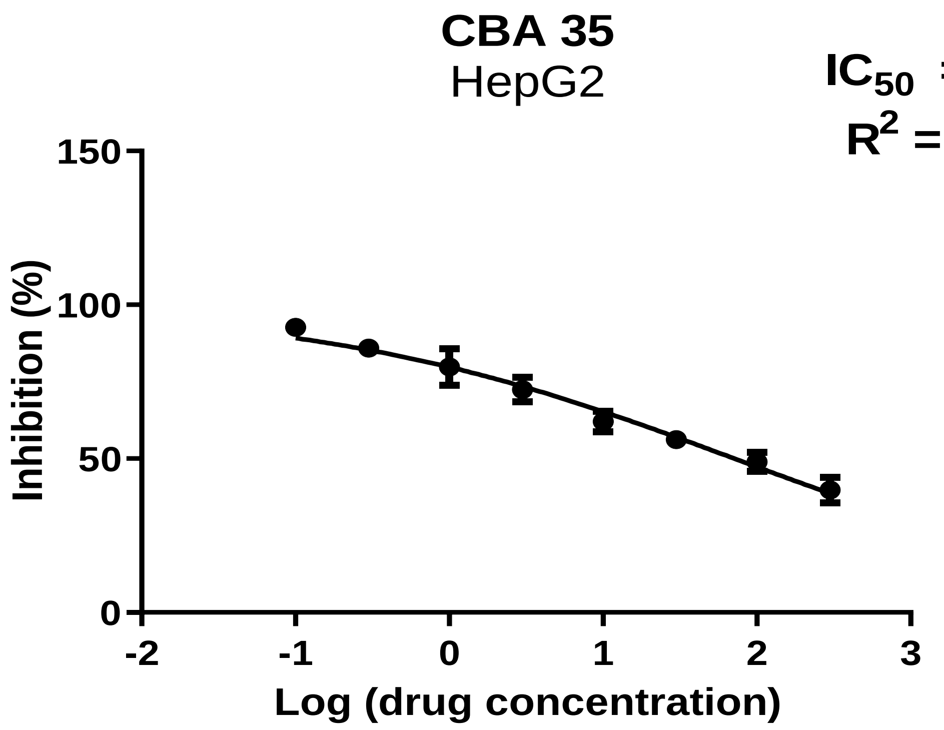

IC_{50} = 71.59

$R^2 = 0.9059$

Cytotoxicity of 12f against HepG2 cell line

Best-fit values	
LogIC50	1.568
HillSlope	-0.2978
IC50	36.96

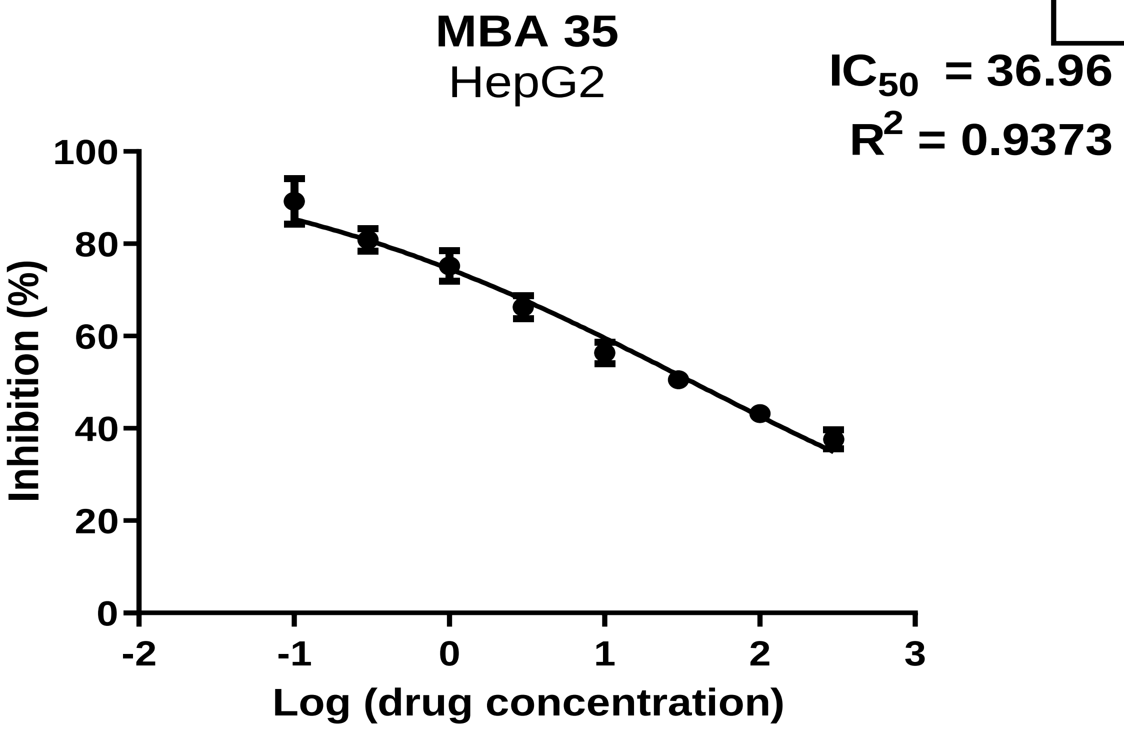

Cytotoxicity of 12g against HepG2 cell line

Best-fit values	
LogIC50	1.564
HillSlope	-0.3429
IC50	36.67

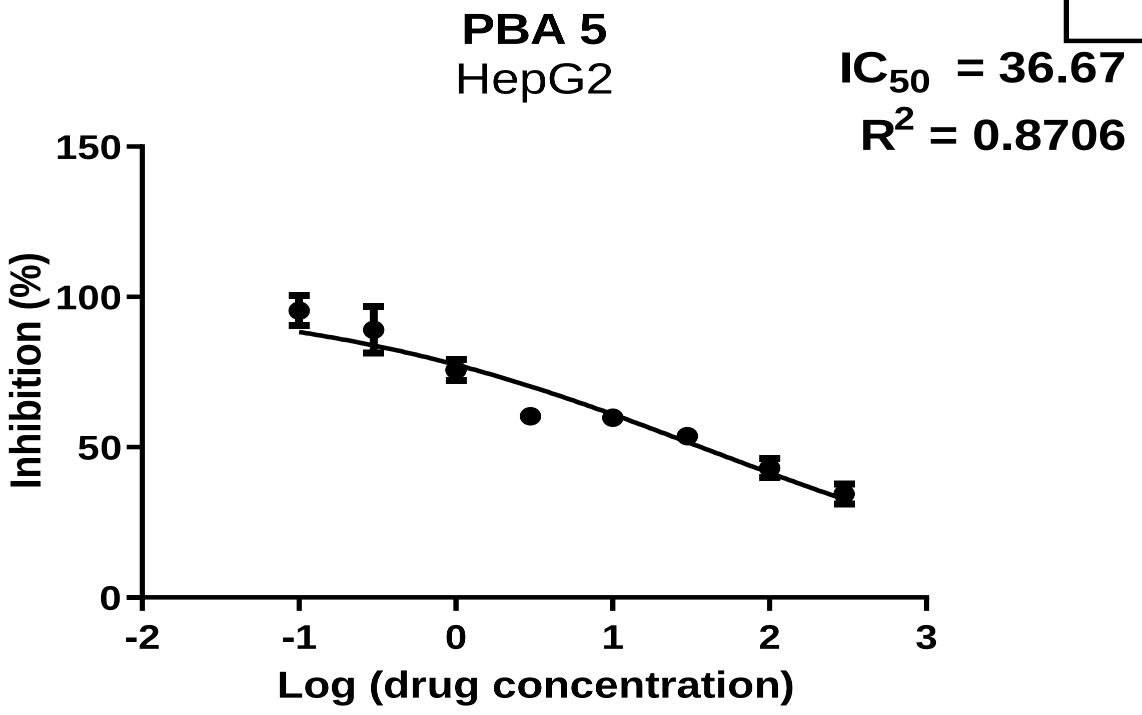

Cytotoxicity of 12h against HepG2 cell line

Best-fit values	
LogIC50	2.009
HillSlope	-0.4247
IC50	102.1

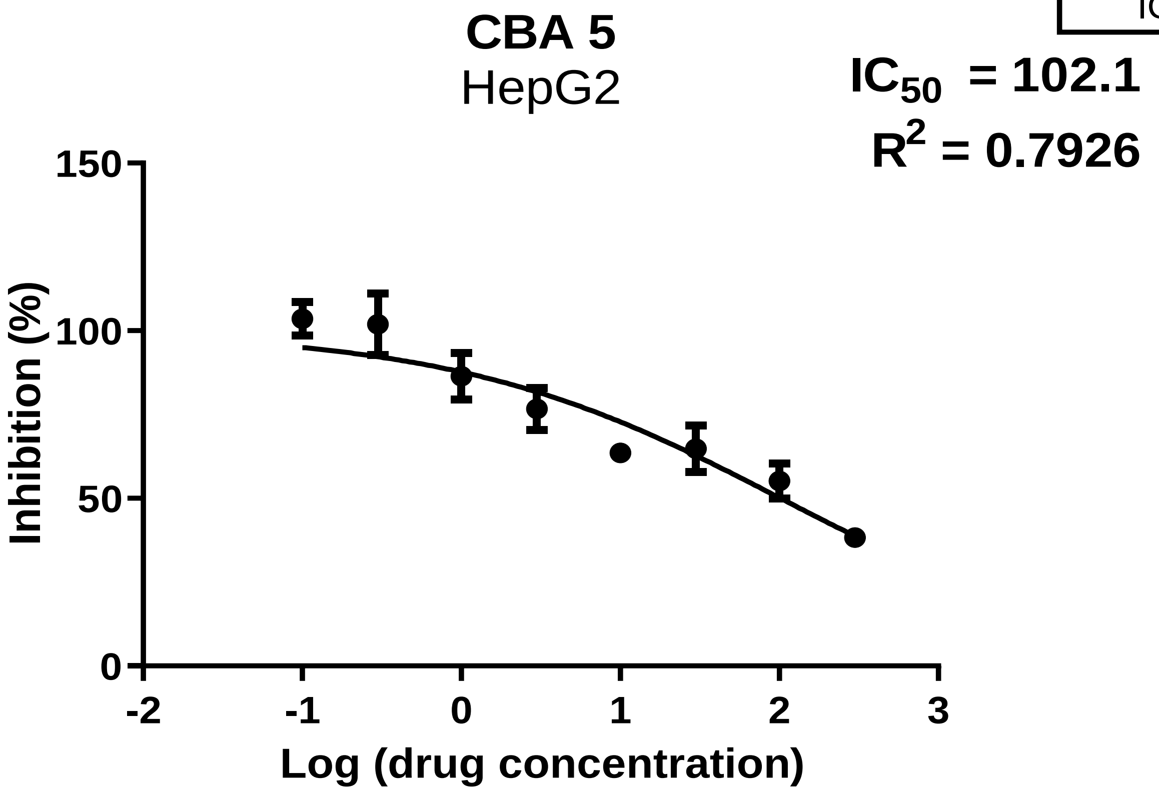

Cytotoxicity of 12i against HepG2 cell line

Best-fit values	
LogIC50	1.436
HillSlope	-0.4222
IC50	27.30

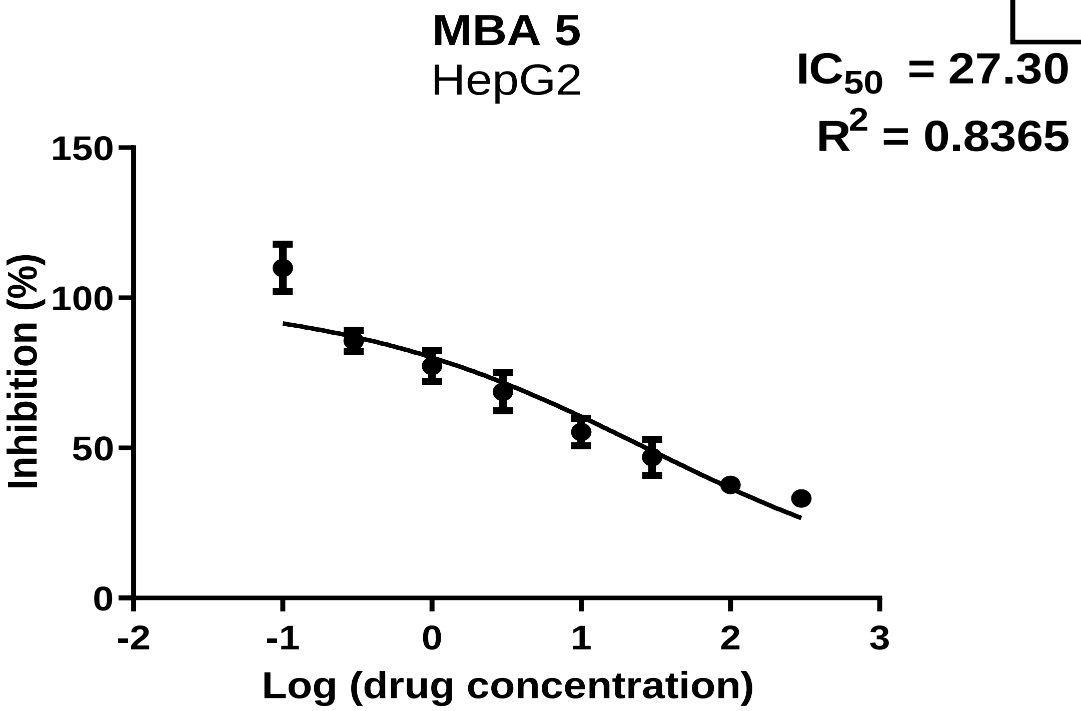

Cytotoxicity of 12j against HepG2 cell line

Best-fit values	
LogIC50	1.707
HillSlope	-0.4183
IC50	50.92

IC_{50} = 50.92

R^2 = 0.8486

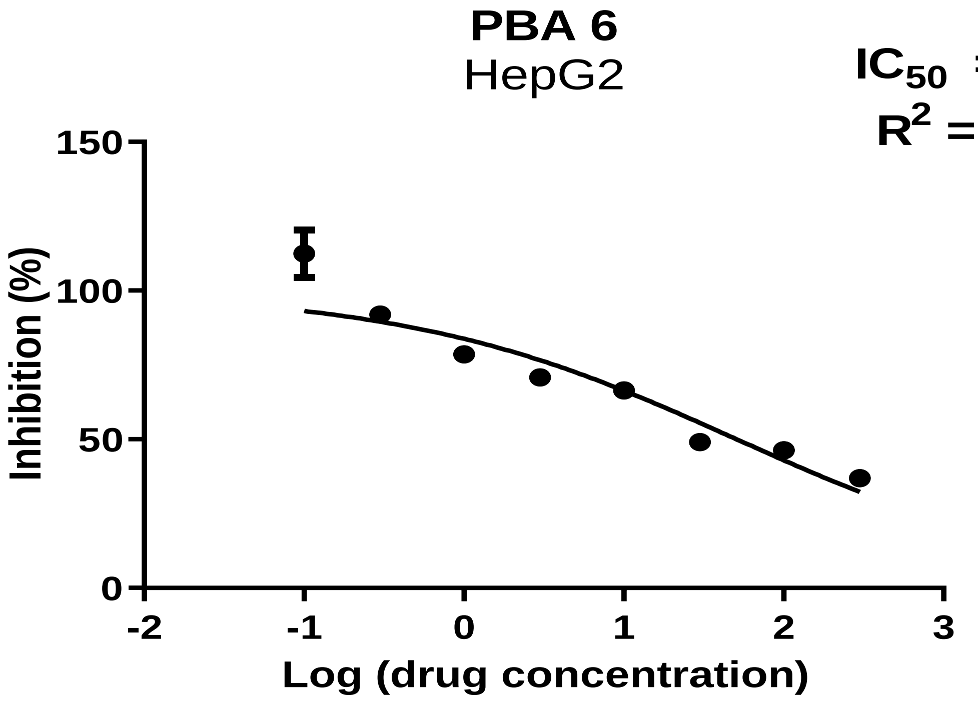

Cytotoxicity of 12k against HepG2 cell line

Best-fit values	
LogIC50	1.453
HillSlope	-0.3732
IC50	28.36

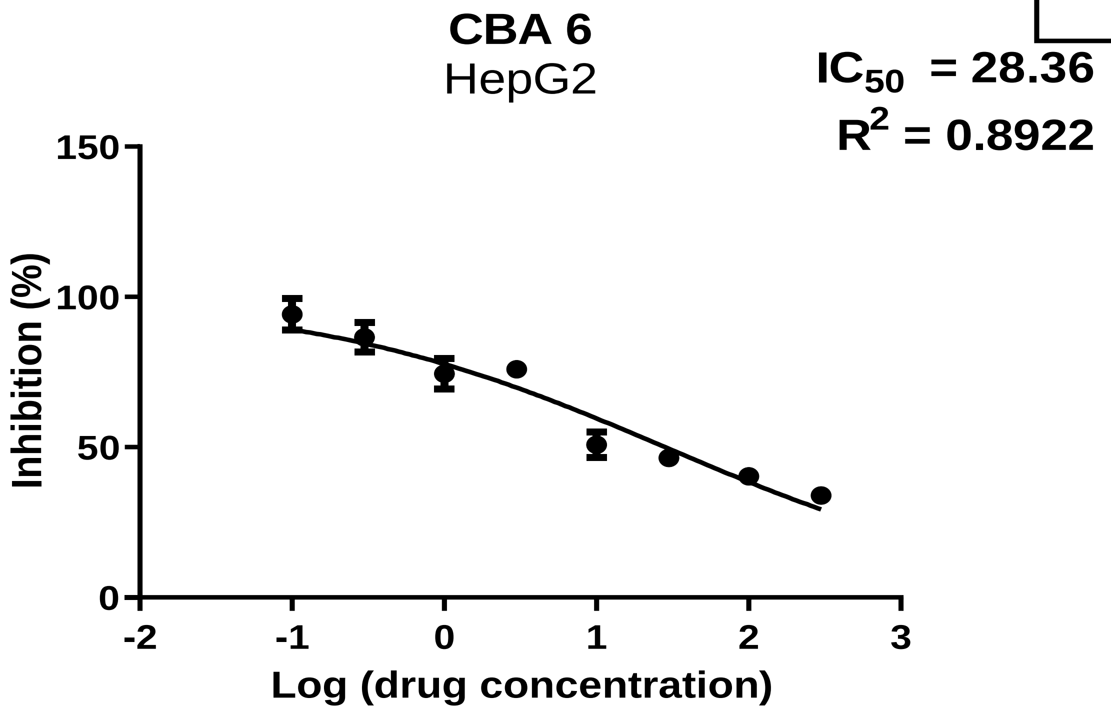

Cytotoxicity of 12l against HepG2 cell line

Best-fit values	
LogIC50	1.021
HillSlope	-0.3539
IC50	10.50

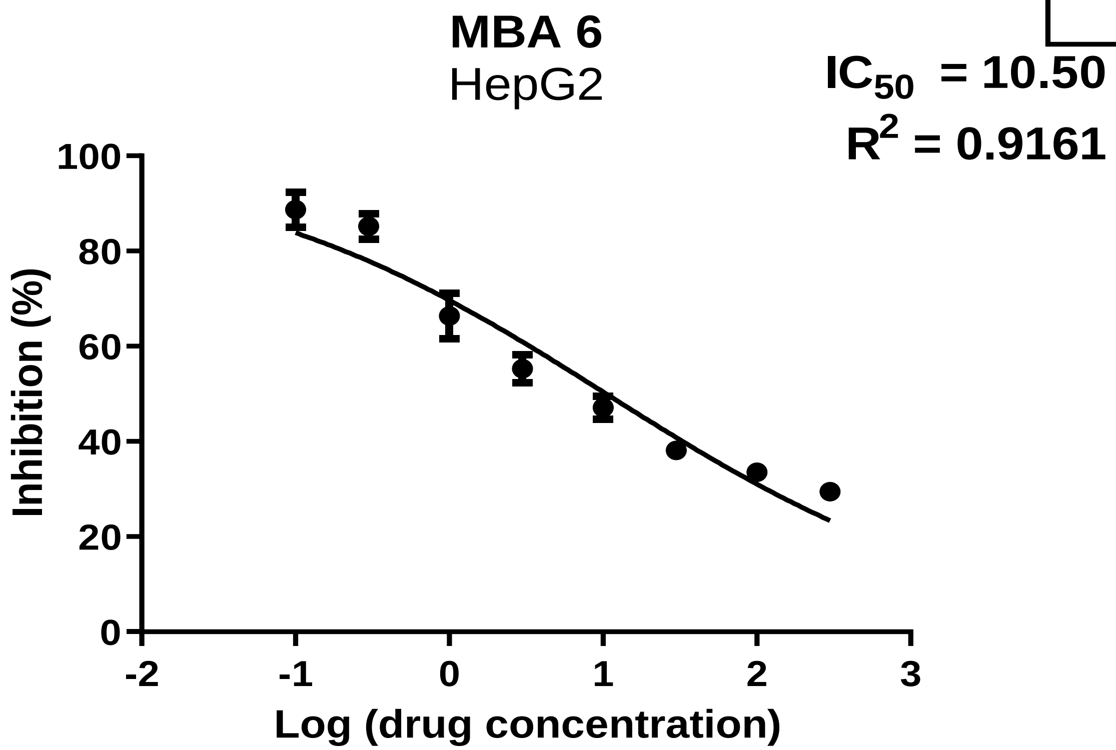

Cytotoxicity of 13a against HepG2 cell line

Best-fit values	
LogIC50	1.406
HillSlope	-0.3251
IC50	25.47

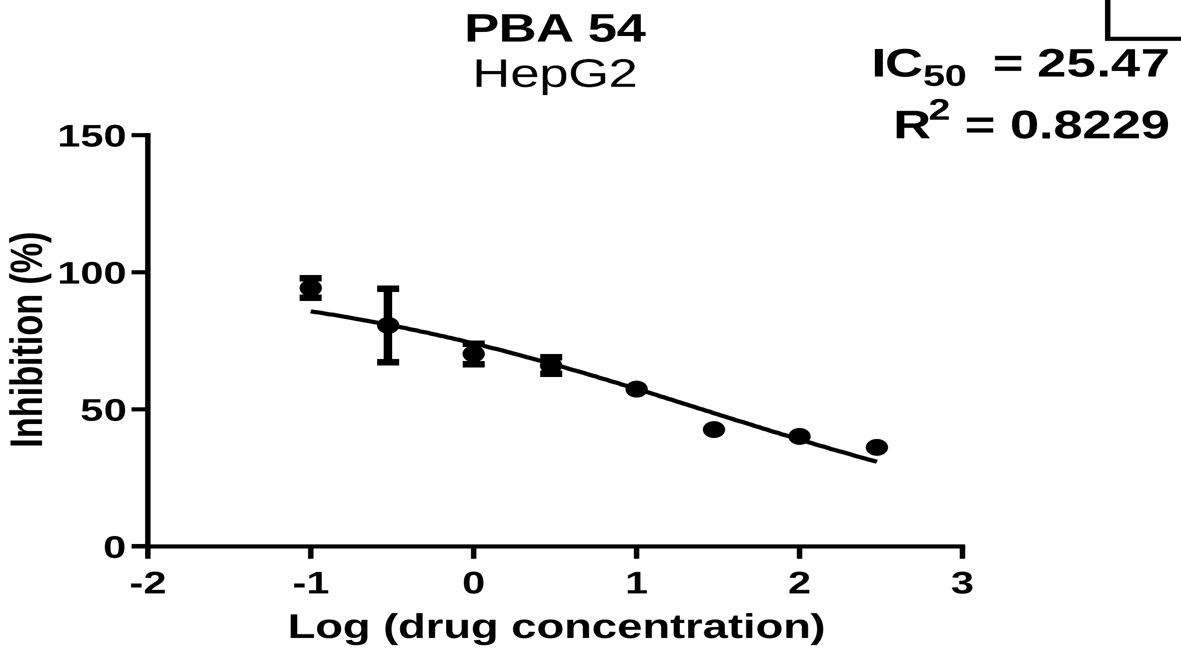

Cytotoxicity of 13b against HepG2 cell line

Best-fit values	
LogIC50	1.624
HillSlope	-0.4269
IC50	42.06

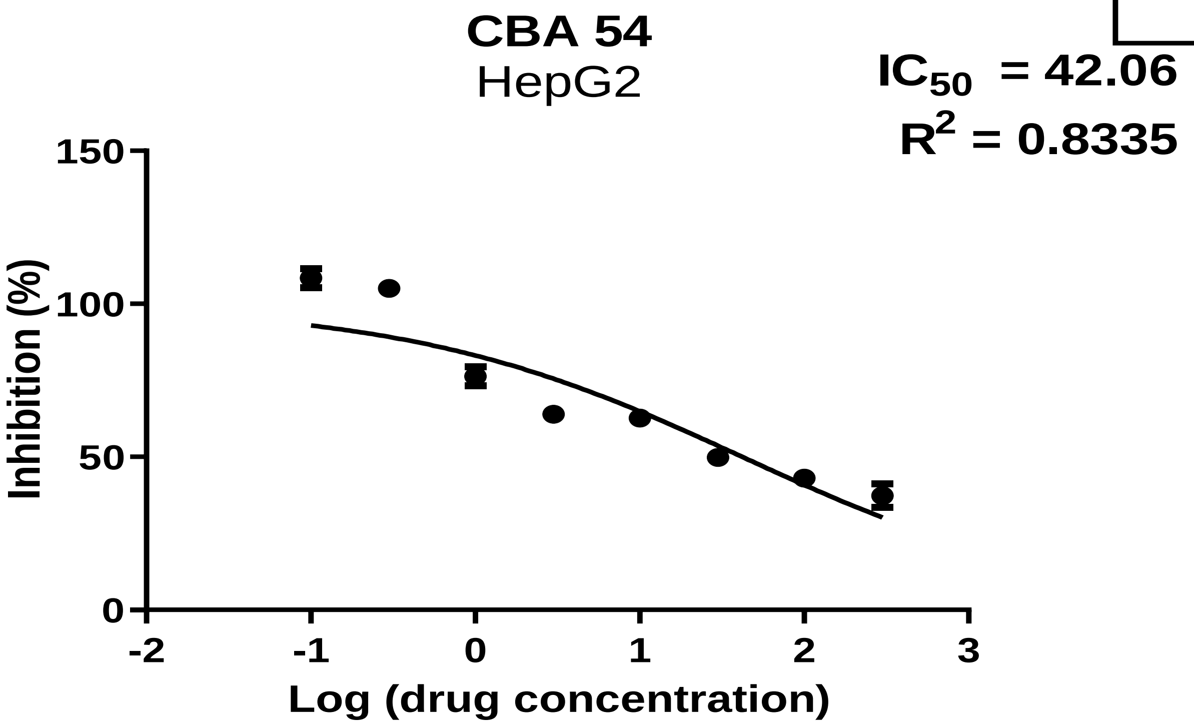

Cytotoxicity of 13c against HepG2 cell line

Best-fit values	
LogIC50	1.388
HillSlope	-0.4036
IC50	24.45

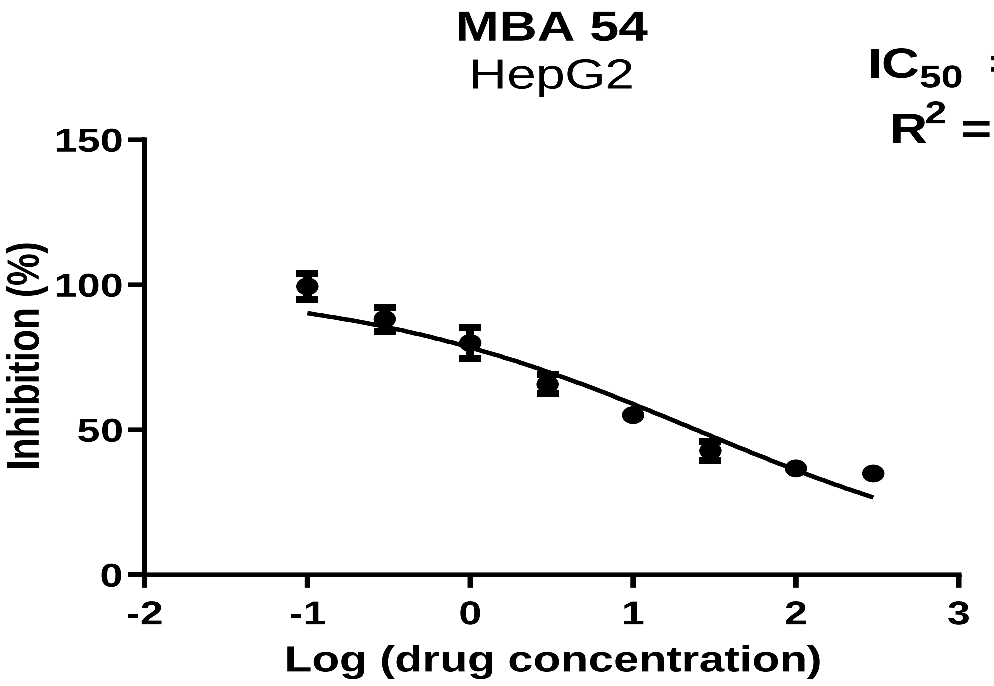

IC_{50} = 24.45

R^2 = 0.9071

Cytotoxicity of sorafeenib against HepG2 cell line

Best-fit values	
LogIC50	0.7461
HillSlope	-0.3199
IC50	5.573

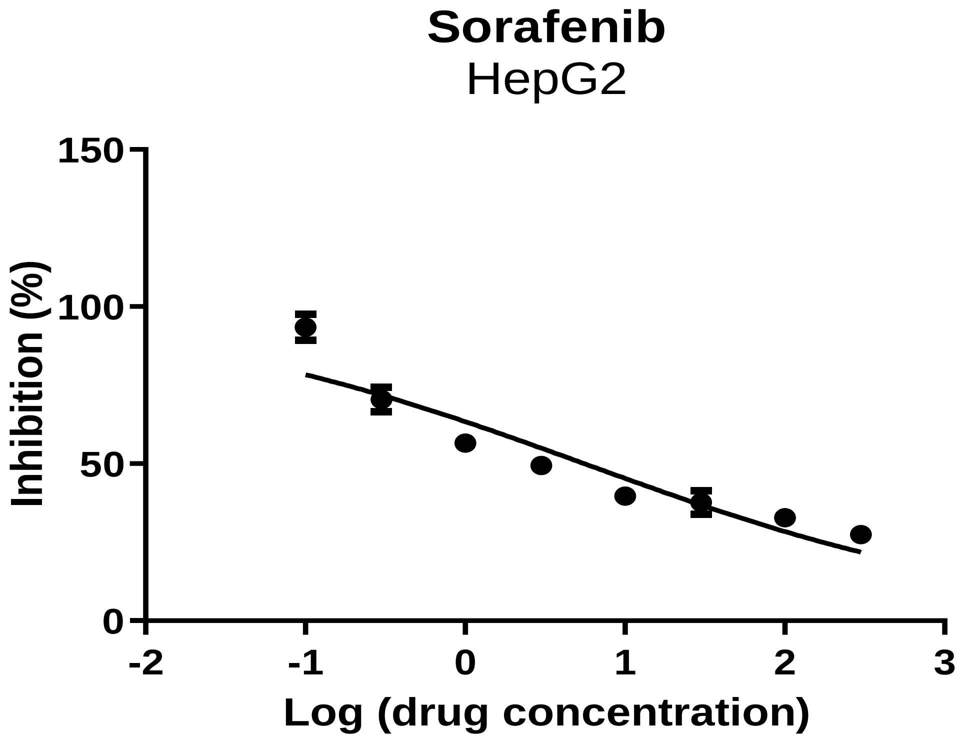

Cytotoxicity of 12a against MCF-7 cell line

Best-fit values	
LogIC50	1.522
HillSlope	-0.3559
IC50	33.27

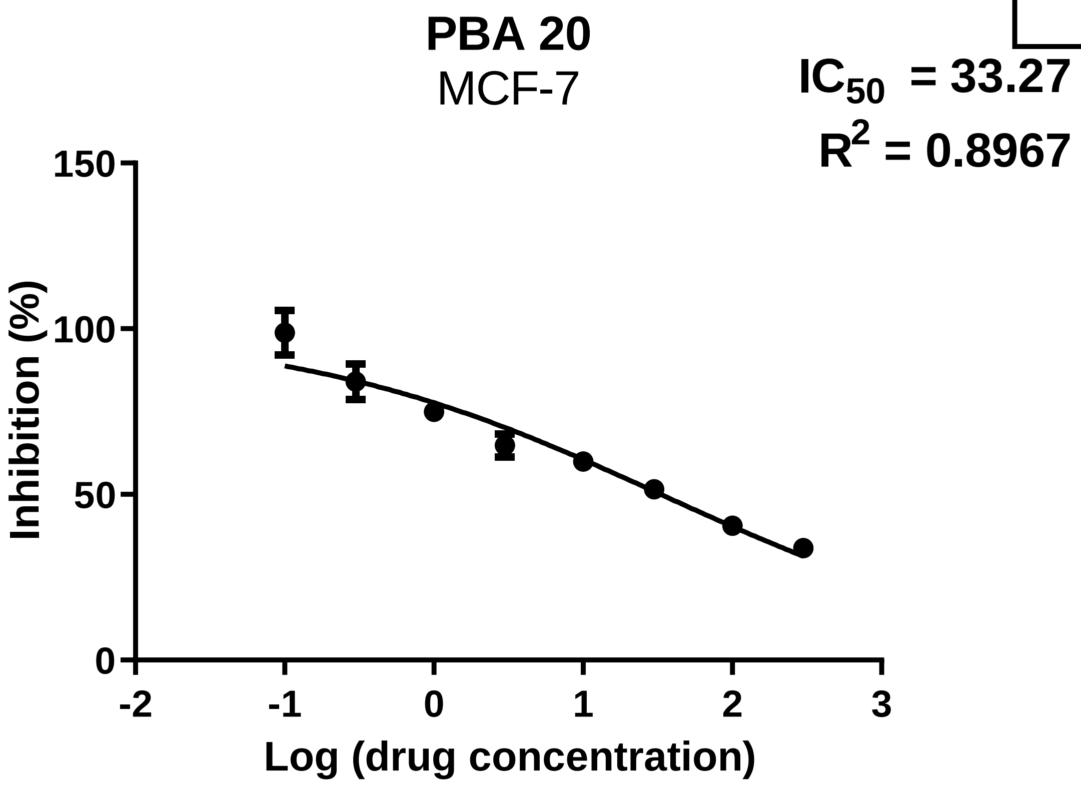

Cytotoxicity of 12b against MCF-7 cell line

Best-fit values	
LogIC50	1.887
HillSlope	-0.3699
IC50	77.03

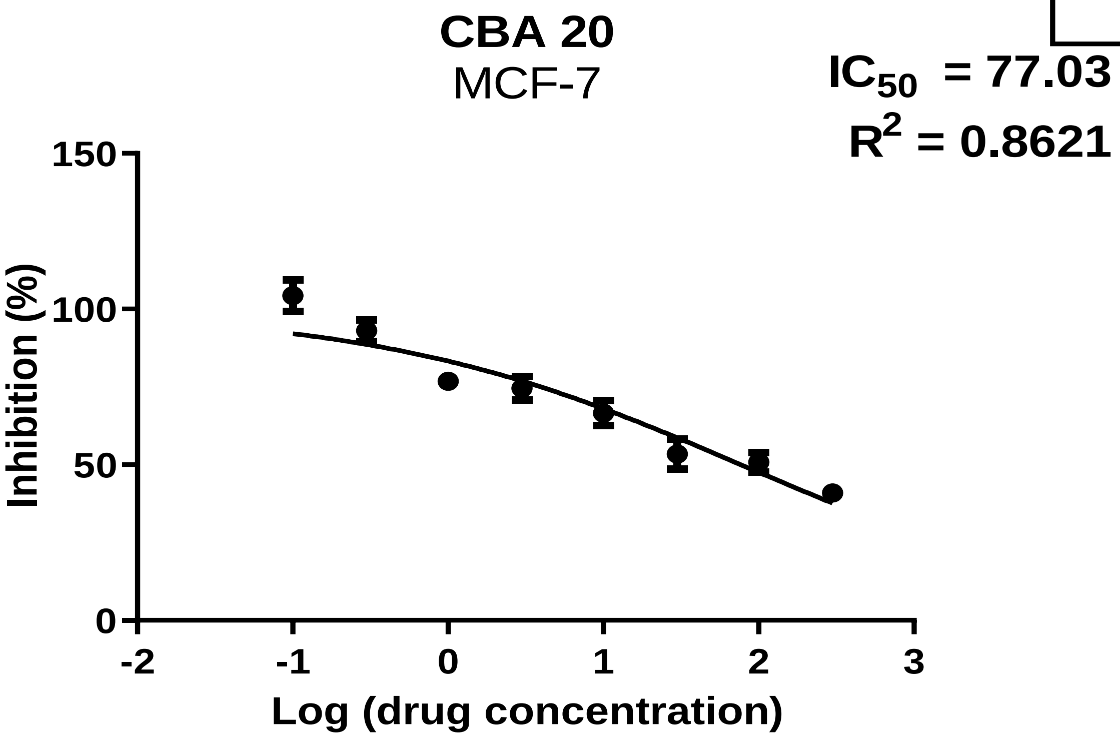

Cytotoxicity of 12c against MCF-7 cell line

Best-fit values	
LogIC50	1.565
HillSlope	-0.3796
IC50	36.72

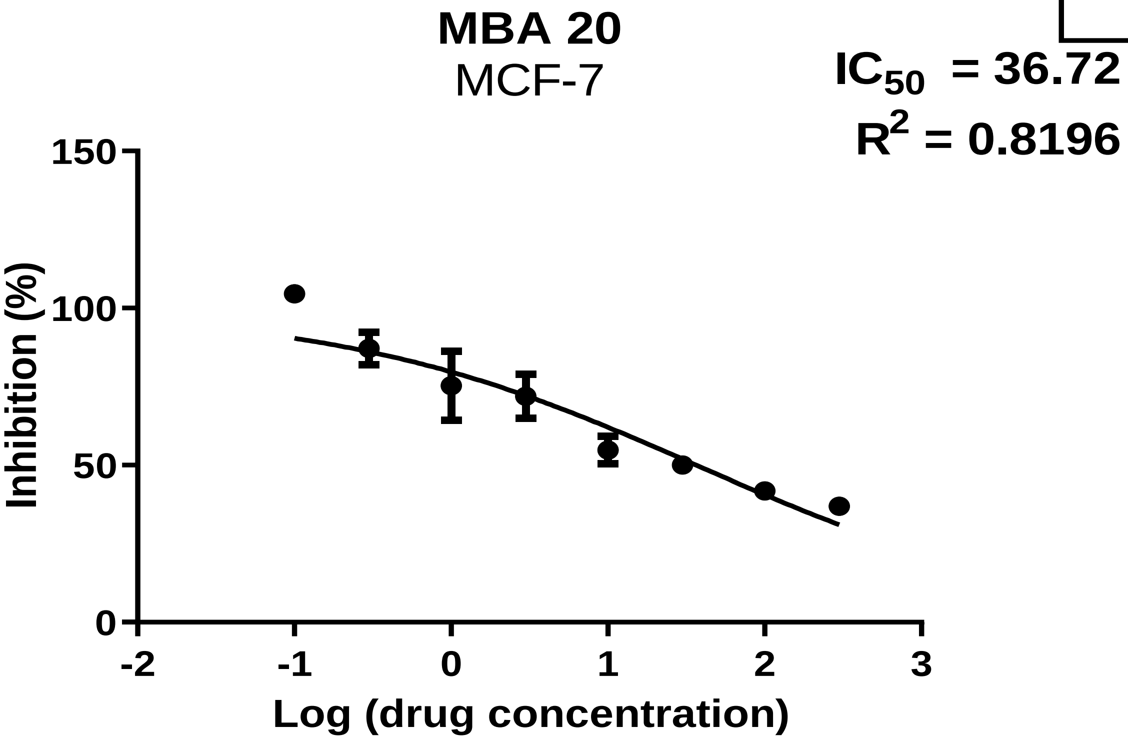

Cytotoxicity of 12d against MCF-7 cell line

Best-fit values	
LogIC50	1.644
HillSlope	-0.3746
IC50	44.09

PBA 35

MCF-7

IC_{50} = 44.09

R^2 = 0.7824

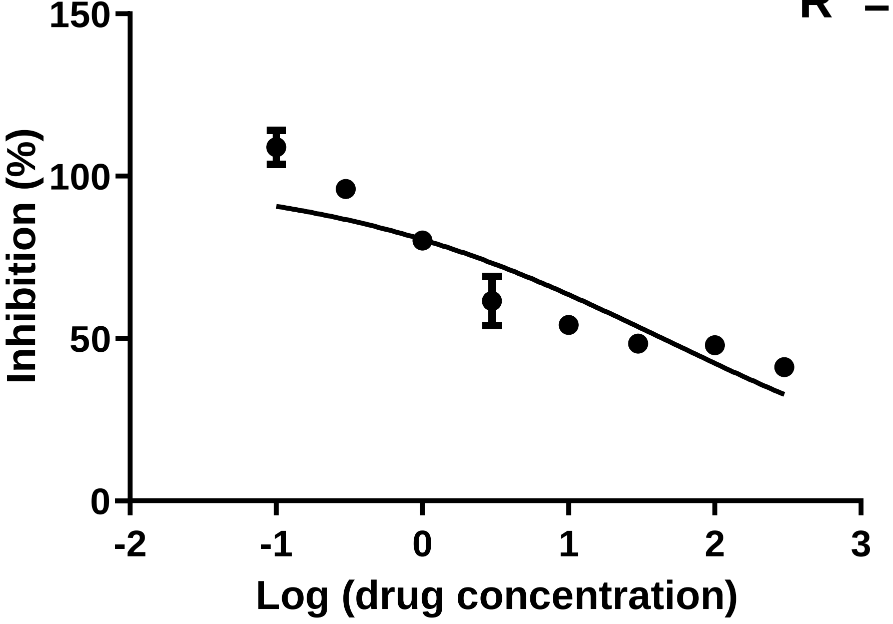

Cytotoxicity of 12e against MCF-7 cell line

Best-fit values	
LogIC50	1.794
HillSlope	-0.3981
IC50	62.29

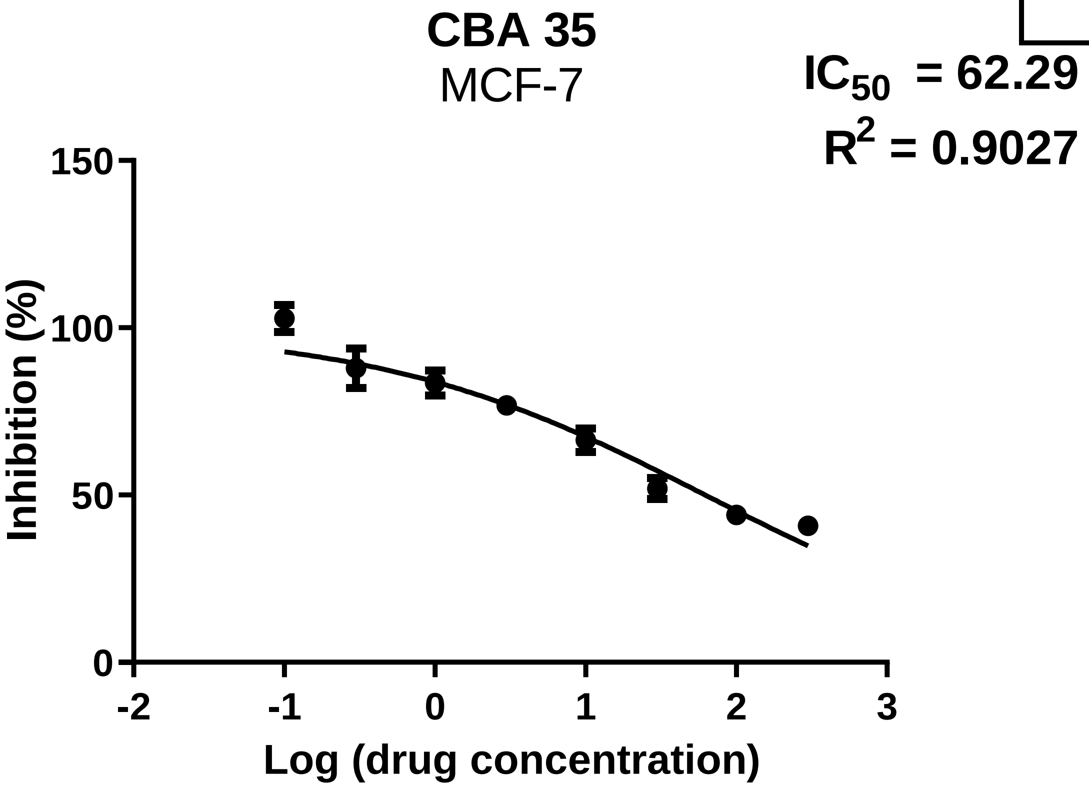

Cytotoxicity of 12f against MCF-7 cell line

Best-fit values	
LogIC50	1.353
HillSlope	-0.4193
IC50	22.54

IC_{50} = 22.54

R^2 = 0.9152

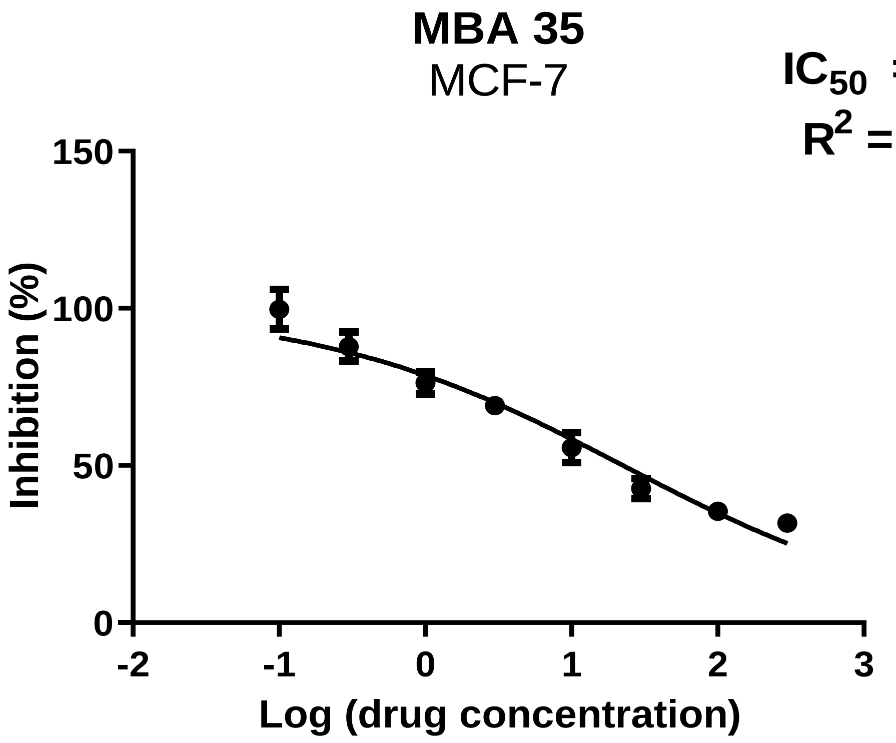

Cytotoxicity of 12g against MCF-7 cell line

Best-fit values	
LogIC50	1.724
HillSlope	-0.3460
IC50	53.01

IC_{50} = 53.01

$R^2 = 0.8893$

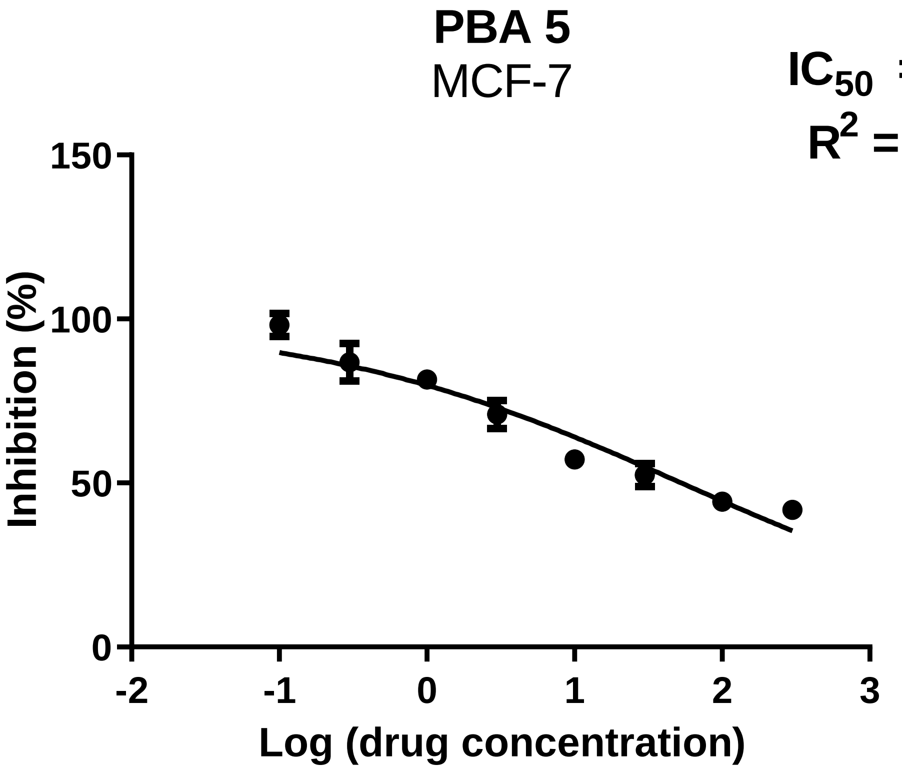

Cytotoxicity of 12h against MCF-7 cell line

Best-fit values	
LogIC50	1.933
HillSlope	-0.3889
IC50	85.62

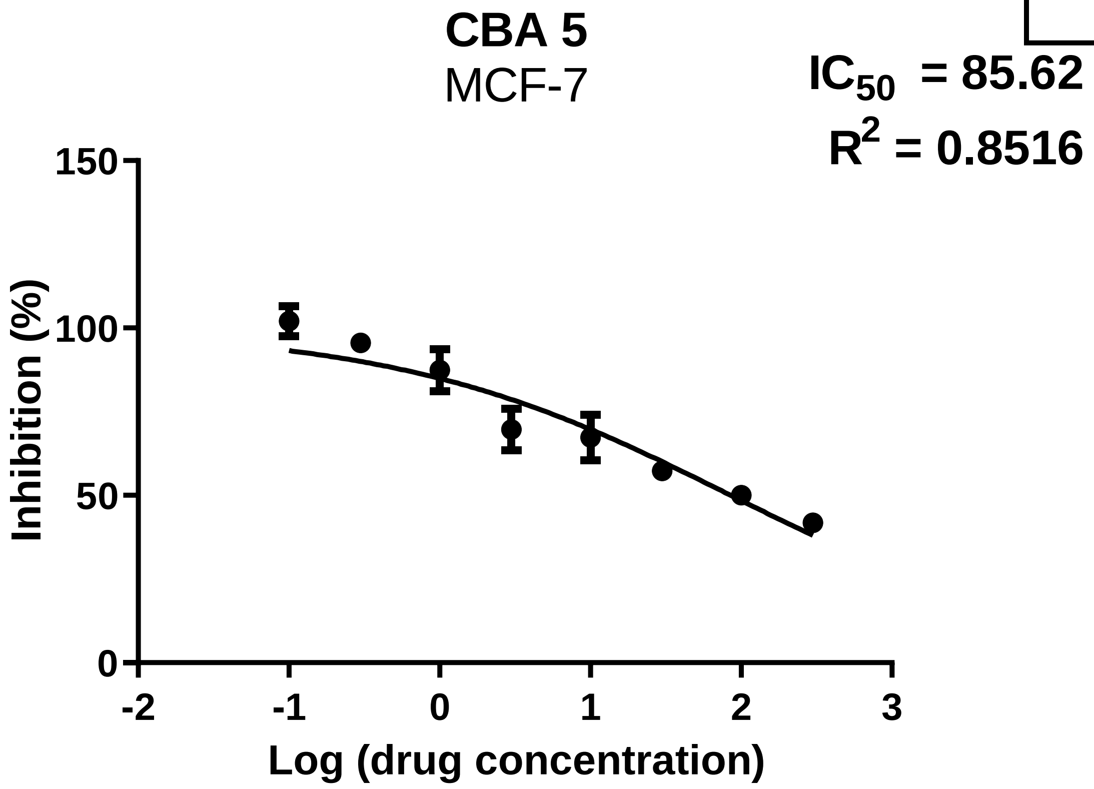

Cytotoxicity of 12i against MCF-7 cell line

Best-fit values	
LogIC50	1.447
HillSlope	-0.3379
IC50	27.99

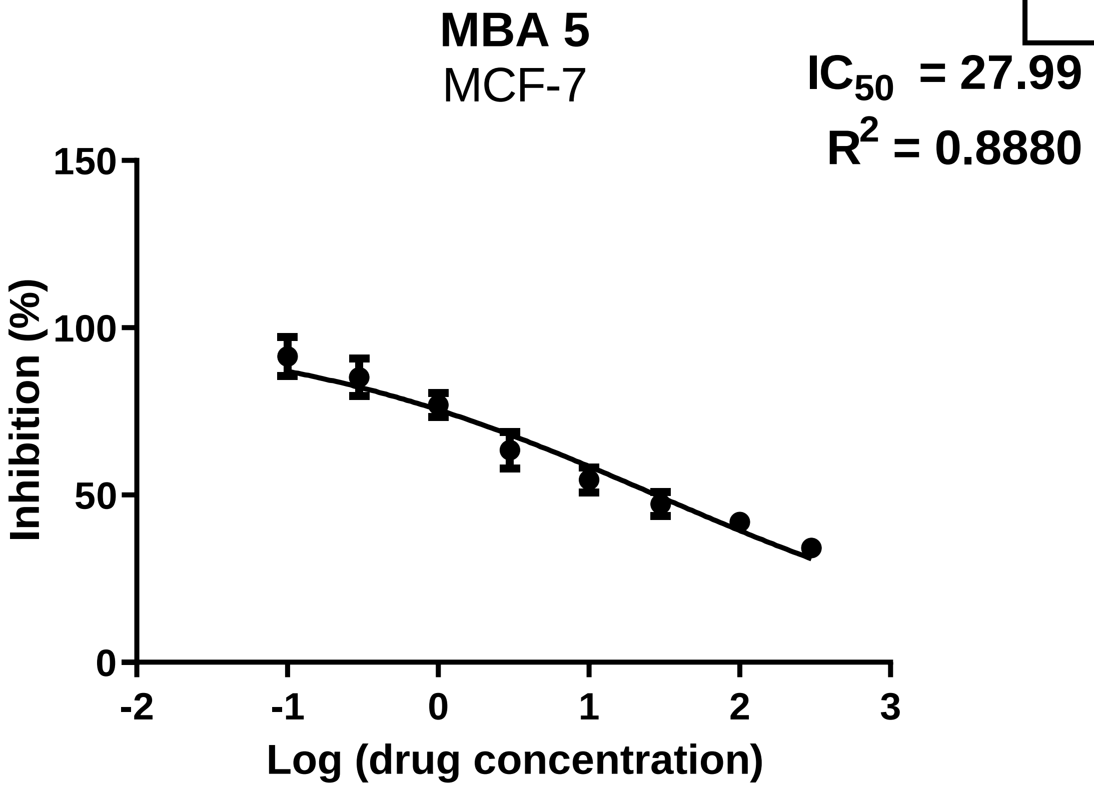

Cytotoxicity of 12j against MCF-7 cell line

Best-fit values	
LogIC50	1.526
HillSlope	-0.2945
IC50	33.61

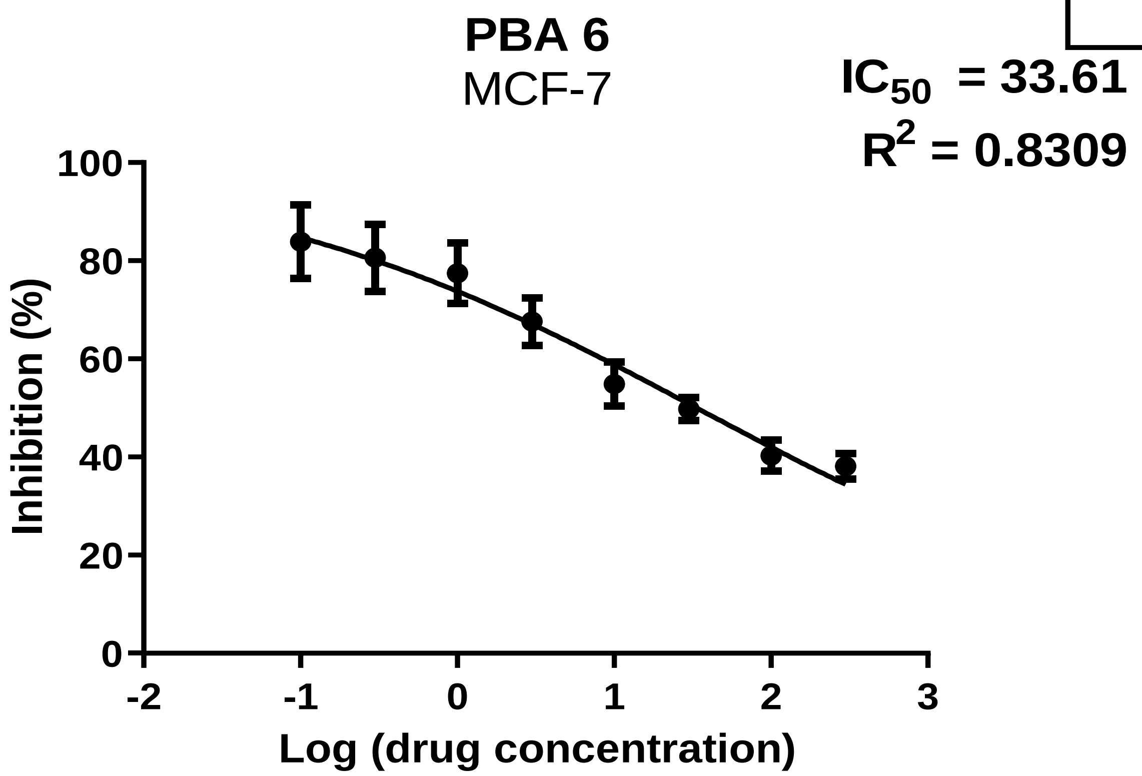

Cytotoxicity of 12k against MCF-7 cell line

Best-fit values	
LogIC50	1.938
HillSlope	-0.5191
IC50	86.62

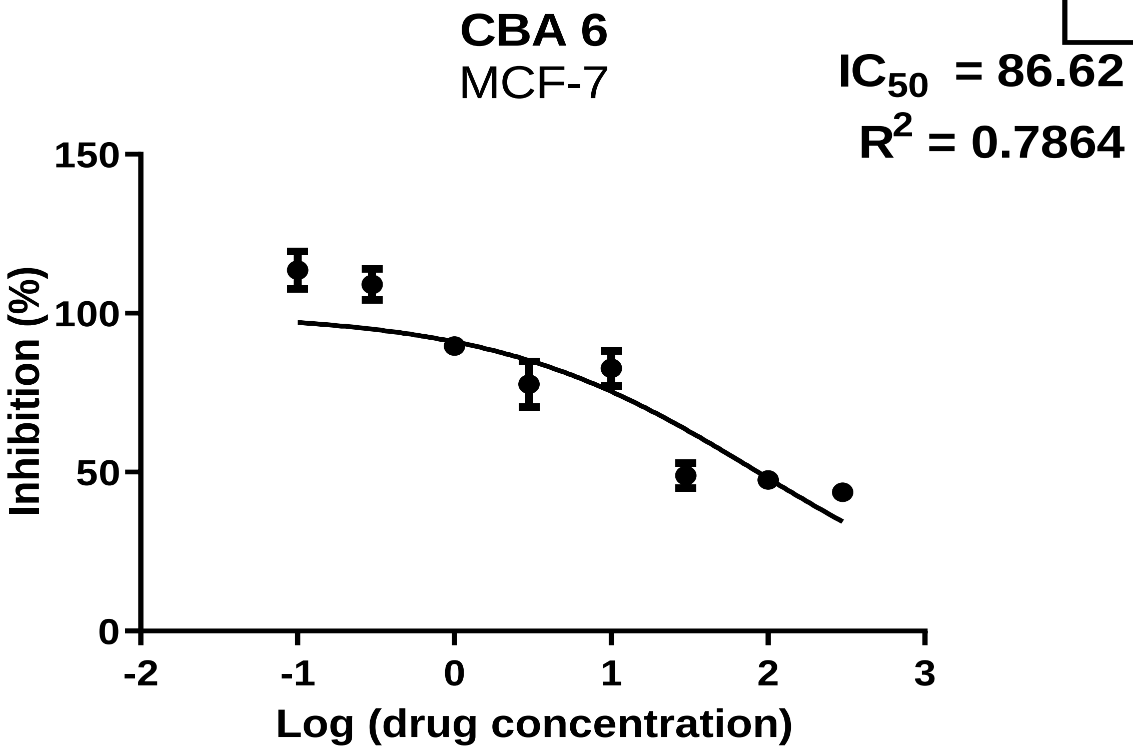

Cytotoxicity of 12l against MCF-7 cell line

Best-fit values	
LogIC50	1.182
HillSlope	-0.3666
IC50	15.21

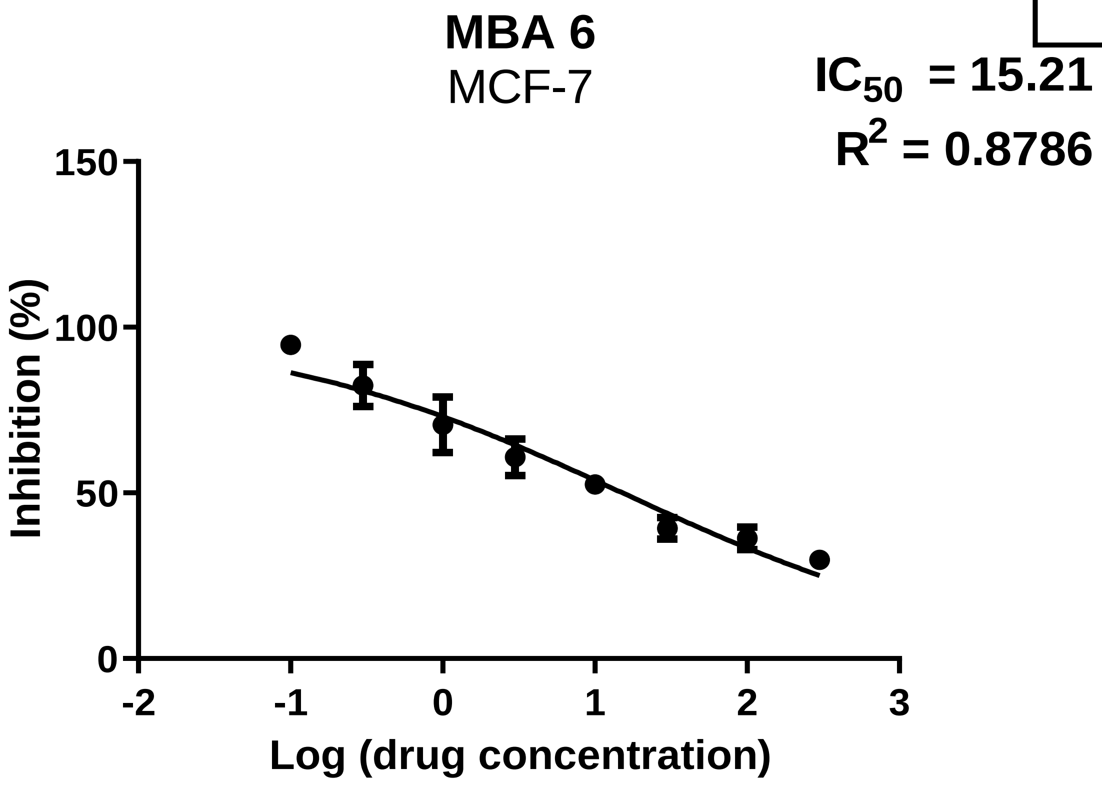

Cytotoxicity of 13a against MCF-7 cell line

Best-fit values	
LogIC50	1.511
HillSlope	-0.3328
IC50	32.47

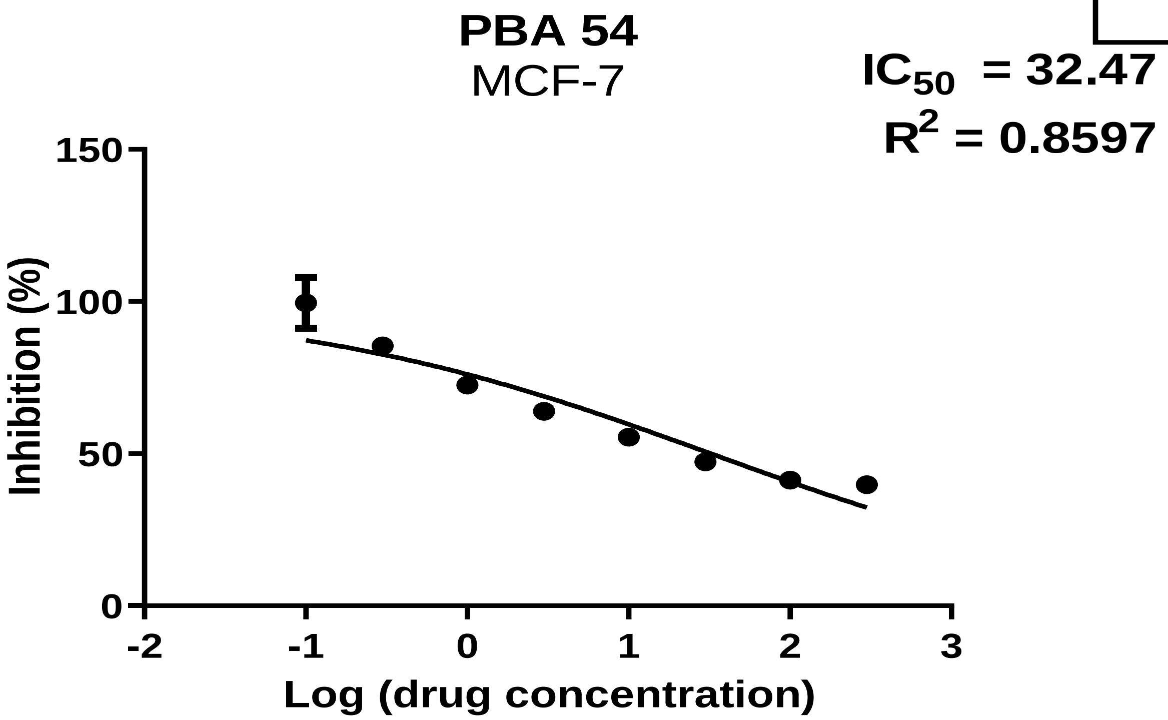

Cytotoxicity of 13b against MCF-7 cell line

Best-fit values	
LogIC50	1.420
HillSlope	-0.3721
IC50	26.31

IC_{50} = 26.31

R^2 = 0.8321

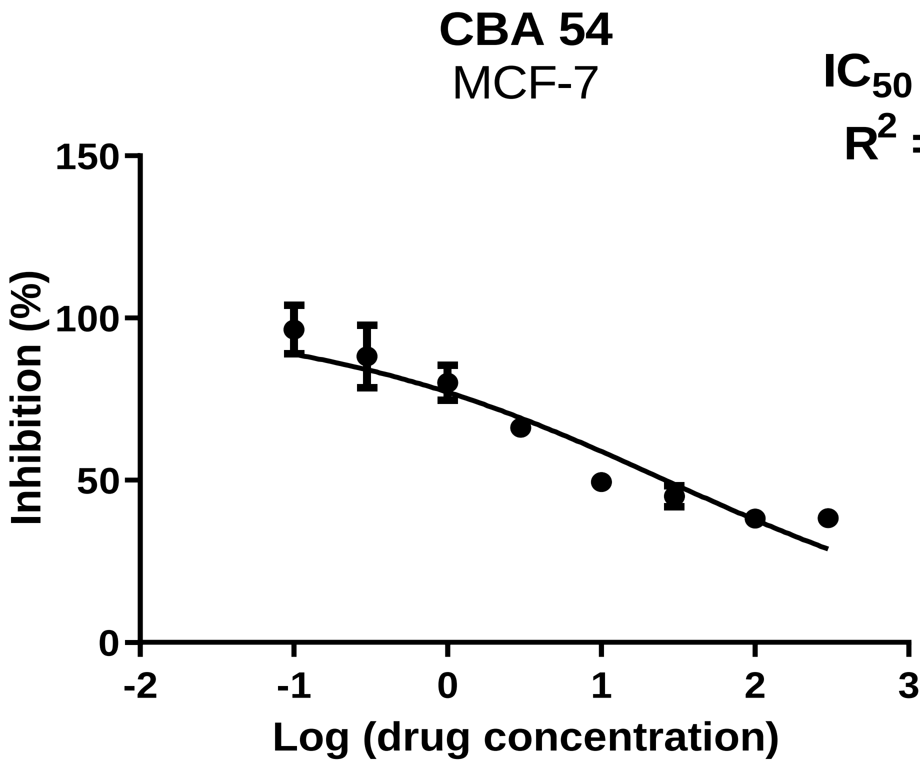

Cytotoxicity of 13c against MCF-7 cell line

Best-fit values	
LogIC50	1.725
HillSlope	-0.3505
IC50	53.13

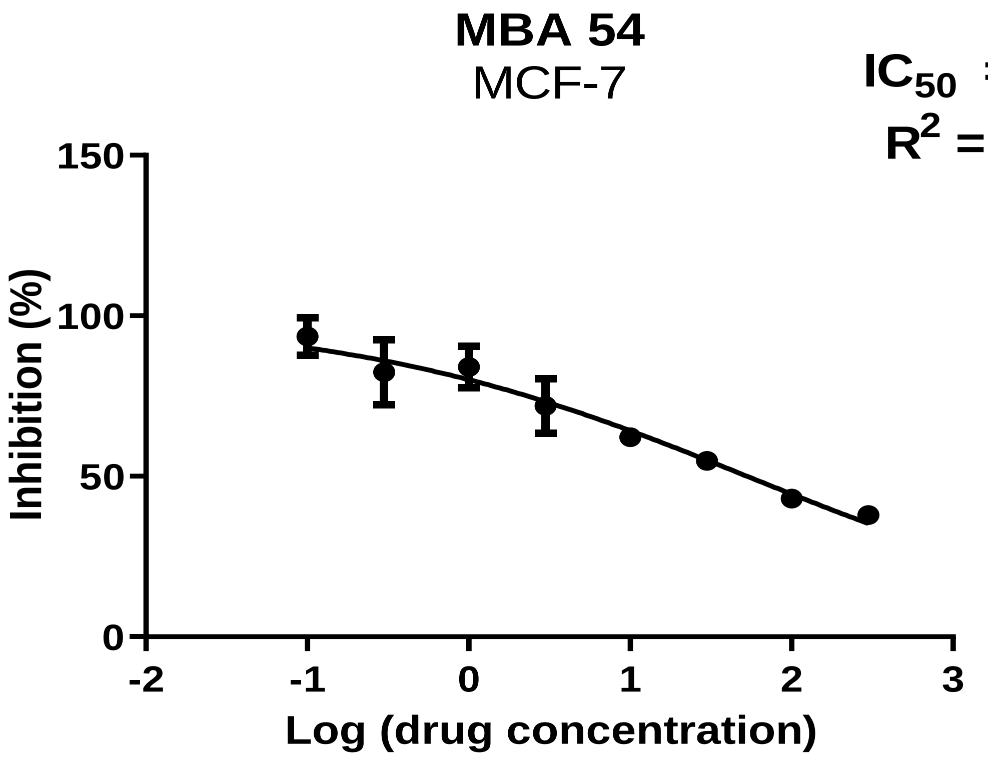

IC_{50} = 53.13

R^2 = 0.8224

Cytotoxicity of sorafenib against MCF-7 cell line

Best-fit values	
LogIC50	0.8104
HillSlope	-0.2596
IC50	6.462

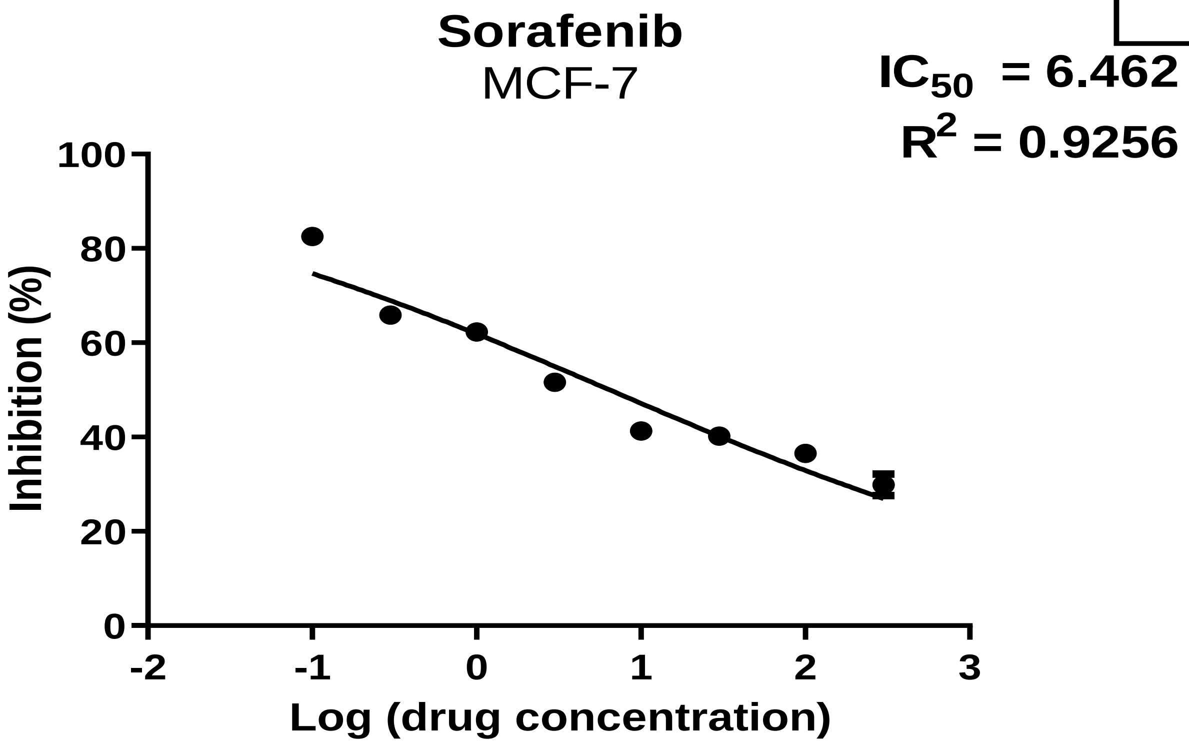

Raw data of the cytotoxicity of the most potent compounds against normal cell line (WI-38)

(WI-38)

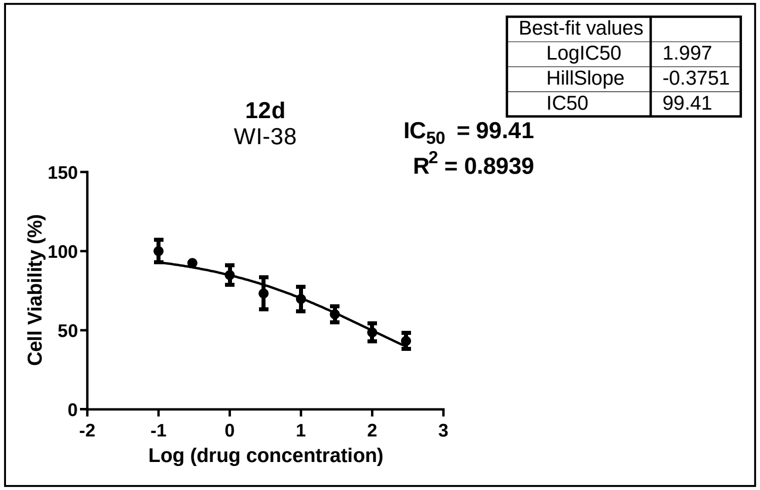

Best-fit values	
LogIC50	1.997
HillSlope	-0.3751
IC50	99.41

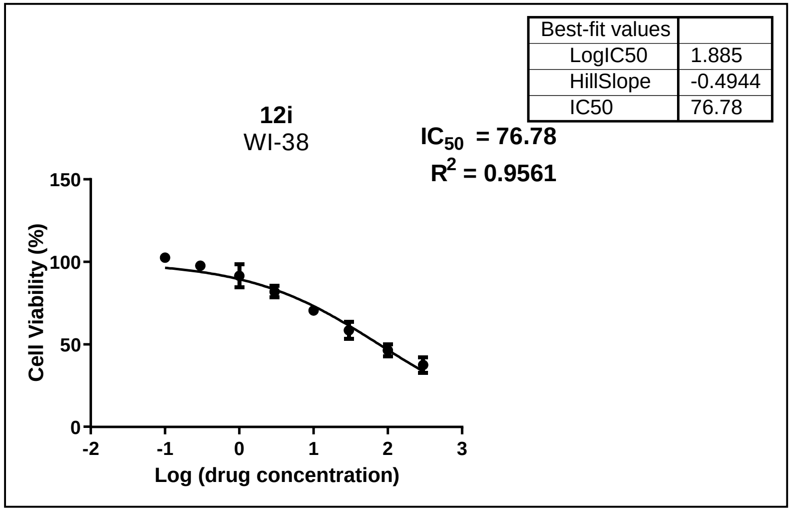

Best-fit values	
LogIC50	1.885
HillSlope	-0.4944
IC50	76.78

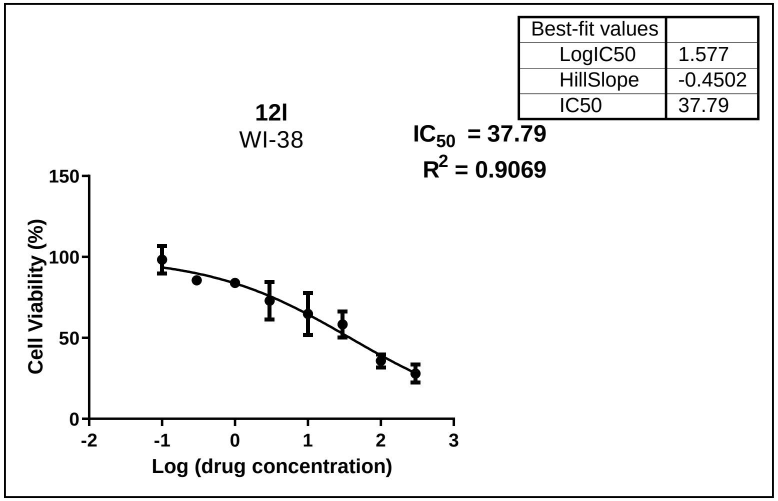

Best-fit values	
LogIC50	1.577
HillSlope	-0.4502
IC50	37.79

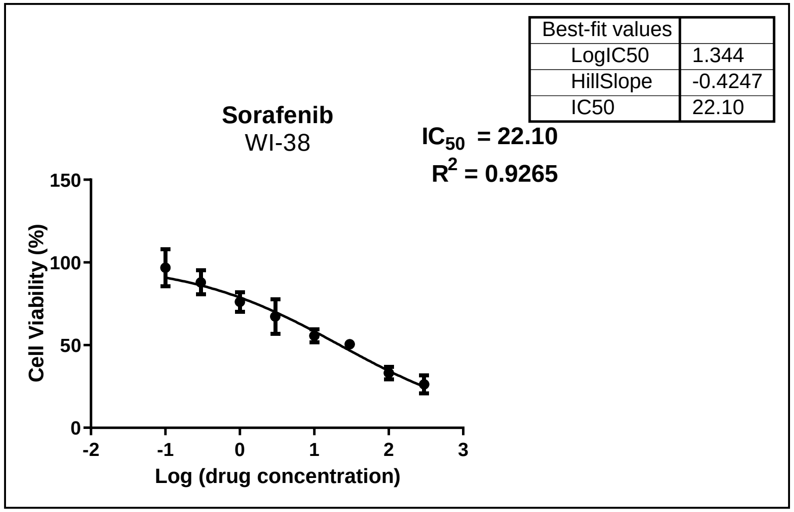

Best-fit values	
LogIC50	1.344
HillSlope	-0.4247
IC50	22.10

Raw data of the synthesized compounds against VEGFR-2

VEGFR-2 assay of compound 12d

Best-fit values	
LogIC50	2.289
HillSlope	-0.4166
IC50	194.6

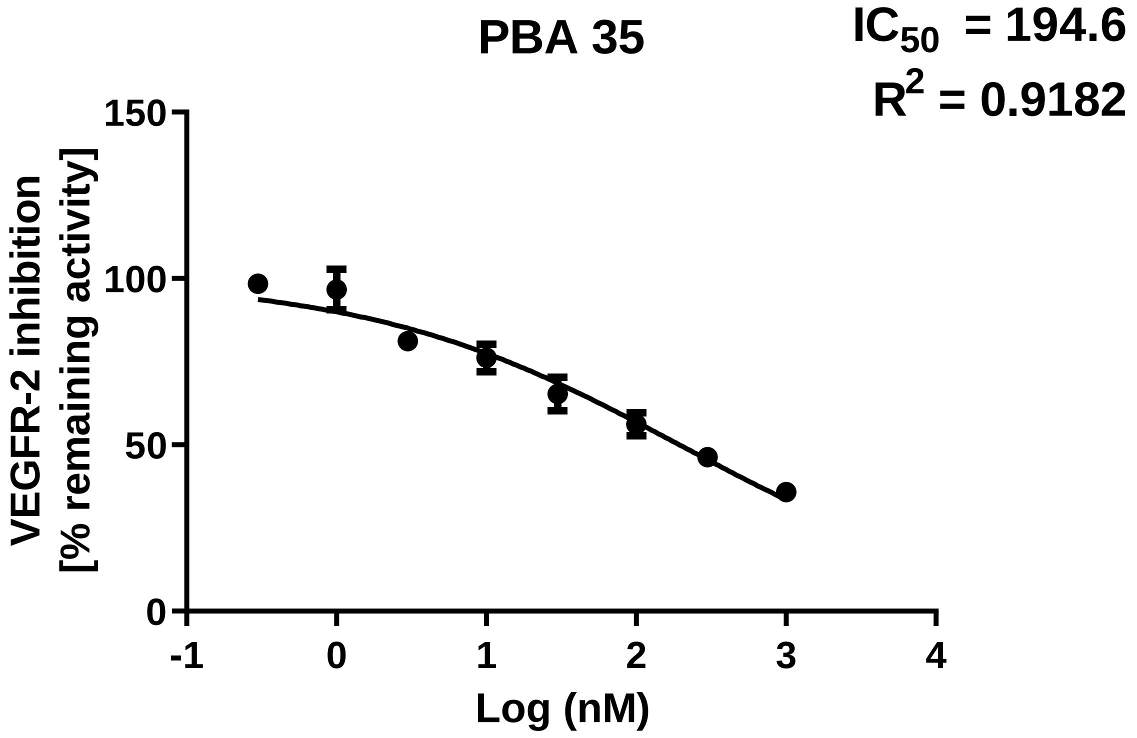

VEGFR-2 assay of compound 12f

Best-fit values	
LogIC50	2.423
HillSlope	-0.3283
IC50	264.9

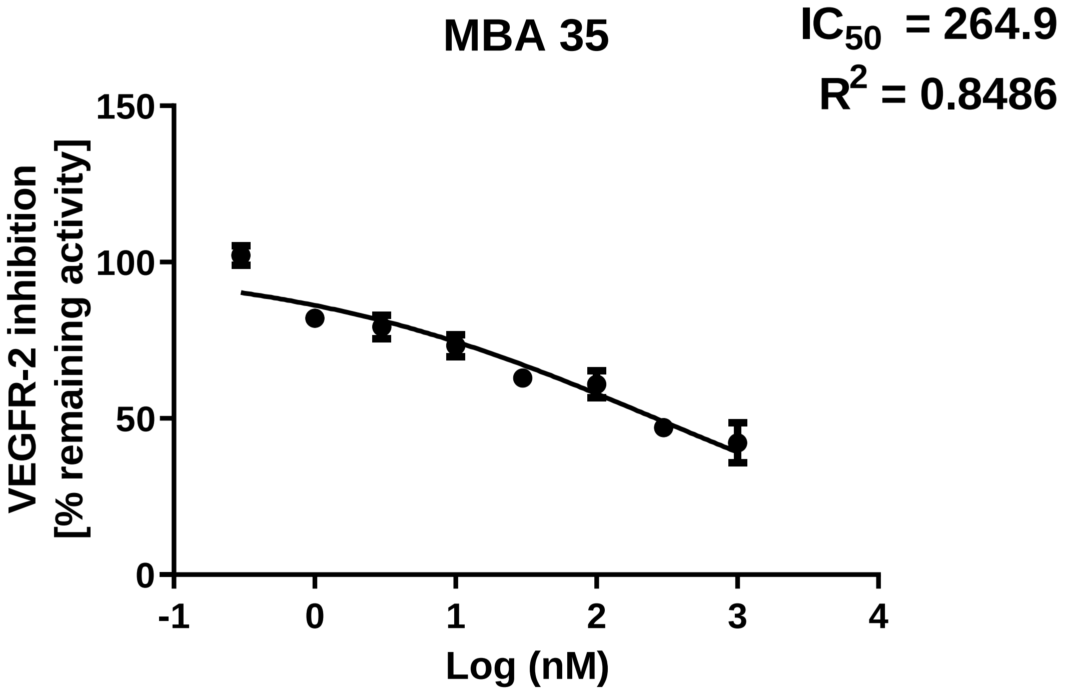

VEGFR-2 assay of compound 12i

Best-fit values	
LogIC50	2.190
HillSlope	-0.3818
IC50	155.0

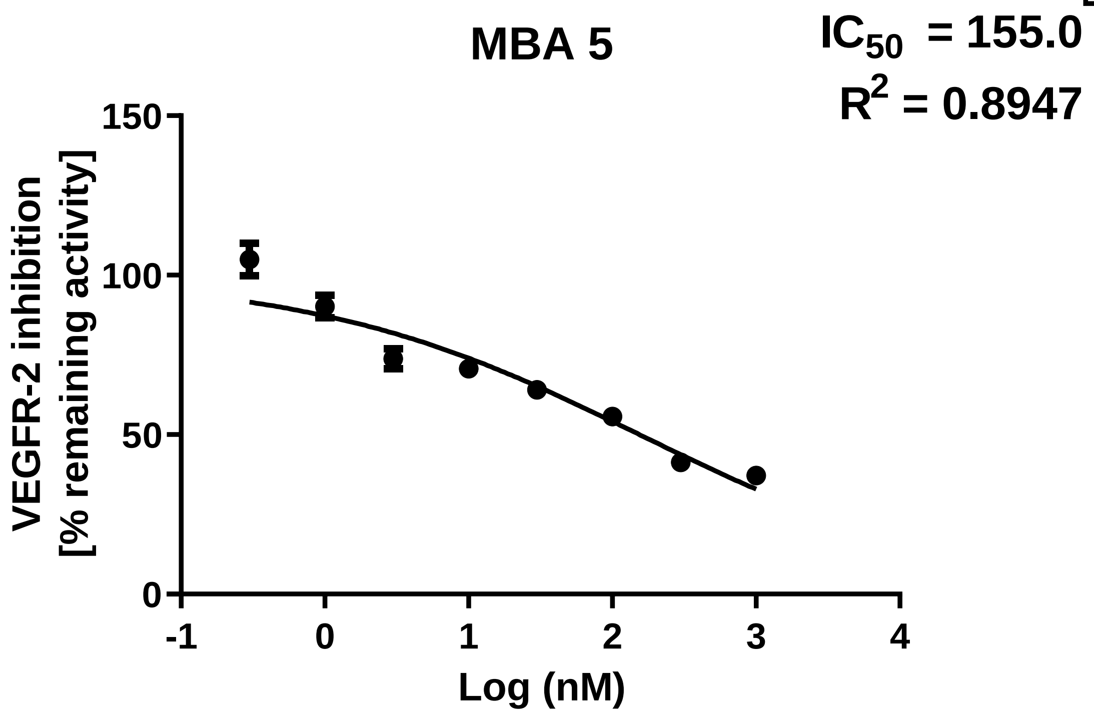

VEGFR-2 assay of compound 12l

Best-fit values	
LogIC50	1.988
HillSlope	-0.4614
IC50	97.38

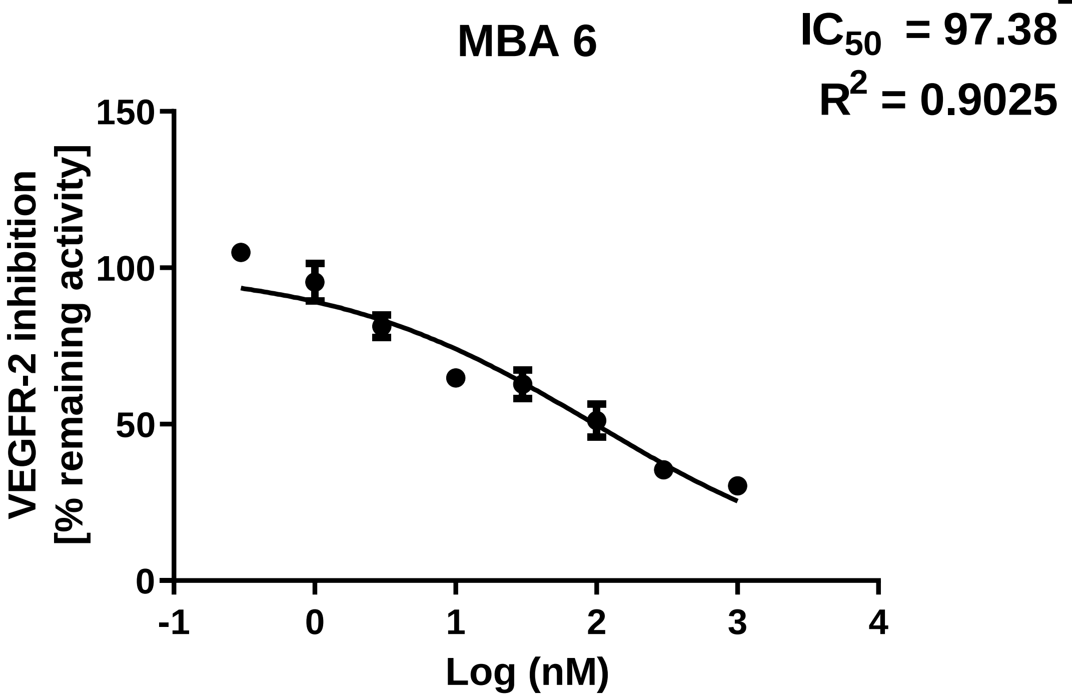

VEGFR-2 assay of compound 13a

Best-fit values	
LogIC50	2.428
HillSlope	-0.5089
IC50	267.8

PBA 54

IC_{50} = 267.8

R^2 = 0.9306

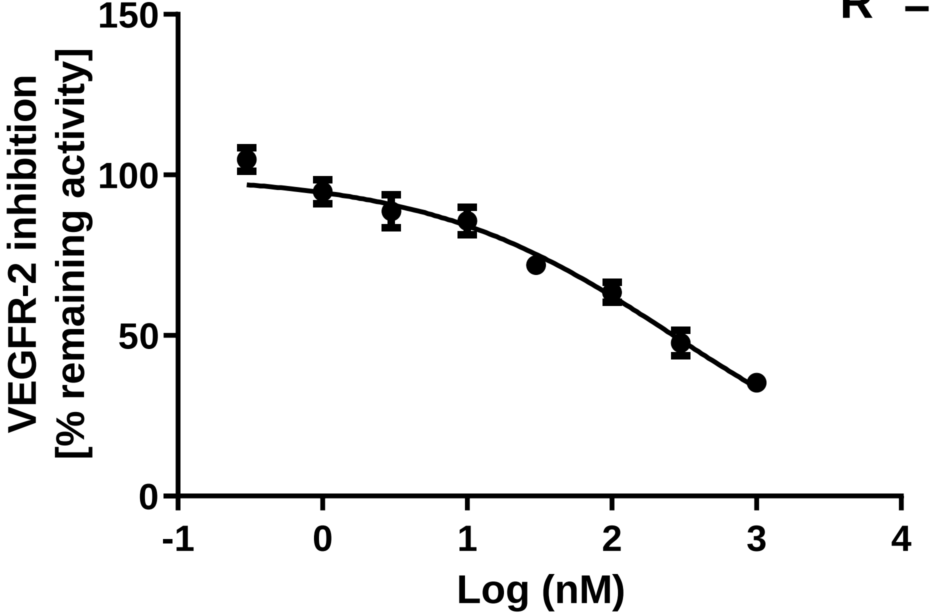

VEGFR-2 assay of compound Sorafenib

Best-fit values	
LogIC50	1.683
HillSlope	-0.4367
IC50	48.16

IC_{50} = 48.16

R^2 = 0.9391

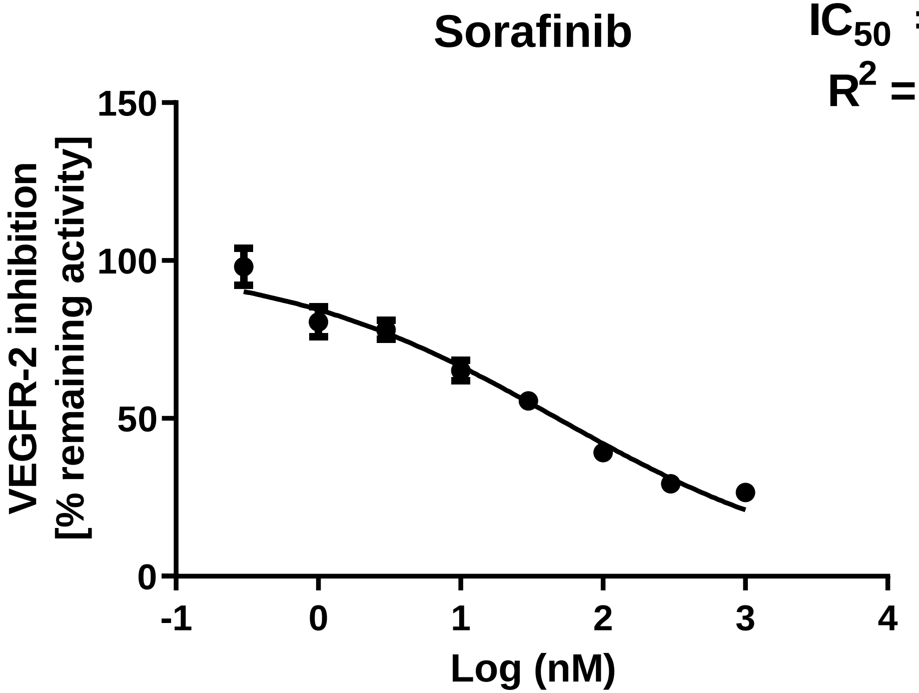

Supplement: Supplemental Material [file IENZ_A_2103552_SM9256.pdf]
